# Psychological Impact of Chemotherapy for Childhood Acute Lymphoblastic Leukemia on Patients and Their Parents

**DOI:** 10.1097/MD.0000000000002280

**Published:** 2015-12-28

**Authors:** Laila M. Sherief, Naglaa M. Kamal, Hadel M. Abdalrahman, Doaa M. Youssef, Mohamed A Abd Alhady, Adel SA Ali, Maha Aly Abd Elbasset, Hiatham M. Hashim

**Affiliations:** From Faculty of Medicine, Zagazig University, Zagazig, Egypt (LMS, HMA, DMY, MAA, ASAA, MAAA, HMH); Faculty of Medicine, Cairo University, Cairo, Egypt.

## Abstract

To assess the self-esteem of pediatric patients on chemotherapy for acute lymphoblastic leukemia (ALL) and psychological status of their parents.

The psychological status of 178 children receiving chemotherapy for ALL and their parents was assessed using parenting stress index (PSI) to determine the degree of stress the parents are exposed to using parent's and child's domains. Self-esteem Scale was used to determine the psychological status of patients.

The study revealed significant low level of self-esteem in 84.83% of patients. Their parents had significant psychological stress. PSI was significantly associated with parents’ low sense of competence, negative attachment to their children, feeling of high restriction, high depression, poor relation to spouse, high social isolation variables of parent's domains. It was significantly associated with low distraction, negative parents’ reinforcement, low acceptability, and high demanding variables of child's domains. Long duration of disease was the most detrimental factor among demographic data of the patients.

Chemotherapy for ALL has a significant impact on the psychological status of both patients and their parents with high prevalence of low self-esteem in children and high degree of stress in their parents.

## Abstract 2

Supplemental Digital Content is available in the text

## INTRODUCTION

Leukemia is the most prevalent pediatric malignancy^1,2^ with acute lymphoblastic leukemia (ALL) being the most common accounting for 75% of leukemia cases with about 2400 newly diagnosed children each year worldwide.^3^

Treatment of ALL requires long course chemotherapy ranging from 30 to 36 months with 20% possibility of relapse.^4^

Survival rate improvement in childhood cancer has led to increase in the numbers of parents who are caring for this population of children.^5^

The diagnosis of childhood leukemia and its stressful treatment, not only adversely impact the physical and psychological health of the children with leukemia, but also impose heavy psychological burden on their parents.^6–8^

The uncertainty of relapse, the possibility of serious infection, and the adverse effects of medications used negatively affect the psychological status of patients and their parents.^7^

Studies about the psychological impact of chemotherapy for ALL on patients and their parents in cultures outside North America and Europe are limited and they are especially rare in the Arab world;^9^ that is why we aimed to address this issue on Egyptian patients and their parents.

## PATIENTS AND METHODS

We carried a cross sectional study over a period of 2 years from October 2012 to November 2014, recruiting all children on chemotherapy for ALL and their parents from those attending the pediatric oncology service Zagazig University Hospitals and Tanta Cancer Center, Egypt.

Patients’ inclusion criteria: age: 6 to 18 years, sex: no sex limitation, conscious, able to communicate, in complete remission in maintenance phase of chemotherapy for ALL with the same maintenance protocols; modified CCG 1991 SR and CCG-1961 HR protocols; daily 6-mercaptopurine (75 mg/m^2^), weekly methotrexate (20 mg/m^2^ body surface area) orally, and monthly pulses of a single dose of IV Vincristine (1.5 mg/m2) and 5 days oral dexamethasone (6 mg/m^2^).

Parents’ inclusion criteria: age: no age limitation, able to communicate, psychologically stable, and accept to participate with their children in the study.

Children and parents having past or current history of psychiatric illness should be excluded.

Written informed consents were obtained from all parents for contribution with their children into the study. The study was approved by the research and ethical committees of the contributing hospitals.

Data were collected using the following methods:

1. Structured Interviewing Questionnaire (Appendix I) which included 2 parts:
2. The first part included the sociodemographic data of patients and their parents such as age, sex, residence, parents’ occupation, birth order of the patient, number of family members, family history of malignancy, ect.
3. The second part included ALL symptoms, signs, and treatment.
4. Parenting Stress Index (PSI) (Abidin, 1995)^10^ (Appendix II)

PSI is a reliable and valid 101-item parents’ report questionnaire that assesses stressful parents–children interaction. It can identify potentially dysfunctional parents–children systems. It is able to focus interventions into high-stress areas and predicts children's future psychosocial adjustment. It assesses parenting perception of the degree of stress related to various dimensions of the parenting role.

### Scale Has 2 Major Parts: the Child's Domain and the Parents’ Domain

#### Child's Domain

It includes 6 domains: hyperactivity/distraction (9 items), adaptability (11 items), reinforces parent (6 items), mood (5 items), demanding (9 items), and acceptability (7 items).

#### Parents’ Domains

It includes 7 domains: sense of competence (12 items), social isolation (6 items), attachment to their children (7 items), health status (5 items), role restriction (sense of having restriction in their life by their role as parents of diseased children) (7 items), depression (9 items), spouse relation (7 items).

### Scoring System

The scale consists of 101 items; each item is rated on a 5-point Likert scale format, ranged from strongly agree (5) to strongly disagree (1). The scores were summarized up and converted into percentage, and then the scores were converted into qualitative variables through categorization based on a cutoff point of 60%. A: Scoring of parenting stress regarding either of the child's domains or parent's domains was considered high with scores **≤**60% and low with scores <60%. B: Total scoring of parenting stress regrading both domains together was considered high with scores ≤60% and low with scores <60%.

#### (3) Self-Esteem (Rosenberg, 1965)^11^ (Appendix III)

This scale was developed by Rosenberg (1965)^12^ to measure self-worth and self-acceptance. It consists of 10 items filled by the researcher according to child observation. The list of statements rated on 4-point Likert scale.

### Scoring System

This ranged from 0 to 30, with high scores reflecting high self-esteem. The scale includes 4 items had reversed score, that is, strongly agree = 3, strongly disagree = 0. Based on the sum of the scores for the 10 items, the total score was calculated. Normal self-esteem was considered as ≤ 60% and low self-esteem as <60%.

#### Validity and Reliability Was Studied by Many Researchers (Appendix 4)

In the current study, before starting data collection the questionnaires of this study were distributed among group of experts in this field, statisticians, pediatric psychiatrists, pediatric oncologist, and socialist. Test–retest reliability was applied, the questioner proved to be strongly reliable. Pilot study was also conducted and no modifications were done.

## STATISTICAL ANALYSIS

Data were analyzed using SPSS (version 15.0., SPSS Inc, Chicago, IL). Statistical analysis was performed using the Student *t* test, corrected X2 test, or Fischer exact test, when appropriate. The results were expressed as counts and percentages for qualitative variables and as means or medians and ranges for discrete variables. A *P* value <0.05 was considered to be statistically significant.

## RESULTS

During the study period 213 children fulfilled the study inclusion criteria. Among them 35 parents refused participation in the study, and 178 parents accepted.

### Demographic Data of the Studied Children

The mean age of the participating children was 10.71 ± 4.39 years (yr), 49.0% were males and 51% were females, 59.0% reside in rural areas, 52.0% were second in order (mean 2.13 ± 0.85), 61% had 3 to 5 siblings and 49% had a family number ≥ 6 (Table 1).

**TABLE 1:**
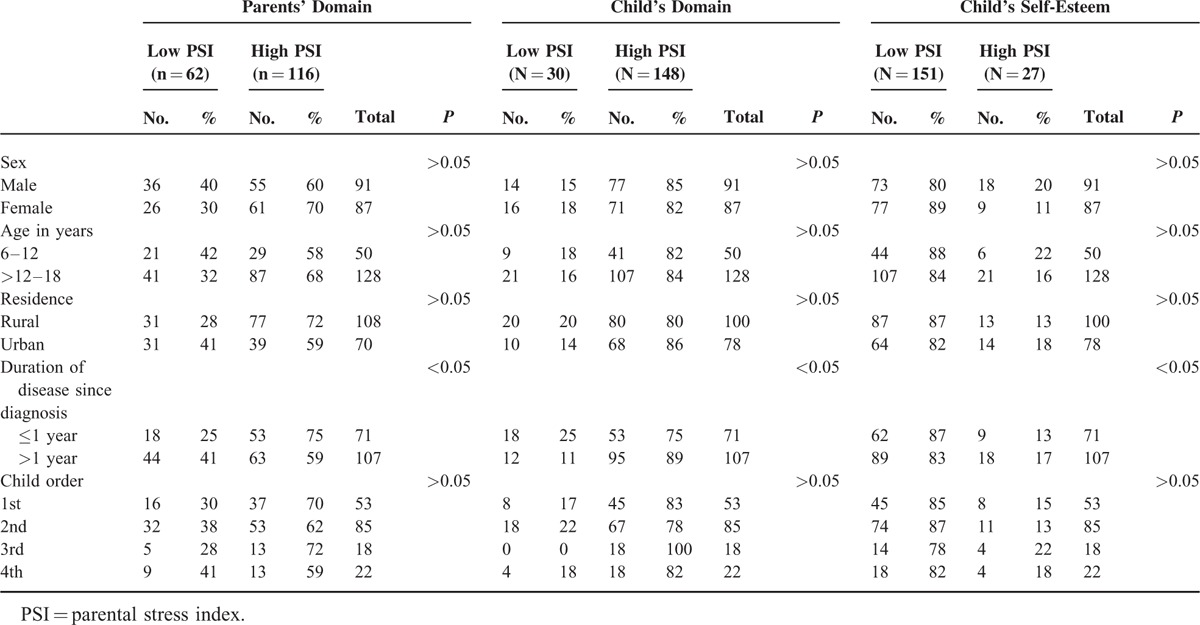
Demographic Data of Patients in Relation to Different Psychiatric Assessments

Fifty-eight percent of patients were diagnosed with ALL for >1 year (mean 1.91 ± 1.42).

Assessment of the effects of demograohic data of children on PSI related to parent's domains, child's domains, and self-esteem was statistically nonsignificant apart from significant association with the duration of disease since diagnosis (Table 1).

### Demographic Data of the Studied Parents

The mean ages of the fathers and mothers were 32.74 ± 8.45 and 30.11 ± 3.71 years respectively. Eighty-nine percent of fathers and 37% of mothers were working.

Assessment of the effects of parent's demograohic data on PSI related to parent's domains were statistically nonsignificant, while young parents and unemployed mothers had statistically significant high PSI related to child's domains (Table 2).

**TABLE 2:**
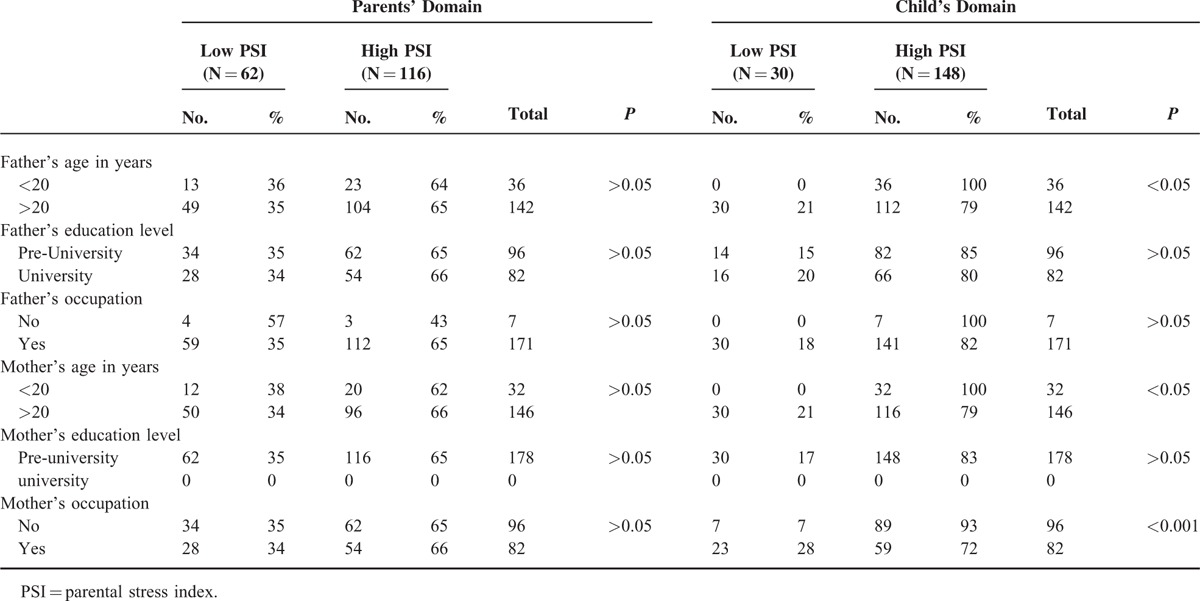
Demographic Data of Parents of Diseased Children in Relation to Different Psychiatric Assessments

### Effects of Child's Domains on PSI

**FIGURE 1:**
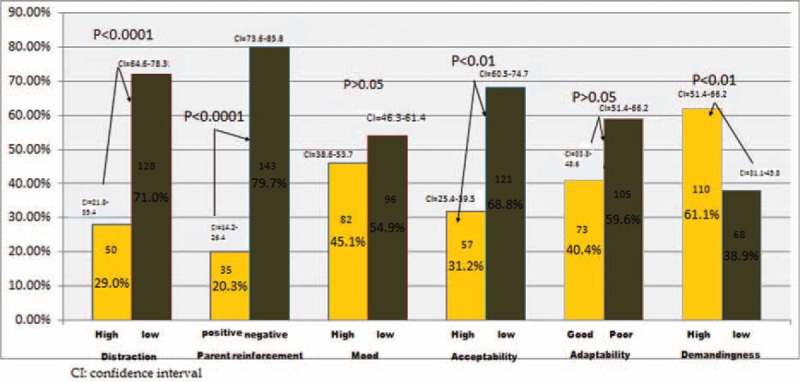
Parental stress in relation to children's domains.

Twenty-nine percent of participated children were highly distracted, 79.7% negatively reinforced their parents, 54.9% were low mood, 68.8% had low acceptability, 59.6% were poorly adapted, and 61.1% had high demanding which resulted in high parenting stress with PSI score of more than >60 (Fig. 1).

### Effects of Parent's Domains on PSI

**FIGURE 2:**
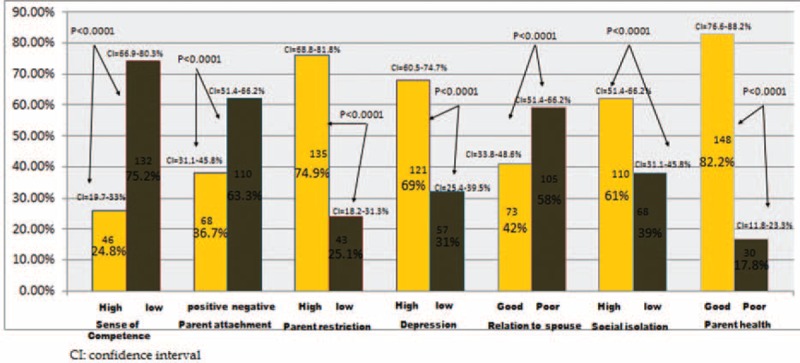
Parental stress in relation to parent's domains.

Around 75.2% of parents had low sense of competence, 63.3% were negatively (poorly) attached to their children, 74.9% felt high restrictions (being parents of a child with leukemia), 69.0% had high level of depression, 58.0% had poor relation with spouse, 61.0% had high social isolation, and only 17.8% had poor health. These factors were associated with high parenting stress with PSI score of more than >60 (Fig. 2).

### Impact of Disease Duration on PSI

**FIGURE 3:**
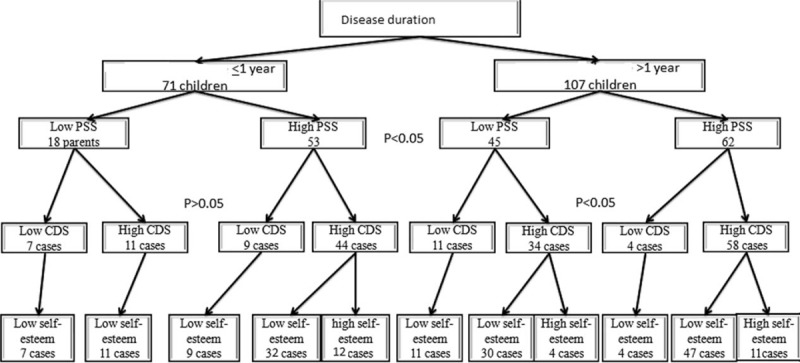
Impact of duration of disease on parental stress score (PSS), child domain score (CDS), and child self-esteem.

Figure 3 illustrates the impact of duration of disease on total parenting stress score (PSS), child domain score (CDS), and child self-esteem. Longer duration of disease >1 year is associated with a significant higher PSS and low self-esteem of diseased children (151/178).

### Child's Self-Esteem

Most of participated children (84.83%) had low level of self-esteem which was significantly colleralted with longer duration of disease (Table 1, Fig. 3).

Significant correlation was found between child's level of self-esteem and the child's acceptability of his illness, the degree of parents’ attachment to their children, the strength of spouse relationship to each other, and the degree of parental social isolation (Table 3).

**TABLE 3:**
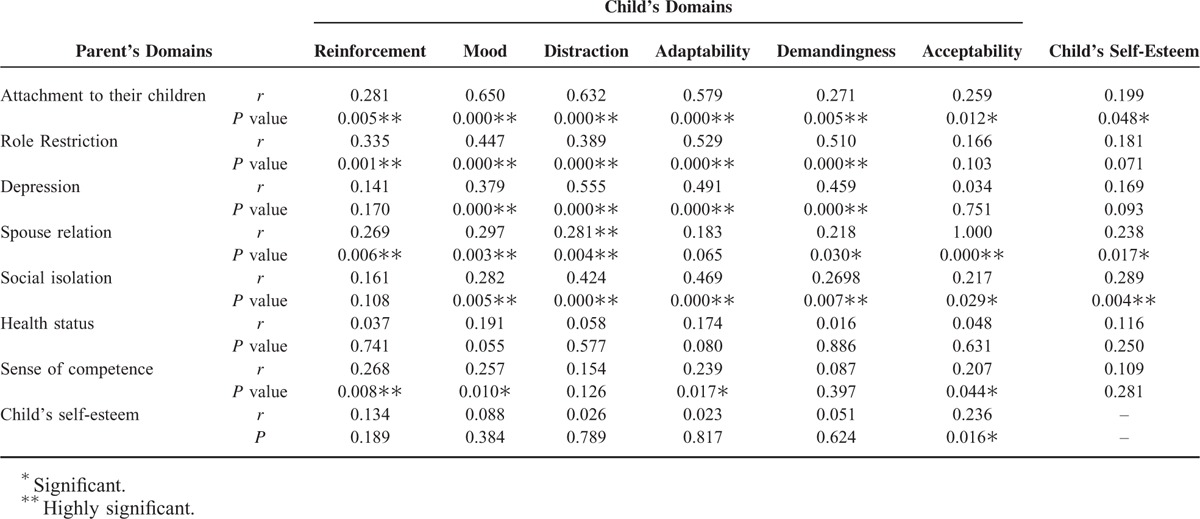
Correlation Between Different Studied Variables

### Correlations Between Parent's Domains Scores (PDS) and Child's Domains Scores (CDS) of Parenting Stress (Table 3)

Significant positive correlations were found between PDS of: attachment and role restriction, and CDS of parent's reinforcement, mood, distraction, adaptability and demandingness. Parent's depression and social isolation, and CDS of mood, distraction, adaptability and demandingness. Relation to spouse and CDS of parent's reinforcement, mood, distraction and acceptability. Parent's sense of competence and CDS of parent's reinforcement, mood, adaptability, and acceptability.

On the other hand, significant negative correlation was found between spouse relation and CDS of demandingness.

## DISSCUSSION

The diagnosis of childhood leukemia is a critical life event that impacts the psychological status of children and their parents.^12^ The current study studied the psychological impact of chemotherapy of ALL on pediatric patients and their parents using Rosenberg self-esteem scale and PSI respectively. Disease duration was the most influential factor. These findings were similar to those of Kyritsi et al^13^ in cancer patients who found significant correlation between the onset of cancer and self-esteem level.

Some demographic factors, including young parents and unemployed mothers, were found to cause high PSI (high CDS) which were in agreement with Dolgin et al^14^ and Chen et al^12^ respectively.

Parents of children with ALL experience considerable psychological burdens despite the high probability of cure and considerable advances of treatment protocols.^15^ Similar findings were observed in studies on childhood cancers where decreased positive mood and self-estees, increased sleeping difficulties, and behavioral problems were reported in patients.^16,17^

The major stressors for patients and their parents were treatment procedures, loss of control, hospital environment, relapses, fear of dying^18^; poor body image, ongoing lack of self-esteem, and difficulties in transition back into their social life.^19^ This agrees with our results where 84.83% of the ALL pediatric patients had low self-esteem, 54% had low mood, 68% had low acceptability, 59% were poorly adapted, and 62% were highly demanding.

We agreed with other researchers^14,20^ that higher level of parenting stress score is associated with lack of support from the other spouse and parents’ feeling of being socially isolated with restricted time for personal activities which in turn affect all aspects of child's domain of PSI.

Parents’ attachment to their diseased child, especially mothers who direct attention solely toward the diseased child, cause marital conflicts, impaired communication, and/or impaired self-care of parents.^21,22^ The changes in routine of daily life and sleep disturbance negatively affect mothers’ mood and initiate problems with the other partner.

Psychiatrists, social workers, and other members of the multidisciplinary oncology team should help pediatric patients and their parents cope with the diagnosis of ALL and its treatment.

## CONCLUSIONS AND RECOMMENDATIONS

Chemotherapy for ALL has a significant impact on the psychological status of both patients and their parents with high prevalence of low self-esteem in patients and high degree of psychological stress in their parents.

An integrated psychosocial support programs for patients and their parents by a multidisciplinary team work including pediatric oncologist, psychiatrist, social worker and a specialized nurse is a must to help them realize and cope with the stresses they face, answer their questions, listen to them patiently, help them to express their emotions, give explanations, advice, and support.

## Supplementary Material

Supplemental Digital Content
