# Supplementary material for: Psychological Impact of Chemotherapy for Childhood Acute Lymphoblastic Leukemia on Patients and Their Parents
Source: Medicine (Baltimore). 2015 Dec 28;94(51):e2280. doi: 10.1097/MD.0000000000002280 (PMC4697977; doi:10.1097/MD.0000000000002280)
Supplement: Supplemental Digital Content [file medi-94-e2280-s001.pdf]

APPENDIX (I)

MEDICAL AND DEMOGRAPHIC DATA:

Demographic data of the child:

1) Child's age: ....

2) Sex: ....

3) Residence: ....

4) Father's age: ....

5) Father's employment: ....

6) Mother's age: ....

7) Mother's employment: ....

8) Current Diagnosis: ....

9) Number of family members: ....

10) Number of siblings: ………

11) Child's order among his/her siblings: ……….

12) With whom the child comes to the hospital:

Father () Mother () One of the siblings (), one of the relatives ()

B) Medical history of the diseases child:

1. Duration of disease since diagnosis in the child:............................

2. Symptoms:…….

3. Chemotherapy:

Type…..

Way of administration: IV (), IM (), Oral (), intrathecal ()

Frequency of chemotherapy administartion: Once per week () twice a week ()

Family History:

1) Family history of malignancy: …

2) If yes: Who is the affected person? Father () Mother () Siblings () Others ()

APPENDIX (II)

The Parenting Stress Index - Standard Form

The PSI is a parent self-report, 101-item questionnaire, designed to identify potentially dysfunctional parent-child systems. The PSI focuses intervention into high stress areas and predicts children's future psychosocial adjustment. There exists a substantial body of published research linking PSI scores to observed parent and child behaviors and to child's attachment style and social skills.

The Parenting Stress Index - Short Form

The PSI-SF consists of 36 items derived from the PSI which comprise three scales: Parental Distress, Difficult Child Characteristics, and Dysfunctional Parent-Child Interaction. It is recommended that all PSI-SF users to consider using the regular PSI given that the savings of 10-15 minutes is not worth the loss of the information from the PSI subscales, each of which have established validity. Given the range of the variables measured by the regular PSI's subscales, treatment effects are more likely to be identified and treatment planning is facilitated.

PSI Scales - Total Score	
**Child Domain**	**Parent Domain**
Distractibility/Hyperactivity	Competence
Adaptability	Social Isolation
Reinforces Parent	Attachment to Child
Demandingness	Health
Mood	Role Restriction
Acceptability	Depression
	Spouse

Citation: Abidin, R.R. (1995). Parenting Stress Index, Third Edition: Professional Manual. Odessa, FL: Psychological Assessment Resources, Inc.

The most recent is 4th edition:

Parenting Stress Index (PSI-4), Fourth Edition By Richard R. Abidin, PhD - See more at: http://www.wpspublish.com/store/p/2925/parenting-stress-index-psi-4-fourth-edition#sthash.M8v0Uzw9.dpuf

The **Parenting Stress Index** (PSI-4), Fourth Edition, published by WPS for clinicians, educators and researchers, can be purchased online.

**Author**	Abidin, Richard R.
**Purpose**	"Designed to evaluate the magnitude of stress in the parent-child system."
**Publication Date**	1983-2012.
**Publisher**	Psychological Assessment Resources, Inc.
**Publisher address**	Psychological Assessment Resources, Inc., 16204 N. Florida Avenue, Lutz, FL 33549-8119; Telephone: 800-331-8378; FAX: 800-727-9329; E-mail: custsupp@parinc.com; Web: http://www4.parinc.com
**Publisher URL**	http://www4.parinc.com
**Reviewed In**	J. F. Carlson, K. F. Geisinger, & J. L. Jonson (Eds.), The nineteenth mental measurements yearbook.

WE USED THE PARENTING STRESS INDEX (PSI) STANDARD FORM: ANSWERED BY THE PARENTS

CHILD DOMAINS

Item	Strongly agree	Agree	Not sure	Disagree	Strongly disagree
**<u>First: Distraction/hyperactivity</u>**<br>1 when my child asks for something, he usually continues in his attempts to get what he wants.<br>2. My son (daughter) is active to the extent of overwhelming me.<br>3. It seems my son (daughter) is easily to get distracted.<br>4. If I compare my son (daughter) to most of the other kids, I find that he had difficulty focusing his attention.<br>5. My son (daughter) remains mostly for more than ten minutes playing with a game.<br>6. My son (daughter) spends a lot of time away from home more than I expected.					

7- My son (daughter) activity is much greater than I expected.<br>8- My son (daughter) shows upset and excessive resistance when wearing his clothes or taking a bath.<br>9-My son (daughter) is easily distractible away from thing he is doing.					
**Second: Reinforces parent (support of the child to his parents)**<br>10. It is rare that my son (daughter) do things to introduce pleasure or satisfaction for me.<br>11. I feel most of the time that my son (daughter) loves me and wants to be close to me.<br>13. Smiling of my son (daughter) to me is much less than I expected.<br>14. When I do something for my son (daughter), I feel that my efforts are not appreciated.					
**Third: Mood**<br>15. My son (daughter)'s screaming and raves:<br>• much less than I expected					

• Less than I expected<br>• much as I was expecting<br>• much more than I expected<br>• This seems to be mostly a case going on with him<br>16. Which of the following describes your child's best:<br>• mostly he/she likes to play with me<br>• In some cases, he/she likes to play with me<br>• usually does not like to play with me<br>• Mostly does not like to play with me<br>17. It's apparent that my child's screaming and fussing is more often than most children<br>18. When playing, my child often do not cheer or laugh<br>19. My son (daughter) usually wake up from sleep in a bad mood<br>20. I feel that my son (my daughter) moody and it is easy to become anxious.					

**Fourth: Adaptability**<br>21. It seems my son (daughter) is a little bit different from what I expected and this is something that bothers me sometimes<br>22. It seems that my son (daughter) to forget what they have learned in the past in some areas and bouncing back to do special things for children younger than their age.<br>23. I think that my son (daughter) doesn't learn quickly unlike most children<br>24 - I think that my son (daughter) is not smiling very much unlike most children<br>25. My son (daughter) does some things that bother me much<br>26. My son (daughter) does not have the ability to work as much as I had expected					
**Fifth: Acceptability**<br>27. My son (daughter) faces many difficulties in adapting to the changes					

that occur around him/her more than most kids<br>28-When something my son (daughter) doesn't like happens, he/she has a very strong reaction.<br>29. The presence of my son (daughter) with other people is usually a big problem<br>30. My son (daughter) became annoyed for the simplest things<br>31. My son (daughter) easily notice high sounds and bright lights, and respond to them more than necessary<br>32. To build a system in sleep or eating for my son (daughter) was much harder than I expected<br>33. My son (daughter) usually avoids playing with a new toy for some time before he/she starts to play with it.<br>34. It is difficult for my son (daughter) to get used to the new things and it takes him a long time.<br>35. My son (daughter) seems not satisfied when he meets with people					

who are strangers<br><br>36. When my son (daughter) is in a state of tension or distress, it is:<br><br>1. Easy to calm him down<br><br>2 - difficult to calm him down more than I expected<br><br>3. It is very difficult to calm him down<br><br>4. does not help anything I'm doing in calm him down<br><br>37. I have found that when I ask my son (daughter) to do something or stop doing something, this demand is:<br><br>1. more difficult than I expected<br><br>2-difficult somehow than I expected<br><br>3-difficult as I was expecting<br><br>4-a little bit easier than I expected<br><br>5-much easier than I expected					
**Sixth: Demandingness (The frequent claim and urgency)**<br><br>38. Your son (daughter) does some things or behaviors that bother you.					

Think carefully and count the number of these things or behaviors, such as that he wasted his time or hesitate to his duties, disobey orders or directions, compulsive activity, nuisance or interrupts others while talking or working, quarrel, moaning and sobbing, etc.<br>You have to write the number of these stuff or behavior as follows:<br>1. 1 to 3<br>2. From 4 to 5<br>3. From 6 to 7<br>4. From 8 to 9<br>5. More than 10<br>39. When my son (daughter) screams, it usually takes:<br>1. less than two minutes.<br>2. From 2 to less than 5 minutes.<br>3. From 5 to less than 10 minutes.<br>4. from 10 to less than 15 minutes.					

5. more than 15 minutes.<br>40. My son (daughter) does some things or acts that cause a lot of distress and anxiety for me.<br>41. My son (daughter) is exposed tp more health problems than I expected.<br>42. The older my son (daughter) and the more he/she becomes dependent on him/herself, I find myself more concerned that he/she will be exposed to harm or fall in a problem.<br>43. My son (daughter) became a trouble for me more than I expected.<br>44. It seems that care of my son (daughter) is much more difficult than most children.<br>45. My son (daughter) is always attached to me.<br>46. My son (daughter) imposes demands on me more than most of other children.					

PARENTAL DOMAINS

Item	Strongly agree	Agree	Not sure	Disagree	Strongly disagree
**First: Sense of Competence**<br>47. When my son (daughter) was diagnosed with this disease, I was in doubt about my ability to perform my duties and my obligations as a mother (or father)<br>48. When I became a father or (mother), this was more difficult than I thought.<br>49. I feel my competence when I take care of my son (daughter)<br>50. I can not make decisions without help<br>51. I has lot of problems related to raising children more than I expected<br>52. I feel my success most of the time when I try to make my son do something or stop doing something					

53. Since I got my last son, I found myself unable to give good care for him as I thought to do, I Need Help<br>54-mostly I feel I cannot treat things properly<br>55. When given careful consideration to myself as a mother (or father), I think:<br>1. I can tackle anything can happen<br>2. I can tackle most things sound way to some extent<br>3. Although in some cases, I have my doubts in my ability to tackle most things, but I find that I can tackle them without any problems<br>4. I have some doubts about my ability to handle stuff<br>5. I do not think at all that I treat things properly.<br>56. I feel:<br>1. A Very good mother (father)<br>2. Better than most mothers (fathers)<br>3.Like most mothers (fathers)					

4.I face some difficulties or problems related to my role as a mother (father)<br>5.I'm not that good in doing my role as a mother (father)<br>57. What is the highest level of education you and your spouse had reached:<br>For the mothers:<br>1. Primary Education<br>2. Elementary education<br>3. Secondary education or secondary technical or medium certificate<br>4. University education<br>5. After Graduate University<br>58. What is the highest level of education you and your spouse had reached:<br>For the fathers:<br>1. Primary Education					

2. Elementary education<br>3. Secondary education or secondary technical certificate or medium certificate<br>4. University education<br>5. After Graduate University<br>59. I cannot make decisions without help<br>60. I have a lot of problems related to raising children more than I expected.					
**Second: Attachment: The emotional bond to the child**<br>61. To what extent is it easy for you to understand what your child wants or needs<br>1. Very easy<br>2. Easy<br>3. Somewhat difficult<br>4. Very difficult					

5. I cannot usually understand or I identify what problem he is facing<br><br>62. It takes long time from parents to have the feelings of warmth and tenderness towards their children<br><br>63. I expected to have feelings of warmth and tenderness towards my son more than I have and this is annoying me<br><br>64. Sometimes my son do things bothering me because I feel as if I'm just a way or instrument for him<br><br>65 - When I was young, I did not feel comfortable at all that I gave birth to a child with chronic disease or to take care of him<br><br>66. My son wants and needs me more than what he wants or needs from other people.<br><br>67. The number of what I now have from children is so much.					
**Third: (Role of Restricts): restrictions of parental role**<br><br>68. I spend most of my life in that joyful work for my son.					

69. I find myself gave a lot of my life to meet the needs of my children more than I expected.<br>70. I feel like I impasse because of my responsibilities as a mother (father).<br>71. I often feel that he necessary needs for my son (daughter) controls my life.<br>72. Since I gave birth to my son (daughter), I became unable to do new and diverse things.<br>73. Since my child was diagnosed with this disease, I feel in most cases that I am unable to work on the things which I like to do.<br>74. It is difficult to find a place in our house where I can be alone with myself.					
**Fourth: Depression**<br>75. When I look at myself as a mother (father), I mostly have a sense of guilt or feeling bad about myself.					

76. I'm not happy by what I bought for myself from clothes in the recent period.<br>77. When my son acts improperly or overly induces a state of agitation or chaos, I feel my responsibility for that. As if I did not do anything properly.<br>78. I feel with every time my son does something wrong, that in fact it was my fault.<br>79. I often feel guilty about the way I feel about my son.<br>80. There are a few things that make me feel worried about my life.<br>81. I felt sadness and depression more than I expected after knowing my son's disease.<br>82. I feel guilty when I get angry of my son and that's what bothers me<br>83. One month after my son was diagnosed with the disease, I noticed that I felt sad and depressed more than I expected.					

**Fifth: Relation of Spouse (the relationship between the spouses)**<br>84. I've noticed that since my son was diagnosed with the disease, my husband (wife) does not give me help as much as I expected.<br>85. As a sequel of my son’s diseases problems happened in my relationship with my husband (wife) more than I expected<br>86. Since my son was diagnosed with the disease, I and my husband (wife) became no longer share together in doing many things.<br>87. Since my son was diagnosed with the disease, I and my husband (wife) became no longer spend a lot of time with each other in contrary to what I expected.<br>88. I lost my interest in sex since my son s was diagnosed with the disease.<br>89. It seems that the problems with relatives have been rising after we got our diseased child.					

90. The presence of children had increased the cost of living more than I expected.					
**Sixth: Social Isolation**<br>91. I feel lonely and without friends.<br>92. When I go to a party, I usually expect that I will not rejoice.<br>93. I no longer care of people as I used to do.<br>94. I feel that people who are in my age do not like my company in particular.<br>95. When I have problems with the care of my son I can resort to some people for help or advice.<br>96-since I had children, the chance to see my friends and to make new friends declined.					
**Seventh: parent health (the health of the parents):**<br>97. During the past six months, my health was more affected than usual or I had more aches and pains than I have under normal					

circumstances.<br><br>98. I feel that my health is good most of the time.<br><br>99. The existence of a child I have, led to changes in my sleep system.<br><br>100. I feel that my health is much better than before.<br><br>101. Since my son was diagnosed with the disease:<br><br>1. I became significantly ill.<br><br>2. I never felt that my health is good.<br><br>3. I didn’t notice any changes in my health.					

Scoring system: the scale consisted of 101 items each item is rated on a 5- point Likert scale format, ranged from strongly agree (5) to strongly disagree (1) . The scores were summarized up and converted into percentage, then the score, were converted into qualitative variables through categorization based on a cut off point of 60%. **A-** scoring of parenting stress regarding either of the child's domains or parent's domains was considered high with scores ≥ 60% and low with scores < 60%. **B-**Total scoring of parenting stress regrading both domains together was considered high with scores ≥ 60% and low with scores < 60%.

Appendix 3: Rosenberg self-esteem scale

STATEMENT		Strongly Agree	Agree	Disagree	Strongly Disagree	
1.	I feel that I am a person of worth, at least on an equal plane with others.	○	○	○	○	
2.	I feel that I have a number of good qualities..	○	○	○	○	
3.	All in all, I am inclined to feel that I am a failure.	○	○	○	○	
4.	I am able to do things as well as most other people.	○	○	○	○	
5.	I feel I do not have much to be proud of.	○	○	○	○	
6.	I take a positive attitude toward myself.	○	○	○	○	
7.	On the whole, I am satisfied with myself.	○	○	○	○	
8.	I wish I could have more respect for myself.	○	○	○	○	

9.	I certainly feel useless at times.	○	○	○	○	
10.	At times I think I am no good at all.	○	○	○	○	

Score Results Reset

Your score on the Rosenberg self-esteem scale is: ____ .

Scores are calculated as follows:

- *For items 1, 2, 4, 6,* and *7:*
  Strongly agree = 3
  Agree = 2
  Disagree = 1
  Strongly disagree = 0

- *For items 3, 5, 8, 9,* and *10* (which are reversed in valence):
  Strongly agree = 0
  Agree = 1
  Disagree = 2
  Strongly disagree = 3

The scale ranges from 0-30. Scores between 15 and 25 are within normal range; scores below 15 suggest low self-esteem.

Bibliography for the *Parenting Stress Index*
Richard R. Abidin, Ed.D.

Long Form References 7

Short Form References 64

Anxiety 88

At Risk 91

Attachment 96

Attention Deficit Hyperactivity Disorder (ADHD) 102

Birth Defects 107

- Biochemical Genetic Disorder 108
- Congenital Diseases 109
- Congenital Heart Disease 110
- Craniofacial Anomalies 111
- Cystic Fibrosis 112
- Spina Bifida 114

Behavior Problems 115

Child Abuse

- Child Abuse/Neglect 118
- Child Abuse Risk Assessment 123

Chronic Health Disease 126

Communication Disorders

- Deaf or Hard of Hearing 129
- Speech Disorders 131

Language Development 132
Conduct Disorder/ODD 134
Cross-Cultural Studies 138
African 139
African American 140
Australian 144
Chinese 145
Dutch 147
Finnish 149
French 150
French Canadian 151
German 153
Hispanic 154
Icelandic 155
Irish 156
Israeli 157
Italian 158
Japanese 159
Korean 160
Malaysian 161
Mexican American 162
Native American 163
Norwegian 164
Portuguese 165
Swedish 166

Turkish 167
Vietnamese 168
Depression 169
Postpartum Depression 178
Developmental Disabilities 179
Autism 181
Asperger’s Syndrome 184
Down's Syndrome 185
Mental Retardation/ID 187
Screening for Disabilities 190
Family
Adoption 192
Discipline 194
Child Custody 195
Divorce 196
Employment 197
Families With A Handicapped Child 198
Family Support 201
Family Violence 202
Foster Care 204
Gay/Lesbian Families 205
Grandparents as primary caregivers 206
Incarceration 207
'Latch Key' Children 208
Military 209

Marital Adjustment/Prediction 210
Parent Disability 212
Parent Personality 213
Parental Mental Illness 214
Parenting Adult Children 215
Prediction of Child Adjustment 216
Single Parents 218
Teenage Parents 219
Fathers 222
Forensic 226
Health Care 227
Apneic 228
Asthma 229
Childhood Cancer 230
Children's Health Care 231
Chronic Pain 234
Diabetes 235
Feeding Issues 236
Epilepsy 237
Failure to Thrive 238
Fragile X Syndrome 239
Functional Somatic Symptoms 240
HIV/AIDS 241
Hospitalization 242
Infant Colic 243

Nocturnal Enuresis 244
Motor Impairment/Cerebral Palsy 245
Otitis Media 246
Parent's Health 247
Physical Disability 248
Premature Infants/Low Birth Weight 249
Reproduction/Assistive Reproduction 253
Sexual Development Disorders 255
Sleep Disorders 256
Tourette Syndrome 257
Traumatic Brain Injury (TBI) 258
Traumatic Injuries 259
Learning Disabled 260
Low Socioeconomic Status 261
Mothers 265
Mother's Health/Addiction 273
Parent-Child Interaction/ Observed Behavior 274
Overprotection 281
Program Evaluation 282
Behavioral Parent Training 289
Cognitive-Behavioral Therapy 292
Evidence-Based Treatment 294
Family Therapy/Filial Therapy 296
Parent Education 297
Parent Groups 300

Play Therapy/Attachment Therapy 301
Premature Termination/Adherence 302
Treatment Acceptability/Expectancies 304
Psychometrics/Clinical cut offs 305
Preschool/Head Start 306
Elementary School 319
Social Support 321
Substance Abuse 328

Long Form References

Abidin, R. R. (1983). Parenting stress and the utilization of pediatric services. Children's Health Care, 11(2), 70-3.

Abidin, R. R. (1992). The Determinants of Parenting Behavior. Journal of Clinical Child Psychology, 21(4), 407-412.

Abidin, R. R. (1997). Parenting Stress Index: A measure of the parent-child system. In Zalaquett, C.P. & Wood, R. (Eds), Evaluating stress: A book of resources (pp. 277-291). Scarecrow Press, Inc: Lanham, MD.

Abidin, R. R., & Brunner, J. F. (1995). Development of a Parenting Alliance Inventory. Journal of Clinical Child Psychology, 24(1), 31-40.

Abidin, R. R., & Wilfong, E. (1989). Parenting stress and its relationship to child health care. Children's Health Care, 18(2), 114-6.

Acton, R. G., & During, S. M. (1992). Preliminary results of aggression management training for aggressive. Journal of Interpersonal Violence, 7(3), 410-417.

Adam, E. K., & Gunnar, M. R. (2001). Relationship functioning and home and work demands predict individual differences in diurnal cortisol patterns in women. Psychoneuroendocrinology, 26(2), 189-208.

Adamakos, H., Kathleen, R., G., U. D., & John, P. (1986). Maternal social support as a predictor of mother-child stress and stimulation. Child Abuse & Neglect, 10(4), 463-470.

Adams, J. W., & Tidwell, R. (1989). An instructional guide for reducing the stress of hearing parents of hearing-impaired children. American Annals of the Deaf, 134(5), 323-328.

Ahmann, E., Wulff, L., & Meny, R. G. (1992). Home apnea monitoring and disruptions in family life: a multidimensional controlled study. American Journal of Public Health, 82(5), 719-22.

Allen, K. D., Maguire, K. B., Williams, G. E., & Sanger, W. G. (1996). The effects of infertility on parent-child relationships and adjustment. Children's Health Care, 25(2), 93-105.

Als, H., & Gilkerson, L. (1997). The Role of Relationship-Based Developmentally Supportive Newborn Intensive-Care in Strengthening Outcome of Preterm Infants. Semeinars in Perinatology, 21(3), 178-189.

Amankwaa, L., & Pickler, R. (2007). Measuring Maternal Responsiveness. ABNF Journal, 18(1), 4-15. doi: Article.

Ammerman, R. T., & Patz, R. J. (1996). Determinants of Child-Abuse Potential - Contribution of Parent and Child Factors. Journal of Clinical Child Psychology, 25(3), 300-307.

Anastopoulos, A. D., Guevremont, D. C., Shelton, T. L., & DuPaul, G. J. (1992). Parenting stress among families of children with attention deficit hyperactivity disorder. Journal of Abnormal Child Psychology, 20(5), 503-20.

Anastopoulos, A. D., Shelton, T. L., DuPaul, G. J., & Guevremont, D. C. (1993). Parent training for attention-deficit hyperactivity disorder: Its impact on parent functioning. Journal of Abnormal Child Psychology, 21(5).

Ando, J., Nonaka, K., Ozaki, K., Sato, N., Fujisawa, K. K., Suzuki, K., et al. (2006). The Tokyo Twin Cohort Project: overview and initial findings. Twin Research and Human Genetics: The Official Journal of the International Society for Twin Studies, 9(6), 817-826. doi: 10.1375/183242706779462480.

Andresen, P. A., & Telleen, S. L. (1992). The Relationship Between Social Support and Maternal Behaviors and Attitudes - A Meta-Analytic Review. American Journal of Community Psychology, 20(6), 753-774.

Andrews-Casal, M., Johnston, D., Fletcher, J., Mulliken, J. B., Stal, S., & Hecht, J. T. (1998). Cleft lip with or without cleft palate: effect of family history on reproductive planning, surgical timing, and parental stress. Cleft Palate Craniofacial Journal, 35(1), 52-7.

Antshel, K. M., & Joseph, G. (2006). Maternal Stress in Nonverbal Learning Disorder: A Comparison With Reading Disorder. Journal of Learning Disabilities, 39(3), 194. doi: Article.

Appelbaum, M., Batten, D. A., Belsky, J., Booth, C., Bradley, R., Brownell, C., Caldwell, B., Campbell, S., Clarkestewart, A., Cohn, J., Cox, M., Fendt, K., Friedman, S., Goldberg, W., Greenberger, E., Hirshpasek, K., Huston, A., Marshall, N., McCartney, K., Obrien, M., Owen, M. T., Phillips, D., Ricciuti, H., Spieker, S., Vandell, D. L., & Weinraub, M. (1997). Familial Factors Associated with the Characteristics of Nonmaternal Care for Infants. Journal of Marriage and the Family, 59(2), 389-408.

Archer, L. A., & Szatmari, P. (1990). Assessment and Treatment of Food Aversion in a 4-Year-Old Boy - A Multidimensional Approach. Canadian Journal of Psychiatry, 35(6), 501-505.

Archer, R. P., Buffington-Vollum, J. K., Stredny, R. V., & Handel, R. W. (2006). A survey of psychological test use patterns among forensic psychologists. Journal of Personality Assessment, 87(1), 84-94. doi: 10.1207/s15327752jpa8701_07.

Arditti, J. A., & Maddenderdich, D. (1997). Joint and Sole Custody Mothers - Implications for Research and Practice. Families in Society - The Journal of Contemporary Human Services, 78(1), 36-45.

Armstrong, K. L., Fraser, J. A., Dadds, M. R., & Morris, J. (2000). Promoting secure attachment, maternal mood and child health in a vulnerable population: a randomized controlled trial. Journal of Pediatrics & Child Health, 36(6), 555-62.

Athanasiou, M. S., & Gunning, M. P. (1999). Filial therapy: effects on two children's behavior and mothers' stress. Psychological Reports, 84(2), 587-90.

August, G. J., Lee, S. S., Bloomquist, M. L., Realmuto, G. M., & Hektner, J. M. (2004). Maintenance Effects of an Evidence-Based Prevention Innovation for Aggressive Children Living in Culturally Diverse Urban Neighborhoods: The Early Risers Effectiveness Study. Journal of Emotional & Behavioral Disorders, 12(4), 194-205. doi: Article.

August, G. J., Realmuto, G. M., Hektner, J. M., & Bloomquist, M. L. (2001). An integrated components preventive intervention for aggressive elementary school children: the early risers program. Journal of Consulting and Clinical Psychology, 69(4), 614-626.

Bagley, C., & Mallick, K. (1997). Temperament, CNS Problems and Maternal Stressors - Interactive Predictors of Conduct Disorder in 9-Yr-Olds. Perceptual and Motor Skills, 84(2), 617-618.

Bagner, D. M., & Eyberg, S. M. (2003). Father involvement in parent training: When does it matter?. Journal of Clinical Child & Adolescent Psychology, 32(4), 599-605.

Baker, B. L., & Heller, T. L. (1996). Preschool-Children with Externalizing Behaviors - Experience of Fathers and Mothers. Journal of Abnormal Child Psychology, 24(4), 513-532.

Baker, D. B. (1994). Parenting stress and ADHD: A comparison of mothers and fathers. Journal of Emotional & Behavioral Disorders, 2(1), 46-50.

Baker, D. B., & Kevin, M. (1995). Parenting stress in parents of children with attention-deficit hyperactivity disorder and parents of children with learning disabilities. Journal of Child & Family Studies, 4(1), 57-68.

Baker-Ericzén, M. J., Brookman-Frazee, L., & Stahmer, A. (2005). Stress levels and adaptability in parents of toddlers with and without autism spectrum disorders. Research and Practice for Persons with Severe Disabilities, 30(4), 194–204.

Barkley, R. A., & Fischer, M. (1988). Development of a multimethod clinical protocol for assessing stimulant drug response in children with attention deficit disorder. Journal of Clinical Child Psychology, 17, 14-24.

Barkley, R. A., Anastopoulos, A., Guevremont, D. C., & Fletcher, K. E. (1992). Adolescents with attention deficit hyperactivity disorder: Mother-adolescent interactions, family beliefs and conflicts, and maternal psychopathology. Journal of Abnormal Child Psychology, 20(3), 263-288.

Barkley, R. A., Fischer, M., Edelbrock, C., & Smallish, L. (1991). The adolescent outcome of hyperactive children diagnosed by research. Journal of Child Psychology & Psychiatry & Allied Disciplines, 32(2), 233-255.

Barkley, R. A., Guevremont, D. C., Anastopoulos, A. D., & Fletcher, K. E. (1992). A comparison of three family therapy programs for treating family conflicts. Journal of Consulting & Clinical Psychology, 60(3), 450-462.

Barkley, R. A., McMurray, M. B., Edelbrock, C. S., & Robbins, K. (1989). The response of aggressive and nonaggressive ADHD children to two doses of Methylphenidate. Journal of the American Academy of Child & Adolescent Psychiatry, 28(6), 873-881.

Barnett, D. W., Hall, J. D., & Bramlett, R. K. (1990). Family factors in preschool assessment and intervention: A validity study of parenting stress and coping measures. Journal of School Psychology, 28(1), 13-20.

Baroni, M. A., Anderson, Y. E., & Mischler, E. (1997). Cystic fibrosis newborn screening: impact of early screening results on parenting stress. Pediatric Nursing, 23(2), 143-51.

Barrera, M. E., & Kitching, K. J. (1991). A 3-year early home intervention follow-up study with low birthweight infants and their parents. Topics in Early Childhood Special Education, 10(4), 14-28.

Barrett, P. M., Farrell, L., Pina, A. A., Peris, T. S., & Piacentini, J. (2008). Evidence-Based Psychosocial Treatments for Child and Adolescent Obsessive-Compulsive Disorder. Journal of Clinical Child & Adolescent Psychology, 37(1), 131-155. doi: 10.1080/15374410701817956.

Bartholomew, L. K., Czyzewski, D. I., Parcel, G. S., Swank, P. R., Sockrider, M. M., Mariotto, M. J., Schidlow, D. V., Fink, R. J., & Seilheimer, D. K. (1997). Self-

management of cystic fibrosis: short-term outcomes of the cystic fibrosis family education program. Health Education & Behavior, 24(5), 652-66.

Bartley, J.-A. (1999). Exploratory study of a model for evaluating wrap-around services: Characteristics of children and youth exhibiting various degrees of success. Dissertation Abstracts International: Section B: the Sciences & Engineering, 60(1-B).

Bauman, L. J., Camacho, S., Westbrook, L., & Forbes-Jones, E. (1997). Correlates of personal stigma and social stigma among mothers with HIV/AIDS : National Conference of Women With HIV.

Beardmore, S., Tate, R., & Liddle, B. (1999). Does Information and Feedback Improve Children's Knowledge and Awareness of Deficits After Traumatic Brain Injury. Neuropsychological Rehabilitation, 9(1), 45-62.

Bech, P., Malt, U. F., Dencker, S. J., & Ahlfors, U. G. (1993). Scales for Assessment of Diagnosis and Severity of Mental-Disorders. Acta Psyciatrica Scandinavica, 87(S372).

Beck, S. J., Young, G. H., & Tarnowski, K. J. (1990). Maternal Characteristics and Perceptions of Pervasive and Situational Hyperactives and Normal Controls. Journal of the American Academy of Child and Adolescent Psychiatry, 29(4), 558-565.

Beckman, P. J. (1991). Comparison of mothers' and fathers' perceptions of the effect of young children with and without disabilities. American Journal on Mental Retardation, 95(5), 585-595.

Beebe, S. A., Casey, R., & Pinto-Martin, J. (1993). Association of reported infant crying and maternal parenting stress. Clinical Pediatrics, 32(1), 15-9.

Beg, M. R., Casey, J. E., & Saunders, C. D. (2007). A typology of behavior problems in preschool children. Assessment, 14(2), 111-128.

Behl, D. D., Akers, J. F., Boyce, G. C., & Taylor, M. J. (1996). Do Mothers Interact Differently with Children Who Are Visually-Impaired. Journal of Visual Impairment and Blindness, 90(6), 501-511.

Bellinger, D. C., Stiles, K. M., & Needleman, H. L. (1992). Low-Level Lead-Exposure, Intelligence and Academic-Achievement - A Long-Term Follow-Up-Study. Pediatrics, 90(6), 855-861.

Bellinger, D., Leviton, A., Allred, E., & Rabinowitz, M. (1994). Prenatal and Postnatal Lead-Exposure and Behavior Problems in School-Aged Children. Environmental Research, 66(1), 12-30.

Bendell, D., Goldberg, M. S., Urbano, M. T., & Urbano, R. C. (1987). Differential impact of parenting sick infants. Infant Mental Health Journal, 8(1), 28-36.

Bendell, D., Stone, W. L., Field, T. M., & Goldstein, S. (1989). Children's effects on parenting stress in a low income, minority population. Topics in Early Childhood Special Education, 8(4), 58-71.

Benzies, K. M., Harrison, M., & Magill-Evans, J. (2004). Parenting stress, marital quality, and child behaviour problems at age 7 years. Public Health Nursing, 21(2), 111-121.

Berry, J. O., & H., J. W. (1995). The Parental Stress Scale: Initial psychometric evidence. Journal of Social & Personal Relationships, 12(3), 463-472.

Bhavnagri, N. P. (1999). Low income African American mothers' parenting stress and instructional strategies to promote peer relationships in preschool children. Early Education & Development, 10(4), 551-571.

Bigras, M., & Lafreniere, P. J. (1994). Influence of Psychosocial Risk, Marital Conflicts and Parental Stress on the Quality of Mother-Son and Mother-Daughter Interactions. Canadian Journal of Behavioural Science, 26(2), 280-297.

Bigras, M., Lafreniere, P., & Dumas, J. (1996). Discriminant validity of the parent and child scales of the parenting stress index. Early Education & Development, 7(2), 167-178.

Bijttebier, P., Vertommen, H., & Florentie, K. (2003). Risk-Taking Behavior as a Mediator of the Relationship between Childrens Temperament and Injury Liability. Psychology and Health, 18, 645-653. doi: 10.1080/0887044031000094831.

Birkeland, R., Thompson, J. K., & Phares, V. (2005). Adolescent motherhood and postpartum depression. Journal of Clinical Child and Adolescent Psychology, 34(2), 292–300.

Bithoney, W. G., Van Sciver, M. M., Foster, S., Corso, S., & Tentindo, C. (1995). Parental stress and growth outcome in growth-deficient children. Pediatrics, 96(4 Pt 1), 707-11.

Black, M. M. (1995). Failure-to-Thrive - Strategies for Evaluation and Intervention. School Psychology Review, 24(2), 171-185.

Black, M. M., & Nitz, K. (1996). Grandmother co-residence, parenting, and child development among low income, urban teen mothers. Journal of Adolescent Health, 18(3), 218-26.

Black, M. M., Hutcheson, J. J., Dubowitz, H., Starr, R. H., & Berensonhoward, J. (1996). The Roots of Competence - Mother-Child Interaction Among Low-Income, Urban, African-American Families. Journal of Applied Developmental Psychology, 17(3), 367-391.

Black, M. M., Nair, P., & Harrington, D. (1994). Maternal HIV-Infection - Parenting and Early Child-Development. Journal of Pediatric Psychology, 19(5), 595-616.

Black, M. M., Nair, P., Kight, C., Wachtel, R., Roby, P., & Schuler, M. (1994). Parenting and early development among children of drug-abusing women: effects of home intervention. Pediatrics, 94(4 Pt 1), 440-8.

Black, M., Schuler, M., & Nair, P. (1993). Prenatal Drug Exposure - Neurodevelopmental Outcome and Parenting Environment. Journal of Pediatric Psychology, 18(5), 605-620.

Blakeney, P., Meyer, W., 3rd, Robert, R., Desai, M., Wolf, S., & Herndon, D. (1998). Long-term psychosocial adaptation of children who survive burns involving 80% or greater total body surface area. Journal of Trauma, 44(4), 625-32.

Blakeney, P., Meyer, W., Moore, P., Murphy, L., Broemeling, L., Robson, M., & Herndon, D. (1993). Psychosocial sequelae of pediatric burns involving 80% or greater total body surface area. Journal of Burn Care & Rehabilitation, 14(6), 684-9.

Blakeney, P., Moore, P., Broemeling, L., Hunt, R., Herndon, D. N., & Robson, M. (1993). Parental stress as a cause and effect of pediatric burn injury. Journal of Burn Care & Rehabilitation, 14(1), 73-9.

Bloom, A. A., Wright, J. A., Morris, R. D., Campbell, R. M., & Krawiecki, N. S. (1997). Additive Impact of In-hospital Cardiac Arrest on the Functioning of Children With Heart Disease. Pediatrics, 99(3), 390-398.

Bohr, Y. (2005). Infant mental health programs: Experimenting with innovative models-One center's experience with new program funding. Infant Mental Health Journal, 26(5), 407. doi: 10.1002/imhj.20062.

Bonner, M. J., & Finney, J. W. (1996). A Psychosocial Model of Childrens Health-Status. Advances in Clinical Child Psychology, 18, 231-282.

Bos, H. M. W., van Balen, F., & van den Boom, D. C. (2007). Child adjustment and parenting in planned lesbian-parent families. The American Journal of Orthopsychiatry, 77(1), 38-48. doi: 10.1037/0002-9432.77.1.38.

Bos, H. M., van Balen, F., & van den Boom, D. C. (2004). Experience of parenthood, couple relationship, social support, and child-rearing goals in planned lesbian mother families. Journal of Child Psychology and Psychiatry, 45(4), 755–764.

Bow, J. N., & Quinnell, F. A. (2002). A critical review of child custody evaluation reports. Family Court Review, 40(2), 164-176. doi: 10.1111/j.174-1617.2002.tb00827.x.

Bradley, R. H., & A., B. J. (1990). Assessment of the home environment. In J. H. Johnson (Ed.), Developmental assessment in clinical child psychology: A handbook (Vol. 163, pp. 219-250). New York: Pergamon Press, Inc.

Bramlett, R. K., Hall, J. D., Barnett, D. W., & Rowell, K. (1995). Child developmental/educational status in kindergarten and family coping as predictors of parenting stress: Issues for parent consultation. Journal of Psychoeducational Assessment, 13(2), 157-166.

Bramlett, R. K., Rowell, R. K., & Mandenberg, K. (2000). Predicting first grade achievement from kindergarten screening measures: A comparison of child and family predictors. Research in the Schools, 7(1), 1-9.

Breen, M. J., & Barkley, R. A. (1988). Child psychopathology and parenting stress in girls and boys having attention deficit disorder with hyperactivity. Journal of Pediatric Psychology, 13(2), 265-280.

Briggs-Gowan, M. J., & Carter, A. S. (1998). Preliminary Acceptability and Psychometrics of the Infant-Toddler Social and Emotional Assessment (ITSEA) - A New Adult-Report Questionnaire. Infant Mental Health Journal, 19(4), 422-445.

Brotman, L. M., Klein, R. G., Kamboukos, D., Brown, E. J., Coard, S. I., & Sosinsky, L. S. (2003). Preventive intervention for urban, low-income preschoolers at familial risk for conduct problems: A randomized pilot study. Journal of Clinical Child and Adolescent Psychology, 32(2), 246–257.

Brown, J. V., Bakeman, R., Coles, C. D., Platzman, K. A., & Lynch, M. E. (2004). Prenatal cocaine exposure: A comparison of 2-year-old children in parental and non-parental care. Child Development, 75(4), 1282–1295.

Browne, J. V., & Talmi, A. (2005). Family-based intervention to enhance infant-parent relationships in the neonatal intensive care unit. Journal of Pediatric Psychology, 30(8), 667-677.

Brunette, M. F., Richardson, F., White, L., Bemis, G., & Eelkema, R. E. (2004). Integrated family treatment for parents with severe psychiatric disabilities. Psychiatric Rehabilitation Journal, 28(2), 177-180. doi: Article.

Bryan, T., Burstein, L., Chao, P., & Ergul, C. (2006). The relationship between health status, language development, and behavior in young children. Physical Disabilities: Education and Related Services, 24(2), 7-19.

Budd, K. S., & Holdsworth, M. J. (1996). Issues in Clinical-Assessment of Minimal Parenting Competence. Journal of Clinical Child Psychology, 25(1), 2-14.

Buist, A. (1998). Childhood Abuse, Parenting and Postpartum Depression. Australian and New Zealand Journal of Psychiatry, 32(4), 479-487.

Buist, A., & Janson, H. (1995). Effect of Exposure to Dothiepin and Northiaden in Breast-Milk on Child-Development. British Journal of Psychiatry, 167(SEP), 370-373.

Burchinal, M., Roberts, J. E., Zeisel, S. A., Hennon, E. A., & Hooper, S. (2006). Social risk and protective child, parenting, and child care factors in early elementary school years. Parenting: Science and Practice, 6(1), 79. doi: 10.1207/s15327922par0601_4

Burke, W., Abidin, R. R., & Younger, J. B. (1991). Parenting Stress Index: A model of parenting stress. Research in Nursing & Health, 14, 197-204.

Burrell, B., Thompson, B., & Sexton, D. (1994). Predicting Child-Abuse Potential Across Family Types. Child Abuse & Neglect, 18(12), 1039-1049.

Burrell, B., Thompson, B., & Sexton, D. (1995). Measurement Characteristics of the Perceived Adequacy of Resources Scale. Educational & Psychological Measurement, 55(2), 249-257.

Butcher, P. R., Wind, T., & Bouma, A. (2008). Parenting stress in mothers and fathers of a child with a hemiparesis: sources of stress, intervening factors and long-term expressions of stress. Child: Care, Health & Development, 34(4), 530-541. doi: 10.1111/j.1365-2214.2008.00842.x.

Butter, E. M., Mulick, J. A., & Metz, B. (2006). Eight case reports of learning recovery in children with pervasive developmental disorders after early intervention. Behavioral Interventions, 21(4), 227-243. doi: 10.1002/bin.225.

Butteweg Gratton, M. (1999). Case studies in the therapeutic introduction of a transitional object to recast the relational experience of abused children. Dissertation Abstracts International, 59(8-A).

Byrne, J. M., Dewolfe, N. A., & Bawden, H. N. (1998). Assessment of Attention-Deficit Hyperactivity Disorder in Preschoolers. Child Neuropsychology, 4(1), 49-66.

Bywater, T., Hutchings, J., Daley, D., Whitaker, C., Yeo, S. T., Jones, K., et al. (2009). Long-term effectiveness of a parenting intervention for children at risk of developing

conduct disorder. The British Journal of Psychiatry: The Journal of Mental Science, 195(4), 318-324. doi: 10.1192/bjp.bp.108.056531

Calam, R., Bolton, C., & Roberts, J. (2002). Maternal expressed emotion, attributions and depression and entry into therapy for children with behaviour problems. British Journal of Clinical Psychology, 41(2), 213216.

Calzada, E. J., Eyberg, S. M., Rich, B., & Querido, J. G. (2004). Parenting disruptive preschoolers: Experiences of mothers and fathers. Journal of Abnormal Child Psychology, 32(2), 203–213.

Cameron, S. J., & Orr, R. (1989). Stress in families of school-aged children with delayed mental development. Canadian Journal of Rehabilitation, 2(3), 137-144.

Cameron, S., Dobson, L., & Day, D. (1991). Stress in parents of developmentally delayed and non-delayed preschool children. Canada's Mental Health, 39(1), 13-17.

Camfield, C., Breau, L., & Camfield, P. (2001). Impact of pediatric epilepsy on the family: a new scale for clinical and research use. Epilepsia, 42(1), 104-12.

Campbell, S. B. (1994). Hard-to-Manage Preschool Boys - Externalizing Behavior, Social Competence, and Family Context at 2-Year Follow-Up. Journal of Abnormal Child Psychology, 22(2), 147-166.

Campbell, S. B. (1997). Behavior Problems in Preschool-Children - Developmental and Family Issues. Advances in Clinical Child Psychology, 19, 1-26.

Campbell, S. B., Pierce, E. W., Moore, G., Marakovitz, S., & Newby, K. (1996). Boys Externalizing Problems at Elementary-School Age - Pathways from Early Behavior Problems, Maternal Control, and Family Stress. Development and Psychopathology, 8(4), 701-719.

Campis, L. B., DeMaso, D. R., & Twente, A. W. (1995). The role of maternal factors in the adaptation of children with craniofacial disfigurement. Cleft Palate Craniofacial Journal, 32(1), 55-61.

Capage, L. C., Bennett, G., & McNeil, C. B. (2001). A comparison between African American and Caucasian children referred for treatment of disruptive behavior disorders. Child & Family Behavior Therapy, 23(1), 1-14.

Cappelli, M., Mcgrath, P. J., Daniels, T., Manion, I., & Schillinger, J. (1994). Marital Quality of Parents of Children with Spina-Bifida - A Case-Comparison Study. Journal of Developmental and Behavioral Pediatrics, 15(5), 320-326.

Carothers, S. S., Borkowski, J. G., & Whitman, T. L. (2006). Children of Adolescent Mothers: Exposure to Negative Life Events and the Role of Social Supports on Their Socioemotional Adjustment. Journal of Youth and Adolescence, 35(5), 822-832. doi: 10.1007/s10964-006-9096-8.

Carson, D. K., & Schauer, R. W. (1992). Mothers of children with asthma: Perceptions of parenting stress and the mother-child relationship. Psychological Reports, 71(3, Pt 2), 1139-1148.

Casady, A., Diener, M., Isabella, R., & Wright, C. (2001). Attachment security among families in poverty: maternal, child, and contextual characteristics. Paper presented at the 2001 Biennial Conference of the Society for Research in Child Development, Minneapolis, MN.

Caulfield, M. B., Fischel, J. E., DeBaryshe, B. D., & Whitehurst, G. J. (1989). Behavioral correlates of developmental expressive language disorder. Journal of Abnormal Child Psychology, 17(2), 187-201.

Chaffee, C. A., Cunningham, C. E., & Secord, G. (1991). The influence of parenting stress and child behavior problems on parental. Journal of Abnormal Child Psychology, 19(1), 65-74.

Chan, R. W., Raboy, B., & Patterson, C. J. (1998). Psychosocial Adjustment Among Children Conceived via Donor Insemination by Lesbian and Heterosexual Mothers. Child Development, 69(2), 443-457.

Chan, Y. C. (1994). Parenting stress and social support of mothers who physically abuse their children in Hong Kong. Child Abuse & Neglect, 18(3), 261-9.

Chang, S. S. Y., Ng, C. F. N., & Wong, S. N. (2002). Behavioural problems in children and parenting stress associated with primary nocturnal enuresis in Hong Kong. Acta Paediatrica, 91(4), 475-479.

Chisholm, K. (1998). A 3 Year Follow-Up of Attachment and Indiscriminate Friendliness in Children Adopted from Romanian Orphanages. Child Development, 69(4), 1092-1106.

Chisholm, K., Carter, M. C., Ames, E. W., & Morison, S. J. (1995). Attachment security and indiscriminately friendly behavior in children adopted from Romanian orphanages. Development & Psychopathology, 7(2), 283-294.

Clark, R., Hyde, J. S., Essex, M. J., & Klein, M. H. (1997). Length of Maternity Leave and Quality of Mother-Infant Interactions. Child Development, 68(2), 364-383.

Cohn, E. S., & Cermak, S. A. (1998). Including the Family Perspective in Sensory Integration Outcomes Research. American Journal of Occupational Therapy, 52(7), 540-546.

Coleman, P. K., & Karraker, K. H. (1998). Self-Efficacy and Parenting Quality - Findings and Future Applications. Developmental Review, 18(1), 47-85.

Colpin, H., & Soenen, S. (2002). Parenting and psychosocial development of IVF children: a follow-up study. Human Reproduction, 17(4), 1116-1123.

Colpin, H., DeMunter, A., Nys, K., & Vandemuelebroecke, L. (2000). Pre- and postnatal determinants of parenting stress in mothers of one-year-old twins. Marriage & Family Review, 30(1-2), 99-107.

Colpin, H., Munter, A. D., Nys, K., & Vandemeulebroecke, L. (1999). Parenting stress and psychosocial well-being among parents with twins conceived naturally or by reproductive technology. Human Reproduction, 14(12), 3133-7.

Conners, N. A., Edwards, M. C., & S. Grant, A. (2007). An Evaluation of a Parenting Class Curriculum for Parents of Young Children: Parenting the Strong-Willed Child. Journal of Child & Family Studies, 16(3), 321-330. doi: 10.1007/s10826-006-9088-z.

Cook, R., Bradley, S., & Golombok, S. (1998). A Preliminary-Study of Parental Stress and Child-Behavior in Families with Twins Conceived by in-Vitro Fertilization. Human Reproduction, 13(11), 3244-3246.

Cook, R., Golombok, S., Bish, A., & Murray, C. (1995). Disclosure of Donor Insemination - Parental Attitudes. American Journal of Orthopsychiatry, 65(4), 549-559.

Cook, R., Vatev, I., Michova, Z., & Golombok, S. (1997). The European Study of Assisted Reproduction Families - A Comparison of Family Functioning and Child-Development Between Eastern and Western-Europe. Journal of Psychosomatic Obstetrics and Gynecology, 18(3), 203-212.

Corbett, B. A, Schupp, C. W., Levine, S., & Mendoza, S. (2009). Comparing cortisol, stress, and sensory sensitivity in children with autism. Autism Research, 2(1),39-49.

Corbett, B., Mendoza, S., Baym, C., Bunge, S., & Levine, S. (2008). Examining cortisol rhythmicity and responsivity to stress in children with Tourette syndrome. Psychoneuroendocrinology, 33(6), 810-820. doi: 10.1016/j.psyneuen.2008.03.014.

Cornish, A. M., McMahon, C. A., Ungerer, J. A., Barnett, B., Kowalenko, N., & Tennant, C. (2006). Maternal depression and the experience of parenting in the second postnatal year. Journal of Reproductive & Infant Psychology, 24(2), 121-132. doi: 10.1080/02646830600644021.

Costas, M. B. (1999). Filial therapy with non-offending parents of children who have been sexually abused. Dissertation Abstracts International, 59(7-A).

Costin, J., & Chambers, S. M. (2007). Parent management training as a treatment for children with oppositional defiant disorder referred to a mental health clinic. Clinical Child Psychology and Psychiatry, 12(4), 511-524.

Cowan, P. A., Cowan, C. P., Pruett, M. K., Pruett, K., & Wong, J. J. (2009). Promoting Fathers' Engagement With Children: Preventive Interventions for Low-Income Families. Journal of Marriage and Family, 71(3), 663-679. doi: 10.1111/j.1741-3737.2009.00625.x.

Cowen, P. S., & Reed, D. A. (2002). Effects of respite care for children with developmental disabilities: evaluation of an intervention for at risk families. Public Health Nursing, 19(4), 272-283.

Crandell, L. E., Fitzgerald, H. E., & Whipple, E. E. (1997). Dyadic Synchrony in Parent-Child Interactions - A Link with Maternal Representations of Attachment Relationships. Infant Mental Health Journal, 18(3), 247-264.

Crist, W., Mcdonnell, P., Beck, M., Gillespie, C. T., Barrett, P., & Mathews, J. (1994). Behavior at Mealtimes and the Young-Child with Cystic-Fibrosis. Journal of Developmental and Behavioral Pediatrics, 15(3), 157-161.

Cuccaro, M. L., Holmes, G. R., & Wright, H. H. (1993). Behavior problems in preschool children: A pilot study. Psychological Reports, 72(1), 121-122.

Cuskelly, M., Chant, D., & Hayes, A. (1998). Behaviour problems in the siblings of children with Down syndrome: Associations with family responsibilities and parental stress. International Journal of Disability, Development & Education, 45(3), 295-311.

Czyzewski, D. I., Mariotto, M. J., Bartholomew, L. K., Lecompte, S. H., & Sockrider, M. M. (1994). Measurement of Quality of Well-Being in a Child and Adolescent Cystic-Fibrosis Population. Medical Care, 32(9), 965-972.

Dalla, R. L., & Gamble, W. C. (1997). Exploring Factors Related to Parenting Competence Among Navajo Teenage Mothers - Dual Techniques of Inquiry. Family Relations, 46(2), 113-121.

Damashek, A. L., Williams, N. A., Sher, K. J., Peterson, L., Lewis, T., & Schweinle, W. (2005). Risk for minor childhood injury: an investigation of maternal and child factors. Journal of Pediatric Psychology, 30(6), 469-480. doi: 10.1093/jpepsy/jsi072.

Danforth, J. S. (1998). The Outcome of Parent Training Using the Behavior Management Flow-Chart with Mothers and Their Children with Oppositional Defiant

Disorder and Attention-Deficit Hyperactivity Disorder. Behavior Modification, 22(4), 443-473.

Danseco, E. R., & Holden, E. W. (1998). Are There Different Types of Homeless Families - A Typology of Homeless Families Based on Cluster-Analysis. Family Relations, 47(2), 159-165.

Darke, P. R., & Goldberg, S. (1994). Father-Infant interaction and parent stress with healthy and medically compromised infants. Infant Behavior & Development, 17(1), 3-14.

Davis, H., & Spurr, P. (1998). Parent Counseling - An Evaluation of a Community Child Mental-Health-Service. Journal of Child Psychology and Psychiatry and Allied Disciplines, 39(3), 365-376.

Davis, N. O., & Carter, A. S. (2008). Parenting Stress in Mothers and Fathers of Toddlers with Autism Spectrum Disorders: Associations with Child Characteristics.. Journal of Autism & Developmental Disorders, 38(7), 1278-1291. doi: 10.1007/s10803-007-0512-z.

Day, C., Davis, H., & Hind, R. (1998). The Development of a Community Child and Family Mental-Health-Service. Child Care Health and Development, 24(6), 487-500.

De Bruyne, E., Van Hoecke, E., Van Gompel, K., Verbeken, S., Baeyens, D., et al. (2009). Problem Behavior, Parental Stress and Enuresis. The Journal of Urology, 182(4), 2015-2021. doi: 10.1016/j.juro.2009.05.102

De Los Reyes, A., & Kazdin, A. E. (2006). Informant Discrepancies in Assessing Child Dysfunction Relate to Dysfunction Within Mother-Child Interactions. Journal of Child & Family Studies, 15(5), 643-661. doi: 10.1007/s10826-006-9031-3.

Deater-Deckard, K. (1998). Parenting Stress and Child Adjustment - Some Old Hypotheses and New Questions. Clinical Psychology - Science and Practice, 5(3), 314-332.

Deater-Deckard, K., & Scarr, S. (1996). Parenting Stress Among Dual-Earner Mothers and Fathers - Are There Gender Differences. Journal of Family Psychology, 10(1), 45-59.

Deater-Deckard, K., Scarr, S., McCartney, K., & Eisenberg, M. (1994). Paternal Separation Anxiety - Relationships with Parenting Stress, Child-Rearing Attitudes, and Maternal Anxieties. Psychological Science, 5(6), 341-346.

DeGangi, G. A., Sickel, R. Z., Kaplan, E. P., & Wiener, A. S. (1997). Mother-infant interactions in infants with disorders of self-regulation. Physical & Occupational Therapy in Pediatrics, 17(1), 17-44.

DeGangi, G. A., Sickel, R. Z., Wiener, A. S., & Kaplan, E. P. (1996). Fussy babies: to treat or not to treat? British Journal of Occupational Therapy, 59(10), 457-64.

Degangi, G. A., Wietlisbach, S., Goodin, M., & Scheiner, N. (1993). A Comparison of Structured Sensorimotor Therapy and Child-Centered Activity in the Treatment of Preschool-Children with Sensorimotor Problems. American Journal of Occupational Therapy, 47(9), 777-786.

Dekovic, M., & Meeus, W. (1997). Peer Relations in Adolescence - Effects of Parenting and Adolescents Self-Concept. Journal of Adolescence, 20(2), 163-176.

Dekovic, M., & Rabotegsaric, Z. (1997). Parental Child-Rearing Practices and Adolescent Peer Relations. Drustvenaistraizivanja, 6(4-5), 427-445.

Deković, M., Janssens, J. M., & van As, N. M. (2003). Family Predictors of Antisocial Behavior in Adolescence. Family Process, 42(2), 223. doi: Article.

Dellve, L., Samuelsson, L., Tallborn, A., Fasth, A., & Hallberg, L. R. (2006). Stress and well-being among parents of children with rare diseases: a prospective intervention study. Journal of Advanced Nursing, 53(4), 392-402. doi: 10.1111/j.1365-2648.2006.03736.x.

DeMaso, D. R., Campis, L. K., Wypij, D., Bertram, S., Lipshitz, M., & Freed, M. (1991). The impact of maternal perceptions and medical severity on the adjustment of children with congenital heart disease. Journal of Pediatric Psychology, 16(2), 137-49.

Demick, J., & Andreoletti, C. (1995). Some Relations Between Clinical and Environmental Psychology. Environment and Behavior, 27(1), 56-72.

Dempsey, I., & Keen, D. (2008). A Review of Processes and Outcomes in Family-Centered Services for Children With a Disability. Topics in Early Childhood Special Education, 28(1), 42-52. doi: 10.1177/0271121408316699.

DiLauro, M. D. (2004). Psychosocial Factors Associated with Types of Child Maltreatment. Child Welfare, 83(1), 69. doi: Article.

Dixon, L., Hamilton-Giachritsis, C., Browne, K., & Ostapuik, E. (2007). The Co-occurrence of Child and Intimate Partner Maltreatment in the Family: Characteristics of the Violent Perpetrators. Journal of Family Violence, 22(8), 675-689. doi: 10.1007/s10896-007-9115-x.

Domoto, P., Weinstein, P., Leroux, B., Koday, M., Ogura, S., & Iatridiroberson, I. (1994). White Spots Caries in Mexican-American Toddlers and Parental Preference for Various Strategies. Journal of Dentistry for Children, 61(5-6), 342-346.

Donenberg, G., & Baker, B. L. (1993). The Impact of Young-Children with Externalizing Behaviors on Their Families. Journal of Abnormal Child Psychology, 21(2), 179-198.

Donohue-Moore, M. (1994). Commentary on Caregiver stress in grandparents raising grandchildren. Nursing Scan in Oncology, 3(4), 10.

Douglas, J. E., & Bryon, M. (1996). Interview Data on Severe Behavioral Eating Difficulties in Young-Children. Archives of Disease in Childhood, 75(4), 304-308.

Douglas, J. E., Hulson, B., & Trompeter, R. S. (1998). Psychosocial Outcome of Parents and Young-Children After Renal-Transplantation. Child Care Health and Development, 24(1), 73-83.

Doussard-Roosevelt, J. A., Porges, S. W., Scanlon, J. W., Alemi, B., & Scanlon, K. B. (1997). Vagal regulation of heart rate in the prediction of developmental outcome for very low birth weight preterm infants. Child Development, 68(2), 173-86.

Doussard-Roosevelt, J., Porges, S. W., & McClenny, B. D. (1996). Behavioral Sleep States in Very-Low-Birth-Weight Preterm Neonates - Relation to Neonatal Health and Vagal Maturation. Journal of Pediatric Psychology, 21(6), 785-802.

Dow, K. H., Harris, J. R., & Roy, C. (1994). Pregnancy after breast-conserving surgery and radiation therapy for breast cancer. Journal of the National Cancer Institute, 16, 131-7.

Drummond, J., Kysela, G. M., McDonald, L., Alexander, J., & Fleming, D. (1996). Risk and resiliency in two samples of Canadian families. Health & Canadian Society, 4(1), 117-51.

Duis, S. S., Summers, M., & Summers, C. R. (1997). Parent Versus Child Stress in Diverse Family Types - An Ecological Approach. Topics in Early Childhood Special Education, 17(1), 53-73.

Dukewich, T. L., Borkowski, J. G., & Whitman, T. L. (1996). Adolescent Mothers and Child-Abuse Potential - An Evaluation of Risk-Factors. Child Abuse & Neglect, 20(11), 1031-1047.

Dumas, J. E., & Wekerle, C. (1995). Maternal Reports of Child-Behavior Problems and Personal Distress as Predictors of Dysfunctional Parenting. Development and Psychopathology, 7(3), 465-479.

Dumas, J. E., Martinez, A., & Lafreniere, P. J. (1998). The Spanish Version of the Social Competence and Behavior Evaluation (SCBE) Preschool Edition - Translation and Field Testing. Hispanic Journal of Behavioral Sciences, 20(2), 255-269.

Dumas, J. E., Wolf, L. C., Fisman, S. N., & Culligan, A. (1991). Parenting stress, child behavior problems, and dysphoria in parents of children with autism, Down syndrome, behavior disorders, and normal development. Exceptionality, 2(2), 97-110.

Dumka, L. E., Gonzales, N. A., Wood, J. L., & Formoso, D. (1998). Using Qualitative Methods to Develop Contextually Relevant Measures and Preventive Interventions - An Illustration. American Journal of Community Psychology, 26(4), 605-637.

Dundas, S., & Kaufman, M. (2000). The Toronto Lesbian Family Study. Journal of Homosexuality, 40(2), 65-79.

Dunham, P. J., Hurshman, A., Litwin, E., Gusella, J., Ellsworth, C., & Dodd, P. W. D. (1998). Computer-Mediated Social Support - Single Young Mothers as a Model System. American Journal of Community Psychology, 26(2), 281-306.

Dunne, L., Sneddon, H., Iwaniec, D., & Stewart, M. C. (2007). Maternal mental health and faltering growth in infants. Child Abuse Review, 16(5), 283-295.

Eddy, M. E., Carter, B. D., Kronenberger, W. G., Conradsen, S., Eid, N. S., Bourland, S. L., & Adams, G. (1998). Parent relationships and compliance in cystic fibrosis. Journal of Pediatric Health Care, 12(4), 196-202.

Edens, J. F., Cavell, T. A., & Hughes, J. N. (1999). The Self-Systems of Aggressive-Children - A Cluster-Analytic Investigation. Journal of Child Psychology and Psychiatry and Allied Disciplines, 40(3), 441-453.

Eisengart, S. P., Singer, L. T., Fulton, S., & Baley, J. E. (2003). Coping and Psychological Distress in Mothers of Very Low Birth Weight Young Children. Parenting: Science and Practice, 3(1), 49. doi: 10.1207/S15327922PAR0301_03.

Eisengart, S. P., Singer, L. T., Kirchner, H. L., Min, M. O., Fulton, S., Short, E. J., et al. (2006). Factor structure of coping: two studies of mothers with high levels of life stress. Psychological Assessment, 18(3), 278-288. doi: 10.1037/1040-3590.18.3.278.

Eisenstadt, T. H., Eyberg, S., Mcneil, C. B., Newcomb, K., & Funderburk, B. (1993). Parent-Child Interaction Therapy with Behavior Problem Children - Relative Effectiveness of 2 Stages and Overall Treatment Outcome. Journal of Clinical Child Psychology, 22(1), 42-51.

Eiserman, W. D., McCoun, M., & Escobar, C. M. (1990). A cost-effectiveness analysis to two alternative program models for serving speech-disordered preschoolers. Journal of Early Intervention, 14, 297-317.

Eiserman, W. D., Weber, C., & McCoun, M. (1992). 2 Alternative Program Models for Serving Speech-Disordered Preschoolers - A 2nd Year Follow-Up. Journal of Communication Disorders, 25(2-3), 77-106.

Eiserman, W. D., Weber, C., & McCoun, M. (1995). Parent and Professional Roles in Early Intervention - A Longitudinal Comparison of the Effects of 2 Intervention Configurations. Journal of Special Education, 29(1), 20-44.

El-Kamary, S. S., Higman, S. M., Fuddy, L., McFarlane, E., Sia, C., & Duggan, A. K. (2004). Hawaii's healthy start home visiting program: determinants and impact of rapid repeat birth. Pediatrics, 114(3), e317-326. doi: 10.1542/peds.2004-0618.

Emery, J., Paquette, D., & Bigras, M. (2008). Factors predicting attachment patterns in infants of adolescent mothers. Journal of Family Studies, 14(1), 65-90. doi: Article.

Endriga, M. C. (1998). Feeding and Attachment in Infants with and Without Orofacial Clefts. Infant Behavior and Development, 21(4), 699-712.

Esdaile, S. A. (1996). A play-focused intervention involving mothers of preschoolers. American Journal of Occupational Therapy, 50(2), 113-23.

Esdaile, S. A., & Greenwood, K. M. (1995). A survey of mothers' relationships with their preschoolers. Occupational Therapy International, 2(3), 204-19.

Esdaile, S. A., & Greenwood, K. M. (2003). A comparison of mother's and father's experience of parenting stress and attributions for parent-child interaction outcomes. Occupational Therapy International, 10(2), 115. doi: Article.

Esdaile, S. E., & Greenwood, K. M. (1995). Issues of parenting stress: A study involving mothers of toddlers. Journal of Family Studies, 1(2), 153-165.

Ethier, L. S. (1992). Developmental factors related to stress in neglectful and abusive mothers. Apprentissage et Socialisation, 15(3), 222-236.

Ethier, L. S., & Lafreniere, P. J. (1993). Single-Parent Maternal Stress with Respect to Preschooler Aggression. International Journal of Psychology, 28(3), 273-289.

Ethier, L. S., & Lafreniere, P. J. (1993). The relationship between maternal stress and preschool children's aggressiveness in single-parent families. International Journal of Psychology, 28(3), 273-289.

Ethier, L. S., Lacharite, C., & Couture, G. (1995). Childhood adversity, parental stress, and depression of negligent mothers. Child Abuse & Neglect, 19(5), 619-32.

Eyberg, S. M. B. S. R. A. J. (1995). Parent-child interaction therapy: A psychosocial model for the treatment of young children with conduct problem behavior and their families. Psychopharmacology Bulletin, 31(1), 83-91.

Eyberg, S. M., Boggs, S. R., & Rodriguez, C. M. (1992). Relationships between maternal parenting stress and child disruptive behavior. Child & Family Behavior Therapy, 14(4), 1-9.

Fagan, J., Bernd, E., & Whiteman, V. (2007). Adolescent Fathers' Parenting Stress, Social Support, and Involvement with Infants. Journal of Research on Adolescence (Blackwell Publishing Limited), 17(1), 1-22. doi: 10.1111/j.1532-7795.2007.00510.x.

Fagnano, M., van Wijngaarden, E., Connolly, H. V., Carno, M. A., Forbes-Jones, E., & Halterman, J. S. (2009). Sleep-Disordered Breathing and Behaviors of Inner-City Children With Asthma. Pediatrics, 124(1), 218-225. doi: 10.1542/peds.2008-2525.

Farel, A. M., & Hooper, S. R. (1998). Relationship between the Maternal Social Support Index and the Parenting Stress Index in mothers of very-low-birthweight children now age 7. Psychological Reports, 83(1), 173-4.

Farel, A. M., Hooper, S. R., Teplin, S. W., Henry, M. M., & Kraybill, E. N. (1998). Very-low-birthweight infants at seven years: an assessment of the health and neurodevelopmental risk conveyed by chronic lung disease. Journal of Learning Disabilities, 31(2), 118-26.

Farmer, J. E., Clippard, D. S., Luehrwiemann, Y., Wright, E., & Owings, S. (1996). Assessing Children with Traumatic Brain Injury During Rehabilitation - Promoting School and Community Reentry. Journal of Learning Disabilities, 29(5), 532-548.

Feinfield, K. A., & Baker, B. L. (2004). Empirical support for a treatment program for families of young children with externalizing problems. Journal of Clinical Child and Adolescent Psychology: The Official Journal for the Society of Clinical Child and Adolescent Psychology, American Psychological Association, Division 53, 33(1), 182-195. doi: 10.1207/S15374424JCCP3301_17

Feldman, M. A., & Waltonallen, N. (1997). Effects of Maternal Mental-Retardation and Poverty on Intellectual, Academic, and Behavioral Status of School-Age-Children. American Journal on Mental Retardation, 101(4), 352-364.

Feldman, M. A., Leger, M., & Walton-Allen, N. (1997). Stress in mothers with intellectual disabilities. Journal of Child & Family Studies, 6(4), 471-485.

Feldman, M. A., Varghese, J., Ramsay, J., & Rajska, D. (2002). Relationships between social support, stress and mother-child interactions in mothers with intellectual disabilities. Journal of Applied Research in Intellectual Disabilities, 15(4), 314-323.

Fey, M. E., Warren, S. F., Brady, N., Finestack, L. H., Bredin-Oja, S. L., Fairchild, M., et al. (2006). Early Effects of Responsivity Education/Prelinguistic Milieu Teaching for Children With Developmental Delays and Their Parents. Journal of Speech, Language & Hearing Research, 49(3), 526-547. doi: 10.1044/1092-4388(2006/039).

Finnstevenson, M., Desimone, L., & Chung, A. M. (1998). Linking Child-Care and Support Services with the School - Pilot Evaluation of the School of the 21st-Century. Children and Youth Services Review, 20(3), 177-205.

Fischel, J. E., Whitehurst, G. J., Caulfield, M. B., & DeBaryshe, B. (1989). Language growth in children with expressive language delay. Pediatrics, 83(2), 218-27.

Fisman, S., Wolf, L., Ellison, D., Gillis, B., Freeman, T., & Szatmari, P. (1996). Risk and Protective Factors Affecting the Adjustment of Siblings of Children with Chronic Disabilities. Journal of the American Academy of Child and Adolescent Psychiatry, 35(11), 1532-1541.

Fite, P. J., Greening, L., & Stoppelbein, L. (2008). Relation between parenting stress and psychopathic traits among children. Behavioral Sciences & the Law, 26(2), 239-248.

Fitzgerald, M., Butler, B., & Kinsella, A. (1990). The burden on a family having a child with special needs. Irish Journal of Psychological Medicine, 7(2), 109-113.

Florsheim, P., Moore, D., Zollinger, L., MacDonald, J., & Sumida, E. (1999). The transition to parenthood among adolescent fathers and their partners: Does antisocial behavior predict problems in parenting? Applied Developmental Science, 3(3), 178-191.

Flory, V. (2004). A Novel Clinical Intervention for Severe Childhood Depression and Anxiety. Clinical Child Psychology and Psychiatry, 9(1), 9-23. doi: 10.1177/1359104504039167.

Forbes, L. M., Evans, E. M., Moran, G., & Pederson, D. R. (2007). Change in Atypical Maternal Behavior Predicts Change in Attachment Disorganization From 12 to 24 Months in a High-Risk Sample. Child Development, 78(3), 955-971. doi: 10.1111/j.1467-8624.2007.01043.x.

Forgays, D. K. (1992). Type A behavior and parenting stress in mothers with young children. Current Psychology: Research & Reviews, 11(1), 3-19.

Forgays, D. K., Hasazi, J. E., & Wasserman, R. C. (1992). Recurrent otitis media and parenting stress in mothers of two-year-old. Journal of Developmental & Behavioral Pediatrics, 13(5), 321-325.

Francis-Williams, N.(2005). Evaluation of a faith-based socioemotional support program for parents of African American youth with antisocial behaviors. (Unpublished doctoral dissertation). Nova Southeastern University, Florida.

Frankel, K. K., & Harmon, R. J. (1996). Depressed Mothers - They Don't Always Look as Bad as They Feel. Journal of the American Academy of Child and Adolescent Psychiatry, 35(3), 289-298.

Freeman, N. L. P. A. (1991). Child behaviours as stressors: Replicating and extending the use of the CARS. Journal of Child Psychology & Psychiatry & Allied Disciplines, 32(6), 1025-1030.

Friars, P., & Mellor, D. (2007). Drop Out from Behavioral Management Training Programs for ADHD: A Prospective Study. Journal of Child & Family Studies, 16(3), 427-441. doi: 10.1007/s10826-006-9096-z.

Fuller, G. B., & Rankin, R. E. (1994). Differences in levels of parental stress among mothers of learning disabled, emotionally impaired, and regular school children. Perceptual & Motor Skills, 78(2), 583-92.

Fuscaldo, D., Kaye, J. W., & Philliber, S. (1998). Evaluation of a Program for Parenting. Families in Society: The Journal of Contemporary Human Services, 79(1), 53-61.

Garbarino, J., & Kostelny, K. (1996). The Effects of Political Violence on Palestinian Childrens Behavior Problems - A Risk Accumulation Model. Child Development, 67(1), 33-45.

Garrison, M. E. B., Blalock, L. B., Zarski, J. J., & Merritt, P. B. (1997). Delayed Parenthood - An Exploratory-Study of Family Functioning. Family Relations, 46(3), 281-290.

Gartstein, M. A., & Marmion, J. (2008). Fear and positive affectivity in infancy: Convergence/discrepancy between parent-report and laboratory-based indicators. Infant Behavior & Development, 31(2), 227-238.

Gartstein, M. A., & Sheeber, L. (2004). Child Behavior Problems and Maternal Symptoms of Depression: A Mediational Model. Journal of Child & Adolescent Psychiatric Nursing, 17(4), 141-150. doi: Article.

Gartstein, M. A., Putnam, S. P., Becken-Jones, L., & Rothbart, M. K. (2002). Infant Behavior Questionnaire-Revised: New evidence in support of reliability and validity. In

Proceedings. Presented at the biannual convention of the International Society for Infant Study, Toronto, Canada.

Gelfand, D. M., Teti, D. M., Seiner, S. A., & Jameson, P. B. (1996). Helping Mothers Fight Depression - Evaluation of a Home-Based Intervention Program for Depressed Mothers and Their Infants. Journal of Clinical Child Psychology, 25(4), 406-422.

Gelman, V. S., & King, N. J. (2001). Wellbeing of Mothers with Children Exhibiting Sleep Disturbance. Australian Journal of Psychology, 53(1), 18-22.

Gershater-Molko, R. M., Lutzker, J. R., & Wesch, D. (2002). Using recidivism data to evaluate Project SafeCare: teaching bonding, safety, and health care skills to parents. Child Maltreatment, 7(3), 277-285.

Gershoff, E. T., Raver, C. C., Aber, J. L., & Lennon, M. C. (2007). Income Is Not Enough: Incorporating Material Hardship Into Models of Income Associations With Parenting and Child Development. Child Development, 78(1), 70-95. doi: 10.1111/j.1467-8624.2007.00986.x.

Geva, R., Yosipof, R., Eschel, R., Leitner, Y., Valevski, A. F., & Harel, S. (2009). Readiness and Adjustments to School for Children With Intrauterine Growth Restriction (IUGR): An Extreme Test Case Paradigm. Exceptional Children, 75(2), 211-230.

Girolametto, L. (1995). Reflection on the Origins of Directiveness - Implications for Intervention. Journal of Early Intervention, 19(2), 104-106.

Girolametto, L., & Tannock, R. (1994). Correlates of Directiveness in the Interactions of Fathers and Mothers of Children with Developmental Delays. Journal of Speech and Hearing Research, 37(5), 1178-1191.

Glass, P., Wagner, A. E., Papero, P. H., Rajasingham, S. R., Civitello, L. A., Kjaer, M. S., Coffman, C. E., Getson, P. R., & Short, B. L. (1995). Neurodevelopmental Status at Age 5 Years of Neonates Treated with Extracorporeal Membrane-Oxygenation. Journal of Pediatrics, 127(3), 447-457.

Glavin, K., Smith, L., Sørum, R., & Ellefsen, B. (2010). Redesigned community postpartum care to prevent and treat postpartum depression in women - a one-year follow-up study. Journal of Clinical Nursing, 19, 3051-3062.

Glenn, S. S., Cunningham, C. C., Poole, H. H., Reeves, D. D., & Weindling, M. M. (2009). Maternal parenting stress and its correlates in families with a young child with cerebral palsy. Child: Care, Health and Development, 35(1), 71-78.

Goldberg, S., Janus, M., Washington, J., Simmons, R. J., Maclusky, I., & Fowler, R. S. (1997). Prediction of Preschool Behavioral-Problems in Healthy and Pediatric Samples. Journal of Developmental and Behavioral Pediatrics, 18(5), 304-313.

Goldberg, S., Morris, P., Simmons, R. J., Fowler, R. S., & Levison, H. (1990). Chronic illness in infancy and parenting stress: a comparison of three groups of parents. Journal of Pediatric Psychology, 15(3), 347-58.

Goldberg, S., Simmons, R. J., Newman, J., Campbell, K., & Fowler, R. S. (1991). Congenital Heart-Disease, Parental Stress, and Infant-Mother Relationships. Journal of Pediatrics, 119(4), 661-666.

Goldman, J., Sorensen, E., & Ward, M. (1995). Brief Child Assessment Battery to Assist with Treatment Planning and Program-Evaluation. Community Mental Health Journal, 31(5), 437-448.

Golombok, S., Brewaeys, A., Cook, R., Giavazzi, M. T., Guerra, D., Mantovani, A., Vanhall, E., Crosignani, P. G., & Dexeus, S. (1996). The European Study of Assisted Reproduction Families - Family Functioning and Child-Development. Human Reproduction, 11(10), 2324-2331.

Golombok, S., Cook, R., Bish, A., & Murray, C. (1993). Quality of Parenting in Families Created by the New Reproductive Technologies - A Brief Report of Preliminary Findings. Journal of Psychosomatic Obstetrics and Gynecology, 14(S), 17-22.

Golombok, S., Cook, R., Bish, A., & Murray, C. (1995). Families Created by the New Reproductive Technologies - Quality of Parenting and Social and Emotional Development of the Children. Child Development, 66(2), 285-298.

Golombok, S., Murray, C., Brinsden, P., & Abdalla, H. (1999). Social versus biological parenting: Family functioning and the socioemotional development of children conceived by egg or sperm donation. Journal of Child Psychology & Psychiatry & Allied Disciplines, 40(4), 519-527.

Gonchar, N. (1995). College-student mothers and on-site child care: luxury or necessity? Social Work in Education, 17(4), 226-34.

Gorzka, P. A. (1999). Homeless parents: parenting education to prevent abusive behaviors. Journal of Child & Adolescent Psychiatric Nursing, 12(3), 101-9.

Gorzka, P. A. (1999). Homeless parents’ perceptions of parenting stress. Journal of Child & Adolescent Psychiatric Nursing, 12(1), 7-16.

Gottlieb, L. N., & Feeley, N. (1996). The McGill Model of Nursing and children with a chronic condition: "who benefits, and why?". Canadian Journal of Nursing Research, 28(3), 29-48.

Grant, D. (1996). Generalizability of Findings of Exploratory Practice-Based Research on Polydrug-Addicted Mothers. Research on Social Work Practice, 6(3), 292-307.

Greaves, D. (1997). The effect of rational-emotive parent education on the stress of mothers of young children with Down syndrome. Journal of Rational-Emotive & Cognitive Behavior Therapy, 15(4), 249-267.

Greaves, D., & Poole, C. (1996). Mothers' observations of children with Downs Syndrome coping with demands to adapt. Journal of Intellectual & Developmental Disability, 21(2), 153-162.

Greene, R. W., Abidin, R. R., & Kmetz, C. (1997). The Index of Teaching Stress - A Measure of Student-Teacher Compatibility. Journal of School Psychology, 35(3), 239-259.

Greene, R. W., Ablon, J. S., Goring, J. C., Raezer-Blakely, L., Markey, J., Monuteaux, M. C., et al. (2004). Effectiveness of collaborative problem solving in affectively dysregulated children with oppositional-defiant disorder: initial findings. Journal of Consulting and Clinical Psychology, 72(6), 1157-1164. doi: 10.1037/0022-006X.72.6.1157

Greenley, R. N., Holmbeck, G. N., & Rose, B. M. (2006). Predictors of Parenting Behavior Trajectories Among Families of Young Adolescents with and without Spina Bifida. J. Pediatr. Psychol., 31(10), 1057-1071. doi: 10.1093/jpepsy/jsj011.

Gross, D., Fogg, L., & Tucker, S. (1995). The Efficacy of Parent Training for Promoting Positive Parent Toddler Relationships. Research in Nursing & Health, 18(6), 489-499.

Grossman, J., & Shigaki, I. S. (1994). Investigation of Familial and School-Based Risk-Factors for Hispanic Head-Start Children. American Journal of Orthopsychiatry, 64(3), 456-467.

Guralnick, M. J., Hammond, M. A., Connor, R. T., & Neville, B. (2006). Stability, change, and correlates of the peer relationships of young children with mild developmental delays. Child development, 77(2), 312–324.

Guralnick, M. J., Hammond, M. A., Neville, B., & Connor, R. T. (2008). The relationship between sources and functions of social support and dimensions of child- and parent-related stress. Journal of Intellectual Disability Research: JIDR, 52(12), 1138-1154. doi: 10.1111/j.1365-2788.2008.01073.x.

Guralnick, M. J., Neville, B., Hammond, M. A., & Connor, R. T. (2007). Linkages Between Delayed Children's Social Interactions With Mothers and Peers. Child Development, 78(2), 459-473. doi: 10.1111/j.1467-8624.2007.01009.x.

Guralnick, M., Neville, B., Hammond, M., & Connor, R. (2007). The friendships of young children with developmental delays: A longitudinal analysis. Journal of Applied Developmental Psychology, 28(1), 64-79. doi: 10.1016/j.appdev.2006.10.004.

Ha, E. H., Oh, K. J., & Kim, E. J. (1999). Depressive symptoms and family relationship of married women: Focused on parenting stress and marital dissatisfaction. Korean Journal of Clinical Psychology, 18(1), 79-93.

Hadadian, A., & Merbler, J. (1996). Mother’s stress: Implications for attachment relationships. Early Child Development & Care, 125, 59-66.

Hall, J. D., & Barnett, D. W. (1991). Classification of risk status in preschool screening: A comparison of alternative measures. Journal of Psychoeducational Assessment, 9(2), 152-159.

Hall, S., Bobrow, M., & Marteau, T. M. (1997). Parents attributions of blame for the birth of a child with Down syndrome: a pilot study. Psychology & Health, 12(4), 579-87.

Hanada, H., Honda, S., Tokumaru, T., & Hiroki, O. (2006). Association Between Mothers' Concern About Child Rearing and Their Parenting Stress. Acta Med Nagasaki Ensia, 51(4), 115-120.

Hanson, M. J., & Hanline, M. F. (1990). Parenting a child with a disability: A longitudinal study of parental stress and adaptation. Journal of Early Intervention, 14(3), 234-248.

Harmer, A. L. M., Sanderson, J., & Mertin, P. (1999). Influence of Negative Childhood Experiences on Psychological Functioning, Social Support, and Parenting for Mothers Recovering from Addiction. Child Abuse & Neglect, 23(5), 421-433.

Harrington, D., Black, M. M., Starr, R. H., & Dubowitz, H. (1998). Child Neglect - Relation to Child Temperament and Family Context. American Journal of Orthopsychiatry, 68(1), 108-116.

Harris, H. E., Ellison, G. T. H., & Clement, S. (1999). Relative Importance of Heritable Characteristics and Life-Style in the Development of Maternal Obesity. Journal of Epidemiology and Community Health, 53(2), 66-74.

Harrison, M. J., & Magill-Evans, J. (1996). Mother and father interactions over the first year with term and preterm infants. Research in Nursing & Health, 19(6), 451-9.

Harvey, E. (1998). Parental Employment and Conduct Problems Among Children with Attention-Deficit/Hyperactivity Disorder - An Examination of Child-Care Workload and Parenting Well-Being as Mediating Variables. Journal of Social and Clinical Psychology, 17(4), 476-490.

Hatcher, J. W., Powers, L. L., & Richtsmeier, A. J. (1993). Parental Anxiety and Response to Symptoms of Minor Illness in Infants. Journal of Pediatric Psychology, 18(3), 397-408.

Hauenstein, E. J., Marvin, R. S., Snyder, A. L., & Clarke, W. L. (1989). Stress in parents of children with diabetes mellitus. Diabetes Care, 12(1), 18-23.

Hausfather, A., Toharia, A., Laroche, C., & Engelsmann, F. (1997). Effects of Age of Entry, Day-Care Quality, and Family Characteristics on Preschool Behavior. Journal of Child Psychology and Psychiatry and Allied Disciplines, 38(4), 441-448.

Heinze, M. C., & Grisso, T. (1996). Review of instruments assessing parenting competencies used in child custody evaluations. Behavioral Sciences & the Law, 14(3), 293-313.

Heller, T. L., Baker, B. L., Henker, B., & Hinshaw, S. P. (1996). Externalizing Behavior and Cognitive-Functioning from Preschool to First-Grade - Stability and Predictors. Journal of Clinical Child Psychology, 25(4), 376-387.

Henderson, L. W., Aydlett, L. A., & Bailey, D. B. (1993). Evaluating Family Needs Surveys - Do Standard Measures of Reliability and Validity Tell Us What We Want to Know. Journal of Psychoeducational Assessment, 11(3), 208-219.

Hindley, P. (1997). Psychiatric Aspects of Hearing Impairments. Journal of Child Psychology and Psychiatry and Allied Disciplines, 38(1), 101-117.

Hintermair, M. (2000). Children who are hearing impaired with additional disabilities and related aspects of parental stress. Exceptional Children, 66(3), 327-332.

Hintermair, M. (2000). Hearing impairment, social networks, and coping: The need for families with hearing-impaired children to relate to other parents and to hearing-impaired adults. American Annals of the Deaf, 145(1), 41-53.

Hodapp, R. M., Ricci, L. A., Ly, T. A., & Fidler, D. J. (2003). The effects of the child with Down syndrome on maternal stress. British Journal of Developmental Psychology, 21(1), 137-151.

Hoffman, C. D., Sweeney, D. P., Hodge, D., Lopez-Wagner, M. C., & Looney, L. (2009). Parenting Stress and Closeness: Mothers of Typically Developing Children and Mothers

of Children With Autism. Focus on Autism and Other Developmental Disabilities, 24(3), 178-187. doi: 10.1177/1088357609338715

Hoffmann, R. G., III, Rodrigue, J. R., Andres, J. M., & Novak, D. A. (1995). Moderating effects of family functioning on the social adjustment of children with liver disease. Children's Health Care, 24(2), 107-17.

Holaday, B., Turner-Henson, A., Kanematsu, Y., Krulik, T., & Wang, R. (1997). Stress in mothers of chronically ill children: a cross cultural study. Australian Paediatric Nurse, 6(1), 2-9.

Holden, E. W., & Banez, G. A. (1996). Child abuse potential and parenting stress within maltreating families. Journal of Family Violence, 11(1), 1-12.

Holigrocki, R. J., & Hudson-Crain, R.(2004).Victim-victimizer relational dynamics as maintained by representational, defensive, and neurobiological functioning. Bulletin of the Menninger Clinic, 68(3), 197-212. doi: Article.

Holmbeck, G. N., Goreyferguson, L., Hudson, T., Seefeldt, T., Shapera, W., Turner, T., & Uhler, J. (1997). Maternal, Paternal, and Marital Functioning in Families of Preadolescents with Spina-Bifida. Journal of Pediatric Psychology, 22(2), 167-181.

Hooper, S. R., Burchinal, M. R., Roberts, J. E., Zeisel, S., & Neebe, E. C. (1998). Social and Family Risk-Factors for Infant Development at One-Year - An Application of the Cumulative Risk Model. Journal of Applied Developmental Psychology, 19(1), 85-96.

Hoppes, K., & Harris, S. L. (1990). Perceptions of child attachment and maternal gratification in mothers of children with Autism and Down's Syndrome. Journal of Clinical Child Psychology, 19, 365-370.

Horsch, U., Weber, C., Bertram, B., & Detrois, P. (1997). Stress experienced by parents of children with cochlear implants compared with parents of deaf children and hearing children. American Journal of Otology, 18(6 Suppl), 161-163.

Hsu, E., Davies, C. A., Hans, L., Sedlar, G., Nash, C. L., Holm, J. W., et al. (2001). Parallel group treatments for sexually abused children and their nonoffending caregivers: Child and family outcome and satisfaction. Presented at the 35th Annual Convention of the Association for the Advancement of Behavioral Therapy, Philadelphia, PA.

Huang, W., Rubin, S. E., & Zhang, F. (1998). Correlates of stress level in Chinese mothers of a child with mental retardation. International Journal of Rehabilitation Research, 21(2), 237-40.

Hughes, J. N., Cavell, T. A., & Grossman, P. B. (1997). A Positive View of Self - Risk or Protection for Aggressive-Children. Development and Psychopathology, 9(1), 75-94.

Hutcheson, J. J., & Black, M. M. (1996). Psychometric properties of the Parenting Stress Index in a sample of low-income African-American mothers of infants and toddlers. Early Education & Development, 7(4), 381-400.

Hutcheson, J. J., Black, M. M., & Starr, R. H. (1993). Developmental Differences in Interactional Characteristics of Mothers and Their Children with Failure-to-Thrive. Journal of Pediatric Psychology, 18(4), 453-466.

Hyde, J. S., Else-Quest, N. M., Goldsmith, H. H., & Biesanz, J. C. (2004). Children's Temperament and Behavior Problems Predict Their Employed Mothers' Work Functioning. Child Development, 75(2), 580-594. doi: 10.1111/j.1467-8624.2004.00694.x.

Ievers-Landis, C. E., Storfer-Isser, A., Rosen, C., Johnson, N. L., & Redline, S. (2008). Relationship of Sleep Parameters, Child Psychological Functioning, and Parenting Stress to Obesity Status Among Preadolescent Children. Journal of Developmental & Behavioral Pediatrics, 29(4), 243-252. doi: 10.1097/DBP.0b013e31816d923d.

Innocenti, M. S., Huh, K., & Boyce, G. C. (1992). Families of children with disabilities: Normative data and other considerations on parenting stress. Topics in Early Childhood Special Education, 12(3), 403-427.

Jackson, A. P. (1998). The Role of Social Support in Parenting for Low-Income, Single, Black Mothers. Social Service Review, 72(3), 365-378.

Jackson, A. P. (1999). The Effects of Nonresident Father Involvement on Single Black Mothers and Their Young-Children. Social Work, 44(2), 156-166.

Jackson, A. P., Bentler, P. M., & Franke, T. M. (2008). Low-Wage Maternal Employment and Parenting Style. Social Work, 53(3), 267. doi: Article.

Jackson, A. P., Gyamfi, P., Brooksgunn, J., & Blake, M. (1998). Employment Status, Psychological Well-Being, Social Support, and Physical Discipline Practices of Single Black Mothers. Journal of Marriage and the Family, 60(4), 894-902.

Jackson, K., Ternestedt, B., Magnuson, A., & Schollin, J. (2007). Parental stress and toddler behaviour at age 18 months after pre-term birth. Acta Paediatrica, 96(2), 227-232. doi: 10.1111/j.1651-2227.2007.00015.x.

Jacobsen, T., & Miller, L. J. (1998). Mentally Ill Mothers Who Have Killed - 3 Cases Addressing the Issue of Future Parenting Capability. Psychiatric Services, 49(5), 650-657.

Jacobsen, T., Miller, L. J., & Kirkwood, K. P. (1997). Assessing Parenting Competence in Individuals with Severe Mental-Illness - A Comprehensive Service. Journal of Mental Health Administration, 24(2), 189-199.

Jang, M. (2000). Effectiveness of filial therapy for Korean parents. International Journal of Play Therapy, 9(2), 39-56

Janus, M., & Goldberg, S. (1997). Factors Influencing Family Participation in a Longitudinal-Study - Comparison of Pediatric and Healthy Samples. Journal of Pediatric Psychology, 22(2), 245-262.

Jarvis, P. A., & Creasey, G. L. (1991). Parental stress, coping, and attachment in families with an 18-month-old infant. Infant Behavior & Development, 14(4), 383-395.

Johnson, J. H., & Goldman, J. (1993). Approaches to Developmental Assessment. Advances in Clinical Child Psychology, 15, 243-274.

Johnston, C., Hessl, D., Blasey, C., Eliez, S., Erba, H., Dyer-Friedman, J.,…Reiss, A. L. (2003). Factors associated with parenting stress in mothers of children with fragile X syndrome. Journal of Developmental and Behavioral Pediatrics, 24(4), 267-275.

Joyner, K., Silver, C., & Stavinoha, P. (2009). Relationship Between Parenting Stress and Ratings of Executive Functioning in Children With ADHD, Journal of Psychoeducational Assessment, 27, 452-464.

Kasari, C., & Sigman, M. (1997). Linking Parental Perceptions to Interactions in Young-Children with Autism. Journal of Autism and Developmental Disorders, 27(1), 39-57.

Kazak, A. E., & Barakat, L. P. (1997). Brief Report - Parenting Stress and Quality-of-Life During Treatment for Childhood Leukemia Predicts Child and Parent Adjustment After Treatment Ends. Journal of Pediatric Psychology, 22(5), 749-758.

Kazak, A. E., & Marvin, R. S. (1984). Differences, difficulties and adaptation: Stress and social networks in families with a handicapped child. Family Relations: Journal of Applied Family & Child Studies, 33(1), 67-77.

Kazak, A. E., Penati, B., Boyer, B. A., Himelstein, B., Brophy, P., Waibel, M. K., Blackall, G. F., Daller, R., & Johnson, K. (1996). A Randomized Controlled Prospective Outcome Study of a Psychological and Pharmacological Intervention Protocol for Procedural Distress in Pediatric Leukemia. Journal of Pediatric Psychology, 21(5), 615-631.

Kazak, A. E., Penati, B., Waibel, M. K., & Blackall, G. F. (1996). The Perception of Procedures Questionnaire: psychometric properties of a brief parent report measure of procedural distress. Journal of Pediatric Psychology, 21(2), 195-207.

Kazak, A. E., Reber, M., & Snitzer, L. (1988). Childhood Chronic Disease and Family Functioning - A Study of Phenylketonuria. Pediatrics, 81(2), 224-230.

Kazdin, A. E. (1994). Family adversity, socioeconomic disadvantage, and parental stress: Contextual variables related to premature termination from child behavior therapy. Psicologia Conductual, 2(1), 5-21.

Kazdin, A. E. (1995). Child, Parent and Family Dysfunction as Predictors of Outcome in Cognitive-Behavioral Treatment of Antisocial Children. Behaviour Research and Therapy, 33(3), 271-281.

Kazdin, A. E., & Crowley, M. J. (1997). Moderators of Treatment Outcome in Cognitively Based Treatment of Antisocial Children. Cognitive Therapy and Research, 21(2), 185-207.

Kazdin, A. E., & Wassell, G. (1998). Treatment Completion and Therapeutic Change Among Children Referred for Outpatient Therapy. Professional Psychology-Research and Practice, 29(4), 332-340.

Kazdin, A. E., Holland, L., & Crowley, M. (1997). Family Experience of Barriers to Treatment and Premature Termination from Child Therapy. Journal of Consulting and Clinical Psychology, 65(3), 453-463.

Kazdin, A. E., Holland, L., Crowley, M., & Breton, S. (1997). Barriers to Treatment Participation Scale - Evaluation and Validation in the Context of Child Outpatient Treatment. Journal of Child Psychology and Psychiatry and Allied Disciplines, 38(8), 1051-1062.

Kazdin, A. E., Mazurick, J. L., & Bass, D. (1993). Risk for Attrition in Treatment of Antisocial Children and Families. Journal of Clinical Child Psychology, 22(1), 2-16.

Kazdin, A. E., Mazurick, J. L., & Siegel, T. C. (1994). Treatment Outcome Among Children with Externalizing Disorder Who Terminate Prematurely Versus Those Who Complete Psychotherapy. Journal of the American Academy of Child and Adolescent Psychiatry, 33(4), 549-557.

Kazdin, A. E., Siegel, T. C., & Bass, D. (1992). Cognitive problem-solving skills training and parent management training in the treatment of antisocial behavior in children. Journal of Consulting and Clinical Psychology, 60, 733-747.

Kazui, M., Muto, T., & Sonoda, N. (1996). The roles of marital quality and parenting stress in mother-preschooler relationships. (Japanese). Japanese Journal of Developmental Psychology, 7(1), 31-40.

Keefe, M. R., Froese-Fretz, A., & Kotzer, A. M. (1997). The REST regimen: an individualized nursing intervention for infant irritability. American Journal of Maternal Child Nursing, 22(1), 16-20.

Kelley, M. L., Herzog-Simmer, P. A., & Harris, M. A. (1994). Effects of military-induced separation on the parenting stress and family functioning of deploying mothers. Military Psychology, 6(2), 125-138.

Kelley, S. J. (1992). Parenting stress and child maltreatment in drug-exposed children. Child Abuse & Neglect, 16(3), 317-28.

Kelley, S. J. (1993). Caregiver stress in grandparents raising grandchildren. Image the Journal of Nursing Scholarship, 25(4), 331-7.

Kelly, L. E. (1995). Adolescent mothers: what factors relate to level of preventive health care sought for their infants? Journal of Pediatric Nursing: Nursing Care of Children & Families, 10(2), 105-13.

Kemp, L., Harris, E., McMahon, C., Matthey, S., Vimpani, G., Anderson, T., et al. (2008). Miller Early Childhood Sustained Home-visiting (MECSH) trial: design, method and sample description. BMC Public Health, 8, 1-12. doi: 10.1186/1471-2458-8-424.

Kenworthy, L., & Charnas, L. (1995). Evidence for a Discrete Behavioral-Phenotype in the Oculocerebrorenal Syndrome of Lowe. American Journal of Medical Genetics, 59(3), 283-290.

Kern, J. K., West, E. Y., Grannemann, B. D., Greer, T., Snell, L. M., Cline, L. L., …Trivedi, M. H. (2004). Reduction in stress and depressive symptoms in mothers of substance-exposed infants, participating in a psychosocial program. Maternal & Child Health Journal, 8(3), 127-136.

Kersh, J., Hedvat, T., Hauser-Cram, P., & Warfield, M. E. (2006). The contribution of marital quality to the well-being of parents of children with developmental disabilities. Journal of Intellectual Disability Research, 50(12), 883-893. doi: 10.1111/j.1365-2788.2006.00906.x.

Kim, H. (. K., Viner-Brown, S. I., & Garcia, J. (2007). Children's Mental Health and Family Functioning in Rhode Island. Pediatrics, 119(Supplement_1), S22-28. doi: 10.1542/peds.2006-2089E.

Kishchuk, N., Laurendeau, M. C., Desjardins, N., & Perreault, R. (1995). Parental Support - Effects of a Mass-Media Intervention. Canadian Journal of Public Health, 86(2), 128-132.

Klee, L., Kronstadt, D., & Zlotnick, C. (1997). Foster care's youngest: A preliminary report. American Journal of Orthopsychiatry, 67(2), 290-299.

Kluger, M. P., & Aprea, D. M. (1999). Grandparents raising grandchildren: A description of the families and a special pilot program. Journal of Gerontological Social Work, 32(1), 5-17.

Knight, D. K., & Wallace, G. (2003). Where are the children? An examination of children's living arrangements when mothers enter residential drug treatment. Journal of Drug Issues, 33(2), 305. doi: Article.

Knoester, M., Helmerhorst, F. M., van der Westerlaken, L. A., Walther, F. J., & Veen, S. (2007). Matched follow-up study of 5 8-year old ICSI singletons: child behaviour, parenting stress and child (health-related) quality of life. Human Reproduction, 22(12), 3098-3107.

Kobe, F. H., & Hammer, D. (1994). Parenting stress and depression in children with mental retardation and developmental disabilities. Research in Developmental Disabilities, 15(3), 209-21.

Koeske, G. F., & Koeske, R. D. (1990). The Buffering Effect of Social Support on Parental Stress. American Journal of Orthopsychiatry, 60(3), 440-451.

Koester, L. S., & Meadow-Orlans, K. P. (1999). Responses to interactive stress: infants who are deaf or hearing. American Annals of the Deaf (Silver Spring, MD), 144(5), 395-403.

Krauss, M. W. (1993). Child-Related and Parenting Stress - Similarities and Differences Between Mothers and Fathers of Children with Disabilities. American Journal on Mental Retardation, 97(4), 393-404.

Krauss, M. W., Upshur, C. C., Shonkoff, J. P., & Hausercram, P. (1993). The Impact of Parent Groups on Mothers of Infants with Disabilities. Journal of Early Intervention, 17(1), 8-20.

Krueckeberg, S. M., & Kappsimon, K. A. (1993). Effect of Parental Factors on Social Skills of Preschool-Children with Craniofacial Anomalies. Cleft Palate-Craniofacial Journal, 30(5), 490-496.

Kushalnagar, P., Krull, K., Hannay, J., Mehta, P., Caudle, S., & Oghalai, J. (2007). Intelligence, parental depression, and behavior adaptability in deaf children being considered for cochlear implantation. Journal of Deaf Studies and Deaf Education, 12(3), 335-349.

Lacharite, C., Ethier, L. S., & Couture, G. (1999). Sensitivity and specificity of the Parenting Stress Index in situations of child maltreatment. Canadian Journal of Behavioural Science, 31(4), 217-220.

Lacharite, C., Ethier, L., & Piche, C. (1992). Parental stress in mothers of preschool children: validation and Quebec norms for the Parental Stress Inventory. Sante Mentale au Quebec, 17(2), 183-203.

LaFiosca, T., & Loyd, B. H. (1986). Defensiveness and the assessment of parental stress and anxiety. Journal of Clinical Child Psychology, 15(3), 254-259.

Lafortune, K. A., & Carpenter, B. N. (1998). Custody Evaluations - A Survey of Mental-Health Professionals. Behavioral Sciences & the Law, 16(2), 207-224.

Lafreniere, P. J., & Capuano, F. (1997). Preventive Intervention as Means of Clarifying Direction of Effects in Socialization - Anxious-Withdrawn Preschoolers Case. Development and Psychopathology, 9(3), 551-564.

Landreth, G. L., & Lobaugh, A. F. (1998). Filial Therapy with Incarcerated Fathers - Effects on Parental Acceptance of Child, Parental Stress, and Child Adjustment. Journal of Counseling and Development, 76(2), 157-165.

LaValle, P. S., Glaros, A., Bohaty, B., & McCunniff, M. (2000). The effect of parental stress on the oral health of children. Journal of Clinical Psychology in Medical Settings, 7(4), 197-201.

Lederberg, A. R., & Golbach, T. (2002). Parenting stress and social support in hearing mothers of deaf and hearing children: a longitudinal study. Journal of Deaf Studies and Deaf Education, 7(4), 330-345.

Leigh, B., & Milgrom, J. (2008). Risk factors for antenatal depression, postnatal depression and parenting stress. BMC psychiatry, 8(1), 24.

Lessenberry, B. M., & Rehfeldt, R. A. (2004). Evaluating Stress Levels of Parents of Children with Disabilities. Exceptional Children, 70(2), 231–245.

Levendosky, A. A., & Grahambermann, S. A. (1998). The Moderating Effects of Parenting Stress on Childrens Adjustment in Woman-Abusing Families. Journal of Interpersonal Violence, 13(3), 383-397.

Levendosky, A. A., & Graham-Bermann, S. A. (2001). Parenting in battered women: the effects of domestic violence on women and their children. Journal of Family Violence, 16(2), 171-192.

Leventhal-Belfer, L., Cowan, P. A., & Cowan, C. P. (1992). Satisfaction with Child-Care Arrangements - Effects on Adaptation to Parenthood. American Journal of Orthopsychiatry, 62(2), 165-177.

Lévesque, S., Clément, M., & Chamberland, C. (2007). Factors Associated with Co-occurrence of Spousal and Parental Violence: Quebec Population Study. Journal of Family Violence, 22(8), 661-674. doi: 10.1007/s10896-007-9106-y.

Levin, R., & Banks, S. (1991). Stress in parents of children with epilepsy. Canadian Journal of Rehabilitation, 4(4), 229-38.

Ligezinska, M., Firestone, P., Manion, I. G., Mcintyre, J., Ensom, R., & Wells, G. (1996). Childrens Emotional and Behavioral Reactions Following the Disclosure of Extrafamilial Sexual Abuse - Initial Effects. Child Abuse & Neglect, 20(2), 111-125.

Lisul-Mihić, I., & Kapor-Stanulović, N. (2002). Cultural influence on aims of the inclusion of mothers in pre-school children's play. Psihologija, 35(1-2), 49-64. doi: 10.2298/PSI0201049L.

Little, M., Murphy, J. M., Jellinek, M. S., Bishop, S. J., & Arnett, H. L. (1994). Screening 4-Year-Old and 5-Year-Old Children for Psychosocial Dysfunction - A Preliminary-Study with the Pediatric Symptom Checklist. Journal of Developmental and Behavioral Pediatrics, 15(3), 191-197.

Lobato, D. (1985). Preschool siblings of handicapped children: Impact of peer support and training. Journal of Autism & Developmental Disorders, 15(3), 345-350.

Loper, A. B. (2006). How Do Mothers in Prison Differ From Non-Mothers? Journal of Child & Family Studies, 15(1), 83-95. doi: 10.1007/s10826-005-9005-x.

Lovko, A. M., & Ullman, D. G. (1989). Research on the adjustment of latchkey children: Role of background/demographic and latchkey situation variables. Journal of Clinical Child Psychology, 18, 16-24.

Loyd, B. H., & Abidin, R. R. (1985). Revision of the Parenting Stress Index. Journal of Pediatric Psychology, 10(2), 169-77.

Lumley, V. A., McNeil, C. B., Herschell, A. D., & Bahl, A. B. (2002). An examination of gender differences among young children with disruptive behavior disorders. Child Study Journal, 32(2), 89-100.

Lutzker, J. R., Bigelow, K. M., Doctor, R. M., & Kessler, M. L. (1998). Safety, health care, and bonding within an ecobehavioral approach to treating and preventing child abuse and neglect. Journal of Family Violence, 13(2), 163-185.

Lutzker, J. R., Vanhasselt, V. B., Bigelow, K. M., Greene, B. F., & Kessler, M. L. (1998). Child-Abuse and Neglect - Behavioral-Research, Treatment, and Theory. Aggression and Violent Behavior, 3(2), 181-196.

Lynn, T., Singer, P., Ann Salvator, M. S., Shenyang Guo, P., Marc Collin, M. D., Lawrence Lilien, M. D., & Jill Baley, M. D. (1999). Maternal Psychological Distress and Parenting Stress After the Birth of a Very Low-Birth-Weight Infant. Journal of the American Medical Association, 281(9), 799-805.

Macturk, R. H., Meadoworlans, K. P., Koester, L. S., & Spencer, P. E. (1993). Social Support, Motivation, Language, and Interaction - A Longitudinal-Study of Mothers and Deaf Infants. American Annals of the Deaf, 138(1), 19-25.

Magill-Evans, J., & Harrison, M. J. (1999). Parent-child interactions and development of toddlers born preterm. Western Journal of Nursing Research, 21(3), 292-307.

Magura, S., Laudet, A., Kang, S.-Y., & Whitney, S. A. (1999). Effectiveness of comprehensive services for crack-dependent mothers with newborns and young children. Journal of Psychoactive Drugs, 31(4), 321-338.

Mahoney, G., Perales, F., Wiggers, B., & Herman, B. (2006). Responsive Teaching: Early intervention for children with Down syndrome and other disabilities. DOWNS SYNDROME RESEARCH AND PRACTICE, 11(1), 18.

Mainemer, H., Gilman, L. C., & Ames, E. W. (1998). Parenting stress in families adopting children from Romanian orphanages. Journal of Family Issues, 19(2), 164-180.

Manassis, K., Bradley, S., Goldberg, S., Hood, J., & Swinson, R. P. (1994). Attachment in Mothers with Anxiety Disorders and Their Children. Journal of the American Academy of Child and Adolescent Psychiatry, 33(8), 1106-1113.

Manassis, K., Bradley, S., Goldberg, S., Hood, J., & Swinson, R. P. (1995). Behavioral-Inhibition, Attachment and Anxiety in Children of Mothers with Anxiety Disorders. Canadian Journal of Psychiatry, 40(2), 87-92.

Manion, I. G., Mcintyre, J., Firestone, P., Ligezinska, M., Ensom, R., & Wells, G. (1996). Secondary Traumatization in Parents Following the Disclosure of Extrafamilial Child Sexual Abuse - Initial Effects. Child Abuse & Neglect, 20(11), 1095-1109.

Marcus, R. F., & Kramer, C. (2001). Reactive and Proactive Aggression: Attachment and Social Competence Predictors. Journal of Genetic Psychology, 162(3), 260. doi: Article.

Markson, S., & Fiese, B. H. (2000). Family rituals as a protective factor for children with asthma. Journal of Pediatric Psychology, 25(7), 471-80.

Marshall, E., Buckner, E., Perkins, J., Lowry, J., Hyatt, C., Campbell, C., & Helms, D. (1996). Effects of child abuse prevention unit in health classes in four schools. Journal of Community Health Nursing, 13(2), 107-22.

Mash, E. J., & Johnston, C. (1983). The prediction of mothers' behavior with their hyperactive children during play and task situations. Child & Family Behavior Therapy, 5(2), 1-14.

Matheny, A. P. (1989). Assessment of Infant Mental-Development - Toward a Broader Perspective. Clinics in Perinatology, 16(2), 565-576.

Mattie-Luksic, M., Javornisky, G., & DiMario, F. J. (2000). Assessment of stress in mothers of children with severe breath-holding spells. Pediatrics, 106(1 Pt 1), 1-5.

McBride, B. A. (1991). Parental support programs and paternal stress: An exploratory study. Early Childhood Research Quarterly, 6(2), 137-140.

McCallum, M. S., & McKim, M. K. (1999). Recurrent otitis media and attachment security: A path model. Early Education and Development, 10(4), 517-534.

McCarthy, P., Sundby, M., Merladet, J., & Luxenberg, M. G. (1997). Identifying Attendance Correlates for a Teen and Young-Adult Parenting Program. Family Relations, 46(2), 107-112.

McCartney, K., Scarr, S., Rocheleau, A., Phillips, D., & Abbottshim, M. (1997). Teacher-Child Interaction and Child-Care Auspices as Predictors of Social Outcomes in Infants, Toddlers, and Preschoolers. Journal of Developmental Psychology, 43(3), 426-450.

McCarty, C., Waterman, J., Burge, D., & Edelstein, S. B. (1999). Experiences, concerns, and service needs of families adopting children with prenatal substance exposure: Summary and recommendations. Child Welfare, 78(5), 561-577.

McClowry, S. G., Giangrande, S. K., Tommasini, N. R., Clinton, W., Foreman, N. S., Lynch, K., & Ferketich, S. L. (1994). The effects of child temperament, maternal characteristics, and family circumstances on the maladjustment of school-age children. Research in Nursing & Health, 17(1), 25-35.

McKay, J. M., Pickens, J., & Stewart, A. L. (1996). Inventoried and observed stress in parent-child interactions. Current Psychology: Developmental, Learning, Personality, Social, 15(3), 223-234.

McNeil, C. B., Capage, I. C., Bahl, A., & Blanc, H. (1999). Importance of early intervention for disruptive behavior problems: Comparison of treatment and waitlist-control groups. Early Education & Development, 10(4), 445-454.

Meadow-Orlans, K. P. (1994). Stress, support, and deafness: Perceptions of infants' mothers and fathers. Journal of Early Intervention, 18(1), 91-102.

Meadow-Orlans, K. P. (1995). Sources of stress for mothers and fathers of deaf and hard of hearing infants. American Annals of the Deaf, 140(4), 352-7.

Meadow-Orlans, K. P., Smith-Gray, S., & Dyssegaard, B. (1995). Infants who are deaf or hard of hearing, with and without physical/cognitive disabilities. American Annals of the Deaf, 140(3), 279-86.

Meager, I., & Milgrom, J. (1996). Group treatment for postpartum depression: A pilot study. Australian & New Zealand Journal of Psychiatry, 30(6), 852-860.

Meijssen, D., Wolf, M., Koldewijn, K., van Wassenaer, A., Kok, J., & van Baar, A. (2010). Parenting stress in mothers after very preterm birth and the effect of the Infant Behavioural Assessment and Intervention Program. Child: care, health and development, 37, 195-202.

Meisels, S. J., & Liaw, F. R. (1993). Failure in Grade - Do Retained Students Catch-Up. Journal of Educational Research, 87(2), 69-77.

Messer, S. C., & Gross, A. M. (1995). Childhood Depression and Family-Interaction - A Naturalistic Observation Study. Journal of Clinical Child Psychology, 24(1), 77-88.

Metsch, L. R., Wolfe, H. P., Fewell, R., McCoy, C. B., Elwood, W. N., Wohler-Torres, B., et al. (2001). Treating Substance-Using Women and Their Children in Public Housing: Preliminary Evaluation Findings. Child Welfare, 80(2), 199-220. doi: Article.

Meyer, W. J., Blakeney, P., Moore, P., Murphy, L., Robson, M., & Herndon, D. (1994). Parental well-being and behavioral adjustment of pediatric survivors of burns. Journal of Burn Care & Rehabilitation, 15(1), 62-8.

Miceli, P. J., Goeke-Morey, M. C., Whitman, T. L., Kolberg, K. S., Miller-Loncar, C., & White, R. D. (2000). Brief report: birth status, medical complications, and social environment: individual differences in development of preterm, very low birth weight infants. Journal of Pediatric Psychology, 25(5), 353-8.

Miles, R., Cowan, F., Glover, V., Stevenson, J., & Modi, N. (2006). A controlled trial of skin-to-skin contact in extremely preterm infants. Early Human Development, 82(7), 447-455. doi: 10.1016/j.earlhumdev.2005.11.008.

Milgrom, J., & McCloud, P. (1996). Parenting Stress and Postnatal Depression. Stress Medicine, 12(3), 177-186.

Milgrom, J., Westley, D. T., & McCloud, P. I. (1995). Do Infants of Depressed Mothers Cry More Than Other Infants. Journal of Paediatrics and Child Health, 31(3), 218-221.

Miller, A. C., Pit-Ten Cate, I. M., Watson, H. S., & Geronemus, R. G. (1999). Stress and family satisfaction in parents of children with facial port-wine stains. Pediatric Dermatology, 16(3), 190-7.

Miller, C. L., Miceli, P. J., Whitman, T. L., & Borkowski, J. G. (1996). Cognitive Readiness to Parent and Intellectual-Emotional Development in Children of Adolescent Mothers. Developmental Psychology, 32(3), 533-541.

Milner, J. S. (1994). Assessing Physical Child-Abuse Risk - The Child-Abuse Potential Inventory. Clinical Psychology Review, 14(6), 547-583.

Milner, J. S., & Crouch, J. L. (1997). Impact and detection of response distortions on parenting measures used to assess risk for child physical abuse. Journal of Personality Assessment, 69(3), 633-50.

Milner, J. S., & Murphy, W. D. (1995). Assessment of Child Physical and Sexual Abuse Offenders. Family Relations, 44(4), 478-488.

Minter, K. R., Roberts, J. E., Hooper, S. R., Burchinal, M. R., & Zeisel, S. A. (2001). Early childhood otitis media in relation to children's attention-related behavior in the first six years of life. Pediatrics, 107(5), 1037-1042.

Misri, S., Reebye, P., Milis, L., & Shah, S. (2006). The impact of treatment intervention on parenting stress in postpartum depressed mothers: a prospective study. American Journal of Orthopsychiatry, 76(1), 115–119.

Mitchelson, J. K., & Burns, L. R. (1998). Career Mothers and Perfectionism - Stress at Work and at Home. Personality and Individual Differences, 25(3), 477-485.

Molfese, V., Rudasill, K., Beswick, J., Jacobi-Vessels, J., Ferguson, M., & White, J. (2010). Infant temperament, maternal personality, and parenting stress as contributors to infant developmental outcomes, Merrill-Palmer Quarterly, 56, 49-79.

Moore, C. G., Probst, J. C., Tompkins, M., Cuffe, S., & Martin, A. B. (2007). The prevalence of violent disagreements in US families: effects of residence, race/ethnicity, and parental stress. Pediatrics, 119, 68-76.

Moran, G., & Pederson, D. R. (1998). Proneness to distress and ambivalent relationships. Infant Behavior & Development, 21(3), 493-503.

Moran, G., Pederson, D. R., Pettit, P., & Krupka, A. (1992). Maternal Sensitivity and Infant Mother Attachment in a Developmentally Delayed Sample. Infant Behavior & Development, 15(4), 427-442.

Most, D. E., Fidler, D. J., Laforce-Booth, C., & Kelly, J. (2006). Stress trajectories in mothers of young children with Down syndrome. Journal of Intellectual Disability Research, 50(7), 501-514. doi: 10.1111/j.1365-2788.2006.00796.x.

Mott, S. E. (1986). Methods for assessing child and family outcomes in early childhood special education programs: Some views from the field. Topics in Early Childhood Special Education, 6(2), 1-15.

Mouton, P. Y., & Tuma, J. M. (1988). Stress, locus of control, and role satisfaction in clinic and control mothers. Journal of Clinical Child Psychology, 17(3), 217-224.

Mrazek, D. A., Mrazek, P., & Klinnert, M. (1995). Clinical-Assessment of Parenting. Journal of the American Academy of Child and Adolescent Psychiatry, 34(3), 272-282.

Msall, M. E., Rogers, B. T., Ripstein, H., Lyon, N., & Wilczenski, F. (1997). Measurements of Functional Outcomes in Children with Cerebral-Palsy. Mental Retardation and Developmental Disabilities Research Reviews, 3(2), 194-203.

Mudge, K., & Langley, J. (1998). Successful parenting: how to live with children. Community Practitioner, 71(9), 289-91.

Murphy, K. R., & Barkley, R. A. (1996). Parents of Children with Attention-Deficit Hyperactivity Disorder - Psychological and Attentional Impairment. American Journal of Orthopsychiatry, 66(1), 93-102.

Mylod, D. E., Whitman, T. L., & Borkowski, J. G. (1997). Predicting Adolescent Mothers Transition to Adulthood. Journal of Research on Adolescence, 7(4), 457-478.

Nachshen, J. S., & Minnes, P. (2005). Empowerment in parents of school-aged children with and without developmental disabilities. Journal of Intellectual Disability Research, 49(12), 889–904.

Nachshen, J. S., Garcin, N., & Minnes, P. (2005). Problem behavior in children with intellectual disabilities: Parenting stress, empowerment and school services. Mental Health Aspects of Developmental Disabilities, 8(4), 105.

Nakagawa, M., Teti, D. M., & Lamb, M. E. (1992). An ecological study of child-mother attachments among Japanese sojourners in the United States. Developmental Psychology, 28, 584-592.

Nam, Y., Meezan, W., & Danziger, S. K. (2006). Welfare recipients' involvement with child protective services after welfare reform. Child abuse & neglect, 30(11), 1181–1199.

Nereo, N. E., Fee, R. J., & Hinton, V. J. (2003). Parental stress in mothers of boys with Duchenne muscular dystrophy. Journal of Pediatric Psychology, 28(7), 473-484.

Nievar, M. A, Jacobson, A., & Dier, S. (2008, November). Home visiting for at-risk preschoolers: A successful model for Latino families. Paper presented at the Annual Meeting of the National Council on Family Relations, Little Rock, Arkansas.

Nixon, R. D. V. (2001). Changes in hyperactivity and temperament in behaviourally disturbed preschoolers after Parent-Child Interaction Therapy (PCIT). Behaviour Change, 18 (3), 168-176.

Nixon, R. D. V., Sweeney, L., Erickson, D. B., & Touyz, S. W. (2004). Parent-child interaction therapy: one- and two-year follow-up of standard and abbreviated treatments for oppositional preschoolers. Journal of Abnormal Child Psychology, 32(3), 263-271.

Nixon, R. D., Sweeney, L., Erickson, D. B., & Touyz, S. W. (2003). Parent-child interaction therapy: A comparison of standard and abbreviated treatments for oppositional defiant preschoolers. Journal of Consulting and Clinical Psychology, 71(2), 251–260.

Nock, M. K., & Kazdin, A. E. (2001). Parent expectancies for child therapy: Assessment and relation to participation in treatment. Journal of Child and Family Studies, 10(2), 155–180.

Olafsen, K. S., Rønning, J. A., Bredrup Dahl, L., Ulvund, S. E., Handegård, B. H., & Kaaresen, P. I.(2007). Infant responsiveness and maternal confidence in the neonatal period. Scandinavian Journal of Psychology, 48(6), 499-509.

Olsen, L. J., Allen, D., & Azzilessing, L. (1996). Assessing Risk in Families Affected by Substance-Abuse. Child Abuse & Neglect, 20(9), 833-842.

Olsen, R. D., Sande, J. R., & Olsen, G. P. (1991). Maternal parenting stress in physicians' families. Clinical Pediatrics, 30(10), 586-90.

Oneill, J. K., Oneill, P. J., Gothowens, T., Horn, B., & Cobb, L. M. (1996). Caregiver Evaluation of Anti-Gastroesophageal Reflux Procedures in Neurologically Impaired Children - What Is the Real-Life Outcome. Journal of Pediatric Surgery, 31(3), 375-380.

Ong, L. C., Afifah, I., Sofiah, A., & Lye, M. S. (1998). Parenting stress among mothers of Malaysian children with cerebral palsy: predictors of child- and parent-related stress. Annals of Tropical Paediatrics, 18(4), 301-7.

Ong, L. C., Chandran, V., & Boo, N. Y. (2001). Comparison of parenting stress between Malaysian mothers of four-year-old very low birthweight and normal birthweight children. Acta Paediatrica, 90(12), 1464-1469.

Ong, L., Chandran, V., & Peng, R. (1999). Stress experienced by mothers of Malaysian children with mental retardation. Journal of Paediatrics & Child Health, 35(4), 358-62.

Onufrak, B., Saylor, C. F., Taylor, M. J., Eyberg, S. M., & Boyce, G. C. (1995). Determinants of responsiveness in mothers of children with intraventricular hemorrhage. Journal of Pediatric Psychology, 20(5), 587-99.

Ooi, Y. P., Lam, C. M., Sung, M., Tan, W. T. S., Goh, T. J., Fung, D. S. S., et al. (2008). Effects of cognitive-behavioural therapy on anxiety for children with high-functioning autistic spectrum disorders. Singapore Medical Journal, 49(3), 215-220.

Orr, R. R., Cameron, S. J., Dobson, L. A., & Day, D. M. (1993). Age-related changes in stress experienced by families with a child who has developmental delays. Mental Retardation, 31(3), 171-6.

Osborne, L. A., & Reed, P. (2009). The Relationship Between Parenting Stress and Behavior Problems of Children With Autistic Spectrum Disorders. Exceptional Children, 76(1), 54-73.

Ostberg, M. (1998). Parental stress, psychosocial problems and responsiveness in help-seeking parents with small (2-45 months old) children. Acta Paediatrica, 87(1), 69-76.

Ostberg, M., & Hagekull, B. (2000). A structural modeling approach to the understanding of parenting stress. Journal of Clinical Child Psychology, 29(4), 615-25.

Ostberg, M., Hagekull, B., & Wettergren, S. (1997). A measure of parental stress in mothers with small children: dimensionality, stability and validity. Scandinavian Journal of Psychology, 38(3), 199-208.

Owen, M. T., & Mulvihill, B. A. (1994). Benefits of a Parent Education and Support Program in the 1st 3 Years. Family Relations, 43(2), 206-212.

Palmerus, K. (1997). Patterns of Swedish Parental Discipline. Nordisk Psykologi, 49(3), 212-230.

Panter, J. (1996). Developmental Observation Checklist System, by W. Hresko, S. Miquel, R. Sherbenou, S. Burton. Journal of Psychoeducational Assessment, 14(2), 178-186.

Parette, H. P., & Angelo, D. H. (1996). Augmentative and Alternative Communication Impact on Families - Trends and Future-Directions. Journal of Special Education, 30(1), 77-98.

Park, U. I. (1995). Patterns of infant-mother attachment and related variables. Korean Journal of Child Studies, 16(1), 113-131.

Passino, A. W., Whitman, T. L., Borkowski, J. G., Schellenbach, C. J., Maxwell, S. E., Keogh, D., & Rellinger, E. (1993). Personal Adjustment During Pregnancy and Adolescent Parenting. Adolescence, 28(109), 97-122.

Patterson, J., Barlow, J., Mockford, C., Klimes, I., Pyper, C., & Stewart-Brown, S. (2002). Improving mental health through parenting programmes: block randomized controlled trial. Archives of Disease in Childhood, 87(6), 472-477.

Patterson, K. A., & Starn, J. R. (1993). Program for women and infants exposed to drugs: a legal alternative. Nurse Practitioner Forum, 4(4), 224-30.

Paulussen-Hoogeboom, M. C., Stams, G. J. J. M., Hermanns, J. M. A., Peetsma, T. T. D., & Van Den Wittenboer, G. L. H. (2008). Parenting style as a mediator between children's negative emotionality and problematic behavior in early childhood. Journal of Genetic Psychology, 169(3), 209.

Pedersen S. D., Parsons, H. G., & Dewey, D. (2004). Stress levels experienced by the parents of enterally fed children. Chld: Care, Health & Development, 30(5), 507-513.

Peebles, C. D. (1997). The Infant and Preschool Psychiatry Clinic - A Model of Evaluation and Treatment. Infant Mental Health Journal, 18(2), 221-230.

Pelchat, D., & Lefebvre, H. (2004). A holistic intervention programme for families with a child with a disability. Journal of Advanced Nursing, 48(2), 124-131. doi: 10.1111/j.1365-2648.2004.03179.x.

Perepletchikova, F., & Kazdin, A. E. (2004). Assessment of Parenting Practices Related to Conduct Problems: Development and Validation of the Management of Children's Behavior Scale. Journal of Child & Family Studies, 13(4), 385-403. doi: Article.

Perrott, S. B., & Taylor, H. G. (1991). Neuropsychological sequelae, familial stress, and environmental adaptation. Developmental Neuropsychology, 7(1), 69-86.

Perry, A., Sarlo-McGarvey, N., & Factor, D. C. (1992). Stress and family functioning in parents of girls with Rett syndrome. Journal of Autism & Developmental Disorders, 22(2), 235-248.

Phipps, S., & Drotar, D. (1990). Determinants of parenting stress in home apnea monitoring. Journal of Pediatric Psychology, 15(3), 385-399.

Pincus, D. B., Eyberg, S. M., & Choate, M. L. (2005). Adapting Parent-Child Interaction Therapy for Young Children with Separation Anxiety Disorder. Education & Treatment of Children, 28(2), 163. doi: Article.

Pithers, W. D., Gray, A., Busconi, A., & Houchens, P. (1998). Caregivers of children with sexual behavior problems: Psychological and familial functioning. Child Abuse & Neglect, 22(2), 129-141.

Pithers, W. D., Gray, A., Busconi, A., & Houchens, P. (1998). Children with sexual behavior problems: identification of five distinct child types and related treatment considerations. Child Maltreatment, 3(4), 384-406.

Planos, R., Zayas, L. H., & Buschrossnagel, N. A. (1997). Mental-Health Factors and Teaching Behaviors Among Low-Income Hispanic Mothers. Families in Society-The Journal of Contemporary Human Services, 78(1), 4-12.

Powers, S. W., Byars, K. C., Mitchell, M. J., Patton, S. R., Standiford, D. A., & Dolan, L. M. (2002). Parent report of mealtime behavior and parenting stress in young children with type 1 diabetes and in healthy control subjects. Diabetes Care, 25(2), 313-318.

Quittner, A. L., Digirolamo, A. M., Michel, M., & Eigen, H. (1992). Parental Response to Cystic-Fibrosis - A Contextual Analysis of the Diagnosis Phase. Journal of Pediatric Psychology, 17(6), 683-704.

Radcliffe, J., Bennett, D., Kazak, A. E., Foley, B., & Phillips, P. C. (1996). Adjustment in childhood brain tumor survival: child, mother, and teacher report. Journal of Pediatric Psychology, 21(4), 529-39.

Raver, C. C., Gershoff, E. T., & Aber, J. L. (2007). Testing Equivalence of Mediating Models of Income, Parenting, and School Readiness for White, Black, and Hispanic Children in a National Sample. Child Development, 78(1), 96-115. doi: 10.1111/j.1467-8624.2007.00987.x.

Ray, D. C. (2008). Impact of play therapy on parent-child relationship stress at a mental health training setting. British Journal of Guidance & Counseling, 36(2), 165-187.

Realmuto, G. M., August, G. J., & Egan, E. A. (2004). Testing the goodness-of-fit of a multifaceted preventive intervention for children at risk for conduct disorder. Canadian Journal of Psychiatry. Revue Canadienne De Psychiatrie, 49(11), 743-752.

Reddihough, D. S., King, J., Coleman, G., & Catanese, T. (1998). Efficacy of Programs Based on Conductive Education for Young-Children with Cerebral-Palsy. Developmental Medicine and Child Neurology, 40(11), 763-770.

Reid, M. J., Walter, A. L., & Oleary, S. G. (1999). Treatment of Young Childrens Bedtime Refusal and Nighttime Wakings - A Comparison of Standard and Graduated Ignoring Procedures. Journal of Abnormal Child Psychology, 27(1), 5-16.

Rhodes, J. E., Ebert, L., & Fischer. (1992). Natural mentors: An overlooked resource in the social networks of young, African-American mothers. American Journal of Community Psychology, 20(4), 445-461.

Rhodes, J. E., Fischer, K., Ebert, L., & Meyers, A. B. (1993). Patterns of Service Utilization Among Pregnant and Parenting African-American Adolescents. Psychology of Women Quarterly, 17(3), 257-274.

Richardson, R. A., Barbour, N. E., & Bubenzer, D. L. (1995). Peer relationships as a source of support for adolescent mothers. Journal of Adolescent Research, 10(2), 278-290.

Richters, J. E. (1992). Depressed Mothers as Informants About Their Children - A Critical-Review of the Evidence for Distortion. Psychological Bulletin, 112(3), 485-499.

Rimmerman, A., Turkel, L., & Crossman, R. (2003). Perception of child development, child-related stress and dyadic adjustment: pair analysis of married couples of young children with developmental disabilities. Journal of Intellectual & Developmental Disability, 28(2), 188. doi: Article.

Ritchie, K. L., & Holden, G. W. (1998). Parenting stress in low income battered and community women: Effects on parenting behavior. Early Education and Development, 9(1), 98-112.

Robbins, F. R., & Dunlap, G. (1992). Effects of task difficulty on parent teaching skills and behavior problems of young children with autism. American Journal on Mental Retardation, 96(6), 631-643.

Robbins, F. R., Dunlap, G., & Plienis, A. J. (1991). Family characteristics, family training, and the progress of young children with autism. Journal of Early Intervention, 15(2), 173-184.

Roberts, A. C., & Nishimoto, R. (2006). Barriers to engaging and retaining African-American post-partum women in drug treatment. Journal of Drug Issues, 36(1), 53. doi: Article.

Roberts, M. C., Jacobs, A. K., Puddy, R. W., Nyre, J. E., & Vernberg, E. M. (2003). Treating children with serious emotional disturbances in schools and community: The intensive mental health program. Professional psychology, research and practice, 34(5), 519–526.

Robson, A. L. (1997). Low-Birth-Weight and Parenting Stress During Early-Childhood. Journal of Pediatric Psychology, 22(3), 297-311.

Rodd, J. (1994). Social Interest, Psychological Well-Being, and Maternal Stress. Individual Psychology, 50(1), 58-68.

Rodger, S., Keen, D., Braithwaite, M., & Cook, S. (2008). Mothers' Satisfaction with a Home Based Early Intervention Programme for Children with ASD. Journal of Applied Research in Intellectual Disabilities, 21(2), 174-182. doi: 10.1111/j.1468-3148.2007.00393.x.

Rodrigue, J. R., Hoffmann, R. G., Macnaughton, K., Grahampole, J., Andres, J. M., Novak, D. A., & Fennell, R. S. (1996). Mothers of Children Evaluated for Transplantation - Stress, Coping Resources, and Perceptions of Family Functioning. Clinical Transplantation, 10(5), 447-450.

Rodrigue, J. R., Macnaughton, K., Hoffmann, R. G., Grahampole, J., Andres, J. M., Novak, D. A., & Fennell, R. S. (1996). Perceptions of Parenting Stress and Family-Relations by Fathers of Children Evaluated for Organ-Transplantation. Psychological Reports, 79(3), 723-727.

Rodrigue, J. R., Macnaughton, K., Hoffmann, R. G., Grahampole, J., Andres, J. M., Novak, D. A., & Fennell, R. S. (1997). Transplantation in Children - A Longitudinal Assessment of Mothers Stress, Coping, and Perceptions of Family Functioning. Psychosomatics, 38(5), 478-486.

Rodriguez, C. M., & Green, A. J. (1997). Parenting stress and anger expression as predictors of child abuse potential. Child Abuse & Neglect, 21(4), 367-77.

Rosenstein, P. (1995). Parental Levels of Empathy as Related to Risk Assessment in Child Protective Services. Child Abuse & Neglect, 19(11), 1349-1360.

Ross, C. N., Blanc, H. M., McNeil, C. B., Eyberg, S. M., & Hembree-Kigin, T. L. (1998). Parenting stress in mothers of young children with oppositional defiant disorder and other severe behavior problems. Child Study Journal, 28(2), 93-110.

Rostain, A. L., Power, T. J., & Atkins, M. S. (1993). Assessing Parents Willingness to Pursue Treatment for Children with Attention-Deficit Hyperactivity Disorder. Journal of the American Academy of Child and Adolescent Psychiatry, 32(1), 175-181.

Ruckart, P. Z., Kakolewski, K., Bove, F. J., & Kaye, W. E. (2004). Long-Term Neurobehavioral Health Effects of Methyl Parathion Exposure in Children in Mississippi and Ohio. Environmental Health Perspectives, 112(1), 46-51. doi: Article.

Rudo, Z. H., Powell, D. S., & Dunlap, G. (1998). The Effects of Violence in the Home on Childrens Emotional, Behavioral, and Social Functioning - A Review of the Literature. Journal of Emotional and Behavioral Disorders, 6(2), 94-113.

Russell, D., & Matson, J. (1998). Fathers as Intervention Agents for Their Children with Developmental-Disabilities. Child & Family Behavior Therapy, 20(3), 29-49.

Sabatelli, R. M., & Waldron, R. J. (1995). Measurement Issues in the Assessment of the Experiences of Parenthood. Journal of Marriage and the Family, 57(4), 969-980.

Sadeh, A., Lavie, P., & Scher, A. (1994). Sleep and temperament: Maternal perceptions of temperament of sleep-disturbed toddlers. Early Education & Development, 5(4), 311-322.

Sanik, M. M. (1993). The Effects of Time Allocation on Parental Stress. Social Indicators Research, 30(2-3), 175-184.

Sarimski, K. (1995). Psychological aspects of the Prader-Willi syndrome: Results of a survey of parents by mail. Zeitschrift Fuer Kinder- und Jugendpsychiatrie, 23(4), 267-274.

Sarimski, K. (1996). Socioemotional Development and Parenting Stress in Williams-Beuren-Syndrome. Monatschrift Kinderheilkunde, 144(8), 838-842.

Sarimski, K. (1997). Behavioural phenotypes and family stress in three mental retardation syndromes. European Child & Adolescent Psychiatry, 6(1), 26-31.

Sarimski, K. (1997). Communication, Social-Emotional Development and Parenting Stress in Cornelia-de-Lange-Syndrome. Journal of Intellectual Disability Research, 41(FEB), 70-75.

Sarimski, K. (1997). Parenting Stress in Families with Craniofacially Disordered Children. Praxis Der Kinderpsychologie und Kinderpsychiatrie, 46(1), 2-14.

Sarimski, K. (1998). Children with Apert-Syndrome - Behavioral-Problems and Family Stress. Developmental Medicine and Child Neurology, 40(1), 44-49.

Sarimski, K. (1998). Stress in Mothers of Children with Genetic Syndromes. Zeitschrift fur Klinische Psychologie, Psychiatrie, und Psychotherapie, 46(3), 233-244.

Sarimski, K. (1998). The Soziale-Orientierungen-Von-Eltern-Behinderter-Kinder. Diagnostika, 44(3), 169-171.

Sawyer, G. K., Di Loreto, A. R., Flood, M. F., & DiLillo, D. (2002, November). Parent-Child Relationship and Family Variables as Predictors of Child Abuse Potential: Implications for Assessment and Early Intervention. Presented at the Annual Convention of the Association for the Advancement of Behavioral Therapy, Poster, Reno, Nevada. Retrieved November 19, 2009, .

Sawyer, G. K., Yancey, C. T., Tsao, E. H., Wynne, A., Hansen, D. J., & Flood, M. F. (2005). Parallel group treatments for sexually abused youth and their nonoffending parents: Treatment integrity, outcomes and social validity of Project SAFE. Presented at the 39th Annual Convention of the Association for Behavioral and Cognitive Therapies, Washington, DC.

Saylor, C. F., Casto, G., & Huntington, L. (1996). Predictors of Developmental Outcomes for Medically Fragile Early Intervention Participants. Journal of Pediatric Psychology, 21(6), 869-887.

Scheel, M. J., & Rieckmann, T. (1998). An empirically derived description of self-efficacy and empowerment for parents of children identified as psychologically disordered. American Journal of Family Therapy, 26(1), 15-27.

Schuhmann, E. M., Foote, R. C., Eyberg, S. M., Boggs, S. R., & Algina, J. (1998). Efficacy of Parent-Child Interaction Therapy - Interim-Report of a Randomized Trial with Short-Term Maintenance. Journal of Clinical Child Psychology, 27(1), 34-45.

Scott, B. S., Atkinson, L., Minton, H. L., & Bowman, T. (1997). Psychological Distress of Parents of Infants with Down-Syndrome. American Journal on Mental Retardation, 102(2), 161-171.

Seabrook, J. A., Gorodzinsky, F., & Freedman, S. (2005). Treatment of primary nocturnal enuresis: A randomized clinical trial comparing hypnotherapy and alarm therapy. Paediatrics & Child Health, 10(10), 609-610.

Secco, M. L., & Moffatt, M. (2003). Situational, maternal, and infant influences on parenting stress among adolescent mothers. Issues in Comprehensive Pediatric Nursing, 26(2), 103-122.

Sheeber, L. B. (1995). Empirical Dissociations Between Temperament and Behavior Problems - A Response. Journal of Developmental Psychology, 41(4), 554-561.

Sheeber, L. B., & Johnson, J. H. (1992). Child Temperament, Maternal Adjustment, and Changes in Family-Life Style. American Journal of Orthopsychiatry, 62(2), 178-185.

Sheeber, L. B., & Johnson, J. H. (1994). Evaluation of a Temperament-Focused, Parent-Training Program. Journal of Clinical Child Psychology, 23(3), 249-259.

Sheeran, T., Marvin, R. S., & Pianta, R. C. (1997). Mothers' resolution of their child's diagnosis and self-reported measures of parenting stress, marital relations, and social support. Journal of Pediatric Psychology, 22(2), 197-212.

Shelton, T. L., & Barkley, R. A. (1994). Critical Issues in the Assessment of Attention-Deficit Disorders in Children. Topics in Language Disorders, 14(4), 26-41.

Shelton, T. L., Barkley, R. A., Crosswait, C., Moorehouse, M., Fletcher, K., Barrett, S., Jenkins, L., & Metevia, L. (1998). Psychiatric and Psychological Morbidity as a Function of Adaptive Disability in Preschool-Children with Aggressive and Hyperactive-Impulsive-Inattentive Behavior. Journal of Abnormal Child Psychology, 26(6), 475-494.

Silver, E. J., Henegham, A. M., Bauman, L. J., & Stein, R. (2006). The relationship of depressive symptoms to parenting competence and social support in inner-city mothers of young children. Maternal & Child Health Journal, 10(1), 105-112.

Simoni, J., M. (1993). Latina mothers' help seeking at a school-based mutual support group. Journal of Community Psychology, 21, 188-199.

Singer, L. T., Davillier, M., Bruening, P., Hawkins, S., & Yamashita, T. S. (1996). Social Support, Psychological Distress, and Parenting Strains in Mothers of Very-Low-Birth-Weight Infants. Family Relations, 45(3), 343-350.

Singer, L. T., Salvator, A., Guo, S. Y., Collin, M., Lilien, L., & Baley, J. (1999). Maternal Psychological Distress and Parenting Stress After the Birth of a Very-Low-Birth-Weight Infant. Journal of the American Medical Association, 281(9), 799-805.

Singer, L. T., Song, L.-y., Hill, B. P., & Jaffe, A. C. (1990). Stress and depression in mothers of failure-to-thrive children. Journal of Pediatric Psychology, 15(6), 711-720.

Singh, D. K. (2003). Families of Head Start Children: A Research Connection. Journal of Instructional Psychology, 30(1), 77. doi: Article.

Singh, N. N., Lancioni, G. E., Winton, A. S., Singh, J., Curtis, W. J., Wahler, R. G., McAleavey, K. M. (2007). Mindful parenting decreases aggression and increases social behavior in children with developmental disabilities. Behavior Modification, 31(6), 749-771.

Slack, K. S., & Yoo, J. (2005). Food Hardship and Child Behavior Problems among Low-Income Children. Social Service Review, 79(3), 511-536. doi: 10.1086/430894.

Sokol, D. K., Ferguson, C. F., Pitcher, G. A., Huster, G. A., Fitzhughbell, K., & Luerssen, T. G. (1996). Behavioral-Adjustment and Parental Stress Associated with Closed-Head Injury in Children. Brain Injury, 10(6), 439-451.

Soliday, E., McCluskey-Fawcett, K., & O'Brien, M. (1999). Postpartum Affect and Depressive Symptoms in Mothers and Fathers. American Journal of Orthopsychiatry, 69(1), 30-38.

Solis, M. L., & Abidin, R. R. (1991). The Spanish version Parenting Stress Index: A psychometric study. Journal of Clinical Child Psychology, 20(4), 372-378.

Solomon, C. R., & Breton, J. (1999). Early warning signals in relationships between parents and young children with cystic fibrosis. Children's Health Care, 28(3), 221-40.

Sommer, K., Whitman, T. L., Borkowski, J. G., Schellenbach, C., Maxwell, S., & Keogh, D. (1993). Cognitive Readiness and Adolescent Parenting. Developmental Psychology, 29(2), 389-398.

Sontag, J. C. (1996). Toward a Comprehensive Theoretical Framework for Disability Research - Bronfenbrenner Revisited. Journal of Special Education, 30(3), 319-344.

Southard, N. A., & May, D. C. (1996). The Effects of Pre-First-Grade Programs on Student Reading and Mathematics Achievement. Psychology in the Schools, 33(2), 132-142.

Speltz, M. L., Endriga, M. C., Fisher, P. A., & Mason, C. A. (1997). Early Predictors of Attachment in Infants with Cleft-Lip and/or Palate. Child Development, 68(1), 12-25.

Speltz, M. L., Goodell, E. W., Endriga, M. C., & Clarren, S. K. (1994). Feeding Interactions of Infants with Unrepaired Cleft-Lip and or Palate. Infant Behavior & Development, 17(2), 131-139.

Sporakowski, M. J. (1995). Assessment and Diagnosis in Marriage and Family Counseling. Journal of Counseling and Development, 74(1), 60-64.

Stapleton, S. R., Drummond, J., Kysela, G. M., McDonald, L., Alexander, J., & Fleming, D. (1996). Team-building: making collaborative practice work. Risk and resiliency in two samples of Canadian families. Journal of Nurse-Midwifery, 4(1), 117-51.

Stewart, K. B., Aten, M., & Minkin, J. (1995). Caregiver-infant interactions in infants prenatally exposed to drugs. Paper presented at the American Occupational Therapy Association, Denver, Colorado.

Stewart, R. B. (1990). The second child: Family transition and adjustment. Newbury Park, CA: Sage Publications, Inc.

Stifter, C. A., Bono, M., & Spinrad, T. (2003). Parent characteristics and conceptualizations associated with the emergence of infant colic. Journal of Reproductive & Infant Psychology, 21(4), 309-322. doi: 10.1080/02646830310001622123.

Stiles, K. M., & Bellinger, D. C. (1993). Neuropsychological Correlates of Low-Level Lead-Exposure in School-Age-Children - A Prospective-Study. Neurotoxicology and Teratology, 15(1), 27-35.

Stoiber, K. C., & Houghton, T. G. (1993). The Relationship of Adolescent Mothers Expectations, Knowledge, and Beliefs to Their Young Childrens Coping Behavior. Infant Mental Health Journal, 14(1), 61-79.

Stoiber, K. C., & Houghton, T. G. (1994). Adolescent mothers' cognitions and behaviors as at-risk indicators. School Psychology Quarterly, 9(4), 295-316.

Strathearn, L., Fonagy, P., Amico, J., & Montague, P. R. (2009). Adult Attachment Predicts Maternal Brain and Oxytocin Response to Infant Cues. Neuropsychopharmacology, 1, 12.

Stuart, A., Moretz, M., & Yang, E. Y. (2000). An investigation of maternal stress after neonatal hearing screening. American Journal of Audiology, 9(2), 135-41.

Summers, M., Summers, C. R., & Ascione, F. R. (1993). A Comparison of Sibling Interaction in Intact and Single-Parent Families. Journal of Divorce & Remarriage, 20(1-2), 215-227.

Symons, D. K. (1998). Postpartum Employment Patterns, Family-Based Care Arrangements, and the Mother-Infant Relationship at Age-2. Canadian Journal of Behavioral Science, 30(2), 121-131.

Szatmari, P., Archer, L., Fisman, S., & Streiner, D. L. (1994). Parent and Teacher Agreement in the Assessment of Pervasive Developmental Disorders. Journal of Autism and Developmental Disorders, 24(6), 703-717.

Tam, K.-k., Chan, Y.-c., & Wong, C.-k. M. (1994). Validation of the Parenting Stress Index among Chinese mothers in Hong Kong. Journal of Community Psychology, 22(3), 211-223.

Tannock, R., Girolametto, L., & Siegel, L. S. (1992). Language intervention with children who have developmental delays: effects of an interactive approach. American Journal of Mental Retardation, 97(2), 145-60.

Tarabulsy, G. M., Avgoustis, E., Phillips, J., Pederson, D. R., & Moran, G. (1997). Similarities and Differences in Mothers and Observers Descriptions of Attachment Behaviors. International Journal of Behavior Development, 21(3), 599-619.

Tarbell, S. E., & Kosmach, B. (1998). Parental psychosocial outcomes in pediatric liver and/or intestinal transplantation: pretransplantation and the early postoperative period. Liver Transplantation & Surgery, 4(5), 378-87.

Tarkka, M. T., Paunonen, M., & Laippala, P. (1999). Factors Related to Successful Breast-Feeding by First-Time Mothers When the Child Is 3 Months Old. Journal of Advanced Nursing, 29(1), 113-118.

Taubman, B., Blum, N. J., & Nemeth, N. (2003). Stool toileting refusal: a prospective intervention targeting parental behavior. Archives of Pediatrics & Adolescent Medicine, 157(12), 1193-1196. doi: 10.1001/archpedi.157.12.1193

Taylor, H. G., Klein, N., Minich, N. M., & Hack, M. (2001). Long-term family outcomes for children with very low birth weights. Archives of Pediatrics & Adolescent Medicine, 155(2), 155-161.

Taylor, H. G., Klein, N., Schatschneider, C., & Hack, M. (1998). Predictors of Early School-Age Outcomes in Very-Low-Birth-Weight Children. Journal of Developmental and Behavioral Pediatrics, 19(4), 235-243.

Taylor, J. A., & Kemper, K. J. (1998). Group well-child care for high-risk families: maternal outcomes. Archives of Pediatrics & Adolescent Medicine, 152(6), 579-84.

Taylor, J. A., Davis, R. L., & Kemper, K. J. (1997). A Randomized Controlled Trial of Group Versus Individual Well Child-Care for High-Risk Children - Maternal-Child Interaction and Developmental Outcomes. Pediatrics, 99(6), E91-E96.

Telleen, S., Herzog, A., & Kilbane, T. L. (1989). Impact of a Family Support Program on Mothers Social Support and Parenting Stress. American Journal of Orthopsychiatry, 59(3), 410-419.

Teplin, S. W., Burchinal, M., Johnsonmartin, N., Humphry, R. A., & Kraybill, E. N. (1991). Neurodevelopmental, Health, and Growth Status at Age 6 Years of Children with Birth Weights Less Than 1001 Grams. Journal of Pediatrics, 118(5), 768-777.

Teti, D. M., Messinger, D. S., Gelfand, D. M., & Isabella, R. (1995). Maternal Depression and the Quality of Early Attachment - An Examination of Infants, Preschoolers, and Their Mothers. Developmental Psychology, 31(3), 364-376.

Teti, D. M., Nakagawa, M., Das, R., & Wirth, O. (1991). Security of attachment between preschoolers and their mothers: Relations among social interaction, parenting stress,

and mother's sorts of the Attachment Q-Set. Developmental Psychology, 27(3), 440-447.

Thomas, J. M., Benham, A. L., Gean, M., J., L., Minde, K., Turner, S., & Wright, H. H. (1997). Practice Parameters for the Psychiatric-Assessment of Infants and Toddlers (0-36 Months). Journal of the American Academy of Child and Adolescent Psychiatry, 36(10), S21-S36.

Thomas, J. M., Guskin, K. A., & Klass, C. S. (1997). Early Development Program - Collaborative Structures and Processes. Infant Mental Health Journal, 18(2), 198-208.

Timmer, S. G., Borrego, J., & Urquiza, A. J. (2002). Antecedents of Coercive Interactions in Physically Abusive Mother-Child Dyads. J Interpers Violence, 17(8), 836-853. doi: 10.1177/0886260502017008003.

Timmer, S. G., Llrquiza, A. I., Herschell, A. D., McGrath, J. M., Zebell, N. M., Porter, A. L., et al. (2006). Parent-Child Interaction Therapy: Application of an Empirically Supported Treatment to Maltreated Children in Foster Care. Child Welfare, 85(6), 919. doi: Article.

Timmer, S. G., Urquiza, A. J., & Zebell, N. (2006). Challenging foster caregiver–maltreated child relationships: The effectiveness of parent–child interaction therapy. Children and Youth Services Review, 28(1), 1-19. doi: 10.1016/j.childyouth.2005.01.006.

Timmer, S. G., Urquiza, A. J., Zebell, N. M., & McGrath, J. M. (2005). Parent-child interaction therapy: application to maltreating parent-child dyads. Child Abuse & Neglect, 29(7), 825-842. doi: 10.1016/j.chiabu.2005.01.003

Tripp, G., Schaughency, E., Langlands, R., & Mouat, K. (2007). Family Interactions in Children With and Without ADHD. Journal of Child & Family Studies, 16(3), 385-400. doi: 10.1007/s10826-006-9093-2.

Tsang, S., Tsang, S., Chan, E., & Lee, C. (1992). Stress of parents with normal and special pre-school children: A comparison study. Bulletin of the Hong Kong Psychological Society, 28, 63-79.

Tucker, S., Gross, D., Fogg, L., Delaney, K., & Lapporte, R. (1998). The long-term efficacy of a behavioral parent training intervention for families with 2-year-olds. Research in Nursing & Health, 21(3), 199-210.

Tynan, W. D., & Nearing, J. (1994). The diagnosis of attention deficit hyperactivity disorder in young children. Infants & Young Children, 6(4), 13-20.

Uno, D., Florsheim, P., & Uchino, B. N. (1998). Psychosocial Mechanisms Underlying Quality of Parenting Among Mexican-American and White Adolescent Mothers. Journal of Youth and Adolescence, 27(5), 585-605.

Van Hasselt, V. B., Hersen, M., Null, J. A., Ammerman, R. T., Bukstein, O. G., Mcgillivray, J., & Hunter, A. (1993). Drug-Abuse Prevention for High-Risk African-American Children and Their Families - A Review and Model Program. Addictive Behaviors, 18(2), 213-234.

Veddovi, M., Gibson, F., Kenny, D., Bowen, J., & Starte, D. (2004). Preterm behavior, maternal adjustment, and competencies in the newborn period: What influence do they have at 12 months postnatal age? Infant Mental Health Journal, 25(6), 580-599. doi: 10.1002/imhj.20026.

Vermaes, I. P. R., Janssens, J. M. A. M., Mullaart, R. A., Vinck, A., & Gerris, J. R. M. (2008). Parents' personality and parenting stress in families of children with spina bifida. Child: Care, Health & Development, 34(5),665-674.

Victor, A. M., Bernat, D. H., Bernstein, G. A., & Layne, A. E. (2007). Effects of parent and family characteristics on treatment outcome of anxious children. Journal of Anxiety Disorders, 21(6), 835-848. doi: 10.1016/j.janxdis.2006.11.005.

Vieira Santos, S. (1994). Characteristics of stress in parents of children with chronic diseases and in parents of children with emotional problems. Analise Psicologica, 12(2-3).

Vieira Santos, S., Ataide, S., & Joao, P. (1996). Parental stress in parents of children with obesity and with chronic renal disease. Analise Psicologica, 14(2-3), 231-243.

Virtanen, T., & Moilanen, I. (1991). Stress and coping in mothers of children with minimal brain dysfunction. Praxis der Kinderpsychologie und Kinderpsychiatrie, 40(7), 260-265.

Vohr, B., Jodoin-Krauzyk, J., Tucker, R., Johnson, M. J., Topol, D., & Ahlgren, M. (2008). Early language outcomes of early-identified infants with permanent hearing loss at 12 to 16 months of age. Pediatrics, 122(3), 535-544. doi: 10.1542/peds.2007-2028.

Volenski, L. T. (1995). Building school support systems for parents of handicapped children: The parent education and guidance program. Psychology in the Schools, 32(2), 124-129.

Volling, B. L., Notaro, P. C., & Larsen, J. J. (1998). Adult Attachment Styles - Relations with Emotional Well-Being, Marriage, and Parenting. Family Relations, 47(4), 355-367.

Vorria, P., Papaligoura, Z., Sarafidou, J., Kopakaki, M., Dunn, J., Van IJzendoorn, M. H., Kontopoulou, A. (2006). The development of adopted children after institutional care: A follow-up study. Journal of Child Psychology and Psychiatry, 47(12), 1246–1253.

Waldron, A., Tobin, G., & McQuaid, P. (2001). Mental health status of homeless children and their families. Irish Journal of Psychological Medicine, 18(1), 11-15.

Walker, C. D. (1999). Stress in parents of children with ADHD vs. depression: A multicultural analysis. Dissertation Abstracts International: Section B: the Sciences & Engineering, 59(7-B).

Wall, J. E., & Holden, E. W. (1994). Aggressive, Assertive, and Submissive Behaviors in Disadvantaged, Inner-City Preschool-Children. Journal of Clinical Child Psychology, 23(4), 382-390.

Wanamaker, C. E., & Glenwick, D. S. (1998). Stress, Coping, and Perceptions of Child-Behavior in Parents of Preschoolers with Cerebral-Palsy. Rehabilitation Psychology, 43(4), 297-312.

Wang, H., & Jong, Y. (2004). Parental Stress and Related Factors in Parents of Children with Cerebral Palsy. The Kaohsiung Journal of Medical Sciences, 20(7), 334-340. doi: 10.1016/S1607-551X(09)70167-6.

Warfield, M. E. (1995). The Cost-Effectiveness of Home Visiting Versus Group Services in Early Intervention. Journal of Early Intervention, 19(2), 130-148.

Warfield, M. E., Krauss, M. W., Hauser-Cram, P., Upshur, C. C., & Shonkoff, J. P. (1999). Adaptation during early childhood among mothers of children with disabilities. Journal of Developmental & Behavioral Pediatrics, 20(1), 9-16.

Warren, S. F., Fey, M. E., Finestack, L. H., Brady, N. C., Bredin-Oja, S. L., & Fleming, K. K. (2008). A Randomized Trial of Longitudinal Effects of Low-Intensity Responsivity Education/Prelinguistic Milieu Teaching. Journal of Speech, Language & Hearing Research, 51(2), 451-470. doi: 10.1044/1092-4388(2008/033).

Webster-Stratton, C. (1988). Mothers' and fathers' perceptions of child deviance: Roles of parent and child behaviors and parent adjustment. Journal of Consulting & Clinical Psychology, 56(6), 909-915.

Webster-Stratton, C. (1994). Advancing Videotape Parent Training - A Comparison Study. Journal of Consulting and Clinical Psychology, 62(3), 583-593.

Webster-Stratton, C. (1996). Early-Onset Conduct Problems - Does Gender Make a Difference. Journal of Consulting and Clinical Psychology, 64(3), 540-551.

Webster-Stratton, C., & Hammond, M. (1997). Treating Children with Early-Onset Conduct Problems - A Comparison of Child and Parent Training Interventions. Journal of Consulting and Clinical Psychology, 65(1), 93-109.

Webster-Stratton, C., & Herman, K. C. (2008). The Impact of Parent Behavior-Management Training on Child Depressive Symptoms. Journal of Counseling Psychology, 55(4), 473-484.

Webster-Stratton, C., & Spitzer, A. (1996). Parenting a Young-Child with Conduct Problems - New Insights Using Qualitative Methods. Advances in Clinical Child Psychology, 18, 1-62.

Weinfield, N. S., Ogawa, J. R., & Egeland, B. (2002). Predictability of observed mother-child interaction from preschool to middle childhood in a high-risk sample. Child development, 528–543.

Weinreb, L., Goldberg, R., Bassuk, E., & Perloff, J. (1998). Determinants of Health and Service Use Patterns in Homeless and Low-Income Housed Children. Pediatrics, 102(3), 554-562.

Weinreb, M., & Varda, K. (1996). Birthmothers: A retrospective analysis of the surrendering experience. Psychotherapy in Private Practice, 15(1), 59-70.

Weinstein, P., Domoto, P., Wohlers, K., & Koday, M. (1992). Mexican-American Parents with Children at Risk for Baby Bottle Tooth-Decay - Pilot-Study at a Migrant Farmworkers Clinic. Journal of Dentistry for Children, 59(5), 376-383.

Weiss, J. A., & Diamond, T. (2005). Stress in parents of adults with intellectual disabilities attending Special Olympics competitions. Journal of Applied Research in Intellectual Disabilities, 18(3), 263-270.

Weiss, J. A., Sullivan, A., & Diamond, T. (2003). Parent stress and adaptive functioning of individuals with developmental disabilities. Journal on Developmental Disabilities, 10(1), 129-136.

Weiss, K. L., Marvin, R. S., & Pianta, R. C. (1997). Ethnographic detection and description of family strategies for child care: Applications to the study of cerebral palsy. Journal of Pediatric Psychology, 22(2), 263-278.

Wells, R. D., & Schwebel, A. I. (1987). Chronically ill children and their mothers: Predictors of resilience and vulnerability to hospitalization and surgical stress. Journal of Developmental & Behavioral Pediatrics, 8(2), 83-89.

Whipple, E. E. (1999). Reaching Families with Preschoolers at Risk of Physical Child-Abuse - What Works. Families in Society-The Journal of Contemporary Human Services, 80(2), 148-160.

Whitley, D. M., White, K. R., Kelley, S. J., & Yorke, B. (1999). Strengths-Based Case-Management - The Application to Grandparents Raising Grandchildren. Families in Society-The Journal of Contemporary Human Services, 80(2), 110-119.

Wiener, L. S., Vasquez, M. J., & Battles, H. B. (2001). Brief report: fathering a child living with HIV/AIDS: psychosocial adjustment and parenting stress. Journal of Pediatric Psychology, 26(6), 353-358.

Willinger, U., Diendorfer-Radner, G., Willnauer, R., Jörgl, G., & Hager, V. (2005). Parenting stress and parental bonding. Behavioral Medicine, 31(2), 63-69.

Winton, M. A. (1990). An evaluation of a support group for parents who have a sexually abused child. Child Abuse & Neglect, 14(3), 397-405.

Wittmer, D., Doll, B., & Strain, P. (1996). Social and Emotional Development in Early-Childhood - The Identification of Competence and Disabilities. Journal of Early Intervention, 20(4), 299-317.

Wolf, L. C., & Fisman, N. S. (1989). Psychological effects of parenting stress on parents of autistic children. Journal of Autism & Developmental Disorders, 19(1), 157-166.

Wolf, L. C., Fisman, S., Ellison, D., & Freeman, T. (1998). Effect of Sibling Perception of Differential Parental Treatment in Sibling Dyads with One Disabled-Child. Journal of the American Academy of Child and Adolescent Psychiatry, 37(12), 1317-1325.

Wolock, I., & Magura, S. (1996). Parental Substance-Abuse as a Predictor of Child Maltreatment Re-Reports. Child Abuse & Neglect, 20(12), 1183-1193.

Wright, M., Crawford, E., & Sebastian, K. (2007). Positive Resolution of Childhood Sexual Abuse Experiences: The Role of Coping, Benefit-Finding and Meaning-Making. Journal of Family Violence, 22(7), 597-608. doi: 10.1007/s10896-007-9111-1.

Wyman, P. A., Moynihan, J., Eberly, S., Cox, C., Cross, W., Jin, X., et al. (2007). Association of Family Stress With Natural Killer Cell Activity and the Frequency of Illnesses in Children. Arch Pediatr Adolesc Med, 161(3), 228-234. doi: 10.1001/archpedi.161.3.228.

Wysocki, T., Huxtable, K., Linscheid, T. R., & Wayne, W. (1989). Adjustment to diabetes mellitus in preschoolers and their mothers. Diabetes Care, 12(8), 524-9.

Youngblut, J. M., Singer, L. T., Madigan, E. A., Swegart, L. A., & Rodgers, W. L. (1998). Maternal employment and parent-child relationships in single-parent families of low-birth-weight preschoolers. Nursing Research, 47(2), 114-21.

Younger, J. B. (1991). A model of parenting stress. Research in Nursing & Health, 14(3), 197-204.

Younger, J. B. (1993). Development and Testing of the Mastery of Stress Instrument. Nursing Research, 42(2), 68-73.

Zalaquett, C. P., & Wood, R. J. (1997). Evaluating stress: A book of resources. Lanham, MD: Scarecrow Press, Inc.

Zeitz, P., Kakolewski, K., Imtiaz, R., & Kaye, W. (2002). Methods of assessing neurobehavioral development in children exposed to methyl parathion in Mississippi and Ohio. Environmental Health Perspectives, 110 Suppl 6, 1079-1083.

Zelazo, P. R. (1997). Infant-Toddler Information-Processing Treatment of Children with Pervasive Developmental Disorder and Autism .2. Infants and Young Children, 10(2), 1-13.

Short Form References

Altmaier, E., & Maloney, R. (2007). An initial evaluation of a mindful parenting program. Journal of Clinical Psychology, 63(12), 1231-1238. doi: 10.1002/jclp.20395.

Anderson, L. S. (2008). Predictors of Parenting Stress in a Diverse Sample of Parents of Early Adolescents in High-Risk Communities. Nursing Research, 57(5), 340-350. doi: 10.1097/01.NNR.0000313502.92227.87.

Badr, L. K., Bookheimer, S., Purdy, I., & Deeb, M. (2009). Predictors of neurodevelopmental outcome for preterm infants with brain injury: MRI, medical and environmental factors. Early human development, 85(5), 279–284.

Badr, L. K., Garg, M., & Kamath, M. (2006). Intervention for infants with brain injury: Results of a randomized controlled study. Infant Behavior and Development, 29(1), 80-90. doi: 10.1016/j.infbeh.2005.08.003.

Bagner, D. M., & Eyberg, S. M. (2007). Parent—Child Interaction Therapy for Disruptive Behavior in Children with Mental Retardation: A Randomized Controlled Trial. Journal of Clinical Child & Adolescent Psychology, 36(3), 418-429. doi: 10.1080/15374410701448448.

Baker, A. J. L., Gries, L., Schneiderman, M., Parker, R., Archer, M., & Friedrich, B. (2008). Children with Problematic Sexualized Behaviors in the Child Welfare System. Child Welfare, 87(1), 5. doi: Article.

Barnes, J., Sutcliffe, A. G., Kristoffersen, I., Loft, A., Wnnerholm, U., Tarlatzis, B. C., … Bonduelle, M. (2004). The influence of assisted reproduction on family functioning and children's socio-emotional development: results from a European study. Human Reproduction, 19(6), 1480-1487.

Barnet, B., Duggan, A. K., Devoe, M., & Burrell, L. (2002). The effect of volunteer home visitation for adolescent mothers on parenting and mental health outcomes: A randomized trial. Archives of Pediatrics & Adolescent Medicine, 156(12), 1216-1222.

Beck, A., Hastings, R. P., Daley, D., & Stevenson, J. (2004). Pro-social behaviour and behaviour problems independently predict maternal stress. Journal of Intellectual and Developmental Disability, 29(4), 339–349.

Belanger, K., Copeland, S., & Cheung, M. (2008). The Role of Faith in Adoption: Achieving Positive Adoption Outcomes for African American Children. Child Welfare, 87(2), 99. doi: Article.

Berz, J. B., Carter, A. S., Wagmiller, R. L., Horwitz, S. M., Murdock, K. K., & Briggs-Gowan, M. (2007). Prevalence and Correlates of Early Onset Asthma and Wheezing in

a Healthy Birth Cohort of 2- to 3-Year Olds. J. Pediatr. Psychol., 32(2), 154-166. doi: 10.1093/jpepsy/jsj123.

Bhavnagri, N. P. (1999). Low income African American mothers' parenting stress and instructional strategies to promote peer relationships in preschool children. Early Education & Development, 10(4), 551-571.

Boukydis, C. Z., & Lester, B. M. (2008). Mother-infant consultation during drug treatment: Research and innovative clinical practice. Harm Reduction Journal, 5(6),

Briggs-Gowan, M. J., Carter, A. S., Moye Skuban, E., & McCue Horwitz, S. (2001). Prevalence of Social-Emotional and Behavioral Problems in a Community Sample of 1- and 2-year-old Children. Journal of the American Academy of Child & Adolescent Psychiatry, 40(7), 811-819.

Britner, P. A., Morog, M. C., Pianta, R. C., & Marvin, R.S. (2003). Stress and coping: a comparison of self-report measures of functioning in families of young children with cerebral palsy or no medical diagnosis. Journal of Child and Family Studies, 12(3), 335-348.

Brown, N. L., & Bhavnagri, N. (1996). Effects of an Early Intervention Program on Stress and Teaching Ability of Single Mothers of Young Multiply Impaired Children (ED398678).

Burbach, A. D., Fox, R. A., & Nicholson, B. C. (2004). Challenging Behaviors in Young Children: The Father's Role. Journal of Genetic Psychology, 165(2), 169. doi: Article.

Button, S., Pianta, R. C., & Marvin, R. S. (2001). Mothers' representations of relationships with their children: Relations with parenting behavior, mother characteristics, and child disability status. Social Development, 10(4), 455-472. doi: 10.1111/1467-9507.00175

Button, S., Pianta, R. C., & Marvin, R. S. (2001). Partner support and maternal stress in families raising young children with cerebral palsy. Journal of Developmental & Physical Disabilities, 13(1), 61-81.

Butz, A. M., Pulsifer, M., Marano, N., Belcher, H., Lears, M. K., & Royall, R. (2001). Effectiveness of a home intervention for perceived child behavioral problems and parenting stress in children with in utero drug exposure. Archives of Pediatrics & Adolescent Medicine, 155(9), 1029-1037.

Cain, D. S., & Combs-Orme, T. (2005). Family structure effects on parenting stress and practices in the African American family. Journal of Sociology and Social Welfare, 32(2), 19-40.

Calkins, S. D., Hungerford, A., & Dedmon, S. E. (2004). Mothers' interactions with temperamentally frustrated infants. Infant Mental Health Journal, 25(3), 219–239.

Carlton-Conway, D., Ahluwalia, R., Henry, L., Michie, C., Wood, L., & Tulloh, R. (2005). Behaviour sequelae following acute Kawasaki disease. BMC Pediatrics, 5(1), 14. doi: 10.1186/1471-2431-5-14.

Chacko, A., Wymbs, B. T., Flammer, L. M., Pelham, W. E., Walker, K. S., Arnold, F. W., …Herbst, L.(2008). A pilot study of the feasibility and efficacy of the Strategies to Enhance Positive Parenting (STEPP) Program for single mothers of children with ADHD. Journal of Attention Disorders, 12(3), 270-280.

Chartrand, M. M., Frank, D. A., White, L. F., & Shope, T. R. (2008). Effect of Parents' Wartime Deployment on the Behavior of Young Children in Military Families. Arch Pediatr Adolesc Med, 162(11), 1009-1014. doi: 10.1001/archpedi.162.11.1009.

Chazan-Cohen, R., Ayoub, C., Pan, B. A., Roggman, L., Raikes, H., Mckelvey, L., et al. (2007). It takes time: Impacts of Early Head Start that lead to reductions in maternal depression two years later. Infant Mental Health Journal, 28(2), 151-170. doi: 10.1002/imhj.20127.

Chisholm, V., Atkinson, L., Donaldson, C., Noyes, K., Payne, A., & Kelnar, C. (2007). Predictors of treatment adherence in young children with type 1 diabetes. Journal of Advanced Nursing, 57(5), 482-493. doi: 10.1111/j.1365-2648.2006.04137.x.

Cole, S. (2005). Infants in foster care: Relational and environmental factors affecting attachment. JOURNAL OF REPRODUCTIVE AND INFANT PSYCHOLOGY, 23, 43-61.

Cole, S. A. (2002). Security of Attachment of Infants in Foster Care. In Proceedings of the... National Symposium on Doctoral Research in Social Work (p. 12). Presented at the National Symposium on Doctoral Research in Social Work, Columbus, OH: Ohio State University.

Colver, A. (2006). Study protocol: SPARCLE -- a multi-centre European study of the relationship of environment to participation and quality of life in children with cerebral palsy. BMC Public Health, 6, 105-110.

Combs-Orme, T., Cain, D. S., & Wilson, E. E. (2004). Do maternal concerns at delivery predict parenting stress during infancy? Child Abuse & Neglect, 28(4), 377–392.

Copeland, S., & Harbaugh, B. L. (2005). Differences in parenting stress between married and single first time mothers at six to eight weeks after birth. Issues in Comprehensive Pediatric Nursing, 28(3), 139-152.

Cowen, P. S. (1998). Crisis child care: An intervention for at-risk families. Issues in Comprehensive Pediatric Nursing, 21(3), 147-158.

Dadds, M., & Roth, J. (2008). Prevention of Anxiety Disorders: Results of a Universal Trial with Young Children. Journal of Child & Family Studies, 17(3), 320-335. doi: 10.1007/s10826-007-9144-3.

Dawe, S., Harnett, P. H., Rendalls, V., & Staiger, P. (2003). Improving family functioning and child outcome in methadone maintained families: the Parents Under Pressure programme. Drug and Alcohol Review, 22(3), 299–307.

Day, C., & Davis, H. (2006). The effectiveness and quality of routine child and adolescent mental health care outreach clinics. British Journal of Clinical Psychology, 45(4), 439-452. doi: 10.1348/014466505X79986.

Deater-Deckard, K. (1996). Within Family Variability in Parental Negativity and Control. Journal of Applied Developmental Psychology, 17(3), 407-422.

Deater-Deckard, K., & Scarr, S. (1996). Parenting Stress Among Dual-Earner Mothers and Fathers - Are There Gender Differences. Journal of Family Psychology, 10(1), 45-59.

Deater-Deckard, K., Pinkerton, R., & Scarr, S. (1996). Child-Care Quality and Childrens Behavioral-Adjustment - A 4-Year Longitudinal-Study. Journal of Child Psychology and Psychiatry and Allied Disciplines, 37(8), 937-948.

DeCaro, J. A., & Worthman, C. M. (2008). Return to school accompanied by changing associations between family ecology and cortisol. Developmental Psychobiology, 50(2), 183–195.

DeMore, M., Adams, C., Wilson, N., & Hogan, M. B. (2005). Parenting Stress, Difficult Child Behavior, and Use of Routines in Relation to Adherence in Pediatric Asthma. Children's Health Care, 34(4), 245-259. doi: 10.1207/s15326888chc3404_1.

Dempsey, I., & Keen, D. (2008). A Review of Processes and Outcomes in Family-Centered Services for Children With a Disability. Topics in Early Childhood Special Education, 28(1), 42-52. doi: 10.1177/0271121408316699.

DePanfilis, D., & Dubowitz, H. (2005). Family connections: a program for preventing child neglect. Child Maltreatment, 10(2), 108-123. doi: 10.1177/1077559505275252.

Dombrowski, S. C., Timmer, S. G., Blacker, D. M., & Urquiza, A. J. (2005). A positive behavioural intervention for toddlers: parent-child attunement therapy. Child Abuse Review, 14(2), 132-151.

Doubleday, E., & Hey, C. (2004). Is statistical significance enough? Behavioural parent training programmes in real life. Clinical Psychology, 37, 5-9.

Douglas, A. R. (2000). Reported anxieties concerning intimate parenting in women sexually abused as children. Child Abuse & Neglect, 24(3), 425-34.

Drummond, J., McDonald, L., MacKenzie-Keating, S., & Fleming, D. (2004). Types of support accessed by families of young children with disabilities living in Alberta. Developmental Disabilities Bulletin, 32(1), 1-27.

Duggan, A., Caldera, D., Rodriguez, K., Burrell, L., Rohde, C., & Crowne, S. S. (2007). Impact of a statewide home visiting program to prevent child abuse. Child Abuse & Neglect, 31(8), 801-827. doi: 10.1016/j.chiabu.2006.06.011.

Duguid, A., Morrison, S., Robertson, A., Chalmers, J., Youngson, G., & Ahmed, S. F. (2007). The psychological impact of genital anomalies on the parents of affected children. Acta Paediatrica, 96(3), 348-352. doi: 10.1111/j.1651-2227.2006.00112.x.

Dumas, J. E., Nissley, J., Nordstrom, A., Smith, E. P., Prinz, R. J., & Levine, D. W. (2005). Home Chaos: Sociodemographic, Parenting, Interactional, and Child Correlates. Journal of Clinical Child & Adolescent Psychology, 34(1), 93-104. doi: 10.1207/s15374424jccp3401_9.

Eccleston, C., Malleson, P. N., Clinch, J., Connell, H., & Sourbut, C. (2003). Chronic pain in adolescents: evaluation of a programme of interdisciplinary cognitive behaviour therapy. Archives of Disease in Childhood, 88(10), 881-885.

Eddy, M. E., Carter, B. D., Kronenberger, W. G., Conradsen, S., Eid, N. S., Bourland, S. L., & Adams, G. (1998). Parent relationships and compliance in cystic fibrosis. Journal of Pediatric Health Care, 12(4), 196-202.

Edelson, M., Hokoda, A., & Ramos-Lira, L. (2007). Differences in Effects of Domestic Violence Between Latina and Non-Latina Women. Journal of Family Violence, 22(1), 1-10. doi: 10.1007/s10896-006-9051-1.

Epstein, T., Saltzman-Benaiah, J., O'Hare, A., Goll, J. C., & Tuck, S. (2008). Associated features of Asperger Syndrome and their relationship to parenting stress. Child: Care, Health & Development, 34(4),503-511.

Evangelou, M., Brooks, G., & Smith, S. (2007). The Birth to School Study: evidence on the effectiveness of PEEP, an early intervention for children at risk of educational under-achievement. Oxford Review of Education, 33(5), 581-609. doi: 10.1080/03054980701476477.

Fedele, D., Grant, D., Wolfe-Christensen, C., Mullins, L., & Ryan, J. (2010). An examination of the factor structure of parenting capacity measures in chronic illness populations, Journal of Pediatric Psychology, 35, 1083-1092.

Feldman, R., Eidelman, A. I., & Rotenberg, N. (2004). Parenting Stress, Infant Emotion Regulation, Maternal Sensitivity, and the Cognitive Development of Triplets: A Model for Parent and Child Influences in a Unique Ecology. Child Development, 75(6), 1774-1791. doi: 10.1111/j.1467-8624.2004.00816.x.

Feldman, R., Granat, A., Pariente, C., Kanety, H., Kuint, J., & Gilboa-Schechtman, E. (2009). Maternal Depression and Anxiety Across the Postpartum Year and Infant Social Engagement, Fear Regulation, and Stress Reactivity. Journal of Amer Academy of Child & Adolescent Psychiatry, 48(9), 919 - 927.

Flake, E., Davis, B., Johnson, P., & Middleton, L. (2009). The Psychosocial Effects of Deployment on Military Children, Journal of Developmental & Behavioral Pediatrics, 30, 271-278.

Flykt, M., Lindblom, J., Punamaki, R., Poikkeus, P., Repokari, L., Unkila-Kallio, L., Vilska, S., Sinkkonen, J., Tiitinen, A., Almqvist, F., & Tulppala, M. (2009). Prenatal expectations in transition to parenthood: Former infertility and family dynamic considerations. Journal of Family Psychology, 23, 779-789.

Forde, H., Lane, H., McCloskey, D., McManus, V., & Tierney, E. (2004). Link Family Support- an evaluation of an in-home support service. Journal of Psychiatric & Mental Health Nursing, 11(6), 698-704.

Gavin, L., & Wysocki, T. (2006). Associations of paternal involvement in disease management with maternal and family outcomes in families with children with chronic illness. Journal of Pediatric Psychology, 31(5), 481-489.

Goldberg, S., Janus, M., Washington, J., Simmons, R. J., Maclusky, I., & Fowler, R. S. (1997). Prediction of Preschool Behavioral-Problems in Healthy and Pediatric Samples. Journal of Developmental and Behavioral Pediatrics, 18(5), 304-313.

Golombok, S., Jadva, V., Lycett, E., Murray, C., & MacCallum, F. (2005). Families created by gamete donation: follow-up at age 2. Human Reproduction, 20(1), 286-293.

Golombok, S., Murray, C., Jadva, V., Lycett, E., MacCallum, F., & Rust, J. (2006). Non-genetic and non-gestational parenthood: consequences for parent-child relationships and the psychological well-being of mothers, fathers and children at age 3. Human Reproduction, 21(7), 1918-1924. doi: 10.1093/humrep/del039

Golombok, S., Murray, C., Jadva, V., MacCallum, F., & Lycett, E. (2004). Families created through surrogacy arrangements: parent-child relationships in the 1st year of life. Developmental Psychology, 40(3), 400-411. doi: 10.1037/0012-1649.40.3.400.

Golombok, S., Olivennes, F., Ramogida, C., Rust, J., & Freeman, T. (2007). Parenting and the psychological development of a representative sample of triplets conceived by assisted reproduction. Human Reproduction (Oxford, England), 22(11), 2896-2902. doi: 10.1093/humrep/dem260.

Golombok, S., Perry, B., Burston, A., Murray, C., Mooney-Somers, J., Stevens, M., et al. (2003). Children with lesbian parents: a community study. Developmental Psychology, 39(1), 20-33.

Golombok, S., Tasker, F., & Murray, C. (1997). Children Raised in Fatherless Families from Infancy - Family Relationships and the Socioemotional Development of Children of Lesbian and Single Heterosexual Mothers. Journal of Child Psychology and Psychiatry and Allied Disciplines, 38(7), 783-791.

Greer, A. J., Gulotta, C. S., Masler, E. A., & Laud, R. B. (2008). Caregiver Stress and Outcomes of Children with Pediatric Feeding Disorders Treated in an Intensive Interdisciplinary Program. J. Pediatr. Psychol., 33(6), 612-620. doi: 10.1093/jpepsy/jsm116.

Grietens, H., Haene, L., & Uyttebroek, K. (2007). Cross-cultural Validation of the Child Abuse Potential Inventory in Belgium (Flanders): Relations with Demographic Characteristics and Parenting Problems. Journal of Family Violence, 22(4), 223-229. doi: 10.1007/s10896-007-9074-2.

Gurian, E. A., Kinnamon, D. D., Henry, J. J., & Waisbren, S. E. (2006). Expanded newborn screening for biochemical disorders: the effect of a false-positive result. Pediatrics, 117(6), 1915-1921.

Halpern, L. F., Brand, K. L., & Malone, A. F. (2001). Parenting stress in mothers of very-low-birth-weight (VLBW) and full-term infants: a function of infant behavioral characteristics and child-rearing attitudes. Journal of Pediatric Psychology, 26(2), 93-104.

Halterman, J. S., Borrelli, B., Tremblay, P., Conn, K. M., Fagnano, M., Montes, G., et al. (2008). Screening for Environmental Tobacco Smoke Exposure Among Inner-City Children With Asthma. PEDIATRICS, 122(6), 1277-1283. doi: 10.1542/peds.2008-0104.

Harnett, P. H., & Dawe, S. (2008). Reducing Child Abuse Potential in Families Identified by Social Services: Implications for Assessment and Treatment. Brief Treatment and Crisis Intervention, 8(3), 226-235.

Harvey, E. (1998). Parental Employment and Conduct Problems Among Children with Attention-Deficit/Hyperactivity Disorder - An Examination of Child-Care Workload and Parenting Well-Being as Mediating Variables. Journal of Social and Clinical Psychology, 17(4), 476-490.

Harwood, M. D., & Eyberg, S. M. (2004). Therapist Verbal Behavior Early in Treatment: Relation to Successful Completion of Parent-Child Interaction Therapy. Journal of Clinical Child & Adolescent Psychology, 33(3), 601-612. doi: Article.

Haskett, M. E., Ahern, L. S., Ward, C. S., & Allaire, J. C. (2006). Factor structure and validity of the Parenting Stress Index -Short Form. Journal of Clinical Child & Adolescent Psychology, 35(2), 302-312.

Haskett, M., Scott, S., Willoughby, M., Ahern, L., & Nears, K. (2006). The Parent Opinion Questionnaire and Child Vignettes for Use with Abusive Parents: Assessment of Psychometric Properties. Journal of Family Violence, 21(2), 137-151. doi: 10.1007/s10896-005-9010-2.

Hassall, R., Rose, J., & McDonald, J. (2005). Parenting stress in mothers of children with an intellectual disability: the effects of parental cognitions in relation to child characteristics and family support. Journal of Intellectual Disability Research, 49(6), 405-418.

Hawley, C. A., Ward, A. B., Magnay, A. R., & Long, J. (2003). Parental stress and burden following traumatic brain injury amongst children and adolescents. Brain Injury, 17(1), 1-23.

Hill, C., & Rose, J. (2009). Parenting stress in mothers of adults with an intellectual disability: parental cognitions in relation to child characteristics and family support. Journal of Intellectual Disability Research: JIDR. doi: 10.1111/j.1365-2788.2009.01207.x

Hintermair, M. (2006). Parental Resources, Parental Stress, and Socioemotional Development of Deaf and Hard of Hearing Children. J. Deaf Stud. Deaf Educ., 11(4), 493-513. doi: 10.1093/deafed/enl005.

Holdsworth, M. J. A. (1999). The relationship between social support, living arrangements, ward status and parenting stress of African-American teenage mothers. Dissertation Abstracts International: Section B: the Sciences & Engineering, 59(7-B).

Holub, C. K., Kershaw, T. S., Ethier, K. A., Lewis, J. B., Milan, S., & Ickovics, J. R. (2007). Prenatal and Parenting Stress on Adolescent Maternal Adjustment: Identifying a High-Risk Subgroup.. Maternal & Child Health Journal, 11(2), 153-159. doi: 10.1007/s10995-006-0159-y.

Horwitz, S. M., Briggs-Gowan, M. J., Storfer-Isser, A., & Carter, A. S. (2009). Persistence of Maternal Depressive Symptoms throughout the Early Years of Childhood. Journal of Women's Health, 18(5), 637-645. doi: 10.1089/jwh.2008.1229.

Huebner, C. E. (2002). Evaluation of a clinic-based parent education program to reduce the risk of infant and toddler maltreatment. Public Health Nursing, 19(5), 377–389.

Hung, J. W., Wu, Y., & Yeh, C. (2004). Comparing stress levels of parents of children with cancer and parents of children with physical disabilities. Psycho-Oncology, 13(12), 898-903.

Hutchings, J., Bywater, T., Daley, D., Gardner, F., Whitaker, C., Jones, K.,... Edwards, R. T. (2007). Parenting intervention in Sure Start services for children at risk of developing conduct disorder: pragmatic randomized controlled trial. British Medical Journal, doi:10.1136/bmj.39126.620799.55 (published 9 March 2007)

Hutchings, J., Lane, E., & Kelly, J. (2004). Comparison of two treatments for children with severely disruptive behaviors: A four-year follow-up. Behavioural and Cognitive Psychotherapy, 32(1), 15-30. doi: 10.1017/S1352465804001018.

Huth-Bocks, A., & Hughes, H. (2008). Parenting Stress, Parenting Behavior, and Children's Adjustment in Families Experiencing Intimate Partner Violence. Journal of Family Violence, 23(4), 243-251. doi: 10.1007/s10896-007-9148-1.

Ispa, J. M., Fine, M. A., Halgunseth, L. C., Harper, S., Robinson, J., Boyce, L., et al. (2004). Maternal Intrusiveness, Maternal Warmth, and Mother–Toddler Relationship Outcomes: Variations Across Low-Income Ethnic and Acculturation Groups. Child Development, 75(6), 1613-1631. doi: 10.1111/j.1467-8624.2004.00806.x.

Janssens, K. A., Oldehinkel, A. J., & Rosmalen, J. G. (2009). Parental overprotection predicts the development of functional somatic symptoms in young adolescents. The Journal of Pediatrics, 154(6), 918-923.

Kazak, A. E., & Barakat, L. P. (1997). Brief Report - Parenting Stress and Quality-of-Life During Treatment for Childhood Leukemia Predicts Child and Parent Adjustment After Treatment Ends. Journal of Pediatric Psychology, 22(5), 749-758.

Kazak, A. E., Penati, B., Boyer, B. A., Himelstein, B., Brophy, P., Waibel, M. K., Blackall, G. F., Daller, R., & Johnson, K. (1996). A Randomized Controlled Prospective Outcome Study of a Psychological and Pharmacological Intervention Protocol for Procedural Distress in Pediatric Leukemia. Journal of Pediatric Psychology, 21(5), 615-631.

Kazak, A. E., Penati, B., Waibel, M. K., & Blackall, G. F. (1996). The Perception of Procedures Questionnaire: psychometric properties of a brief parent report measure of procedural distress. Journal of Pediatric Psychology, 21(2), 195-207.

Keeley, M., & Wiens, B. (2008). Family Influences on Treatment Refusal in School-linked Mental Health Services. Journal of Child & Family Studies, 17(1), 109-126. doi: 10.1007/s10826-007-9141-6.

Kelley, S. J. (1998). Stress and coping behaviors of substance-abusing mothers. Journal of the Society of Pediatric Nurses, 3(3), 103-10.

Kinnunen, U., Geurts, S., & Mauno, S. (2004). Work-to-family conflict and its relationship with satisfaction and well-being: a one-year longitudinal study on gender differences. Work & Stress, 18(1), 1-22. doi: 10.1080/02678370410001682005.

Kuhn, J. C., & Carter, A. S. (2006). Maternal self-efficacy and associated parenting cognitions among mothers of children with autism. American Journal of Orthopsychiatry, 76(4), 564–575.

Larson, N. C. (2004). Parenting stress among adolescent mothers in the transition to adulthood. Child & Adolescent Social Work Journal, 21(5), 457-476.

Lauth, G. W., Otte, T., & Heubeck, B. G. (2009). Effectiveness of a competence training programme for parents of socially disruptive children. Emotional & Behavioural Difficulties, 14(2), 117-126.

Lavigne, J. V., LeBailly, S. A., Gouze, K. R., Cicchetti, C., Jessup, B. W., Arend, R., et al. (2008). Predictor and Moderator Effects in the Treatment of Oppositional Defiant Disorder in Pediatric Primary Care. Journal of Pediatric Psychology, 33(5), 462.

Lecavalier, L., Leone, S., & Wiltz, J. (2006). The impact of behaviour problems on caregiver stress in young people with autism spectrum disorders. Journal of Intellectual Disability Research, 50 (3), 172-183.

Lederman, C., & Osofsky, J. (2004). Infant mental health interventions in juvenile court. Psychology, Public Policy, and Law, 10(1-2), 162-177. doi: 10.1037/1076-8971.10.1.162.

Lee, M., Chen, Y., Wang, H., & Chen, D. (2007). Parenting stress and related factors in parents of children with Tourette Syndrome. Journal of Nursing Research, 15(3), 165-174.

Lees, D. G., & Ronan, K. R. (2008). Engagement and Effectiveness of Parent Management Training (Incredible Years) for Solo High-Risk Mothers: A Multiple Baseline Evaluation. Behaviour Change, 25(2), 109-128. doi: 10.1375/bech.25.2.109.

Leung, S. S. L., Leung, C., & Chan, R. (2007). Perceived child behaviour problems, parenting stress, and marital satisfaction: comparison of new arrival and local parents of preschool children in Hong Kong. Hong Kong Medical Journal = Xianggang Yi Xue Za Zhi / Hong Kong Academy of Medicine, 13(5), 364-371.

Limperopoulos, C., Robertson, R. L., Estroff, J. A., Barnewolt, C., Levine, D., Bassan, H., du Plessis, A. J. (2006). Diagnosis of inferior vermian hypoplasia by fetal magnetic resonance imaging: potential pitfalls and neurodevelopmental outcome. American Journal of Obstetrics and Gynecology, 194(4), 1070-1076. doi: 10.1016/j.ajog.2005.10.191

Lipman, E. L., Boyle, M. H., Cunningham, C., Kenny, M., Sniderman, C., Duku, E., et al. (2006). Testing effectiveness of a community-based aggression management program for children 7 to 11 years old and their families. Journal of the American Academy of Child and Adolescent Psychiatry, 45(9), 1085-1093. doi: 10.1097/01.chi.0000228132.64579.73.

Luthar, S. S., & Sexton, C. C. (2007). Maternal drug abuse versus maternal depression: Vulnerability and resilience among school-age and adolescent offspring. Development and Psychopathology, 19(01). doi: 10.1017/S0954579407070113.

Macias, M. M., Saylor, C. F., Haire, K. B., & Bell, N. L. (2007). Predictors of paternal versus maternal stress in families of children with neural tube defects. Children's Health Care, 36(2), 99-115.

Mackintosh, V. H., Myers, B. J., & Kennon, S. S. (2006). Children of Incarcerated Mothers and Their Caregivers: Factors Affecting the Quality of Their Relationship. Journal of Child & Family Studies, 15(5), 579-594. doi: 10.1007/s10826-006-9030-4.

Maclas, M. M., Roberts, K. M., Saylor, C. F., & Fussell, J. J. (2006). Toileting concerns, parenting stress, and behavior problems in children with special health care needs. Clinical Pediatrics, 45(5), 415-422.

Majnemer, A., Limperopoulos, C., Shevell, M., Rohlicek, C., Rosenblatt, B. & Tchervenkov, C. (2006). Health and well-being of children with congenital cardiac malformations, and their families, following open-heart surgery. Cardiology in the Young, 16(2), 157-164.

Mak, W. W. S., Ho, A .H. Y., & Law, R. W. (2007). Sense of coherence, parenting attitudes and stress among mothers of children with autism in Hong Kong. Journal of Applied Research in Intellectual Disabilities, 20(2), 157-167.

Mäntymaa, M., Puura, K., Luoma, I., Salmelin, R. K., & Tamminen, T. (2006). Mother's early perception of her infant's difficult temperament, parenting stress and early mother-infant interaction. Nordic Journal of Psychiatry, 60(5), 379-386.

Margalit, M., & Kleitman, T. (2006). Mothers' stress, resilience and early intervention. European Journal of Special Needs Education, 21(3), 269-283.

Marhefka, S. L., Tepper, V. J., Brown, J. L., & Farley, J. J. (2006). Caregiver psychosocial characteristics and children's adherence to antiretroviral therapy. AIDS Patient Care & STDs, 20(6), 429-437.

McAuley, C., McCurry, N., Knapp, M., Beecham, J., & Sleed, M. (2006). Young families under stress: assessing maternal and child well-being using a mixed-methods approach. Child & Family Social Work, 11(1), 43-54. doi: 10.1111/j.1365-2206.2006.00390.x.

McCabe, K., & Yeh, M. (2009). Parent–Child Interaction Therapy for Mexican Americans: A Randomized Clinical Trial. Journal of Clinical Child & Adolescent Psychology, 38(5), 753. doi: 10.1080/15374410903103544

McCue Horwitz, S., Briggs-Gowan, M. J., Storfer-Isser, A., & Carter, A. S. (2007). Prevalence, Correlates, and Persistence of Maternal Depression. Journal of Women's Health, 16(5), 678-691. doi: 10.1089/jwh.2006.0185.

McGlone, K., Santos, L., Kazama, L., Fong, R., & Mueller, C. (2002). Psychological Stress in Adoptive Parents of Special-Needs Children. Child Welfare, 81(2), 151. doi: Article.

McKelvey, L. M., Whiteside-Mansell, L., Faldowski, R. A., Shears, J., Ayoub, C., & Hart, A. D. (2009). Validity of the short form of the parenting stress index for fathers of toddlers. Journal of Child and Family Studies, 18(1), 102-111.

McPherson, A., Lewis, K., Lynn, A., Haskett, M., & Behrend, T. (2009). Predictors of Parenting Stress for Abusive and Nonabusive Mothers. Journal of Child and Family Studies, 18(1), 61-69. doi: 10.1007/s10826-008-9207-0.

Meijssen, D., Wolf, M., Koldewijn, K., van Wassenaer, A., Kok, J., & van Baar, A. (2010). Parenting stress in mothers after very preterm birth and the effect of the Infant Behavioural Assessment and Intervention Program. Child: care, health and development, 37, 195-202.

Mert, E., Hallioglu, O., & Ankarali Camdeviren, H. (2008). Turkish Version of the Parenting Stress Index Short Form: A Psychometric Study. Turkiye Klinikleri J Med Sci, 28, 291–296.

Miller, A. C., Cate, I. M. P., & Johann-Murphy, M. (2001). When Chronic Disability Meets Acute Stress: Psychological and Functional Changes. Developmental Medicine & Child Neurology, 43(3), 214-216.

Miller-Loncar, C., Bigsby, R., High, P., Wallach, M., & Lester, B. (2004). Infant colic and feeding difficulties. Archives of Disease in Childhood, 89(10), 908.

Mills-Koonce, W. R., Propper, C. B., Gariepy, J., Blair, C., Garrett-Peters, P., & Cox, M. J. (2007). Bidirectional genetic and environmental influences on mother and child behavior: the family system as the unit of analyses. Development and Psychopathology, 19(4), 1073-1087. doi: 10.1017/S0954579407000545.

Mitchell, M. D., Hargrove, G. L., Collins, M. H., Thompson, M. P., Reddick, T. L., & Kaslow, N. J. (2006). Coping variables that mediate the relation between intimate partner violence and mental health outcomes among low-income, African American women. Journal of Clinical Psychology, 62(12), 1503-1520. doi: 10.1002/jclp.20305.

Morrell, C. J., Slade, P., Warner, R., Paley, G., Dixon, S., Walters, S. J., … Nicholl, J. (2009). Clinical effectiveness of health visitor training in psychologically informed approaches for depression in postnatal women: pragmatic cluster randomized trial in primary care. British Medical Journal, 338:a3045.

Mowbray, C. T., Bybee, D., Hollingsworth, L., Goodkind, S., & Oyserman, D. (2005). Living Arrangements and Social Support: Effects on the Well-Being of Mothers with Mental Illness. Social Work Research, 29(1), 41. doi: Article.

Mowbray, C., Oyserman, D., Bybee, D., & MacFarlane, P. (2002). Parenting of mothers with a serious mental illness: Differential effects of diagnosis, clinical history, and other mental health variables. Social Work Research, 26(4), 225. doi: Article.

Mullins, L. L., Wolfe-Christensen, C., Hoff Pai, A. L., Carpentier, M. Y., Gillaspy, S., Cheek, J., & Page, M. (2007). The relationship of parental overprotection, perceived child vulnerability, and parenting stress to uncertainty in youth with chronic illness. Journal of Pediatric Psychology, 32(8),973-982.

Murray, C., & Golombok, S. (2005). Solo mothers and their donor insemination infants: follow-up at age 2 years. Human Reproduction (Oxford, England), 20(6), 1655-1660. doi: 10.1093/humrep/deh823.

Musil, C. M. (1998). Health, stress, coping, and social support in grandmother caregivers. Health Care for Women International, 19(5), 441-55.

Naik-Polan, A. T., & Budd, K.S. (2008). Stimulus generalization of parenting skills during parent-child interaction therapy. Journal of Early and Intensive Behavior Intervention, 5(3), 71-92.

Nekkebroeck, J., Bonduelle, M., & Ponjaert-Kristoffersen, I. (2008). Maternal disclosure attitudes and practices of ICSI/IVF conception vis-à-vis a 5-year-old child. Journal of Reproductive & Infant Psychology, 26(1), 44-56. doi: 10.1080/02646830701813343.

Nekkebroeck, J., Bonduelle, M., Desmyttere, S., Van den Broeck, W., & Ponjaert-Kristoffersen, I. (2008). Socio-emotional and language development of 2-year-old children born after PGD/PGS, and parental well-being. Hum. Reprod., den179. doi: 10.1093/humrep/den179.

Nelson, J. R., Stage, S., Duppong-Hurley, K., Synhorst, L., & Epstein, M. H. (2007). Risk Factors Predictive of the Problem Behavior of Children At Risk for Emotional and Behavioral Disorders. Exceptional Children, 73(3), 367-379. doi: Article.

Nereo, N. E., Fee, R. J., & Hinton, V. J. (2003). Parental stress in mothers of boys with Duchenne muscular dystrophy. Journal of Pediatric Psychology, 28(7), 473-484.

New, M., Razzino, B., Lewin, A., Schlumpf, K., & Joseph, J. (2002). Mental Health Service Use in a Community Head Start Population. Arch Pediatr Adolesc Med, 156(7), 721-727. doi: 10.1001/archpedi.156.7.721.

Newman, L. K., Stevenson, C. S., Bergman, L. R., & Boyce, P. (2007). Borderline personality disorder, mother-infant interaction and parenting perceptions: preliminary findings. Australian & New Zealand Journal of Psychiatry, 41(7), 598-605. doi: 10.1080/00048670701392833.

Nicholson, B., Anderson, M., Fox, R., & Brenner, V. (2002). One Family at a Time: a prevention program for at-risk parents. Journal of Counseling & Development, 80(3), 362-371.

Nitz, K., Ketterlinus, R. D., & Brandt, L. J. (1995). The role of stress, social support, and family environment in adolescent mothers’ parenting. Journal of Adolescent Research, 10(3), 358-382.

Noel, M., Peterson, C., & Jesso, B. (2008). The relationship of parenting stress and child temperament to language development among economically disadvantaged preschoolers. Journal of Child Language, 35(4), 823-843. doi: 10.1017/S0305000908008805.

Oberlander, T. F., Reebye, P., Misri, S., Papsdorf, M., Kim, J., & Grunau, R. E. (2007). Externalizing and attentional behaviors in children of depressed mothers treated with a

selective serotonin reuptake inhibitor antidepressant during pregnancy. Archives of Pediatrics & Adolescent Medicine, 161(1), 22-29. doi: 10.1001/archpedi.161.1.22.

Oelofsen, N., & Richardson, P. (2006). Sense of coherence and parenting stress in mothers and fathers of preschool children with developmental disability. Journal of Intellectual & Developmental Disability, 31(1), 1-12. doi: 10.1080/13668250500349367.

O'Neil, M. E., Palisano, R. J., & Westcott, S. L. (2001). Relationship of therapists' attitudes, children's motor ability, and parenting stress to mothers' perceptions of therapists' behaviors during early intervention. Physical Therapy, 81(8), 1412-1424.

Oord, S., Prins, P., Oosterlaan, J., & Emmelkamp, P. (2007). Does brief, clinically based, intensive multimodal behavior therapy enhance the effects of methylphenidate in children with ADHD? European Child & Adolescent Psychiatry, 16(1), 48-57. doi: 10.1007/s00787-006-0574-z.

Oyserman, D., Bybee, D., Mowbray, C. T., & MacFarlane, P. (2002). Positive Parenting among African American Mothers with a Serious Illness. Journal of Marriage and Family, 64(1), 65-77.

Pan, B. A., Rowe, M. L., Singer, J. D., & Snow, C. E. (2005). Maternal correlates of growth in toddler vocabulary production in low-income families. Child Development, 76(4), 763-782. doi: 10.1111/j.1467-8624.2005.00876.x.

Paradise, J. L., Campbell, T. F., Dollaghan, C. A., Feldman, H. M., Bernard, B. S., Colborn, D. K., et al. (2005). Developmental outcomes after early or delayed insertion of tympanostomy tubes. The New England Journal of Medicine, 353(6), 576.

Paradise, J. L., Dollaghan, C. A., Campbell, T. F., Feldman, H. M., Bernard, B. S., Colborn, D. K., …Smith, C. G. (2003). Otitis media and tympanostomy tube insertion during the first three years of life: developmental outcomes at the age of four years. Pediatrics, 112(2), 265-277.

Paradise, J. L., Feldman, H. M., Campbell, T. F., Dollaghan, C. A., Colborn, D. K., Bernard, B. S., et al. (2001). Effect of early or delayed insertion of tympanostomy tubes for persistent otitis media on developmental outcomes at the age of three years. The New England Journal of Medicine, 344(16), 1179-1187.

Paradise, J. L., Feldman, H. M., Colborn, D. K., Campbell, T. F., Dollaghan, C. A., Rockette, H. E., Janosky, J. E., Kurs-Lasky, M., Bernard, B. S., & Smith, C. G. (1999). Parental stress and parent-rated child behavior in relation to otitis media in the first three years of life. Pediatrics, 104(6), 1264-73.

Pemberton, J. R., & Borrego, J. (2005). The relationship between treatment acceptability and familism. International Journal of Behavioral Consultation and Therapy, 1(4), 329-337.

Phillips, J., Morgan, S., Cawthorne, K., & Barnett, B. (2008). Pilot evaluation of parent-child interaction therapy delivered in an Australian community early childhood clinic setting. Australian & New Zealand Journal of Psychiatry, 42(8), 712-719. doi: 10.1080/00048670802206320.

Plant, K., Byrne, L., Barkla, J., McLean, D., Hearle, J., & McGrath, J. (2002). Parents with Psychosis: A Pilot Study Examining Self-Report Measures Related to Family Functioning. Australian e-Journal for the Advancement of Mental Health, 1(1).

Ponjaert-Kristoffersen, I., Tjus, T., Nekkebroeck, J., Squires, J., Verté, D., Heimann, M., ... Wennerholm, U. B. (2004). Psychological follow-up study of 5-year-old ICSI children. Human Reproduction, 19(12), 2791-2797.

Potterton, J., Stewart, A., & Cooper, P. (2007). Parenting stress of caregivers of young children who are HIV positive. African Journal of Psychiatry, 10(4), 210-214.

Putnick, D. L., Bornstein, M. H., Hendricks, C., Painter, K. M., Suwalsky, J. T. D., & Collins, W. A. (2008). Parenting stress, perceived parenting behaviors, and adolescent self-concept in European American families. Journal of Family Psychology: JFP: Journal of the Division of Family Psychology of the American Psychological Association (Division 43), 22(5), 752-762. doi: 10.1037/a0013177.

Qin, X., Tang, C., Zhu, S., Liang, Y., & Zou, X. (2009). Parenting stress and related factors in mothers of children with autism. Chinese Mental Health Journal, 23(9), 629-633.

Quinn, M., Carr, A., Carroll, L., & O Sullivan, D. (2006). An evaluation of the Parents Plus Programme for pre-school children with conduct problems: A comparison of those with and without developmental disabilities. Irish Journal of Psychology, 27(3/4), 168-182.

Quinn, M., Carr, A., Carroll, L., & O'Sullivan, D. (2007). Parents Plus Programme 1: Evaluation of its effectivesness for pre-school children with developmental disabilities and behavioral problems. Journal of Applied Research in Intellectual Disabilities, 20(4), 345-359.

Radcliffe, J., Bennett, D., Kazak, A. E., Foley, B., & Phillips, P. C. (1996). Adjustment in childhood brain tumor survival: child, mother, and teacher report. Journal of Pediatric Psychology, 21(4), 529-39.

Ratner, H. H., Chiodo, L., Covington, C., Sokol, R. J., Ager, J., & Delaney, V. (2006). Violence Exposure, IQ, Academic Performance, and Children's Perception of Safety: Evidence of Protective Effects. Merrill-Palmer Quarterly, 52(2), 264-287. doi: Article.

Razzino, B. E., New, M., Lewin, A., & Joseph, J. (2004). Need for and use of mental health services among parents of children in the head start program. Psychiatric Services (Washington, D.C.), 55(5), 583-586.

Reissland, N., Hopkins, B., Helms, P., & Williams, B. (2009). Maternal stress and depression and the lateralisation of infant cradling. Journal of Child Psychology and Psychiatry, and Allied Disciplines, 50(3), 263-269. doi: 10.1111/j.1469-7610.2007.01791.x.

Reitman, D., Currier, R. O., & Stickle, T. R. (2002). A critical evaluation of the Parenting Stress Index-Short Form (PSI-SF) in a head start population. Journal of Clinical Child & Adolescent Psychology, 31(3), 384-392.

Rholes, W. S., Simpson, J. A., & Friedman, M. (2006). Avoidant attachment and the experience of parenting. Personality and Social Psychology Bulletin, 32(3), 275-285. doi: 10.1177/0146167205280910.

Richman, D., Belmont, J., Kim, M., Slavin, C., & Hayner, A. (2009). Parenting stress in families of children with Cornelia de Lange Syndrome and Down Syndrome, Journal of Developmental and Physical Disabilities, 21, 537-553.

Ritchie, K. L., & Holden, G. W. (1998). Parenting stress in low income battered and community women: Effects on parenting behavior. Early Education and Development, 9(1), 98-112.

Roddenberry, A., & Renk, K. (2008). Quality of Life in Pediatric Cancer Patients: The Relationships Among Parents' Characteristics, Children's Characteristics, and Informant Concordance. Journal of Child & Family Studies, 17(3), 402-426. doi: 10.1007/s10826-007-9155-0.

Rogers, M., Wiener, J., Marton, I., & Tannock, R. (2009). Supportive and Controlling Parental Involvement as Predictors of Children's Academic Achievement: Relations to Children's ADHD Symptoms and Parenting Stress. School Mental Health, 1(2), 89-102. doi: 10.1007/s12310-009-9010-0.

Sadoski, C. M. (1999). Family-school partnerships and the efficacy of parent support groups. Dissertation Abstracts International, 59(7-A).

Schaeffer, C. M., Alexander, P. C., Bethke, K., & Kretz, L. S. (2005). Predictors of Child Abuse Potential Among Military Parents: Comparing Mothers and Fathers. Journal of Family Violence, 20(2), 123-129. doi: 10.1007/s10896-005-3175-6.

Schuck, L. A. (1998). The relationship between family rituals and social skills in preschoolers from low-income families. Dissertation Abstracts International: Section B: the Sciences & Engineering, 58(12-B).

Scott, K., & Crooks, C. V. (2007). Preliminary Evaluation of an Intervention Program for Maltreating Fathers. Brief Treatment and Crisis Intervention, 7(3), 224-238. doi: 10.1093/brief-treatment/mhm007.

Selkirk, R., McLaren, S., Ollerenshaw, A., McLachlan, A. J., & Moten, J. (2006). The longitudinal effects of midwife-led postnatal debriefing on the psychological health of mothers. Journal of Reproductive & Infant Psychology, 24(2), 133-147. doi: 10.1080/02646830600643916.

Sheeran, T., Marvin, R. S., & Pianta, R. C. (1997). Mothers' resolution of their child's diagnosis and self-reported measures of parenting stress, marital relations, and social support. Journal of Pediatric Psychology, 22(2), 197-212.

Shin, J., Nhan, N. V., Crittenden, K. S., Hong, H. T. D., Flory, M., & Ladinsky, J. (2006). Parenting stress of mothers and fathers of young children with cognitive delays in Vietnam. Journal of Intellectual Disability Research, 50(10), 748-760. doi: 10.1111/j.1365-2788.2006.00840.x.

Shuman, A. L. (1998). Facilitating treatment utilization at a child guidance clinic: The effect of preparing parents for child psychotherapy. Dissertation Abstracts International: Section B: the Sciences & Engineering, 59(5-B), 1998.

Silovsky, J. F., Niec, L., Bard, D., & Hecht, D. B. (2007). Treatment for Preschool Children With Interpersonal Sexual Behavior Problems: A Pilot Study. Journal of Clinical Child & Adolescent Psychology, 36(3), 378-391. doi: 10.1080/15374410701444330.

Small, F., Alderdice, F., McCusker, C., Stevenson, M., & Stewart, M. (2005). A prospective cohort study comparing hospital admission for gastro-enteritis with home management. Child: Care, Health & Development, 31(5), 555-562. doi: 10.1111/j.1365-2214.2005.00550.x.

Smith, T. B., Oliver, M. N., & Innocenti, M. S. (2001). Parenting stress in families of children with disabilities. American Journal of Orthopsychiatry, 71(2), 257-261.

Soliday, E., & Lande, M. B. (2002). Family structure and the course of steroid-sensitive nephrotic syndrome. Pediatric Nephrology, 17(1), 41. doi: Article.

Soliday, E., McCluskey-Fawcett, K., & O'Brien, M. (1999). Postpartum affect and depressive symptoms in mothers and fathers. American Journal of Orthopsychiatry, 69(1), 30-38.

Solomon, M., Ono, M., Timmer, S., & Goodlin-Jones, B. (2008). The Effectiveness of Parent–Child Interaction Therapy for Families of Children on the Autism Spectrum.. Journal of Autism & Developmental Disorders, 38(9), 1767-1776. doi: 10.1007/s10803-008-0567-5.

Sperry, L. A., & Symons, F. J. (2003). Maternal judgments of intentionality in young children with autism: the effects of diagnostic information and stereotyped behavior. Journal of Autism and Developmental Disorders, 33(3), 281–287.

Steele, M., Weiss, M., Swanson, J., Wang, J., Prinzo, R. S., & Binder, C. E. (2006). A randomized, controlled effectiveness trial of OROS-methylphenidate compared to usual care with immediate-release methylphenidate in attention deficit-hyperactivity disorder. Can J Clin Pharmacol, 13(1), e50–e62.

Streisnad, R., Braniecki, S., Tercyak, K. P., & Kazak, A. E. (2001). Childhood illness-related parenting stress: the pediatric inventory for parents. Journal of Pediatric Psychology, 26(3), 155-162.

Suchman, N. E., & Luthar, S. S. (2001). The mediating role of parenting stress in methadone-maintained mothers' parenting. Parenting, Science and Practice, 1(4), 285-315.

Sullivan-Bolyai, S., Deatrick, J., Gruppuso, P., Tamborlane, W., & Grey, M. (2002). Mothers' Experiences Raising Young Children With Type 1 Diabetes. Journal for Specialists in Pediatric Nursing, 7(3), 93. doi: Article.

Surkan, P. J., Zhang, A., Trachtenberg, F., Daniel, D. B., McKinlay, S., & Bellinger, D. C. (2007). Neuropsychological function in children with blood lead levels< 10μg/dL. Neurotoxicology, 28(6), 1170–1177.

Taft, A. J., Small, R., Hegarty, K. L., Lumley, J., Watson, L. F., & Gold, L. (2009). MOSAIC (Mothers' Advocates In the Community): protocol and sample description of a cluster randomised trial of mentor mother support to reduce intimate partner violence among pregnant of recent mothers. BMC Public Health, 9, 1-13. doi: 10.1186/1471-2458-9-159.

Tan, S., & Rey, J. (2005). Depression in the young, parental depression and parenting stress. Australasian Psychiatry, 13(1), 76-79. doi: 10.1111/j.1440-1665.2004.02155.x.

Tandon, S. D., Parillo, K. M., Jenkins, C., & Duggan, A. K. (2005). Formative Evaluation of Home Visitors' Role in Addressing Poor Mental Health, Domestic Violence, and Substance Abuse Among Low-Income Pregnant and Parenting Women.. Maternal & Child Health Journal, 9(3), 273-283. doi: 10.1007/s10995-005-0012-8.

Taylor, C. A., Guterman, N. B., Lee, S. J., & Rathouz, P. J. (2009). Intimate Partner Violence, Maternal Stress, Nativity, and Risk for Maternal Maltreatment of Young Children. American Journal of Public Health, 99(1), 175. doi: 10.2105/AJPH.2007.126722.

Tervo, R. (2010). Attention Problems and Parent-Rated Behavior and Stress in Young Children at Risk for Developmental Delay, Journal of Child Neurology, 25, 1325-1330.

Thome, M. (2003). Severe postpartum distress in Icelandic mothers with difficult infants: a follow-up study on their health care. Scandinavian Journal of Caring Sciences, 17(2), 104-112. doi: 10.1046/j.1471-6712.2003.00110.x.

Thome, M., & Alder, B. (1999). A Telephone Intervention to Reduce Fatigue and Symptom Distress in Mothers with Difficult Infants in the Community. Journal of Advanced Nursing, 29(1), 128-137.

Thome, M., & Skuladottir, A. (2005). Changes in sleep problems, parents distress and impact of sleep problems from infancy to preschool age for referred and unreferred children. Scandinavian Journal of Caring Sciences, 19(2), 86-94. doi: 10.1111/j.1471-6712.2005.00322.x.

Thome, M., & Skuladottir, A. (2005). Evaluating a family-centered intervention for infant sleep problems. Journal of Advanced Nursing, 50(1), 5-11. doi: 10.1111/j.1365-2648.2004.03343.x.

Thunström, M. (2002). Severe sleep problems in infancy associated with subsequent development of attention-deficit/hyperactivity disorder at 5.5 years of age. Acta Paediatrica, 91(5), 584. doi: Article.

Timmer, S. G., Sedlar, G., & Urquiza, A. J. (2004). Challenging children in kin versus nonkin foster care: perceived costs and benefits to caregivers. Child Maltreatment, 9(3), 251-262. doi: 10.1177/1077559504266998.

Tomanik, S., Harris, G. E., & Hawkings, J. (2004). The relationship between behaviours exhibited by children with autism and maternal stress. Journal of Intellectual & Developmental Disability, 29(1), 16-26.

Travis, W. J., & Combs-Orme, T. (2007). Resilient Parenting: Overcoming Poor Parental Bonding. Social Work Research, 31(3), 135. doi: Article.

Trute, B., & Hiebert-Murphy, D. (2002). Family adjustment to childhood developmental disability: A measure of parent appraisal of family impacts. Journal of Pediatric Psychology, 27(3), 271-280.

Trute, B., & Hiebert-Murphy, D. (2005). Predicting family adjustment and parenting stress in childhood disability services using brief assessment tools. Journal of Intellectual & Developmental Disability, 30(4), 217-225. doi: 10.1080/13668250500349441.

Tsang, S. K. M., & Leung, C. (2005). Developing a database for evaluating the effectiveness of parent education and support programs: Results of a pilot study. Illinois Child Welfare Journal, 2(1-2), 77–89.

Unger, D. G., Jones, C. W., Park, E., & Tressell, P. A. (2001). Promoting Involvement Between Low-Income Single Caregivers and Urban Early Intervention Programs. Topics in Early Childhood Special Education, 21(4), 197. doi: Article.

van der Pal, S. M., Maguire, C. M., le Cessie, S., van Zwieten, P., Veen, S., Wit, J., & Walther, F. (2008). Very pre-term infants' behaviour at 1 and 2 years of age and parental stress following basic developmental care. British Journal of Developmental Psychology, 26(1), 103-115.

Van Hiel, A., & De Clercq, B. (2009). Authoritarianism is good for you: Right-wing authoritarianism as a buffering factor for mental distress. European Journal of Personality, 23(1), 33-50. doi: 10.1002/per.702.

Vohr, B. R., Jodoin-Krauzyk, J., Tucker, R., Johnson, M. J., Topol, D., & Ahlgren, M. (2008). Results of newborn screening for hearing loss: effects on the family in the first 2 years of life. Archives of Pediatrics & Adolescent Medicine, 162(3), 205-211.

Waisbren, S. E., Albers, S., Amato, S., Brewster, T. G., Demmer, L., Eaton, R. B., …Levy, H. L. (2003). Effect of expanded newborn screening for biochemical genetic disorders on child outcomes and parental stress. Journal of the American Medical Association, 290(19), 2564-2572.

Waisbren, S. E., Rones, M., Read, C. Y., Marsden, D., & Levy, H. L. (2004). Brief report: Predictors of parenting stress among parents of children with biochemical genetic disorders. Journal of Pediatric Psychology, 29(7), 565-570.

Wake, M., Morton-Allen, E., Poulakis, Z., Hiscock, H., Gallagher, S., & Oberklaid, F. (2006). Prevalence, stability, and outcomes of cry-fuss and sleep problems in the first 2 years of life: prospective community-based study. Pediatrics, 117(3), 836.

Weberling, L. C., Forgays, D. K., Crain-Thoreson, C., & Hyman, I. (2003). Prenatal Child Abuse Risk Assessment: A Preliminary Validation Study. Child Welfare, 82(3), 319. doi: Article.

Weisel, A., Most, T., & Michael, R. (2007). Mothers' Stress and Expectations as a Function of Time Since Child's Cochlear Implantation. J. Deaf Stud. Deaf Educ., 12(1), 55-64. doi: 10.1093/deafed/enl020.

Weiss, K. L., Marvin, R. S., & Pianta, R. C. (1997). Ethnographic detection and description of family strategies for child care: Applications to the study of cerebral palsy. Journal of Pediatric Psychology, 22(2), 263-278.

Wheeler, A., Hatton, D., Reichardt, A., & Bailey, D. (2007). Correlates of maternal behaviours in mothers of children with fragile X syndrome. Journal of Intellectual Disability Research, 51(6), 447-462. doi: 10.1111/j.1365-2788.2006.00896.x.

White, C., & Verduyn, C. (2006). The Children And Parents Service (CAPS): a multi-agency early intervention initiative for young children and their families. Child and Adolescent Mental Health, 11(4), 192. doi: 10.1111/j.1475-3588.2006.00410.x.

White-Koning, M., Arnaud, C., Dickinson, H. O., Thyen, U., Beckung, E., Fauconnier, J.,…Colver, A. (2007). Determinants of child-parent agreement in quality-of-life reports: a European study of children with cerebral palsy. Pediatrics, 120(4), e804-814. doi: 10.1542/peds.2006-3272

Wilkinson, B., Marshall, R., & Curtwright, B. (2008). Impact of Tourette's Disorder on Parent Reported Stress. Journal of Child & Family Studies, 17(4), 582-598. doi: 10.1007/s10826-007-9176-8.

Williford, A. P., Calkins, S. D., & Keane, S. P. (2007). Predicting change in parenting stress across early childhood: Child and maternal factors. Journal of Abnormal Child Psychology, 35(2), 251–263.

Wolf, L. C., Fisman, S., Ellison, D., & Freeman, T. (1998). Effect of Sibling Perception of Differential Parental Treatment in Sibling Dyads with One Disabled-Child. Journal of the American Academy of Child and Adolescent Psychiatry, 37(12), 1317-1325.

Wolfe, R. B., & Hirsch, B. J. (2003). Outcomes of parent education programs based on reevaluation counseling. Journal of Child & Family Studies, 12(1), 61-76.

Wolff, N., Darlington, A., Hunfeld, J., Verhulst, F., Jaddoe, V., Hofman, A., et al. (2009). Determinants of Somatic Complaints in 18-month-old Children: The Generation R Study. J. Pediatr. Psychol.. doi: 10.1093/jpepsy/jsp058

Wong, F., & Poon, A. (2010). Cognitive behavioural group treatment for Chinese parents with children with developmental disabilities in Melbourne, Australia: An efficacy study, Australian and New Zealand Journal of Psychiatry, 44, 742-749.

Wong, V., & Kwan, Q. (2009). Randomized control trial for early intervention for Autism: A pilot study of the Autism 1-2-3 Project. Journal of Autism and Developmental Disorders, 40, 677-688.

Woolfson, L., & Grant, E. (2006). Authoritative parenting and parental stress in parents of pre-school and older children with developmental disabilities. Child: Care, Health & Development, 32(2), 177-184.

Wulffaert, J., Scholte, E. M., Dijkxhoorn, Y. M., Bergman, J. E., van Ravenswaaij-Arts, C. M., & van Berckelaer-Onnes, I. A. (2009). Parenting stress in CHARGE syndrome and the relationship with child characteristics. Journal of Developmental and Physical Disabilities, 21(4), 301-313.

Wulffaert, J., Scholte, E., & van Berckelaer-Onnes, I. (2010). Maternal parenting stress in families with a child with Angelman syndrome or Prader–Willi syndrome, Journal of Intellectual & Developmental Disability, 35, 165-174.

Wysocki, T., & Gavin, L. (2004). Psychometric Properties of a New Measure of Fathers' Involvement in the Management of Pediatric Chronic Diseases. J. Pediatr. Psychol., 29(3), 231-240. doi: 10.1093/jpepsy/jsh024.

Yeh, C., Chen, M., & Chuang, H. (2001). The Chinese version of the Parenting Stress Index: a psychometric study. Acta Paediatrica, 90(12), 1470-1477.

Youngblut, J. M., & Brooten, D. (2006). Pediatric head trauma: parent, parent-child, and family functioning 2 weeks after hospital discharge. Journal of Pediatric Psychology, 31(6), 608-618.

Youngblut, J. M., & Brooten, D. (2008). Mother's mental health, mother-child relationship, and family functioning 3 months after a preschooler's head injury. The Journal of Head Trauma Rehabilitation, 23(2), 92-102. doi: 10.1097/01.HTR.0000314528.85758.30.

Yu, M. S., Norris, J. M., Mitchell, C. M., Butler-Simon, N., Groshek, M., Follansbee, D., Erlich, H., Rewers, M., & Klingensmith, G. J. (1999). Impact on maternal parenting stress of receipt of genetic information regarding risk of diabetes in newborn infants. American Journal of Medical Genetics, 86(3), 219-226.

Zaidman-Zait, A., Mirena, P., Zumbo, B., Wellington, S., Dua, V., & Kalynchuk, K. (2010). An item response theory analysis of the Parenting Stress Index-Short Form with

parents of children with autism spectrum disorders. Journal of Child Psychology and Psychiatry, 51, 1269-1277.

Zelkowitz, P., & Milet, T. H. (2001). The course of postpartum psychiatric disorders in women and their partners. The Journal of Nervous and Mental Disease, 189(9), 575-582.

ANXIETY

Andrews-Casal, M., Johnston, D., Fletcher, J., Mulliken, J. B., Stal, S., & Hecht, J. T. (1998). Cleft lip with or without cleft palate: effect of family history on reproductive planning, surgical timing, and parental stress. Cleft Palate Craniofacial Journal, 35(1), 52-7.

Beebe, S. A., Casey, R., & Pinto-Martin, J. (1993). Association of reported infant crying and maternal parenting stress. Clinical Pediatrics, 32(1), 15-9.

Buist, A. (1998). Childhood Abuse, Parenting and Postpartum Depression. Australian and New Zealand Journal of Psychiatry, 32(4), 479-487.

Buist, A., & Janson, H. (1995). Effect of Exposure to Dothiepin and Northiaden in Breast-Milk on Child-Development. British Journal of Psychiatry, 167(SEP), 370-373.

Campis, L. B., DeMaso, D. R., & Twente, A. W. (1995). The role of maternal factors in the adaptation of children with craniofacial disfigurement. Cleft Palate Craniofacial Journal, 32(1), 55-61.

Cuccaro, M. L., Holmes, G. R., & Wright, H. H. (1993). Behavior problems in preschool children: A pilot study. Psychological Reports, 72(1), 121-122.

Dadds, M., & Roth, J. (2008). Prevention of Anxiety Disorders: Results of a Universal Trial with Young Children. Journal of Child & Family Studies, 17(3), 320-335. doi: 10.1007/s10826-007-9144-3.

Deater-Deckard, K., Scarr, S., McCartney, K., & Eisenberg, M. (1994). Paternal Separation Anxiety - Relationships with Parenting Stress, Child-Rearing Attitudes, and Maternal Anxieties. Psychological Science, 5(6), 341-346.

Douglas, A. R. (2000). Reported anxieties concerning intimate parenting in women sexually abused as children. Child Abuse & Neglect, 24(3), 425-34.

Feldman, R., Granat, A., Pariente, C., Kanety, H., Kuint, J., & Gilboa-Schechtman, E. (2009). Maternal Depression and Anxiety Across the Postpartum Year and Infant Social Engagement, Fear Regulation, and Stress Reactivity. Journal of Amer Academy of Child & Adolescent Psychiatry, 48(9), 919 - 927.

Flory, V. (2004). A Novel Clinical Intervention for Severe Childhood Depression and Anxiety. Clinical Child Psychology and Psychiatry, 9(1), 9-23. doi: 10.1177/1359104504039167.

Golombok, S., Cook, R., Bish, A., & Murray, C. (1995). Families Created by the New Reproductive Technologies - Quality of Parenting and Social and Emotional Development of the Children. Child Development, 66(2), 285-298.

Ha, E. H., Oh, K. J., & Kim, E. J. (1999). Depressive symptoms and family relationship of married women: Focused on parenting stress and marital dissatisfaction. Korean Journal of Clinical Psychology, 18(1), 79-93.

Hatcher, J. W., Powers, L. L., & Richtsmeier, A. J. (1993). Parental Anxiety and Response to Symptoms of Minor Illness in Infants. Journal of Pediatric Psychology, 18(3), 397-408.

Kazak, A. E., & Barakat, L. P. (1997). Brief Report - Parenting Stress and Quality-of-Life During Treatment for Childhood Leukemia Predicts Child and Parent Adjustment After Treatment Ends. Journal of Pediatric Psychology, 22(5), 749-758.

Kelley, M. L., Herzog-Simmer, P. A., & Harris, M. A. (1994). Effects of military-induced separation on the parenting stress and family functioning of deploying mothers. Military Psychology, 6(2), 125-138.

Kobe, F. H., & Hammer, D. (1994). Parenting stress and depression in children with mental retardation and developmental disabilities. Research in Developmental Disabilities, 15(3), 209-21.

LaFiosca, T., & Loyd, B. H. (1986). Defensiveness and the assessment of parental stress and anxiety. Journal of Clinical Child Psychology, 15(3), 254-259.

Luthar, S. S., & Sexton, C. C. (2007). Maternal drug abuse versus maternal depression: Vulnerability and resilience among school-age and adolescent offspring. Development and Psychopathology, 19(01). doi: 10.1017/S0954579407070113.

Manassis, K., Bradley, S., Goldberg, S., Hood, J., & Swinson, R. P. (1994). Attachment in Mothers with Anxiety Disorders and Their Children. Journal of the American Academy of Child and Adolescent Psychiatry, 33(8), 1106-1113.

Manassis, K., Bradley, S., Goldberg, S., Hood, J., & Swinson, R. P. (1995). Behavioral-Inhibition, Attachment and Anxiety in Children of Mothers with Anxiety Disorders. Canadian Journal of Psychiatry, 40(2), 87-92.

Markson, S., & Fiese, B. H. (2000). Family rituals as a protective factor for children with asthma. Journal of Pediatric Psychology, 25(7), 471-80.

Musil, C. M. (1998). Health, stress, coping, and social support in grandmother caregivers. Health Care for Women International, 19(5), 441-55.

Pincus, D. B., Eyberg, S. M., & Choate, M. L. (2005). Adapting Parent-Child Interaction Therapy for Young Children with Separation Anxiety Disorder. Education & Treatment of Children, 28(2), 163. doi: Article.

Planos, R., Zayas, L. H., & Buschrossnagel, N. A. (1997). Mental-Health Factors and Teaching Behaviors Among Low-Income Hispanic Mothers. Families in Society-The Journal of Contemporary Human Services, 78(1), 4-12.

Radcliffe, J., Bennett, D., Kazak, A. E., Foley, B., & Phillips, P. C. (1996). Adjustment in childhood brain tumor survival: child, mother, and teacher report. Journal of Pediatric Psychology, 21(4), 529-39.

Realmuto, G. M., August, G. J., & Egan, E. A. (2004). Testing the goodness-of-fit of a multifaceted preventive intervention for children at risk for conduct disorder. Canadian Journal of Psychiatry. Revue Canadienne De Psychiatrie, 49(11), 743-752.

Victor, A. M., Bernat, D. H., Bernstein, G. A., & Layne, A. E. (2007). Effects of parent and family characteristics on treatment outcome of anxious children. Journal of Anxiety Disorders, 21(6), 835-848. doi: 10.1016/j.janxdis.2006.11.005.

Wells, R. D., & Schwebel, A. I. (1987). Chronically ill children and their mothers: Predictors of resilience and vulnerability to hospitalization and surgical stress. Journal of Developmental & Behavioral Pediatrics, 8(2), 83-89.

Wong, F., & Poon, A. (2010). Cognitive behavioural group treatment for Chinese parents with children with developmental disabilities in Melbourne, Australia: An efficacy study, Australian and New Zealand Journal of Psychiatry, 44, 742-749.

Wysocki, T., Huxtable, K., Linscheid, T. R., & Wayne, W. (1989). Adjustment to diabetes mellitus in preschoolers and their mothers. Diabetes Care, 12(8), 524-9.

At Risk

Adamakos, H., Kathleen, R., G., U. D., & John, P. (1986). Maternal social support as a predictor of mother-child stress and stimulation. Child Abuse & Neglect, 10(4), 463-470.

Allen, K. D., Maguire, K. B., Williams, G. E., & Sanger, W. G. (1996). The effects of infertility on parent-child relationships and adjustment. Children's Health Care, 25(2), 93-105.

Anderson, L. S. (2008). Predictors of Parenting Stress in a Diverse Sample of Parents of Early Adolescents in High-Risk Communities. Nursing Research, 57(5), 340-350. doi: 10.1097/01.NNR.0000313502.92227.87.

Bagley, C., & Mallick, K. (1997). Temperament, CNS Problems and Maternal Stressors - Interactive Predictors of Conduct Disorder in 9-Yr-Olds. Perceptual and Motor Skills, 84(2), 617-618.

Baker, B. L., & Heller, T. L. (1996). Preschool-Children with Externalizing Behaviors - Experience of Fathers and Mothers. Journal of Abnormal Child Psychology, 24(4), 513-532.

Baroni, M. A., Anderson, Y. E., & Mischler, E. (1997). Cystic fibrosis newborn screening: impact of early screening results on parenting stress. Pediatric Nursing, 23(2), 143-51.

Barrera, M. E., & Kitching, K. J. (1991). A 3-year early home intervention follow-up study with low birthweight infants and their parents. Topics in Early Childhood Special Education, 10(4), 14-28.

Bijttebier, P., Vertommen, H., & Florentie, K. (2003). Risk-Taking Behavior as a Mediator of the Relationship between Childrens Temperament and Injury Liability. Psychology and Health, 18, 645-653. doi: 10.1080/0887044031000094831.

Bohr, Y. (2005). Infant mental health programs: Experimenting with innovative models-One center's experience with new program funding. Infant Mental Health Journal, 26(5), 407. doi: 10.1002/imhj.20062.

Bramlett, R. K., Rowell, R. K., & Mandenberg, K. (2000). Predicting first grade achievement from kindergarten screening measures: A comparison of child and family predictors. Research in the Schools, 7(1), 1-9.

Brotman, L. M., Klein, R. G., Kamboukos, D., Brown, E. J., Coard, S. I., & Sosinsky, L. S. (2003). Preventive intervention for urban, low-income preschoolers at familial risk for conduct problems: A randomized pilot study. Journal of Clinical Child and Adolescent Psychology, 32(2), 246–257.

Browne, J. V., & Talmi, A. (2005). Family-based intervention to enhance infant-parent relationships in the neonatal intensive care unit. Journal of Pediatric Psychology, 30(8), 667-677.

Brunette, M. F., Richardson, F., White, L., Bemis, G., & Eelkema, R. E. (2004). Integrated family treatment for parents with severe psychiatric disabilities. Psychiatric Rehabilitation Journal, 28(2), 177-180. doi: Article.

Burchinal, M., Roberts, J. E., Zeisel, S. A., Hennon, E. A., & Hooper, S. (2006). Social risk and protective child, parenting, and child care factors in early elementary school years. Parenting: Science and Practice, 6(1), 79. doi: 10.1207/s15327922par0601_4

Casady, A., Diener, M., Isabella, R., & Wright, C. (2001). Attachment security among families in poverty: maternal, child, and contextual characteristics. Paper presented at the 2001 Biennial Conference of the Society for Research in Child Development, Minneapolis, MN.

Caserta, M. T., O'Connor, T. G., Wyman, P. A., Wang, H., Moynihan, J., Cross, W., et al. (2008). The associations between psychosocial stress and the frequency of illness, and innate and adaptive immune function in children☆. Brain, Behavior, and Immunity, 22(6), 933-940. doi: 10.1016/j.bbi.2008.01.007.

Coleman, P. K., & Karraker, K. H. (1998). Self-Efficacy and Parenting Quality - Findings and Future Applications. Developmental Review, 18(1), 47-85.

Cornish, A. M., McMahon, C. A., Ungerer, J. A., Barnett, B., Kowalenko, N., & Tennant, C. (2006). Maternal depression and the experience of parenting in the second postnatal year. Journal of Reproductive & Infant Psychology, 24(2), 121-132. doi: 10.1080/02646830600644021.

Cowen, P. S., & Reed, D. A. (2002). Effects of respite care for children with developmental disabilities: evaluation of an intervention for at risk families. Public Health Nursing, 19(4), 272-283.

Dadds, M., & Roth, J. (2008). Prevention of Anxiety Disorders: Results of a Universal Trial with Young Children. Journal of Child & Family Studies, 17(3), 320-335. doi: 10.1007/s10826-007-9144-3.

DiLauro, M. D. (2004). Psychosocial Factors Associated with Types of Child Maltreatment. Child Welfare, 83(1), 69. doi: Article.

Dixon, L., Hamilton-Giachritsis, C., Browne, K., & Ostapuik, E. (2007). The Co-occurrence of Child and Intimate Partner Maltreatment in the Family: Characteristics of

the Violent Perpetrators. Journal of Family Violence, 22(8), 675-689. doi: 10.1007/s10896-007-9115-x.

Duggan, A., Caldera, D., Rodriguez, K., Burrell, L., Rohde, C., & Crowne, S. S. (2007). Impact of a statewide home visiting program to prevent child abuse. Child Abuse & Neglect, 31(8), 801-827. doi: 10.1016/j.chiabu.2006.06.011.

Dukewich, T. L., Borkowski, J. G., & Whitman, T. L. (1996). Adolescent Mothers and Child-Abuse Potential - An Evaluation of Risk-Factors. Child Abuse & Neglect, 20(11), 1031-1047.

El-Kamary, S. S., Higman, S. M., Fuddy, L., McFarlane, E., Sia, C., & Duggan, A. K. (2004). Hawaii's healthy start home visiting program: determinants and impact of rapid repeat birth. Pediatrics, 114(3), e317-326. doi: 10.1542/peds.2004-0618.

Feldman, M. A., & Waltonallen, N. (1997). Effects of Maternal Mental-Retardation and Poverty on Intellectual, Academic, and Behavioral Status of School-Age-Children. American Journal on Mental Retardation, 101(4), 352-364.

Flake, E., Davis, B., Johnson, P., & Middleton, L. (2009). The Psychosocial Effects of Deployment on Military Children, Journal of Developmental & Behavioral Pediatrics, 30, 271-278.

Florsheim, P., Moore, D., Zollinger, L., MacDonald, J., & Sumida, E. (1999). The transition to parenthood among adolescent fathers and their partners: Does antisocial behavior predict problems in parenting? Applied Developmental Science, 3(3), 178-191.

Forbes, L. M., Evans, E. M., Moran, G., & Pederson, D. R. (2007). Change in Atypical Maternal Behavior Predicts Change in Attachment Disorganization From 12 to 24 Months in a High-Risk Sample. Child Development, 78(3), 955-971. doi: 10.1111/j.1467-8624.2007.01043.x.

Goldberg, S., Janus, M., Washington, J., Simmons, R. J., Maclusky, I., & Fowler, R. S. (1997). Prediction of Preschool Behavioral-Problems in Healthy and Pediatric Samples. Journal of Developmental and Behavioral Pediatrics, 18(5), 304-313.

Hadadian, A., & Merbler, J. (1996). Mother’s stress: Implications for attachment relationships. Early Child Development & Care, 125, 59-66.

Hall, J. D., & Barnett, D. W. (1991). Classification of risk status in preschool screening: A comparison of alternative measures. Journal of Psychoeducational Assessment, 9(2), 152-159.

Holub, C. K., Kershaw, T. S., Ethier, K. A., Lewis, J. B., Milan, S., & Ickovics, J. R. (2007). Prenatal and Parenting Stress on Adolescent Maternal Adjustment: Identifying a

High-Risk Subgroup.. Maternal & Child Health Journal, 11(2), 153-159. doi: 10.1007/s10995-006-0159-y.

Hutchings, J., Bywater, T., Daley, D., Gardner, F., Whitaker, C., Jones, K.,... Edwards, R. T. (2007). Parenting intervention in Sure Start services for children at risk of developing conduct disorder: pragmatic randomized controlled trial. British Medical Journal, doi:10.1136/bmj.39126.620799.55 (published 9 March 2007)

Kazdin, A. E., Holland, L., Crowley, M., & Breton, S. (1997). Barriers to Treatment Participation Scale - Evaluation and Validation in the Context of Child Outpatient Treatment. Journal of Child Psychology and Psychiatry and Allied Disciplines, 38(8), 1051-1062.

Kluger, M. P., & Aprea, D. M. (1999). Grandparents raising grandchildren: A description of the families and a special pilot program. Journal of Gerontological Social Work, 32(1), 5-17.

LaFiosca, T., & Loyd, B. H. (1986). Defensiveness and the assessment of parental stress and anxiety. Journal of Clinical Child Psychology, 15(3), 254-259.

Lutzker, J. R., Bigelow, K. M., Doctor, R. M., & Kessler, M. L. (1998). Safety, health care, and bonding within an ecobehavioral approach to treating and preventing child abuse and neglect. Journal of Family Violence, 13(2), 163-185.

Manassis, K., Bradley, S., Goldberg, S., Hood, J., & Swinson, R. P. (1995). Behavioral-Inhibition, Attachment and Anxiety in Children of Mothers with Anxiety Disorders. Canadian Journal of Psychiatry, 40(2), 87-92.

Meadow-Orlans, K. P., Smith-Gray, S., & Dyssegaard, B. (1995). Infants who are deaf or hard of hearing, with and without physical/cognitive disabilities. American Annals of the Deaf, 140(3), 279-86.

Meisels, S. J., & Liaw, F. R. (1993). Failure in Grade - Do Retained Students Catch-Up. Journal of Educational Research, 87(2), 69-77.

Milner, J. S., & Crouch, J. L. (1997). Impact and detection of response distortions on parenting measures used to assess risk for child physical abuse. Journal of Personality Assessment, 69(3), 633-50.

Nelson, J. R., Stage, S., Duppong-Hurley, K., Synhorst, L., & Epstein, M. H. (2007). Risk Factors Predictive of the Problem Behavior of Children At Risk for Emotional and Behavioral Disorders. Exceptional Children, 73(3), 367-379. doi: Article.

Nereo, N. E., Fee, R. J., & Hinton, V. J. (2003). Parental stress in mothers of boys with Duchenne muscular dystrophy. Journal of Pediatric Psychology, 28(7), 473-484.

Nievar, M. A, Jacobson, A., & Dier, S. (2008, November). Home visiting for at-risk preschoolers: A successful model for Latino families. Paper presented at the Annual Meeting of the National Council on Family Relations, Little Rock, Arkansas.

Rudo, Z. H., Powell, D. S., & Dunlap, G. (1998). The Effects of Violence in the Home on Childrens Emotional, Behavioral, and Social Functioning - A Review of the Literature. Journal of Emotional and Behavioral Disorders, 6(2), 94-113.

Stoiber, K. C., & Houghton, T. G. (1994). Adolescent mothers' cognitions and behaviors as at-risk indicators. School Psychology Quarterly, 9(4), 295-316.

Stuart, A., Moretz, M., & Yang, E. Y. (2000). An investigation of maternal stress after neonatal hearing screening. American Journal of Audiology, 9(2), 135-41.

Van Hasselt, V. B., Hersen, M., Null, J. A., Ammerman, R. T., Bukstein, O. G., Mcgillivray, J., & Hunter, A. (1993). Drug-Abuse Prevention for High-Risk African-American Children and Their Families - A Review and Model Program. Addictive Behaviors, 18(2), 213-234.

Waldron, A., Tobin, G., & McQuaid, P. (2001). Mental health status of homeless children and their families. Irish Journal of Psychological Medicine, 18(1), 11-15.

Weinfield, N. S., Ogawa, J. R., & Egeland, B. (2002). Predictability of observed mother-child interaction from preschool to middle childhood in a high-risk sample. Child development, 528–543.

Weinstein, P., Domoto, P., Wohlers, K., & Koday, M. (1992). Mexican-American Parents with Children at Risk for Baby Bottle Tooth-Decay - Pilot-Study at a Migrant Farmworkers Clinic. Journal of Dentistry for Children, 59(5), 376-383.

Whipple, E. E. (1999). Reaching Families with Preschoolers at Risk of Physical Child-Abuse - What Works. Families in Society-The Journal of Contemporary Human Services, 80(2), 148-160.

Attachment

Ando, J., Nonaka, K., Ozaki, K., Sato, N., Fujisawa, K. K., Suzuki, K., et al. (2006). The Tokyo Twin Cohort Project: overview and initial findings. Twin Research and Human Genetics: The Official Journal of the International Society for Twin Studies, 9(6), 817-826.

Andreozzi, L., Flanagan, P., Seifer, R., Brunner, S., & Lester, B. (2002). Attachment classifications among 18-month-old children of adolescent mothers. Archives of Pediatrics & Adolescent Medicine, 156(1), 20-26.

Armstrong, K. L., Fraser, J. A., Dadds, M. R., & Morris, J. (2000). Promoting secure attachment, maternal mood and child health in a vulnerable population: a randomized controlled trial. Journal of Paediatrics & Child Health, 36(6), 555-62.

Bigras, M., & Lafreniere, P. J. (1994). Influence of Psychosocial Risk, Marital Conflicts and Parental Stress on the Quality of Mother-Son and Mother-Daughter Interactions. Canadian Journal of Behavioural Science, 26(2), 280-297.

Black, M. M., Hutcheson, J. J., Dubowitz, H., Starr, R. H., & Berensonhoward, J. (1996). The Roots of Competence - Mother-Child Interaction Among Low-Income, Urban, African-American Families. Journal of Applied Developmental Psychology, 17(3), 367-391.

Butteweg Gratton, M. (1999). Case studies in the therapeutic introduction of a transitional object to recast the relational experience of abused children. Dissertation Abstracts International, 59(8-A).

Campbell, S. B. (1997). Behavior Problems in Preschool-Children - Developmental and Family Issues. Advances in Clinical Child Psychology, 19, 1-26.

Carson, D. K., & Schauer, R. W. (1992). Mothers of children with asthma: Perceptions of parenting stress and the mother-child relationship. Psychological Reports, 71(3, Pt 2), 1139-1148.

Casady, A., Diener, M., Isabella, R., & Wright, C. (2001). Attachment security among families in poverty: maternal, child, and contextual characteristics. Paper presented at the 2001 Biennial Conference of the Society for Research in Child Development, Minneapolis, MN.

Chisholm, K. (1998). A 3 Year Follow-Up of Attachment and Indiscriminate Friendliness in Children Adopted from Romanian Orphanages. Child Development, 69(4), 1092-1106.

Chisholm, K., Carter, M. C., Ames, E. W., & Morison, S. J. (1995). Attachment security and indiscriminately friendly behavior in children adopted from Romanian orphanages. Development & Psychopathology, 7(2), 283-294.

Clark, R., Hyde, J. S., Essex, M. J., & Klein, M. H. (1997). Length of Maternity Leave and Quality of Mother-Infant Interactions. Child Development, 68(2), 364-383.

Cole, S. (2005). Infants in foster care: Relational and environmental factors affecting attachment. JOURNAL OF REPRODUCTIVE AND INFANT PSYCHOLOGY, 23, 43-61.

Cole, S. A. (2002). Security of Attachment of Infants in Foster Care. In Proceedings of the... National Symposium on Doctoral Research in Social Work (p. 12). Presented at the National Symposium on Doctoral Research in Social Work, Columbus, OH: Ohio State University.

Crandell, L. E., Fitzgerald, H. E., & Whipple, E. E. (1997). Dyadic Synchrony in Parent-Child Interactions - A Link with Maternal Representations of Attachment Relationships. Infant Mental Health Journal, 18(3), 247-264.

Deater-Deckard, K., Scarr, S., McCartney, K., & Eisenberg, M. (1994). Paternal Separation Anxiety - Relationships with Parenting Stress, Child-Rearing Attitudes, and Maternal Anxieties. Psychological Science, 5(6), 341-346.

Dekovic, M., & Meeus, W. (1997). Peer Relations in Adolescence - Effects of Parenting and Adolescents Self-Concept. Journal of Adolescence, 20(2), 163-176.

Dekovic, M., & Rabotegsaric, Z. (1997). Parental Child-Rearing Practices and Adolescent Peer Relations. Drustvenaistraizivanja, 6(4-5), 427-445.

Edens, J. F., Cavell, T. A., & Hughes, J. N. (1999). The Self-Systems of Aggressive-Children - A Cluster-Analytic Investigation. Journal of Child Psychology and Psychiatry and Allied Disciplines, 40(3), 441-453.

Emery, J., Paquette, D., & Bigras, M. (2008). Factors predicting attachment patterns in infants of adolescent mothers. Journal of Family Studies, 14(1), 65-90. doi: Article.

Endriga, M. C. (1998). Feeding and Attachment in Infants with and Without Orofacial Clefts. Infant Behavior and Development, 21(4), 699-712.

Ethier, L. S., Lacharite, C., & Couture, G. (1995). Childhood adversity, parental stress, and depression of negligent mothers. Child Abuse & Neglect, 19(5), 619-32.

Forbes, L. M., Evans, E. M., Moran, G., & Pederson, D. R. (2007). Change in Atypical Maternal Behavior Predicts Change in Attachment Disorganization From 12 to 24

Months in a High-Risk Sample. Child Development, 78(3), 955-971. doi: 10.1111/j.1467-8624.2007.01043.x.

Frankel, K. K., & Harmon, R. J. (1996). Depressed Mothers - They Dont Always Look as Bad as They Feel. Journal of the American Academy of Child and Adolescent Psychiatry, 35(3), 289-298.

Gelfand, D. M., Teti, D. M., Seiner, S. A., & Jameson, P. B. (1996). Helping Mothers Fight Depression - Evaluation of a Home-Based Intervention Program for Depressed Mothers and Their Infants. Journal of Clinical Child Psychology, 25(4), 406-422.

Goldberg, S., Janus, M., Washington, J., Simmons, R. J., Maclusky, I., & Fowler, R. S. (1997). Prediction of Preschool Behavioral-Problems in Healthy and Pediatric Samples. Journal of Developmental and Behavioral Pediatrics, 18(5), 304-313.

Golombok, S., Tasker, F., & Murray, C. (1997). Children Raised in Fatherless Families from Infancy - Family Relationships and the Socioemotional Development of Children of Lesbian and Single Heterosexual Mothers. Journal of Child Psychology and Psychiatry and Allied Disciplines, 38(7), 783-791.

Hadadian, A., & Merbler, J. (1996). Mother's stress: Implications for attachment relationships. Early Child Development & Care, 125, 59-66.

Hanson, M. J., & Hanline, M. F. (1990). Parenting a child with a disability: A longitudinal study of parental stress and adaptation. Journal of Early Intervention, 14(3), 234-248.

Hauenstein, E. J., Marvin, R. S., Snyder, A. L., & Clarke, W. L. (1989). Stress in parents of children with diabetes mellitus. Diabetes Care, 12(1), 18-23.

Hoppes, K., & Harris, S. L. (1990). Perceptions of child attachment and maternal gratification in mothers of children with Autism and Down's Syndrome. Journal of Clinical Child Psychology, 19, 365-370.

Huth-Bocks, A. C., Levendosky, A. A., Bogat, G. A., & Von Eye, A. (2004). The Impact of Maternal Characteristics and Contextual Variables on Infant–Mother Attachment. Child Development, 75(2), 480-496. doi: 10.1111/j.1467-8624.2004.00688.x.

Janus, M., & Goldberg, S. (1997). Factors Influencing Family Participation in a Longitudinal-Study - Comparison of Pediatric and Healthy Samples. Journal of Pediatric Psychology, 22(2), 245-262.

Jarvis, P. A., & Creasey, G. L. (1991). Parental stress, coping, and attachment in families with an 18-month-old infant. Infant Behavior & Development, 14(4), 383-395.

Kazui, M., Muto, T., & Sonoda, N. (1996). The roles of marital quality and parenting stress in mother-preschooler relationships. (Japanese). Japanese Journal of Developmental Psychology, 7(1), 31-40.

Lafreniere, P. J., & Capuano, F. (1997). Preventive Intervention as Means of Clarifying Direction of Effects in Socialization - Anxious-Withdrawn Preschoolers Case. Development and Psychopathology, 9(3), 551-564.

Lutzker, J. R., Bigelow, K. M., Doctor, R. M., & Kessler, M. L. (1998). Safety, health care, and bonding within an ecobehavioral approach to treating and preventing child abuse and neglect. Journal of Family Violence, 13(2), 163-185.

Macturk, R. H., Meadoworlans, K. P., Koester, L. S., & Spencer, P. E. (1993). Social Support, Motivation, Language, and Interaction - A Longitudinal-Study of Mothers and Deaf Infants. American Annals of the Deaf, 138(1), 19-25.

Mainemer, H., Gilman, L. C., & Ames, E. W. (1998). Parenting stress in families adopting children from Romanian orphanages. Journal of Family Issues, 19(2), 164-180.

Manassis, K., Bradley, S., Goldberg, S., Hood, J., & Swinson, R. P. (1994). Attachment in Mothers with Anxiety Disorders and Their Children. Journal of the American Academy of Child and Adolescent Psychiatry, 33(8), 1106-1113.

Manassis, K., Bradley, S., Goldberg, S., Hood, J., & Swinson, R. P. (1995). Behavioral-Inhibition, Attachment and Anxiety in Children of Mothers with Anxiety Disorders. Canadian Journal of Psychiatry, 40(2), 87-92.

Marcus, R. F., & Kramer, C. (2001). Reactive and Proactive Aggression: Attachment and Social Competence Predictors. Journal of Genetic Psychology, 162(3), 260. doi: Article.

Mattie-Luksic, M., Javornisky, G., & DiMario, F. J. (2000). Assessment of stress in mothers of children with severe breath-holding spells. Pediatrics, 106(1 Pt 1), 1-5.

McCallum, M. S., & McKim, M. K. (1999). Recurrent otitis media and attachment security: A path model. Early Education and Development, 10(4), 517-534.

McCartney, K., Scarr, S., Rocheleau, A., Phillips, D., & Abbottshim, M. (1997). Teacher-Child Interaction and Child-Care Auspices as Predictors of Social Outcomes in Infants, Toddlers, and Preschoolers. Journal of Developmental Psychology, 43(3), 426-450.

McCarty, C., Waterman, J., Burge, D., & Edelstein, S. B. (1999). Experiences, concerns, and service needs of families adopting children with prenatal substance exposure: Summary and recommendations. Child Welfare, 78(5), 561-577.

Milgrom, J., & McCloud, P. (1996). Parenting Stress and Postnatal Depression. Stress Medicine, 12(3), 177-186.

Moran, G., & Pederson, D. R. (1998). Proneness to distress and ambivalent relationships. Infant Behavior & Development, 21(3), 493-503.

Moran, G., Pederson, D. R., Pettit, P., & Krupka, A. (1992). Maternal Sensitivity and Infant Mother Attachment in a Developmentally Delayed Sample. Infant Behavior & Development, 15(4), 427-442.

Nakagawa, M., Teti, D. M., & Lamb, M. E. (1992). An ecological study of child-mother attachments among Japanese sojourners in the United States. Developmental Psychology, 28, 584-592.

Park, U. I. (1995). Patterns of infant-mother attachment and related variables. Korean Journal of Child Studies, 16(1), 113-131.

Phipps, S., & Drotar, D. (1990). Determinants of parenting stress in home apnea monitoring. Journal of Pediatric Psychology, 15(3), 385-399.

Pithers, W. D., Gray, A., Busconi, A., & Houchens, P. (1998). Caregivers of children with sexual behavior problems: Psychological and familial functioning. Child Abuse & Neglect, 22(2), 129-141.

Rholes, W. S., Simpson, J. A., & Friedman, M. (2006). Avoidant attachment and the experience of parenting. Personality and Social Psychology Bulletin, 32(3), 275-285. doi: 10.1177/0146167205280910.

Robson, A. L. (1997). Low-Birth-Weight and Parenting Stress During Early-Childhood. Journal of Pediatric Psychology, 22(3), 297-311.

Singer, L. T., Song, L.-y., Hill, B. P., & Jaffe, A. C. (1990). Stress and depression in mothers of failure-to-thrive children. Journal of Pediatric Psychology, 15(6), 711-720.

Speltz, M. L., Endriga, M. C., Fisher, P. A., & Mason, C. A. (1997). Early Predictors of Attachment in Infants with Cleft-Lip and/or Palate. Child Development, 68(1), 12-25.

Stoiber, K. C., & Houghton, T. G. (1993). The Relationship of Adolescent Mothers Expectations, Knowledge, and Beliefs to Their Young Childrens Coping Behavior. Infant Mental Health Journal, 14(1), 61-79.

Strathearn, L., Fonagy, P., Amico, J., & Montague, P. R. (2009). Adult Attachment Predicts Maternal Brain and Oxytocin Response to Infant Cues. Neuropsychopharmacology, 1, 12.

Stuart, A., Moretz, M., & Yang, E. Y. (2000). An investigation of maternal stress after neonatal hearing screening. American Journal of Audiology, 9(2), 135-41.

Symons, D. K. (1998). Postpartum Employment Patterns, Family-Based Care Arrangements, and the Mother-Infant Relationship at Age-2. Canadian Journal of Behavioral Science, 30(2), 121-131.

Tarabulsy, G. M., Avgoustis, E., Phillips, J., Pederson, D. R., & Moran, G. (1997). Similarities and Differences in Mothers and Observers Descriptions of Attachment Behaviors. International Journal of Behavior Development, 21(3), 599-619.

Teti, D. M., Messinger, D. S., Gelfand, D. M., & Isabella, R. (1995). Maternal Depression and the Quality of Early Attachment - An Examination of Infants, Preschoolers, and Their Mothers. Developmental Psychology, 31(3), 364-376.

Teti, D. M., Nakagawa, M., Das, R., & Wirth, O. (1991). Security of attachment between preschoolers and their mothers: Relations among social interaction, parenting stress, and mother's sorts of the Attachment Q-Set. Developmental Psychology, 27(3), 440-447.

Travis, W. J., & Combs-Orme, T. (2007). Resilient Parenting: Overcoming Poor Parental Bonding. Social Work Research, 31(3), 135. doi: Article.

Volling, B. L., Notaro, P. C., & Larsen, J. J. (1998). Adult Attachment Styles - Relations with Emotional Well-Being, Marriage, and Parenting. Family Relations, 47(4), 355-367.

Willinger, U., Diendorfer-Radner, G., Willnauer, R., Jörgl, G., & Hager, V. (2005). Parenting stress and parental bonding. Behavioral Medicine, 31(2), 63-69.

Wittmer, D., Doll, B., & Strain, P. (1996). Social and Emotional Development in Early-Childhood - The Identification of Competence and Disabilities. Journal of Early Intervention, 20(4), 299-317.

Attention Deficit Hyperactivity Disorder (ADHD)

Ammerman, R. T., & Patz, R. J. (1996). Determinants of Child-Abuse Potential - Contribution of Parent and Child Factors. Journal of Clinical Child Psychology, 25(3), 300-307.

Anastopoulos, A. D., Guevremont, D. C., Shelton, T. L., & DuPaul, G. J. (1992). Parenting stress among families of children with attention deficit hyperactivity disorder. Journal of Abnormal Child Psychology, 20(5), 503-20.

Anastopoulos, A. D., Shelton, T. L., DuPaul, G. J., & Guevremont, D. C. (1993). Parent training for attention-deficit hyperactivity disorder: Its impact on parent functioning. Journal of Abnormal Child Psychology, 21(5).

Baker, B. L., & Heller, T. L. (1996). Preschool-Children with Externalizing Behaviors - Experience of Fathers and Mothers. Journal of Abnormal Child Psychology, 24(4), 513-532.

Baker, D. B. (1994). Parenting stress and ADHD: A comparison of mothers and fathers. Journal of Emotional & Behavioral Disorders, 2(1), 46-50.

Baker, D. B., & Kevin, M. (1995). Parenting stress in parents of children with attention-deficit hyperactivity disorder and parents of children with learning disabilities. Journal of Child & Family Studies, 4(1), 57-68.

Barkley, R. A., & Fischer, M. (1988). Development of a multimethod clinical protocol for assessing stimulant drug response in children with attention deficit disorder. Journal of Clinical Child Psychology, 17, 14-24.

Barkley, R. A., Anastopoulos, A., Guevremont, D. C., & Fletcher, K. E. (1992). Adolescents with attention deficit hyperactivity disorder: Mother-adolescent interactions, family beliefs and conflicts, and maternal psychopathology. Journal of Abnormal Child Psychology, 20(3), 263-288.

Barkley, R. A., Fischer, M., Edelbrock, C., & Smallish, L. (1991). The adolescent outcome of hyperactive children diagnosed by research. Journal of Child Psychology & Psychiatry & Allied Disciplines, 32(2), 233-255.

Barkley, R. A., Guevremont, D. C., Anastopoulos, A. D., & Fletcher, K. E. (1992). A comparison of three family therapy programs for treating family conflicts. Journal of Consulting & Clinical Psychology, 60(3), 450-462.

Barkley, R. A., McMurray, M. B., Edelbrock, C. S., & Robbins, K. (1989). The response of aggressive and nonaggressive ADHD children to two doses of Methylphenidate. Journal of the American Academy of Child & Adolescent Psychiatry, 28(6), 873-881.

Bellinger, D., Leviton, A., Allred, E., & Rabinowitz, M. (1994). Prenatal and Postnatal Lead-Exposure and Behavior Problems in School-Aged Children. Environmental Research, 66(1), 12-30.

Breen, M. J., & Barkley, R. A. (1988). Child psychopathology and parenting stress in girls and boys having attention deficit disorder with hyperactivity. Journal of Pediatric Psychology, 13(2), 265-280.

Byrne, J. M., Dewolfe, N. A., & Bawden, H. N. (1998). Assessment of Attention-Deficit Hyperactivity Disorder in Preschoolers. Child Neuropsychology, 4(1), 49-66.

Campbell, S. B., Pierce, E. W., Moore, G., Marakovitz, S., & Newby, K. (1996). Boys Externalizing Problems at Elementary-School Age - Pathways from Early Behavior Problems, Maternal Control, and Family Stress. Development and Psychopathology, 8(4), 701-719.

Chacko, A., Wymbs, B. T., Flammer, L. M., Pelham, W. E., Walker, K. S., Arnold, F. W., …Herbst, L.(2008). A pilot study of the feasibility and efficacy of the Strategies to Enhance Positive Parenting (STEPP) Program for single mothers of children with ADHD. Journal of Attention Disorders, 12(3), 270-280.

Cohn, E. S., & Cermak, S. A. (1998). Including the Family Perspective in Sensory Integration Outcomes Research. American Journal of Occupational Therapy, 52(7), 540-546.

Danforth, J. S. (1998). The Outcome of Parent Training Using the Behavior Management Flow-Chart with Mothers and Their Children with Oppositional Defiant Disorder and Attention-Deficit Hyperactivity Disorder. Behavior Modification, 22(4), 443-473.

Deater-Deckard, K. (1998). Parenting Stress and Child Adjustment - Some Old Hypotheses and New Questions. Clinical Psychology - Science and Practice, 5(3), 314-332.

Deater-Deckard, K., & Scarr, S. (1996). Parenting Stress Among Dual-Earner Mothers and Fathers - Are There Gender Differences. Journal of Family Psychology, 10(1), 45-59.

Demick, J., & Andreoletti, C. (1995). Some Relations Between Clinical and Environmental Psychology. Environment and Behavior, 27(1), 56-72.

Donenberg, G., & Baker, B. L. (1993). The Impact of Young-Children with Externalizing Behaviors on Their Families. Journal of Abnormal Child Psychology, 21(2), 179-198.

Friars, P., & Mellor, D. (2007). Drop Out from Behavioral Management Training Programs for ADHD: A Prospective Study. Journal of Child & Family Studies, 16(3), 427-441. doi: 10.1007/s10826-006-9096-z.

Greene, R. W., Abidin, R. R., & Kmetz, C. (1997). The Index of Teaching Stress - A Measure of Student-Teacher Compatibility. Journal of School Psychology, 35(3), 239-259.

Harvey, E. (1998). Parental Employment and Conduct Problems Among Children with Attention-Deficit/Hyperactivity Disorder - An Examination of Child-Care Workload and Parenting Well-Being as Mediating Variables. Journal of Social and Clinical Psychology, 17(4), 476-490.

Harwood, M. D., & Eyberg, S. M. (2004). Therapist Verbal Behavior Early in Treatment: Relation to Successful Completion of Parent-Child Interaction Therapy. Journal of Clinical Child & Adolescent Psychology, 33(3), 601-612. doi: Article.

Heller, T. L., Baker, B. L., Henker, B., & Hinshaw, S. P. (1996). Externalizing Behavior and Cognitive-Functioning from Preschool to First-Grade - Stability and Predictors. Journal of Clinical Child Psychology, 25(4), 376-387.

Hindley, P. (1997). Psychiatric Aspects of Hearing Impairments. Journal of Child Psychology and Psychiatry and Allied Disciplines, 38(1), 101-117.

Joyner, K., Silver, C., & Stavinoha, P. (2009). Relationship Between Parenting Stress and Ratings of Executive Functioning in Children With ADHD, Journal of Psychoeducational Assessment, 27, 452-464.

Kelley, S. J. (1992). Parenting stress and child maltreatment in drug-exposed children. Child Abuse & Neglect, 16(3), 317-28.

Lacharite, C., Ethier, L., & Piche, C. (1992). Parental stress in mothers of preschool children: validation and Quebec norms for the Parental Stress Inventory. Sante Mentale au Quebec, 17(2), 183-203.

Lumley, V. A., McNeil, C. B., Herschell, A. D., & Bahl, A. B. (2002). An examination of gender differences among young children with disruptive behavior disorders. Child Study Journal, 32(2), 89-100.

Miranda, A., Grau, D., Rosel, J., & Meliá, A. (2009). Understanding discipline in families of children with attention-deficit/hyperactivity disorder: A structural equation model. The Spanish Journal of Psychology, 12(2), 496-505.

Murphy, K. R., & Barkley, R. A. (1996). Parents of Children with Attention-Deficit Hyperactivity Disorder - Psychological and Attentional Impairment. American Journal of Orthopsychiatry, 66(1), 93-102.

Nixon, R. D. V. (2001). Changes in hyperactivity and temperament in behaviourally disturbed preschoolers after Parent-Child Interaction Therapy (PCIT). Behaviour Change, 18 (3), 168-176.

Oord, S., Prins, P., Oosterlaan, J., & Emmelkamp, P. (2007). Does brief, clinically based, intensive multimodal behavior therapy enhance the effects of methylphenidate in children with ADHD? European Child & Adolescent Psychiatry, 16(1), 48-57. doi: 10.1007/s00787-006-0574-z.

Rogers, M., Wiener, J., Marton, I., & Tannock, R. (2009). Supportive and Controlling Parental Involvement as Predictors of Children's Academic Achievement: Relations to Children's ADHD Symptoms and Parenting Stress. School Mental Health, 1(2), 89-102.

Ross, C. N., Blanc, H. M., McNeil, C. B., Eyberg, S. M., & Hembree-Kigin, T. L. (1998). Parenting stress in mothers of young children with oppositional defiant disorder and other severe behavior problems. Child Study Journal, 28(2), 93-110.

Rostain, A. L., Power, T. J., & Atkins, M. S. (1993). Assessing Parents Willingness to Pursue Treatment for Children with Attention-Deficit Hyperactivity Disorder. Journal of the American Academy of Child and Adolescent Psychiatry, 32(1), 175-181.

Shelton, T. L., & Barkley, R. A. (1994). Critical Issues in the Assessment of Attention-Deficit Disorders in Children. Topics in Language Disorders, 14(4), 26-41.

Shelton, T. L., Barkley, R. A., Crosswait, C., Moorehouse, M., Fletcher, K., Barrett, S., Jenkins, L., & Metevia, L. (1998). Psychiatric and Psychological Morbidity as a Function of Adaptive Disability in Preschool-Children with Aggressive and Hyperactive-Impulsive-Inattentive Behavior. Journal of Abnormal Child Psychology, 26(6), 475-494.

Steele, M., Weiss, M., Swanson, J., Wang, J., Prinzo, R. S., & Binder, C. E. (2006). A randomized, controlled effectiveness trial of OROS-methylphenidate compared to usual care with immediate-release methylphenidate in attention deficit-hyperactivity disorder. Can J Clin Pharmacol, 13(1), e50–e62.

Tervo, R. (2010). Attention Problems and Parent-Rated Behavior and Stress in Young Children at Risk for Developmental Delay, Journal of Child Neurology, 25, 1325-1330.

Thunström, M. (2002). Severe sleep problems in infancy associated with subsequent development of attention-deficit/hyperactivity disorder at 5.5 years of age. Acta Paediatrica, 91(5), 584. doi: Article.

Tripp, G., Schaughency, E., Langlands, R., & Mouat, K. (2007). Family Interactions in Children With and Without ADHD. Journal of Child & Family Studies, 16(3), 385-400. doi: 10.1007/s10826-006-9093-2.

Tynan, W. D., & Nearing, J. (1994). The diagnosis of attention deficit hyperactivity disorder in young children. Infants & Young Children, 6(4), 13-20.

Tzang, R., Chang, Y., & Liu, S. (2009). The association between children's ADHD subtype and parenting stress and parental symptoms, International Journal of Psychiatry in Clinical Practice, 13, 318-325.

Birth Defects

Britner, P. A., Morog, M. C., Pianta, R. C., & Marvin, R.S. (2003). Stress and coping: a comparison of self-report measures of functioning in families of young children with cerebral palsy or no medical diagnosis. Journal of Child and Family Studies, 12(3), 335-348.

Colver, A. (2006). Study protocol: SPARCLE -- a multi-centre European study of the relationship of environment to participation and quality of life in children with cerebral palsy. BMC Public Health, 6, 105-110.

Glenn, S. S., Cunningham, C. C., Poole, H. H., Reeves, D. D., & Weindling, M. M. (2009). Maternal parenting stress and its correlates in families with a young child with cerebral palsy. Child: Care, Health and Development, 35(1), 71-78.

Hung, J. W., Wu, Y., & Yeh, C. (2004). Comparing stress levels of parents of children with cancer and parents of children with physical disabilities. Psycho-Oncology, 13(12), 898-903.

Biochemical Genetic Disorders

Bagner, D. M., & Eyberg, S. M. (2003). Father involvement in parent training: When does it matter?. Journal of Clinical Child & Adolescent Psychology, 32(4), 599-605.

Fedele, D., Grant, D., Wolfe-Christensen, C., Mullins, L., & Ryan, J. (2010). An examination of the factor structure of parenting capacity measures in chronic illness populations, Journal of Pediatric Psychology, 35, 1083-1092.

Waisbren, S. E., Rones, M., Read, C. Y., Marsden, D., & Levy, H. L. (2004). Brief report: Predictors of parenting stress among parents of children with biochemical genetic disorders. Journal of Pediatric Psychology, 29(7), 565-570.

Congenital Diseases

Geva, R., Yosipof, R., Eschel, R., Leitner, Y., Valevski, A. F., & Harel, S. (2009). Readiness and Adjustments to School for Children With Intrauterine Growth Restriction (IUGR): An Extreme Test Case Paradigm. Exceptional Children, 75(2), 211-230.

Hung, J. W., Wu, Y., & Yeh, C. (2004). Comparing stress levels of parents of children with cancer and parents of children with physical disabilities. Psycho-Oncology, 13(12), 898-903.

Limperopoulos, C., Robertson, R. L., Estroff, J. A., Barnewolt, C., Levine, D., Bassan, H., du Plessis, A. J. (2006). Diagnosis of inferior vermian hypoplasia by fetal magnetic resonance imaging: potential pitfalls and neurodevelopmental outcome. American Journal of Obstetrics and Gynecology, 194(4), 1070-1076. doi: 10.1016/j.ajog.2005.10.191

Wulffaert, J., Scholte, E., & van Berckelaer-Onnes, I. (2010). Maternal parenting stress in families with a child with Angelman syndrome or Prader–Willi syndrome, Journal of Intellectual & Developmental Disability, 35, 165-174.

Congenital Heart Disease

Bloom, A. A., Wright, J. A., Morris, R. D., Campbell, R. M., & Krawiecki, N. S. (1997). Additive Impact of In-hospital Cardiac Arrest on the Functioning of Children With Heart Disease. Pediatrics, 99(3), 390-398.

Darke, P. R., & Goldberg, S. (1994). Father-Infant interaction and parent stress with healthy and medically compromised infants. Infant Behavior & Development, 17(1), 3-14.

DeMaso, D. R., Campis, L. K., Wypij, D., Bertram, S., Lipshitz, M., & Freed, M. (1991). The impact of maternal perceptions and medical severity on the adjustment of children with congenital heart disease. Journal of Pediatric Psychology, 16(2), 137-49.

Goldberg, S., Janus, M., Washington, J., Simmons, R. J., Maclusky, I., & Fowler, R. S. (1997). Prediction of Preschool Behavioral-Problems in Healthy and Pediatric Samples. Journal of Developmental and Behavioral Pediatrics, 18(5), 304-313.

Goldberg, S., Morris, P., Simmons, R. J., Fowler, R. S., & Levison, H. (1990). Chronic illness in infancy and parenting stress: a comparison of three groups of parents. Journal of Pediatric Psychology, 15(3), 347-58.

Janus, M., & Goldberg, S. (1997). Factors Influencing Family Participation in a Longitudinal-Study - Comparison of Pediatric and Healthy Samples. Journal of Pediatric Psychology, 22(2), 245-262.

Majnemer, A., Limperopoulos, C., Shevell, M., Rohlicek, C., Rosenblatt, B. & Tchervenkov, C. (2006). Health and well-being of children with congenital cardiac malformations, and their families, following open-heart surgery. Cardiology in the Young, 16(2), 157-164.

Symons, D. K. (1998). Postpartum Employment Patterns, Family-Based Care Arrangements, and the Mother-Infant Relationship at Age-2. Canadian Journal of Behavioral Science, 30(2), 121-131.

Craniofacial Abnormalities

Andrews-Casal, M., Johnston, D., Fletcher, J., Mulliken, J. B., Stal, S., & Hecht, J. T. (1998). Cleft lip with or without cleft palate: effect of family history on reproductive planning, surgical timing, and parental stress. Cleft Palate Craniofacial Journal, 35(1), 52-7.

Campis, L. B., DeMaso, D. R., & Twente, A. W. (1995). The role of maternal factors in the adaptation of children with craniofacial disfigurement. Cleft Palate Craniofacial Journal, 32(1), 55-61.

Endriga, M. C. (1998). Feeding and Attachment in Infants with and Without Orofacial Clefts. Infant Behavior and Development, 21(4), 699-712.

Krueckeberg, S. M., & Kappsimon, K. A. (1993). Effect of Parental Factors on Social Skills of Preschool-Children with Craniofacial Anomalies. Cleft Palate-Craniofacial Journal, 30(5), 490-496.

Miller, A. C., Pit-Ten Cate, I. M., Watson, H. S., & Geronemus, R. G. (1999). Stress and family satisfaction in parents of children with facial port-wine stains. Pediatric Dermatology, 16(3), 190-7.

Sarimski, K. (1997). Parenting Stress in Families with Craniofacially Disordered Children. Praxis Der Kinderpsychologie und Kinderpsychiatrie, 46(1), 2-14.

Sarimski, K. (1998). Children with Apert-Syndrome - Behavioral-Problems and Family Stress. Developmental Medicine and Child Neurology, 40(1), 44-49.

Speltz, M. L., Goodell, E. W., Endriga, M. C., & Clarren, S. K. (1994). Feeding Interactions of Infants with Unrepaired Cleft-Lip and or Palate. Infant Behavior & Development, 17(2), 131-139.

Speltz, M. L., Endriga, M. C., Fisher, P. A., & Mason, C. A. (1997). Early Predictors of Attachment in Infants with Cleft-Lip and/or Palate. Child Development, 68(1), 12-25.

Cystic Fibrosis

Baroni, M. A., Anderson, Y. E., & Mischler, E. (1997). Cystic fibrosis newborn screening: impact of early screening results on parenting stress. Pediatric Nursing, 23(2), 143-51.

Bartholomew, L. K., Czyzewski, D. I., Parcel, G. S., Swank, P. R., Sockrider, M. M., Mariotto, M. J., Schidlow, D. V., Fink, R. J., & Seilheimer, D. K. (1997). Self-management of cystic fibrosis: short-term outcomes of the cystic fibrosis family education program. Health Education & Behavior, 24(5), 652-66.

Crist, W., Mcdonnell, P., Beck, M., Gillespie, C. T., Barrett, P., & Mathews, J. (1994). Behavior at Mealtimes and the Young-Child with Cystic-Fibrosis. Journal of Developmental and Behavioral Pediatrics, 15(3), 157-161.

Darke, P. R., & Goldberg, S. (1994). Father-Infant interaction and parent stress with healthy and medically compromised infants. Infant Behavior & Development, 17(1), 3-14.

Eddy, M. E., Carter, B. D., Kronenberger, W. G., Conradsen, S., Eid, N. S., Bourland, S. L., & Adams, G. (1998). Parent relationships and compliance in cystic fibrosis. Journal of Pediatric Health Care, 12(4), 196-202.

Fedele, D., Grant, D., Wolfe-Christensen, C., Mullins, L., & Ryan, J. (2010). An examination of the factor structure of parenting capacity measures in chronic illness populations, Journal of Pediatric Psychology, 35, 1083-1092.

Goldberg, S., Janus, M., Washington, J., Simmons, R. J., Maclusky, I., & Fowler, R. S. (1997). Prediction of Preschool Behavioral-Problems in Healthy and Pediatric Samples. Journal of Developmental and Behavioral Pediatrics, 18(5), 304-313.

Goldberg, S., Morris, P., Simmons, R. J., Fowler, R. S., & Levison, H. (1990). Chronic illness in infancy and parenting stress: a comparison of three groups of parents. Journal of Pediatric Psychology, 15(3), 347-58.

Janus, M., & Goldberg, S. (1997). Factors Influencing Family Participation in a Longitudinal-Study - Comparison of Pediatric and Healthy Samples. Journal of Pediatric Psychology, 22(2), 245-262.

Krueckeberg, S. M., & Kappsimon, K. A. (1993). Effect of Parental Factors on Social Skills of Preschool-Children with Craniofacial Anomalies. Cleft Palate-Craniofacial Journal, 30(5), 490-496.

Quittner, A. L., Digirolamo, A. M., Michel, M., & Eigen, H. (1992). Parental Response to Cystic-Fibrosis - A Contextual Analysis of the Diagnosis Phase. Journal of Pediatric Psychology, 17(6), 683-704.

Solomon, C. R., & Breton, J. (1999). Early warning signals in relationships between parents and young children with cystic fibrosis. Children’s Health Care, 28(3), 221-40.

Vieira Santos, S. (1994). Characteristics of stress in parents of children with chronic diseases and in parents of children with emotional problems. Analise Psicologica, 12(2-3).

Spina Bifida

Cappelli, M., Mcgrath, P. J., Daniels, T., Manion, I., & Schillinger, J. (1994). Marital Quality of Parents of Children with Spina-Bifida - A Case-Comparison Study. Journal of Developmental and Behavioral Pediatrics, 15(5), 320-326.

Greenley, R. N., Holmbeck, G. N., & Rose, B. M. (2006). Predictors of Parenting Behavior Trajectories Among Families of Young Adolescents with and without Spina Bifida. J. Pediatr. Psychol., 31(10), 1057-1071. doi: 10.1093/jpepsy/jsj011.

Holmbeck, G. N., Goreyferguson, L., Hudson, T., Seefeldt, T., Shapera, W., Turner, T., & Uhler, J. (1997). Maternal, Paternal, and Marital Functioning in Families of Preadolescents with Spina-Bifida. Journal of Pediatric Psychology, 22(2), 167-181.

Hung, J. W., Wu, Y., & Yeh, C. (2004). Comparing stress levels of parents of children with cancer and parents of children with physical disabilities. Psycho-Oncology, 13(12), 898-903.

Kazak, A. E., & Marvin, R. S. (1984). Differences, difficulties and adaptation: Stress and social networks in families with a handicapped child. Family Relations: Journal of Applied Family & Child Studies, 33(1), 67-77.

Macias, M. M., Saylor, C. F., Haire, K. B., & Bell, N. L. (2007). Predictors of paternal versus maternal stress in families of children with neural tube defects. Children's Health Care, 36(2), 99-115.

Vermaes, I. P. R., Janssens, J. M. A. M., Mullaart, R. A., Vinck, A., & Gerris, J. R. M. (2008). Parents' personality and parenting stress in families of children with spina bifida. Child: Care, Health & Development, 34(5),665-674.

Behavior Problems

Baker, A. J. L., Gries, L., Schneiderman, M., Parker, R., Archer, M., & Friedrich, B. (2008). Children with Problematic Sexualized Behaviors in the Child Welfare System. Child Welfare, 87(1), 5. doi: Article.

Beck, A., Hastings, R. P., Daley, D., & Stevenson, J. (2004). Pro-social behaviour and behaviour problems independently predict maternal stress. Journal of Intellectual and Developmental Disability, 29(4), 339–349.

Beg, M. R., Casey, J. E., & Saunders, C. D. (2007). A typology of behavior problems in preschool children. Assessment, 14(2), 111-128.

Brown, J. V., Bakeman, R., Coles, C. D., Platzman, K. A., & Lynch, M. E. (2004). Prenatal cocaine exposure: A comparison of 2-year-old children in parental and nonparental care. Child Development, 75(4), 1282–1295.

Butz, A. M., Pulsifer, M., Marano, N., Belcher, H., Lears, M. K., & Royall, R. (2001). Effectiveness of a home intervention for perceived child behavioral problems and parenting stress in children with in utero drug exposure. Archives of Pediatrics & Adolescent Medicine, 155(9), 1029-1037.

Bywater, T., Hutchings, J., Daley, D., Whitaker, C., Yeo, S. T., Jones, K., et al. (2009). Long-term effectiveness of a parenting intervention for children at risk of developing conduct disorder. The British Journal of Psychiatry: The Journal of Mental Science, 195(4), 318-324. doi: 10.1192/bjp.bp.108.056531

Calam, R., Bolton, C., & Roberts, J. (2002). Maternal expressed emotion, attributions and depression and entry into therapy for children with behaviour problems. British Journal of Clinical Psychology, 41(2), 213216.

Chang, S. S. Y., Ng, C. F. N., & Wong, S. N. (2002). Behavioural problems in children and parenting stress associated with primary nocturnal enuresis in Hong Kong. Acta Paediatrica, 91(4), 475-479.

Chazan-Cohen, R., Ayoub, C., Pan, B. A., Roggman, L., Raikes, H., Mckelvey, L., et al. (2007). It takes time: Impacts of Early Head Start that lead to reductions in maternal depression two years later. Infant Mental Health Journal, 28(2), 151-170. doi: 10.1002/imhj.20127.

Cole, S. A. (2002). Security of Attachment of Infants in Foster Care. In Proceedings of the... National Symposium on Doctoral Research in Social Work (p. 12). Presented at the National Symposium on Doctoral Research in Social Work, Columbus, OH: Ohio State University.

De Bruyne, E., Van Hoecke, E., Van Gompel, K., Verbeken, S., Baeyens, D., et al. (2009). Problem Behavior, Parental Stress and Enuresis. The Journal of Urology, 182(4), 2015-2021. doi: 10.1016/j.juro.2009.05.102

De Los Reyes, A., & Kazdin, A. E. (2006). Informant Discrepancies in Assessing Child Dysfunction Relate to Dysfunction Within Mother-Child Interactions. Journal of Child & Family Studies, 15(5), 643-661. doi: 10.1007/s10826-006-9031-3.

Deković, M., Janssens, J. M., & van As, N. M. (2003). Family Predictors of Antisocial Behavior in Adolescence. Family Process, 42(2), 223. doi: Article.

DeMore, M., Adams, C., Wilson, N., & Hogan, M. B. (2005). Parenting Stress, Difficult Child Behavior, and Use of Routines in Relation to Adherence in Pediatric Asthma. Children's Health Care, 34(4), 245-259. doi: 10.1207/s15326888chc3404_1.

Embregts, P., Grimbel du Bois, M., & Graef, N. (2010). Behavior problems in children with mild intellectual disabilities: An initial step towards prevention. Research in Developmental Disabilities, 31, 1398-1403.

Feinfield, K. A., & Baker, B. L. (2004). Empirical support for a treatment program for families of young children with externalizing problems. Journal of Clinical Child and Adolescent Psychology: The Official Journal for the Society of Clinical Child and Adolescent Psychology, American Psychological Association, Division 53, 33(1), 182-195. doi: 10.1207/S15374424JCCP3301_17

Flake, E., Davis, B., Johnson, P., & Middleton, L. (2009). The Psychosocial Effects of Deployment on Military Children, Journal of Developmental & Behavioral Pediatrics, 30, 271-278.

Lauth, G. W., Otte, T., & Heubeck, B. G. (2009). Effectiveness of a competence training programme for parents of socially disruptive children. Emotional & Behavioural Difficulties, 14(2), 117-126.

Mäntymaa, M., Puura, K., Luoma, I., Salmelin, R. K., & Tamminen, T. (2006). Mother's early perception of her infant's difficult temperament, parenting stress and early mother-infant interaction. Nordic Journal of Psychiatry, 60(5), 379-386.

Richman, D., Belmont, J., Kim, M., Slavin, C., & Hayner, A. (2009). Parenting stress in families of children with Cornelia de Lange Syndrome and Down Syndrome, Journal of Developmental and Physical Disabilities, 21, 537-553.

Tervo, R. (2010). Attention Problems and Parent-Rated Behavior and Stress in Young Children at Risk for Developmental Delay, Journal of Child Neurology, 25, 1325-1330.

Wulffaert, J., Scholte, E., & van Berckelaer-Onnes, I. (2010). Maternal parenting stress in families with a child with Angelman syndrome or Prader–Willi syndrome, Journal of Intellectual & Developmental Disability, 35, 165-174.

Child Abuse

Acton, R. G., & During, S. M. (1992). Preliminary results of aggression management training for aggressive. Journal of Interpersonal Violence, 7(3), 410-417.

Adamakos, H., Kathleen, R., G., U. D., & John, P. (1986). Maternal social support as a predictor of mother-child stress and stimulation. Child Abuse & Neglect, 10(4), 463-470.

Ammerman, R. T., & Patz, R. J. (1996). Determinants of Child-Abuse Potential - Contribution of Parent and Child Factors. Journal of Clinical Child Psychology, 25(3), 300-307.

Black, M. M., Nair, P., & Harrington, D. (1994). Maternal HIV-Infection - Parenting and Early Child-Development. Journal of Pediatric Psychology, 19(5), 595-616.

Black, M. M., Nair, P., Kight, C., Wachtel, R., Roby, P., & Schuler, M. (1994). Parenting and early development among children of drug-abusing women: effects of home intervention. Pediatrics, 94(4 Pt 1), 440-8.

Budd, K. S., & Holdsworth, M. J. (1996). Issues in Clinical-Assessment of Minimal Parenting Competence. Journal of Clincal Child Psychology, 25(1), 2-14.

Burrell, B., Thompson, B., & Sexton, D. (1994). Predicting Child-Abuse Potential Across Family Types. Child Abuse & Neglect, 18(12), 1039-1049.

Chan, Y. C. (1994). Parenting stress and social support of mothers who physically abuse their children in Hong Kong. Child Abuse & Neglect, 18(3), 261-9.

Cornish, A. M., McMahon, C. A., Ungerer, J. A., Barnett, B., Kowalenko, N., & Tennant, C. (2006). Maternal depression and the experience of parenting in the second postnatal year. Journal of Reproductive & Infant Psychology, 24(2), 121-132. doi: 10.1080/02646830600644021.

DePanfilis, D., & Dubowitz, H. (2005). Family connections: a program for preventing child neglect. Child Maltreatment, 10(2), 108-123. doi: 10.1177/1077559505275252.

DiLauro, M. D. (2004). Psychosocial Factors Associated with Types of Child Maltreatment. Child Welfare, 83(1), 69. doi: Article.

Dixon, L., Hamilton-Giachritsis, C., Browne, K., & Ostapuik, E. (2007). The Co-occurrence of Child and Intimate Partner Maltreatment in the Family: Characteristics of the Violent Perpetrators. Journal of Family Violence, 22(8), 675-689. doi: 10.1007/s10896-007-9115-x.

Donohue-Moore, M. (1994). Commentary on Caregiver stress in grandparents raising grandchildren. Nursing Scan in Oncology, 3(4), 10.

Douglas, A. R. (2000). Reported anxieties concerning intimate parenting in women sexually abused as children. Child Abuse & Neglect, 24(3), 425-34.

Duggan, A., Caldera, D., Rodriguez, K., Burrell, L., Rohde, C., & Crowne, S. S. (2007). Impact of a statewide home visiting program to prevent child abuse. Child Abuse & Neglect, 31(8), 801-827. doi: 10.1016/j.chiabu.2006.06.011.

Dukewich, T. L., Borkowski, J. G., & Whitman, T. L. (1996). Adolescent Mothers and Child-Abuse Potential - An Evaluation of Risk-Factors. Child Abuse & Neglect, 20(11), 1031-1047.

El-Kamary, S. S., Higman, S. M., Fuddy, L., McFarlane, E., Sia, C., & Duggan, A. K. (2004). Hawaii's healthy start home visiting program: determinants and impact of rapid repeat birth. Pediatrics, 114(3), e317-326. doi: 10.1542/peds.2004-0618.

Ethier, L. S. (1992). Developmental factors related to stress in neglectful and abusive mothers. Apprentissage et Socialisation, 15(3), 222-236.

Ethier, L. S., Lacharite, C., & Couture, G. (1995). Childhood adversity, parental stress, and depression of negligent mothers. Child Abuse & Neglect, 19(5), 619-32.

Fuscaldo, D., Kaye, J. W., & Philliber, S. (1998). Evaluation of a Program for Parenting. Families in Society:The Journal of Contemporary Human Services, 79(1), 53-61.

Garbarino, J., & Kostelny, K. (1996). The Effects of Political Violence on Palestinian Childrens Behavior Problems - A Risk Accumulation Model. Child Development, 67(1), 33-45.

Gershater-Molko, R. M., Lutzker, J. R., & Wesch, D. (2002). Using recidivism data to evaluate Project SafeCare: teaching bonding, safety, and health care skills to parents. Child Maltreatment, 7(3), 277-285.

Gorzka, P. A. (1999). Homeless parents’ perceptions of parenting stress. Journal of Child & Adolescent Psychiatric Nursing, 12(1), 7-16.

Harmer, A. L. M., Sanderson, J., & Mertin, P. (1999). Influence of Negative Childhood Experiences on Psychological Functioning, Social Support, and Parenting for Mothers Recovering from Addiction. Child Abuse & Neglect, 23(5), 421-433.

Harrington, D., Black, M. M., Starr, R. H., & Dubowitz, H. (1998). Child Neglect - Relation to Child Temperament and Family Context. American Journal of Orthopsychiatry, 68(1), 108-116.

Haskett, M., Scott, S., Willoughby, M., Ahern, L., & Nears, K. (2006). The Parent Opinion Questionnaire and Child Vignettes for Use with Abusive Parents: Assessment of Psychometric Properties. Journal of Family Violence, 21(2), 137-151. doi: 10.1007/s10896-005-9010-2.

Heinze, M. C., & Grisso, T. (1996). Review of instruments assessing parenting competencies used in child custody evaluations. Behavioral Sciences & the Law, 14(3), 293-313.

Holden, E. W., & Banez, G. A. (1996). Child abuse potential and parenting stress within maltreating families. Journal of Family Violence, 11(1), 1-12.

Hsu, E., Davies, C. A., Hans, L., Sedlar, G., Nash, C. L., Holm, J. W., et al. (2001). Parallel group treatments for sexually abused children and their nonoffending caregivers: Child and family outcome and satisfaction. Presented at the 35th Annual Convention of the Association for the Advancement of Behavioral Therapy, Philadelphia, PA.

Jacobsen, T., & Miller, L. J. (1998). Mentally Ill Mothers Who Have Killed - 3 Cases Addressing the Issue of Future Parenting Capability. Psychiatric Services, 49(5), 650-657.

Jacobsen, T., Miller, L. J., & Kirkwood, K. P. (1997). Assessing Parenting Competence in Individuals with Severe Mental-Illness - A Comprehensive Service. Journal of Mental Health Administration, 24(2), 189-199.

Kelley, S. J. (1992). Parenting stress and child maltreatment in drug-exposed children. Child Abuse & Neglect, 16(3), 317-28.

Kelley, S. J. (1993). Caregiver stress in grandparents raising grandchildren. Image the Journal of Nursing Scholarship, 25(4), 331-7.

Lacharite, C., Ethier, L. S., & Couture, G. (1999). Sensitivity and specificity of the Parenting Stress Index in situations of child maltreatment. Canadian Journal of Behavioural Science, 31(4), 217-220.

Lederman, C., & Osofsky, J. (2004). Infant mental health interventions in juvenile court. Psychology, Public Policy, and Law, 10(1-2), 162-177. doi: 10.1037/1076-8971.10.1.162.

Ligezinska, M., Firestone, P., Manion, I. G., Mcintyre, J., Ensom, R., & Wells, G. (1996). Childrens Emotional and Behavioral Reactions Following the Disclosure of Extrafamilial Sexual Abuse - Initial Effects. Child Abuse & Neglect, 20(2), 111-125.

Lutzker, J. R., Bigelow, K. M., Doctor, R. M., & Kessler, M. L. (1998). Safety, health care, and bonding within an ecobehavioral approach to treating and preventing child abuse and neglect. Journal of Family Violence, 13(2), 163-185.

Lutzker, J. R., Vanhasselt, V. B., Bigelow, K. M., Greene, B. F., & Kessler, M. L. (1998). Child-Abuse and Neglect - Behavioral-Research, Treatment, and Theory. Aggression and Violent Behavior, 3(2), 181-196.

Manion, I. G., Mcintyre, J., Firestone, P., Ligezinska, M., Ensom, R., & Wells, G. (1996). Secondary Traumatization in Parents Following the Disclosure of Extrafamilial Child Sexual Abuse - Initial Effects. Child Abuse & Neglect, 20(11), 1095-1109.

Marshall, E., Buckner, E., Perkins, J., Lowry, J., Hyatt, C., Campbell, C., & Helms, D. (1996). Effects of child abuse prevention unit in health classes in four schools. Journal of Community Health Nursing, 13(2), 107-22.

McPherson, A., Lewis, K., Lynn, A., Haskett, M., & Behrend, T. (2009). Predictors of Parenting Stress for Abusive and Nonabusive Mothers. Journal of Child and Family Studies, 18(1), 61-69. doi: 10.1007/s10826-008-9207-0.

Milner, J. S. (1994). Assessing Physical Child-Abuse Risk - The Child-Abuse Potential Inventory. Clinical Psychology Review, 14(6), 547-583.

Milner, J. S., & Crouch, J. L. (1997). Impact and detection of response distortions on parenting measures used to assess risk for child physical abuse. Journal of Personality Assessment, 69(3), 633-50.

Milner, J. S., & Murphy, W. D. (1995). Assessment of Child Physical and Sexual Abuse Offenders. Family Relations, 44(4), 478-488.

Mylod, D. E., Whitman, T. L., & Borkowski, J. G. (1997). Predicting Adolescent Mothers Transition to Adulthood. Journal of Research on Adolescence, 7(4), 457-478.

Nam, Y., Meezan, W., & Danziger, S. K. (2006). Welfare recipients' involvement with child protective services after welfare reform. Child abuse & neglect, 30(11), 1181–1199.

Nereo, N. E., Fee, R. J., & Hinton, V. J. (2003). Parental stress in mothers of boys with Duchenne muscular dystrophy. Journal of Pediatric Psychology, 28(7), 473-484.

Olsen, L. J., Allen, D., & Azzilessing, L. (1996). Assessing Risk in Families Affected by Substance-Abuse. Child Abuse & Neglect, 20(9), 833-842.

Park, U. I. (1995). Patterns of infant-mother attachment and related variables. Korean Journal of Child Studies, 16(1), 113-131.

Patterson, K. A., & Starn, J. R. (1993). Program for women and infants exposed to drugs: a legal alternative. Nurse Practitioner Forum, 4(4), 224-30.

Pithers, W. D., Gray, A., Busconi, A., & Houchens, P. (1998). Caregivers of children with sexual behavior problems: Psychological and familial functioning. Child Abuse & Neglect, 22(2), 129-141.

Pithers, W. D., Gray, A., Busconi, A., & Houchens, P. (1998). Children with sexual behavior problems: identification of five distinct child types and related treatment considerations. Child Maltreatment, 3(4), 384-406.

Rodriguez, C. M., & Green, A. J. (1997). Parenting stress and anger expression as predictors of child abuse potential. Child Abuse & Neglect, 21(4), 367-77.

Rosenstein, P. (1995). Parental Levels of Empathy as Related to Risk Assessment in Child Protective Services. Child Abuse & Neglect, 19(11), 1349-1360.

Sawyer, G. K., Yancey, C. T., Tsao, E. H., Wynne, A., Hansen, D. J., & Flood, M. F. (2005). Parallel group treatments for sexually abused youth and their nonoffending parents: Treatment integrity, outcomes and social validity of Project SAFE. Presented at the 39th Annual Convention of the Association for Behavioral and Cognitive Therapies, Washington, DC.

Timmer, S. G., Borrego, J., & Urquiza, A. J. (2002). Antecedents of Coercive Interactions in Physically Abusive Mother-Child Dyads. J Interpers Violence, 17(8), 836-853. doi: 10.1177/0886260502017008003.

Whipple, E. E. (1999). Reaching Families with Preschoolers at Risk of Physical Child-Abuse - What Works. Families in Society-The Journal of Contemporary Human Services, 80(2), 148-160.

Winton, M. A. (1990). An evaluation of a support group for parents who have a sexually abused child. Child Abuse & Neglect, 14(3), 397-405.

Wolock, I., & Magura, S. (1996). Parental Substance-Abuse as a Predictor of Child Maltreatment Re-Reports. Child Abuse & Neglect, 20(12), 1183-1193.

Wright, M., Crawford, E., & Sebastian, K. (2007). Positive Resolution of Childhood Sexual Abuse Experiences: The Role of Coping, Benefit-Finding and Meaning-Making. Journal of Family Violence, 22(7), 597-608. doi: 10.1007/s10896-007-9111-1.

Child Abuse Risk Assessment

Adamakos, H., Kathleen, R., G., U. D., & John, P. (1986). Maternal social support as a predictor of mother-child stress and stimulation. Child Abuse & Neglect, 10(4), 463-470.

Ammerman, R. T., & Patz, R. J. (1996). Determinants of Child-Abuse Potential - Contribution of Parent and Child Factors. Journal of Clinical Child Psychology, 25(3), 300-307.

Black, M. M., Nair, P., & Harrington, D. (1994). Maternal HIV-Infection - Parenting and Early Child-Development. Journal of Pediatric Psychology, 19(5), 595-616.

Buist, A. (1998). Childhood Abuse, Parenting and Postpartum Depression. Australian and New Zealand Journal of Psychiatry, 32(4), 479-487.

Chan, Y. C. (1994). Parenting stress and social support of mothers who physically abuse their children in Hong Kong. Child Abuse & Neglect, 18(3), 261-9.

Combs-Orme, T., Cain, D. S., & Wilson, E. E. (2004). Do maternal concerns at delivery predict parenting stress during infancy? Child Abuse & Neglect, 28(4), 377–392.

DiLauro, M. D. (2004). Psychosocial Factors Associated with Types of Child Maltreatment. Child Welfare, 83(1), 69. doi: Article.

Duggan, A., Caldera, D., Rodriguez, K., Burrell, L., Rohde, C., & Crowne, S. S. (2007). Impact of a statewide home visiting program to prevent child abuse. Child Abuse & Neglect, 31(8), 801-827. doi: 10.1016/j.chiabu.2006.06.011.

Dukewich, T. L., Borkowski, J. G., & Whitman, T. L. (1996). Adolescent Mothers and Child-Abuse Potential - An Evaluation of Risk-Factors. Child Abuse & Neglect, 20(11), 1031-1047.

Ethier, L. S., Lacharite, C., & Couture, G. (1995). Childhood adversity, parental stress, and depression of negligent mothers. Child Abuse & Neglect, 19(5), 619-32.

Grietens, H., Haene, L., & Uyttebroek, K. (2007). Cross-cultural Validation of the Child Abuse Potential Inventory in Belgium (Flanders): Relations with Demographic Characteristics and Parenting Problems. Journal of Family Violence, 22(4), 223-229. doi: 10.1007/s10896-007-9074-2.

Harnett, P. H., & Dawe, S. (2008). Reducing Child Abuse Potential in Families Identified by Social Services: Implications for Assessment and Treatment. Brief Treatment and Crisis Intervention, 8(3), 226-235.

Huebner, C. E. (2002). Evaluation of a clinic-based parent education program to reduce the risk of infant and toddler maltreatment. Public Health Nursing, 19(5), 377–389.

Jacobsen, T., & Miller, L. J. (1998). Mentally Ill Mothers Who Have Killed - 3 Cases Addressing the Issue of Future Parenting Capability. Psychiatric Services, 49(5), 650-657.

Jacobsen, T., Miller, L. J., & Kirkwood, K. P. (1997). Assessing Parenting Competence in Individuals with Severe Mental-Illness - A Comprehensive Service. Journal of Mental Health Administration, 24(2), 189-199.

Kelley, S. J. (1992). Parenting stress and child maltreatment in drug-exposed children. Child Abuse & Neglect, 16(3), 317-28.

Kelley, S. J. (1998). Stress and coping behaviors of substance-abusing mothers. Journal of the Society of Pediatric Nurses, 3(3), 103-10.

Levendosky, A. A., & Grahambermann, S. A. (1998). The Moderating Effects of Parenting Stress on Childrens Adjustment in Woman-Abusing Families. Journal of Interpersonal Violence, 13(3), 383-397.

Lutzker, J. R., Bigelow, K. M., Doctor, R. M., & Kessler, M. L. (1998). Safety, health care, and bonding within an ecobehavioral approach to treating and preventing child abuse and neglect. Journal of Family Violence, 13(2), 163-185.

Milner, J. S. (1994). Assessing Physical Child-Abuse Risk - The Child-Abuse Potential Inventory. Clinical Psychology Review, 14(6), 547-583.

Milner, J. S., & Crouch, J. L. (1997). Impact and detection of response distortions on parenting measures used to assess risk for child physical abuse. Journal of Personality Assessment, 69(3), 633-50.

Milner, J. S., & Murphy, W. D. (1995). Assessment of Child Physical and Sexual Abuse Offenders. Family Relations, 44(4), 478-488.

Mullick, M., Miller, L. J., & Jacobsen, T. (2001). Insight into mental illness and child maltreatment risk among mothers with major psychiatric disorders. Psychiatric Services, 52(4), 488-492.

Nicholson, B., Anderson, M., Fox, R., & Brenner, V. (2002). One Family at a Time: a prevention program for at-risk parents. Journal of Counseling & Development, 80(3), 362-371.

Olsen, L. J., Allen, D., & Azzilessing, L. (1996). Assessing Risk in Families Affected by Substance-Abuse. Child Abuse & Neglect, 20(9), 833-842.

Rosenstein, P. (1995). Parental Levels of Empathy as Related to Risk Assessment in Child Protective Services. Child Abuse & Neglect, 19(11), 1349-1360.

Rudo, Z. H., Powell, D. S., & Dunlap, G. (1998). The Effects of Violence in the Home on Childrens Emotional, Behavioral, and Social Functioning - A Review of the Literature. Journal of Emotional and Behavioral Disorders, 6(2), 94-113.

Sawyer, G. K., Di Loreto, A. R., Flood, M. F., & DiLillo, D. (2002, November). Parent-Child Relationship and Family Variables as Predictors of Child Abuse Potential: Implications for Assessment and Early Intervention. Presented at the Annual Convention of the Association for the Advancement of Behavioral Therapy, Poster, Reno, Nevada. Retrieved November 19, 2009.

Schaeffer, C. M., Alexander, P. C., Bethke, K., & Kretz, L. S. (2005). Predictors of Child Abuse Potential Among Military Parents: Comparing Mothers and Fathers. Journal of Family Violence, 20(2), 123-129. doi: 10.1007/s10896-005-3175-6.

Taylor, C. A., Guterman, N. B., Lee, S. J., & Rathouz, P. J. (2009). Intimate Partner Violence, Maternal Stress, Nativity, and Risk for Maternal Maltreatment of Young Children. American Journal of Public Health, 99(1), 175. doi: 10.2105/AJPH.2007.126722.

Taylor, J. A., & Kemper, K. J. (1998). Group well-child care for high-risk families: maternal outcomes. Archives of Pediatrics & Adolescent Medicine, 152(6), 579-84.

Taylor, J. A., Davis, R. L., & Kemper, K. J. (1997). A Randomized Controlled Trial of Group Versus Individual Well Child-Care for High-Risk Children - Maternal-Child Interaction and Developmental Outcomes. Pediatrics, 99(6), E91-E96.

Van Hasselt, V. B., Hersen, M., Null, J. A., Ammerman, R. T., Bukstein, O. G., Mcgillivray, J., & Hunter, A. (1993). Drug-Abuse Prevention for High-Risk African-American Children and Their Families - A Review and Model Program. Addictive Behaviors, 18(2), 213-234.

Weberling, L. C., Forgays, D. K., Crain-Thoreson, C., & Hyman, I. (2003). Prenatal Child Abuse Risk Assessment: A Preliminary Validation Study. Child Welfare, 82(3), 319. doi: Article.

Whipple, E. E. (1999). Reaching Families with Preschoolers at Risk of Physical Child-Abuse - What Works. Families in Society-The Journal of Contemporary Human Services, 80(2), 148-160.

Chronic Health Disease

Abidin, R. R. (1983). Parenting stress and the utilization of pediatric services. Children's Health Care, 11(2), 70-3.

Bendell, D., Goldberg, M. S., Urbano, M. T., & Urbano, R. C. (1987). Differential impact of parenting sick infants. Infant Mental Health Journal, 8(1), 28-36.

Bloom, A. A., Wright, J. A., Morris, R. D., Campbell, R. M., & Krawiecki, N. S. (1997). Additive Impact of In-hospital Cardiac Arrest on the Functioning of Children With Heart Disease. Pediatrics, 99(3), 390-398.

Britner, P. A., Morog, M. C., Pianta, R. C., & Marvin, R.S. (2003). Stress and coping: a comparison of self-report measures of functioning in families of young children with cerebral palsy or no medical diagnosis. Journal of Child and Family Studies, 12(3), 335-348.

Cappelli, M., Mcgrath, P. J., Daniels, T., Manion, I., & Schillinger, J. (1994). Marital Quality of Parents of Children with Spina-Bifida - A Case-Comparison Study. Journal of Developmental and Behavioral Pediatrics, 15(5), 320-326.

Crist, W., Mcdonnell, P., Beck, M., Gillespie, C. T., Barrett, P., & Mathews, J. (1994). Behavior at Mealtimes and the Young-Child with Cystic-Fibrosis. Journal of Developmental and Behavioral Pediatrics, 15(3), 157-161.

DeGangi, G. A., Sickel, R. Z., Kaplan, E. P., & Wiener, A. S. (1997). Mother-infant interactions in infants with disorders of self-regulation. Physical & Occupational Therapy in Pediatrics, 17(1), 17-44.

DeGangi, G. A., Sickel, R. Z., Wiener, A. S., & Kaplan, E. P. (1996). Fussy babies: to treat or not to treat? British Journal of Occupational Therapy, 59(10), 457-64.

Dellve, L., Samuelsson, L., Tallborn, A., Fasth, A., & Hallberg, L. R. (2006). Stress and well-being among parents of children with rare diseases: a prospective intervention study. Journal of Advanced Nursing, 53(4), 392-402. doi: 10.1111/j.1365-2648.2006.03736.x.

DeMaso, D. R., Campis, L. K., Wypij, D., Bertram, S., Lipshitz, M., & Freed, M. (1991). The impact of maternal perceptions and medical severity on the adjustment of children with congenital heart disease. Journal of Pediatric Psychology, 16(2), 137-49.

Douglas, J. E., Hulson, B., & Trompeter, R. S. (1998). Psychosocial Outcome of Parents and Young-Children After Renal-Transplantation. Child Care Health and Development, 24(1), 73-83.

Dow, K. H., Harris, J. R., & Roy, C. (1994). Pregnancy after breast-conserving surgery and radiation therapy for breast cancer. Journal of the National Cancer Institute, 16, 131-7.

Fisman, S., Wolf, L., Ellison, D., Gillis, B., Freeman, T., & Szatmari, P. (1996). Risk and Protective Factors Affecting the Adjustment of Siblings of Children with Chronic Disabilities. Journal of the American Academy of Child and Adolescent Psychiatry, 35(11), 1532-1541.

Gavin, L., & Wysocki, T. (2006). Associations of paternal involvement in disease management with maternal and family outcomes in families with children with chronic illness. Journal of Pediatric Psychology, 31(5), 481-489.

Girolametto, L., & Tannock, R. (1994). Correlates of Directiveness in the Interactions of Fathers and Mothers of Children with Developmental Delays. Journal of Speech and Hearing Research, 37(5), 1178-1191.

Goldberg, S., Janus, M., Washington, J., Simmons, R. J., Maclusky, I., & Fowler, R. S. (1997). Prediction of Preschool Behavioral-Problems in Healthy and Pediatric Samples. Journal of Developmental and Behavioral Pediatrics, 18(5), 304-313.

Goldberg, S., Morris, P., Simmons, R. J., Fowler, R. S., & Levison, H. (1990). Chronic illness in infancy and parenting stress: a comparison of three groups of parents. Journal of Pediatric Psychology, 15(3), 347-58.

Goldberg, S., Simmons, R. J., Newman, J., Campbell, K., & Fowler, R. S. (1991). Congenital Heart-Disease, Parental Stress, and Infant-Mother Relationships. Journal of Pediatrics, 119(4), 661-666.

Gottlieb, L. N., & Feeley, N. (1996). The McGill Model of Nursing and children with a chronic condition: "who benefits, and why?". Canadian Journal of Nursing Research, 28(3), 29-48.

Janus, M., & Goldberg, S. (1997). Factors Influencing Family Participation in a Longitudinal-Study - Comparison of Pediatric and Healthy Samples. Journal of Pediatric Psychology, 22(2), 245-262.

Kazak, A. E., & Barakat, L. P. (1997). Brief Report - Parenting Stress and Quality-of-Life During Treatment for Childhood Leukemia Predicts Child and Parent Adjustment After Treatment Ends. Journal of Pediatric Psychology, 22(5), 749-758.

Kazak, A. E., Penati, B., Boyer, B. A., Himelstein, B., Brophy, P., Waibel, M. K., Blackall, G. F., Daller, R., & Johnson, K. (1996). A Randomized Controlled Prospective Outcome Study of a Psychological and Pharmacological Intervention Protocol for

Procedural Distress in Pediatric Leukemia. Journal of Pediatric Psychology, 21(5), 615-631.

Kazak, A. E., Penati, B., Waibel, M. K., & Blackall, G. F. (1996). The Perception of Procedures Questionnaire: psychometric properties of a brief parent report measure of procedural distress. Journal of Pediatric Psychology, 21(2), 195-207.

Manassis, K., Bradley, S., Goldberg, S., Hood, J., & Swinson, R. P. (1994). Attachment in Mothers with Anxiety Disorders and Their Children. Journal of the American Academy of Child and Adolescent Psychiatry, 33(8), 1106-1113.

Manassis, K., Bradley, S., Goldberg, S., Hood, J., & Swinson, R. P. (1995). Behavioral-Inhibition, Attachment and Anxiety in Children of Mothers with Anxiety Disorders. Canadian Journal of Psychiatry, 40(2), 87-92.

Onufrak, B., Saylor, C. F., Taylor, M. J., Eyberg, S. M., & Boyce, G. C. (1995). Determinants of responsiveness in mothers of children with intraventricular hemorrhage. Journal of Pediatric Psychology, 20(5), 587-99.

Quittner, A. L., Digirolamo, A. M., Michel, M., & Eigen, H. (1992). Parental Response to Cystic-Fibrosis - A Contextual Analysis of the Diagnosis Phase. Journal of Pediatric Psychology, 17(6), 683-704.

Soliday, E., & Lande, M. B. (2002). Family structure and the course of steroid-sensitive nephrotic syndrome. Pediatric Nephrology, 17(1), 41. doi: Article.

Vieira Santos, S. (1994). Characteristics of stress in parents of children with chronic diseases and in parents of children with emotional problems. Analise Psicologica, 12(2-3).

Wulffaert, J., Scholte, E. M., Dijkxhoorn, Y. M., Bergman, J. E., van Ravenswaaij-Arts, C. M., & van Berckelaer-Onnes, I. A. (2009). Parenting stress in CHARGE syndrome and the relationship with child characteristics. Journal of Developmental and Physical Disabilities, 21(4), 301-313.

Wyman, P. A., Moynihan, J., Eberly, S., Cox, C., Cross, W., Jin, X., et al. (2007). Association of Family Stress With Natural Killer Cell Activity and the Frequency of Illnesses in Children. Arch Pediatr Adolesc Med, 161(3), 228-234. doi: 10.1001/archpedi.161.3.228.

Yu, M. S., Norris, J. M., Mitchell, C. M., Butler-Simon, N., Groshek, M., Follansbee, D., Erlich, H., Rewers, M., & Klingensmith, G. J. (1999). Impact on maternal parenting stress of receipt of genetic information regarding risk of diabetes in newborn infants. American Journal of Medical Genetics, 86(3), 219-26.

Deaf or Hard of Hearing

Adams, J. W., & Tidwell, R. (1989). An instructional guide for reducing the stress of hearing parents of hearing-impaired children. American Annals of the Deaf, 134(5), 323-328.

Hindley, P. (1997). Psychiatric Aspects of Hearing Impairments. Journal of Child Psychology and Psychiatry and Allied Disciplines, 38(1), 101-117.

Hintermair, M. (2000). Children who are hearing impaired with additional disabilities and related aspects of parental stress. Exceptional Children, 66(3), 327-332.

Hintermair, M. (2000). Hearing impairment, social networks, and coping: The need for families with hearing-impaired children to relate to other parents and to hearing-impaired adults. American Annals of the Deaf, 145(1), 41-53.

Hintermair, M. (2006). Parental Resources, Parental Stress, and Socioemotional Development of Deaf and Hard of Hearing Children. J. Deaf Stud. Deaf Educ., 11(4), 493-513. doi: 10.1093/deafed/enl005.

Horsch, U., Weber, C., Bertram, B., & Detrois, P. (1997). Stress experienced by parents of children with cochlear implants compared with parents of deaf children and hearing children. American Journal of Otology, 18(6 Suppl), 161-163.

Koester, L. S., & Meadow-Orlans, K. P. (1999). Responses to interactive stress: infants who are deaf or hearing. American Annals of the Deaf (Silver Spring, MD), 144(5), 395-403.

Kushalnagar, P., Krull, K., Hannay, J., Mehta, P., Caudle, S., & Oghalai, J. (2007). Intelligence, parental depression, and behavior adaptability in deaf children being considered for cochlear implantation. Journal of Deaf Studies and Deaf Education, 12(3), 335-349.

Lederberg, A. R., & Golbach, T. (2002). Parenting stress and social support in hearing mothers of deaf and hearing children: a longitudinal study. Journal of Deaf Studies and Deaf Education, 7(4), 330-345.

Macturk, R. H., Meadoworlans, K. P., Koester, L. S., & Spencer, P. E. (1993). Social Support, Motivation, Language, and Interaction - A Longitudinal-Study of Mothers and Deaf Infants. American Annals of the Deaf, 138(1), 19-25.

Meadow-Orlans, K. P. (1994). Stress, support, and deafness: Perceptions of infants' mothers and fathers. Journal of Early Intervention, 18(1), 91-102.

Meadow-Orlans, K. P. (1995). Sources of stress for mothers and fathers of deaf and hard of hearing infants. American Annals of the Deaf, 140(4), 352-7.

Meadow-Orlans, K. P., Smith-Gray, S., & Dyssegaard, B. (1995). Infants who are deaf or hard of hearing, with and without physical/cognitive disabilities. American Annals of the Deaf, 140(3), 279-86.

Vohr, B. R., Jodoin-Krauzyk, J., Tucker, R., Johnson, M. J., Topol, D., & Ahlgren, M. (2008). Results of newborn screening for hearing loss: effects on the family in the first 2 years of life. Archives of Pediatrics & Adolescent Medicine, 162(3), 205-211.

Vohr, B., Jodoin-Krauzyk, J., Tucker, R., Johnson, M. J., Topol, D., & Ahlgren, M. (2008). Early language outcomes of early-identified infants with permanent hearing loss at 12 to 16 months of age. Pediatrics, 122(3), 535-544. doi: 10.1542/peds.2007-2028.

Weisel, A., Most, T., & Michael, R. (2007). Mothers' Stress and Expectations as a Function of Time Since Child's Cochlear Implantation. J. Deaf Stud. Deaf Educ., 12(1), 55-64. doi: 10.1093/deafed/enl020.

Speech Disorders

Eiserman, W. D., McCoun, M., & Escobar, C. M. (1990). A cost-effectiveness analysis to two alternative program models for serving speech-disordered preschoolers. Journal of Early Intervention, 14, 297-317.

Eiserman, W. D., Weber, C., & McCoun, M. (1992). 2 Alternative Program Models for Serving Speech-Disordered Preschoolers - A 2nd Year Follow-Up. Journal of Communication Disorders, 25(2-3), 77-106.

Eiserman, W. D., Weber, C., & McCoun, M. (1995). Parent and Professional Roles in Early Intervention - A Longitudinal Comparison of the Effects of 2 Intervention Configurations. Journal of Special Education, 29(1), 20-44.

Girolametto, L., & Tannock, R. (1994). Correlates of Directiveness in the Interactions of Fathers and Mothers of Children with Developmental Delays. Journal of Speech and Hearing Research, 37(5), 1178-1191.

Girolametto, L. (1995). Reflection on the Origins of Directiveness - Implications for Intervention. Journal of Early Intervention, 19(2), 104-106.

Sarimski, K. (1997). Communication, Social-Emotional Development and Parenting Stress in Cornelia-de-Lange-Syndrome. Journal of Intellectual Disability Research, 41(FEB), 70-75.

Shelton, T. L., & Barkley, R. A. (1994). Critical Issues in the Assessment of Attention-Deficit Disorders in Children. Topics in Language Disorders, 14(4), 26-41.

Thomas, J. M., Guskin, K. A., & Klass, C. S. (1997). Early Development Program - Collaborative Structures and Processes. Infant Mental Health Journal, 18(2), 198-208.

Language Development

Bryan, T., Burstein, L., Chao, P., & Ergul, C. (2006). The relationship between health status, language development, and behavior in young children. Physical Disabilities: Education and Related Services, 24(2), 7-19.

Byrne, J. M., Dewolfe, N. A., & Bawden, H. N. (1998). Assessment of Attention-Deficit Hyperactivity Disorder in Preschoolers. Child Neuropsychology, 4(1), 49-66.

Caulfield, M. B., Fischel, J. E., DeBaryshe, B. D., & Whitehurst, G. J. (1989). Behavioral correlates of developmental expressive language disorder. Journal of Abnormal Child Psychology, 17(2), 187-201.

Chaffee, C. A., Cunningham, C. E., & Secord, G. (1991). The influence of parenting stress and child behavior problems on parental. Journal of Abnormal Child Psychology, 19(1), 65-74.

Feldman, M. A., & Waltonallen, N. (1997). Effects of Maternal Mental-Retardation and Poverty on Intellectual, Academic, and Behavioral Status of School-Age-Children. American Journal on Mental Retardation, 101(4), 352-364.

Fey, M. E., Warren, S. F., Brady, N., Finestack, L. H., Bredin-Oja, S. L., Fairchild, M., et al. (2006). Early Effects of Responsivity Education/Prelinguistic Milieu Teaching for Children With Developmental Delays and Their Parents. Journal of Speech, Language & Hearing Research, 49(3), 526-547. doi: 10.1044/1092-4388(2006/039).

Fischel, J. E., Whitehurst, G. J., Caulfield, M. B., & DeBaryshe, B. (1989). Language growth in children with expressive language delay. Pediatrics, 83(2), 218-27.

Girolametto, L., & Tannock, R. (1994). Correlates of Directiveness in the Interactions of Fathers and Mothers of Children with Developmental Delays. Journal of Speech and Hearing Research, 37(5), 1178-1191.

Golombok, S., Olivennes, F., Ramogida, C., Rust, J., & Freeman, T. (2007). Parenting and the psychological development of a representative sample of triplets conceived by assisted reproduction. Human Reproduction (Oxford, England), 22(11), 2896-2902. doi: 10.1093/humrep/dem260.

Macturk, R. H., Meadoworlans, K. P., Koester, L. S., & Spencer, P. E. (1993). Social Support, Motivation, Language, and Interaction - A Longitudinal-Study of Mothers and Deaf Infants. American Annals of the Deaf, 138(1), 19-25.

Nekkebroeck, J., Bonduelle, M., Desmyttere, S., Van den Broeck, W., & Ponjaert-Kristoffersen, I. (2008). Socio-emotional and language development of 2-year-old

children born after PGD/PGS, and parental well-being. Hum. Reprod., den179. doi: 10.1093/humrep/den179.

Noel, M., Peterson, C., & Jesso, B. (2008). The relationship of parenting stress and child temperament to language development among economically disadvantaged preschoolers. Journal of Child Language, 35(4), 823-843. doi: 10.1017/S0305000908008805.

Pan, B. A., Rowe, M. L., Singer, J. D., & Snow, C. E. (2005). Maternal correlates of growth in toddler vocabulary production in low-income families. Child Development, 76(4), 763-782. doi: 10.1111/j.1467-8624.2005.00876.x.

Paradise, J. L., Feldman, H. M., Colborn, D. K., Campbell, T. F., Dollaghan, C. A., Rockette, H. E., Janosky, J. E., Kurs-Lasky, M., Bernard, B. S., & Smith, C. G. (1999). Parental stress and parent-rated child behavior in relation to otitis media in the first three years of life. Pediatrics, 104(6), 1264-73.

Shelton, T. L., & Barkley, R. A. (1994). Critical Issues in the Assessment of Attention-Deficit Disorders in Children. Topics in Language Disorders, 14(4), 26-41.

Tannock, R., Girolametto, L., & Siegel, L. S. (1992). Language intervention with children who have developmental delays: effects of an interactive approach. American Journal of Mental Retardation, 97(2), 145-60.

Vohr, B., Jodoin-Krauzyk, J., Tucker, R., Johnson, M. J., Topol, D., & Ahlgren, M. (2008). Early language outcomes of early-identified infants with permanent hearing loss at 12 to 16 months of age. Pediatrics, 122(3), 535-544. doi: 10.1542/peds.2007-2028.

Conduct Disorder/ODD

Acton, R. G., & During, S. M. (1992). Preliminary results of aggression management training for aggressive. Journal of Interpersonal Violence, 7(3), 410-417.

August, G. J., Lee, S. S., Bloomquist, M. L., Realmuto, G. M., & Hektner, J. M. (2004). Maintenance Effects of an Evidence-Based Prevention Innovation for Aggressive Children Living in Culturally Diverse Urban Neighborhoods: The Early Risers Effectiveness Study. Journal of Emotional & Behavioral Disorders, 12(4), 194-205. doi: Article.

August, G. J., Realmuto, G. M., Hektner, J. M., & Bloomquist, M. L. (2001). An integrated components preventive intervention for aggressive elementary school children: the early risers program. Journal of Consulting and Clinical Psychology, 69(4), 614-626.

Bagley, C., & Mallick, K. (1997). Temperament, CNS Problems and Maternal Stressors - Interactive Predictors of Conduct Disorder in 9-Yr-Olds. Perceptual and Motor Skills, 84(2), 617-618.

Barkley, R. A., McMurray, M. B., Edelbrock, C. S., & Robbins, K. (1989). The response of aggressive and nonaggressive ADHD children to two doses of Methylphenidate. Journal of the American Academy of Child & Adolescent Psychiatry, 28(6), 873-881.

Brotman, L. M., Klein, R. G., Kamboukos, D., Brown, E. J., Coard, S. I., & Sosinsky, L. S. (2003). Preventive intervention for urban, low-income preschoolers at familial risk for conduct problems: A randomized pilot study. Journal of Clinical Child and Adolescent Psychology, 32(2), 246–257.

Calzada, E. J., Eyberg, S. M., Rich, B., & Querido, J. G. (2004). Parenting disruptive preschoolers: Experiences of mothers and fathers. Journal of Abnormal Child Psychology, 32(2), 203–213.

Capage, L. C., Bennett, G., & McNeil, C. B. (2001). A comparison between African American and Caucasian children referred for treatment of disruptive behavior disorders. Child & Family Behavior Therapy, 23(1), 1-14.

Chisholm, K. (1998). A 3 Year Follow-Up of Attachment and Indiscriminate Friendliness in Children Adopted from Romanian Orphanages. Child Development, 69(4), 1092-1106.

Costin, J., & Chambers, S. M. (2007). Parent management training as a treatment for children with oppositional defiant disorder referred to a mental health clinic. Clinical Child Psychology and Psychiatry, 12(4), 511-524.

Dombrowski, S. C., Timmer, S. G., Blacker, D. M., & Urquiza, A. J. (2005). A positive behavioural intervention for toddlers: parent-child attunement therapy. Child Abuse Review, 14(2), 132-151.

Donenberg, G., & Baker, B. L. (1993). The Impact of Young-Children with Externalizing Behaviors on Their Families. Journal of Abnormal Child Psychology, 21(2), 179-198.

Dukewich, T. L., Borkowski, J. G., & Whitman, T. L. (1996). Adolescent Mothers and Child-Abuse Potential - An Evaluation of Risk-Factors. Child Abuse & Neglect, 20(11), 1031-1047.

Edens, J. F., Cavell, T. A., & Hughes, J. N. (1999). The Self-Systems of Aggressive-Children - A Cluster-Analytic Investigation. Journal of Child Psychology and Psychiatry and Allied Disciplines, 40(3), 441-453.

Ethier, L. S., & Lafreniere, P. J. (1993). Single-Parent Maternal Stress with Respect to Preschooler Aggression. International Journal of Psychology, 28(3), 273-289.

Ethier, L. S., & Lafreniere, P. J. (1993). The relationship between maternal stress and preschool children's aggressiveness in single-parent families. International Journal of Psychology, 28(3), 273-289.

Eyberg, S. M. B. S. R. A. J. (1995). Parent-child interaction therapy: A psychosocial model for the treatment of young children with conduct problem behavior and their families. Psychopharmacology Bulletin, 31(1), 83-91.

Greene, R. W., Ablon, J. S., Goring, J. C., Raezer-Blakely, L., Markey, J., Monuteaux, M. C., et al. (2004). Effectiveness of collaborative problem solving in affectively dysregulated children with oppositional-defiant disorder: initial findings. Journal of Consulting and Clinical Psychology, 72(6), 1157-1164. doi: 10.1037/0022-006X.72.6.1157

Hughes, J. N., Cavell, T. A., & Grossman, P. B. (1997). A Positive View of Self - Risk or Protection for Aggressive-Children. Development and Psychopathology, 9(1), 75-94.

Hutchings, J., Bywater, T., Daley, D., Gardner, F., Whitaker, C., Jones, K.,... Edwards, R. T. (2007). Parenting intervention in Sure Start services for children at risk of developing conduct disorder: pragmatic randomized controlled trial. British Medical Journal, doi:10.1136/bmj.39126.620799.55

Hutchings, J., Lane, E., & Kelly, J. (2004). Comparison of two treatments for children with severely disruptive behaviors: A four-year follow-up. Behavioural and Cognitive Psychotherapy, 32(1), 15-30. doi: 10.1017/S1352465804001018.

Kazdin, A. E. (1995). Child, Parent and Family Dysfunction as Predictors of Outcome in Cognitive-Behavioral Treatment of Antisocial Children. Behaviour Research and Therapy, 33(3), 271-281.

Kazdin, A. E., & Crowley, M. J. (1997). Moderators of Treatment Outcome in Cognitively Based Treatment of Antisocial Children. Cognitive Therapy and Research, 21(2), 185-207.

Kazdin, A. E., & Wassell, G. (1998). Treatment Completion and Therapeutic Change Among Children Referred for Outpatient Therapy. Professional Psychology-Research and Practice, 29(4), 332-340.

Kazdin, A. E., Holland, L., & Crowley, M. (1997). Family Experience of Barriers to Treatment and Premature Termination from Child Therapy. Journal of Consulting and Clinical Psychology, 65(3), 453-463.

Kazdin, A. E., Mazurick, J. L., & Siegel, T. C. (1994). Treatment Outcome Among Children with Externalizing Disorder Who Terminate Prematurely Versus Those Who Complete Psychotherapy. Journal of the American Academy of Child and Adolescent Psychiatry, 33(4), 549-557.

Lavigne, J. V., LeBailly, S. A., Gouze, K. R., Cicchetti, C., Jessup, B. W., Arend, R., et al. (2008). Predictor and Moderator Effects in the Treatment of Oppositional Defiant Disorder in Pediatric Primary Care. Journal of Pediatric Psychology, 33(5), 462.

Lumley, V. A., McNeil, C. B., Herschell, A. D., & Bahl, A. B. (2002). An examination of gender differences among young children with disruptive behavior disorders. Child Study Journal, 32(2), 89-100.

McNeil, C. B., Capage, I. C., Bahl, A., & Blanc, H. (1999). Importance of early intervention for disruptive behavior problems: Comparison of treatment and waitlist-control groups. Early Education & Development, 10(4), 445-454.

Nixon, R. D. V., Sweeney, L., Erickson, D. B., & Touyz, S. W. (2004). Parent-child interaction therapy: one- and two-year follow-up of standard and abbreviated treatments for oppositional preschoolers. Journal of Abnormal Child Psychology, 32(3), 263-271.

Quinn, M., Carr, A., Carroll, L., & O Sullivan, D. (2006). An evaluation of the Parents Plus Programme for pre-school children with conduct problems: A comparison of those with and without developmental disabilities. Irish Journal of Psychology, 27(3/4), 168-182.

Realmuto, G. M., August, G. J., & Egan, E. A. (2004). Testing the goodness-of-fit of a multifaceted preventive intervention for children at risk for conduct disorder. Canadian Journal of Psychiatry. Revue Canadienne De Psychiatrie, 49(11), 743-752.

Ross, C. N., Blanc, H. M., McNeil, C. B., Eyberg, S. M., & Hembree-Kigin, T. L. (1998). Parenting stress in mothers of young children with oppositional defiant disorder and other severe behavior problems. Child Study Journal, 28(2), 93-110.

Schuhmann, E. M., Foote, R. C., Eyberg, S. M., Boggs, S. R., & Algina, J. (1998). Efficacy of Parent-Child Interaction Therapy - Interim-Report of a Randomized Trial with Short-Term Maintenance. Journal of Clinical Child Psychology, 27(1), 34-45.

Shelton, T. L., Barkley, R. A., Crosswait, C., Moorehouse, M., Fletcher, K., Barrett, S., Jenkins, L., & Metevia, L. (1998). Psychiatric and Psychological Morbidity as a Function of Adaptive Disability in Preschool-Children with Aggressive and Hyperactive-Impulsive-Inattentive Behavior. Journal of Abnormal Child Psychology, 26(6), 475-494.

Sokol, D. K., Ferguson, C. F., Pitcher, G. A., Huster, G. A., Fitzhughbell, K., & Luerssen, T. G. (1996). Behavioral-Adjustment and Parental Stress Associated with Closed-Head Injury in Children. Brain Injury, 10(6), 439-451.

Teti, D. M., Nakagawa, M., Das, R., & Wirth, O. (1991). Security of attachment between preschoolers and their mothers: Relations among social interaction, parenting stress, and mother's sorts of the Attachment Q-Set. Developmental Psychology, 27(3), 44 0-447.

Wall, J. E., & Holden, E. W. (1994). Aggressive, Assertive, and Submissive Behaviors in Disadvantaged, Inner-City Preschool-Children. Journal of Clinical Child Psychology, 23(4), 382-390.

Webster-Stratton, C., & Hammond, M. (1997). Treating Children with Early-Onset Conduct Problems - A Comparison of Child and Parent Training Interventions. Journal of Consulting and Clinical Psychology, 65(1), 93-109.

Cross-Cultural Studies

Barnes, J., Sutcliffe, A. G., Kristoffersen, I., Loft, A., Wnnerholm, U., Tarlatzis, B. C., … Bonduelle, M. (2004). The influence of assisted reproduction on family functioning and children's socio-emotional development: results from a European study. Human Reproduction, 19(6), 1480-1487.

Butcher, P. R., Wind, T., & Bouma, A. (2008). Parenting stress in mothers and fathers of a child with a hemiparesis: sources of stress, intervening factors and long-term expressions of stress. Child: Care, Health & Development, 34(4), 530-541. doi: 10.1111/j.1365-2214.2008.00842.x.

Bywater, T., Hutchings, J., Daley, D., Whitaker, C., Yeo, S. T., Jones, K., et al. (2009). Long-term effectiveness of a parenting intervention for children at risk of developing conduct disorder. The British Journal of Psychiatry: The Journal of Mental Science, 195(4), 318-324. doi: 10.1192/bjp.bp.108.056531

Calam, R., Bolton, C., & Roberts, J. (2002). Maternal expressed emotion, attributions and depression and entry into therapy for children with behaviour problems. British Journal of Clinical Psychology, 41(2), 213216.

Colver, A. (2006). Study protocol: SPARCLE -- a multi-centre European study of the relationship of environment to participation and quality of life in children with cerebral palsy. BMC Public Health, 6, 105-110.

Drummond, J., McDonald, L., MacKenzie-Keating, S., & Fleming, D. (2004). Types of support accessed by families of young children with disabilities living in Alberta. Developmental Disabilities Bulletin, 32(1), 1-27.

Edelson, M., Hokoda, A., & Ramos-Lira, L. (2007). Differences in Effects of Domestic Violence Between Latina and Non-Latina Women. Journal of Family Violence, 22(1), 1-10. doi: 10.1007/s10896-006-9051-1.

El-Kamary, S. S., Higman, S. M., Fuddy, L., McFarlane, E., Sia, C., & Duggan, A. K. (2004). Hawaii's healthy start home visiting program: determinants and impact of rapid repeat birth. Pediatrics, 114(3), e317-326. doi: 10.1542/peds.2004-0618.

Evangelou, M., Brooks, G., & Smith, S. (2007). The Birth to School Study: evidence on the effectiveness of PEEP, an early intervention for children at risk of educational under-achievement. Oxford Review of Education, 33(5), 581-609. doi: 10.1080/03054980701476477.

African

Potterton, J., Stewart, A., & Cooper, P. (2007). Parenting stress of caregivers of young children who are HIV positive. African Journal of Psychiatry, 10(4), 210-214.

African American

Belanger, K., Copeland, S., & Cheung, M. (2008). The Role of Faith in Adoption: Achieving Positive Adoption Outcomes for African American Children. Child Welfare, 87(2), 99. doi: Article.

Bendell, D., Stone, W. L., Field, T. M., & Goldstein, S. (1989). Children's effects on parenting stress in a low income, minority population. Topics in Early Childhood Special Education, 8(4), 58-71.

Bhavnagri, N. P. (1999). Low income African American mothers' parenting stress and instructional strategies to promote peer relationships in preschool children. Early Education & Development, 10(4), 551-571.

Black, M. M., Hutcheson, J. J., Dubowitz, H., Starr, R. H., & Berensonhoward, J. (1996). The Roots of Competence - Mother-Child Interaction Among Low-Income, Urban, African-American Families. Journal of Applied Developmental Psychology, 17(3), 367-391.

Black, M. M., Nair, P., Kight, C., Wachtel, R., Roby, P., & Schuler, M. (1994). Parenting and early development among children of drug-abusing women: effects of home intervention. Pediatrics, 94(4 Pt 1), 440-8.

Burchinal, M., Roberts, J. E., Zeisel, S. A., Hennon, E. A., & Hooper, S. (2006). Social risk and protective child, parenting, and child care factors in early elementary school years. Parenting: Science and Practice, 6(1), 79. doi: 10.1207/s15327922par0601_4

Button, S., Pianta, R. C., & Marvin, R. S. (2001). Partner support and maternal stress in families raising young children with cerebral palsy. Journal of Developmental & Physical Disabilities, 13(1), 61-81.

Cain, D. S., & Combs-Orme, T. (2005). Family structure effects on parenting stress and practices in the African American family. Journal of Sociology and Social Welfare, 32(2), 19-40.

Capage, L. C., Bennett, G., & McNeil, C. B. (2001). A comparison between African American and Caucasian children referred for treatment of disruptive behavior disorders. Child & Family Behavior Therapy, 23(1), 1-14.

Carothers, S. S., Borkowski, J. G., & Whitman, T. L. (2006). Children of Adolescent Mothers: Exposure to Negative Life Events and the Role of Social Supports on Their Socioemotional Adjustment. Journal of Youth and Adolescence, 35(5), 822-832. doi: 10.1007/s10964-006-9096-8.

Cole, S. A. (2002). Security of Attachment of Infants in Foster Care. In Proceedings of the... National Symposium on Doctoral Research in Social Work (p. 12). Presented at the National Symposium on Doctoral Research in Social Work, Columbus, OH: Ohio State University.

Dalla, R. L., & Gamble, W. C. (1997). Exploring Factors Related to Parenting Competence Among Navajo Teenage Mothers - Dual Techniques of Inquiry. Family Relations, 46(2), 113-121.

Francis-Williams, N.(2005). Evaluation of a faith-based socioemotional support program for parents of African American youth with antisocial behaviors. (Unpublished doctoral dissertation). Nova Southeastern University, Florida.

Gonchar, N. (1995). College-student mothers and on-site child care: luxury or necessity? Social Work in Education, 17(4), 226-34.

Hooper, S. R., Burchinal, M. R., Roberts, J. E., Zeisel, S., & Neebe, E. C. (1998). Social and Family Risk-Factors for Infant Development at One-Year - An Application of the Cumulative Risk Model. Journal of Applied Developmental Psychology, 19(1), 85-96.

Hutcheson, J. J., & Black, M. M. (1996). Psychometric properties of the Parenting Stress Index in a sample of low-income African-American mothers of infants and toddlers. Early Education & Development, 7(4), 381-400.

Ispa, J. M., Fine, M. A., Halgunseth, L. C., Harper, S., Robinson, J., Boyce, L., et al. (2004). Maternal Intrusiveness, Maternal Warmth, and Mother–Toddler Relationship Outcomes: Variations Across Low-Income Ethnic and Acculturation Groups. Child Development, 75(6), 1613-1631. doi: 10.1111/j.1467-8624.2004.00806.x.

Jackson, A. P. (1999). The Effects of Nonresident Father Involvement on Single Black Mothers and Their Young-Children. Social Work, 44(2), 156-166.

Jackson, A. P., Gyamfi, P., Brooksgunn, J., & Blake, M. (1998). Employment Status, Psychological Well-Being, Social Support, and Physical Discipline Practices of Single Black Mothers. Journal of Marriage and the Family, 60(4), 894-902.

Kazdin, A. E., Mazurick, J. L., & Bass, D. (1993). Risk for Attrition in Treatment of Antisocial Children and Families. Journal of Clinical Child Psychology, 22(1), 2-16.

Kelley, S. J. (1998). Stress and coping behaviors of substance-abusing mothers. Journal of the Society of Pediatric Nurses, 3(3), 103-10.

Minter, K. R., Roberts, J. E., Hooper, S. R., Burchinal, M. R., & Zeisel, S. A. (2001). Early childhood otitis media in relation to children's attention-related behavior in the first six years of life. Pediatrics, 107(5), 1037-1042.

Mitchell, M. D., Hargrove, G. L., Collins, M. H., Thompson, M. P., Reddick, T. L., & Kaslow, N. J. (2006). Coping variables that mediate the relation between intimate partner violence and mental health outcomes among low-income, African American women. Journal of Clinical Psychology, 62(12), 1503-1520. doi: 10.1002/jclp.20305.

Mowbray, C. T., Bybee, D., Hollingsworth, L., Goodkind, S., & Oyserman, D. (2005). Living Arrangements and Social Support: Effects on the Well-Being of Mothers with Mental Illness. Social Work Research, 29(1), 41. doi: Article.

Nitz, K., Ketterlinus, R. D., & Brandt, L. J. (1995). The role of stress, social support, and family environment in adolescent mothers' parenting. Journal of Adolescent Research, 10(3), 358-382.

Oyserman, D., Bybee, D., Mowbray, C. T., & MacFarlane, P. (2002). Positive Parenting among African American Mothers with a Serious Illness. Journal of Marriage and Family, 64(1), 65-77.

Rhodes, J. E., Ebert, L., & Fischer. (1992). Natural mentors: An overlooked resource in the social networks of young, African-American mothers. American Journal of Community Psychology, 20(4), 445-461.

Rhodes, J. E., Fischer, K., Ebert, L., & Meyers, A. B. (1993). Patterns of Service Utilization Among Pregnant and Parenting African-American Adolescents. Psychology of Women Quarterly, 17(3), 257-274.

Roberts, A. C., & Nishimoto, R. (2006). Barriers to engaging and retaining African-American post-partum women in drug treatment. Journal of Drug Issues, 36(1), 53. doi: Article.

Saylor, C. F., Casto, G., & Huntington, L. (1996). Predictors of Developmental Outcomes for Medically Fragile Early Intervention Participants. Journal of Pediatric Psychology, 21(6), 869-887.

Singh, D. K. (2003). Families of Head Start Children: A Research Connection. Journal of Instructional Psychology, 30(1), 77. doi: Article.

Uno, D., Florsheim, P., & Uchino, B. N. (1998). Psychosocial Mechanisms Underlying Quality of Parenting Among Mexican-American and White Adolescent Mothers. Journal of Youth and Adolescence, 27(5), 585-605.

Van Hasselt, V. B., Hersen, M., Null, J. A., Ammerman, R. T., Bukstein, O. G., Mcgillivray, J., & Hunter, A. (1993). Drug-Abuse Prevention for High-Risk African-American Children and Their Families - A Review and Model Program. Addictive Behaviors, 18(2), 213-234.

Walker, C. D. (1999). Stress in parents of children with ADHD vs. depression: A multicultural analysis. Dissertation Abstracts International: Section B: the Sciences & Engineering, 59(7-B).

Wolfe, R. B., & Hirsch, B. J. (2003). Outcomes of parent education programs based on reevaluation counseling. Journal of Child & Family Studies, 12(1), 61-76.

Australian

Dawe, S., Harnett, P. H., Rendalls, V., & Staiger, P. (2003). Improving family functioning and child outcome in methadone maintained families: the Parents Under Pressure programme. Drug and Alcohol Review, 22(3), 299–307.

Esdaile, S. A., & Greenwood, K. M. (2003). A comparison of mother's and father's experience of parenting stress and attributions for parent-child interaction outcomes. Occupational Therapy International, 10(2), 115. doi: Article.

Kemp, L., Harris, E., McMahon, C., Matthey, S., Vimpani, G., Anderson, T., et al. (2008). Miller Early Childhood Sustained Home-visiting (MECSH) trial: design, method and sample description. BMC Public Health, 8, 1-12. doi: 10.1186/1471-2458-8-424.

Phillips, J., Morgan, S., Cawthorne, K., & Barnett, B. (2008). Pilot evaluation of parent-child interaction therapy delivered in an Australian community early childhood clinic setting. Australian & New Zealand Journal of Psychiatry, 42(8), 712-719. doi: 10.1080/00048670802206320.

Wong, F., & Poon, A. (2010). Cognitive behavioural group treatment for Chinese parents with children with developmental disabilities in Melbourne, Australia: An efficacy study, Australian and New Zealand Journal of Psychiatry, 44, 742-749.

Chinese

Chan, Y. C. (1994). Parenting stress and social support of mothers who physically abuse their children in Hong Kong. Child Abuse & Neglect, 18(3), 261-9.

Chang, S. S. Y., Ng, C. F. N., & Wong, S. N. (2002). Behavioural problems in children and parenting stress associated with primary nocturnal enuresis in Hong Kong. Acta Paediatrica, 91(4), 475-479.

Huang, W., Rubin, S. E., & Zhang, F. (1998). Correlates of stress level in Chinese mothers of a child with mental retardation. International Journal of Rehabilitation Research, 21(2), 237-40.

Lee, M., Chen, Y., Wang, H., & Chen, D. (2007). Parenting stress and related factors in parents of children with Tourette Syndrome. Journal of Nursing Research, 15(3), 165-174.

Leung, S. S. L., Leung, C., & Chan, R. (2007). Perceived child behaviour problems, parenting stress, and marital satisfaction: comparison of new arrival and local parents of preschool children in Hong Kong. Hong Kong Medical Journal = Xianggang Yi Xue Za Zhi / Hong Kong Academy of Medicine, 13(5), 364-371.

Mak, W. W. S., Ho, A .H. Y., & Law, R. W. (2007). Sense of coherence, parenting attitudes and stress among mothers of children with autism in Hong Kong. Journal of Applied Research in Intellectual Disabilities, 20(2), 157-167.

Ong, L. C., Afifah, I., Sofiah, A., & Lye, M. S. (1998). Parenting stress among mothers of Malaysian children with cerebral palsy: predictors of child- and parent-related stress. Annals of Tropical Paediatrics, 18(4), 301-7.

Ooi, Y. P., Lam, C. M., Sung, M., Tan, W. T. S., Goh, T. J., Fung, D. S. S., et al. (2008). Effects of cognitive-behavioural therapy on anxiety for children with high-functioning autistic spectrum disorders. Singapore Medical Journal, 49(3), 215-220.

Scott, B. S., Atkinson, L., Minton, H. L., & Bowman, T. (1997). Psychological Distress of Parents of Infants with Down-Syndrome. American Journal on Mental Retardation, 102(2), 161-171.

Tam, K.-k., Chan, Y.-c., & Wong, C.-k. M. (1994). Validation of the Parenting Stress Index among Chinese mothers in Hong Kong. Journal of Community Psychology, 22(3), 211-223.

Tsang, S. K. M., & Leung, C. (2005). Developing a database for evaluating the effectiveness of parent education and support programs: Results of a pilot study. Illinois Child Welfare Journal, 2(1-2), 77–89.

Tsang, S., Tsang, S., Chan, E., & Lee, C. (1992). Stress of parents with normal and special pre-school children: A comparison study. Bulletin of the Hong Kong Psychological Society, 28, 63-79.

Wang, H., & Jong, Y. (2004). Parental Stress and Related Factors in Parents of Children with Cerebral Palsy. The Kaohsiung Journal of Medical Sciences, 20(7), 334-340. doi: 10.1016/S1607-551X(09)70167-6.

Wong, F., & Poon, A. (2010). Cognitive behavioural group treatment for Chinese parents with children with developmental disabilities in Melbourne, Australia: An efficacy study, Australian and New Zealand Journal of Psychiatry, 44, 742-749.

Yeh, C., Chen, M., & Chuang, H. (2001). The Chinese version of the Parenting Stress Index: a psychometric study. Acta Paediatrica, 90(12), 1470-1477.

Dutch

Colpin, H., & Soenen, S. (2002). Parenting and psychosocial development of IVF children: a follow-up study. Human Reproduction, 17(4), 1116-1123.

Deković, M., Janssens, J. M., & van As, N. M. (2003). Family Predictors of Antisocial Behavior in Adolescence. Family Process, 42(2), 223. doi: Article.

Embregts, P., Grimbel du Bois, M., & Graef, N. (2010). Behavior problems in children with mild intellectual disabilities: An initial step towards prevention. Research in Developmental Disabilities, 31, 1398-1403.

Grietens, H., Haene, L., & Uyttebroek, K. (2007). Cross-cultural Validation of the Child Abuse Potential Inventory in Belgium (Flanders): Relations with Demographic Characteristics and Parenting Problems. Journal of Family Violence, 22(4), 223-229. doi: 10.1007/s10896-007-9074-2.

Janssens, K. A., Oldehinkel, A. J., & Rosmalen, J. G. (2009). Parental overprotection predicts the development of functional somatic symptoms in young adolescents. The Journal of Pediatrics, 154(6), 918-923.

Knoester, M., Helmerhorst, F. M., van der Westerlaken, L. A., Walther, F. J., & Veen, S. (2007). Matched follow-up study of 5 8-year old ICSI singletons: child behaviour, parenting stress and child (health-related) quality of life. Human Reproduction, 22(12), 3098-3107.

Lux, A., Kropf, S., Kleinemeier, E., Jürgensen, M., & Thyen, U. (2009). Clinical evaluation study of the German network of disorders of sex development (DSD)/intersexuality: study design, description of the study population, and data quality. BMC Public Health, 9, 1-17. doi: 10.1186/1471-2458-9-110.

Meijssen, D., Wolf, M., Koldewijn, K., van Wassenaer, A., Kok, J., & van Baar, A. (2010). Parenting stress in mothers after very preterm birth and the effect of the Infant Behavioural Assessment and Intervention Program. Child: care, health and development, 37, 195-202.

Nekkebroeck, J., Bonduelle, M., Desmyttere, S., Van den Broeck, W., & Ponjaert-Kristoffersen, I. (2008). Socio-emotional and language development of 2-year-old children born after PGD/PGS, and parental well-being. Hum. Reprod., den179. doi: 10.1093/humrep/den179.

Oord, S., Prins, P., Oosterlaan, J., & Emmelkamp, P. (2007). Does brief, clinically based, intensive multimodal behavior therapy enhance the effects of methylphenidate in

children with ADHD? European Child & Adolescent Psychiatry, 16(1), 48-57. doi: 10.1007/s00787-006-0574-z.

Rodenburg, R., Meijer, A. M., Deković, M., & Aldenkamp, A. P. (2007). Parents of children with enduring epilepsy: Predictors of parenting stress and parenting. Epilepsy and Behavior, 11(2), 197–207.

van der Pal, S. M., Maguire, C. M., le Cessie, S., van Zwieten, P., Veen, S., Wit, J., & Walther, F. (2008). Very pre-term infants' behaviour at 1 and 2 years of age and parental stress following basic developmental care. British Journal of Developmental Psychology, 26(1), 103-115.

Van Hiel, A., & De Clercq, B. (2009). Authoritarianism is good for you: Right-wing authoritarianism as a buffering factor for mental distress. European Journal of Personality, 23(1), 33-50. doi: 10.1002/per.702.

Vermaes, I. P. R., Janssens, J. M. A. M., Mullaart, R. A., Vinck, A., & Gerris, J. R. M. (2008). Parents' personality and parenting stress in families of children with spina bifida. Child: Care, Health & Development, 34(5),665-674.

Wolff, N., Darlington, A., Hunfeld, J., Verhulst, F., Jaddoe, V., Hofman, A., et al. (2009). Determinants of Somatic Complaints in 18-month-old Children: The Generation R Study. J. Pediatr. Psychol.. doi: 10.1093/jpepsy/jsp058

Wulffaert, J., Scholte, E. M., Dijkxhoorn, Y. M., Bergman, J. E., van Ravenswaaij-Arts, C. M., & van Berckelaer-Onnes, I. A. (2009). Parenting stress in CHARGE syndrome and the relationship with child characteristics. Journal of Developmental and Physical Disabilities, 21(4), 301-313.

Wulffaert, J., Scholte, E., & van Berckelaer-Onnes, I. (2010). Maternal parenting stress in families with a child with Angelman syndrome or Prader–Willi syndrome, Journal of Intellectual & Developmental Disability, 35, 165-174.

Finnish

Flykt, M., Lindblom, J., Punamaki, R., Poikkeus, P., Repokari, L., Unkila-Kallio, L., Vilska, S., Sinkkonen, J., Tiitinen, A., Almqvist, F., & Tulppala, M. (2009). Prenatal expectations in transition to parenthood: Former infertility and family dynamic considerations. Journal of Family Psychology, 23, 779-789.

Kinnunen, U., Geurts, S., & Mauno, S. (2004). Work-to-family conflict and its relationship with satisfaction and well-being: a one-year longitudinal study on gender differences. Work & Stress, 18(1), 1-22. doi: 10.1080/02678370410001682005.

Mäntymaa, M., Puura, K., Luoma, I., Salmelin, R. K., & Tamminen, T. (2006). Mother's early perception of her infant's difficult temperament, parenting stress and early mother-infant interaction. Nordic Journal of Psychiatry, 60(5), 379-386.

Tarkka, M. T., Paunonen, M., & Laippala, P. (1999). Factors Related to Successful Breast-Feeding by First-Time Mothers When the Child Is 3 Months Old. Journal of Advanced Nursing, 29(1), 113-118.

Virtanen, T., & Moilanen, I. (1991). Stress and coping in mothers of children with minimal brain dysfunction. Praxis der Kinderpsychologie und Kinderpsychiatrie, 40(7), 260-265.

French

Golombok, S., Olivennes, F., Ramogida, C., Rust, J., & Freeman, T. (2007). Parenting and the psychological development of a representative sample of triplets conceived by assisted reproduction. Human Reproduction (Oxford, England), 22(11), 2896-2902. doi: 10.1093/humrep/dem260.

French Canadian

Bigras, M., & Lafreniere, P. J. (1994). Influence of Psychosocial Risk, Marital Conflicts and Parental Stress on the Quality of Mother-Son and Mother-Daughter Interactions. Canadian Journal of Behavioural Science, 26(2), 280-297.

Bigras, M., Lafreniere, P., & Dumas, J. (1996). Discriminant validity of the parent and child scales of the parenting stress index. Early Education & Development, 7(2), 167-178.

Cameron, S. J., & Orr, R. (1989). Stress in families of school-aged children with delayed mental development. Canadian Journal of Rehabilitation, 2(3), 137-144.

Cameron, S., Dobson, L., & Day, D. (1991). Stress in parents of developmentally delayed and non-delayed preschool children. Canada's Mental Health, 39(1), 13-17.

Chisholm, K., Carter, M. C., Ames, E. W., & Morison, S. J. (1995). Attachment security and indiscriminately friendly behavior in children adopted from Romanian orphanages. Development & Psychopathology, 7(2), 283-294.

Drummond, J., Kysela, G. M., McDonald, L., Alexander, J., & Fleming, D. (1996). Risk and resiliency in two samples of Canadian families. Health & Canadian Society, 4(1), 117-51.

Emery, J., Paquette, D., & Bigras, M. (2008). Factors predicting attachment patterns in infants of adolescent mothers. Journal of Family Studies, 14(1), 65-90. doi: Article.

Ethier, L. S. (1992). Developmental factors related to stress in neglectful and abusive mothers. Apprentissage et Socialisation, 15(3), 222-236.

Ethier, L. S., & Lafreniere, P. J. (1993). The relationship between maternal stress and preschool children's aggressiveness in single-parent families. International Journal of Psychology, 28(3), 273-289.

Ethier, L. S., Lacharite, C., & Couture, G. (1995). Childhood adversity, parental stress, and depression of negligent mothers. Child Abuse & Neglect, 19(5), 619-32.

Gottlieb, L. N., & Feeley, N. (1996). The McGill Model of Nursing and children with a chronic condition: "who benefits, and why?". Canadian Journal of Nursing Research, 28(3), 29-48.

Harrison, M. J., & Magill-Evans, J. (1996). Mother and father interactions over the first year with term and preterm infants. Research in Nursing & Health, 19(6), 451-9.

Lévesque, S., Clément, M., & Chamberland, C. (2007). Factors Associated with Co-occurrence of Spousal and Parental Violence: Quebec Population Study. Journal of Family Violence, 22(8), 661-674. doi: 10.1007/s10896-007-9106-y.

Levin, R., & Banks, S. (1991). Stress in parents of children with epilepsy. Canadian Journal of Rehabilitation, 4(4), 229-38.

Mainemer, H., Gilman, L. C., & Ames, E. W. (1998). Parenting stress in families adopting children from Romanian orphanages. Journal of Family Issues, 19(2), 164-180.

Stapleton, S. R., Drummond, J., Kysela, G. M., McDonald, L., Alexander, J., & Fleming, D. (1996). Team-building: making collaborative practice work. Risk and resiliency in two samples of Canadian families. Journal of Nurse-Midwifery, 4(1), 117-51.

German

Lauth, G. W., Otte, T., & Heubeck, B. G. (2009). Effectiveness of a competence training programme for parents of socially disruptive children. Emotional & Behavioural Difficulties, 14(2), 117-126.

Willinger, U., Diendorfer-Radner, G., Willnauer, R., Jörgl, G., & Hager, V. (2005). Parenting stress and parental bonding. Behavioral Medicine, 31(2), 63-69.

Hispanic

Bendell, D., Goldberg, M. S., Urbano, M. T., & Urbano, R. C. (1987). Differential impact of parenting sick infants. Infant Mental Health Journal, 8(1), 28-36.

Dalla, R. L., & Gamble, W. C. (1997). Exploring Factors Related to Parenting Competence Among Navajo Teenage Mothers - Dual Techniques of Inquiry. Family Relations, 46(2), 113-121.

Dumas, J. E., Martinez, A., & Lafreniere, P. J. (1998). The Spanish Version of the Social Competence and Behavior Evaluation (Scbe) Preschool Edition - Translation and Field Testing. Hispanic Journal of Behavioral Sciences, 20(2), 255-269.

Grossman, J., & Shigaki, I. S. (1994). Investigation of Familial and School-Based Risk-Factors for Hispanic Head-Start Children. American Journal of Orthopsychiatry, 64(3), 456-467.

Kazdin, A. E. (1994). Family adversity, socioeconomic disadvantage, and parental stress: Contextual variables related to premature termination from child behavior therapy. Psicologia Conductual, 2(1), 5-21.

Meisels, S. J., & Liaw, F. R. (1993). Failure in Grade - Do Retained Students Catch-Up. Journal of Educational Research, 87(2), 69-77.

Nievar, M. A, Jacobson, A., & Dier, S. (2008, November). Home visiting for at-risk preschoolers: A successful model for Latino families. Paper presented at the Annual Meeting of the National Council on Family Relations, Little Rock, Arkansas.

Planos, R., Zayas, L. H., & Buschrossnagel, N. A. (1997). Mental-Health Factors and Teaching Behaviors Among Low-Income Hispanic Mothers. Families in Society-The Journal of Contemporary Human Services, 78(1), 4-12.

Simoni, J., M. (1993). Latina mothers’ help seeking at a school-based mutual support group. Journal of Community Psychology, 21, 188-199.

Solis, M. L., & Abidin, R. R. (1991). The Spanish version Parenting Stress Index: A psychometric study. Journal of Clinical Child Psychology, 20(4), 372-378.

Icelandic

Thome, M. (2003). Severe postpartum distress in Icelandic mothers with difficult infants: a follow-up study on their health care. Scandinavian Journal of Caring Sciences, 17(2), 104-112. doi: 10.1046/j.1471-6712.2003.00110.x.

Irish

Fitzgerald, M., Butler, B., & Kinsella, A. (1990). The burden on a family having a child with special needs. Irish Journal of Psychological Medicine, 7(2), 109-113.

Waldron, A., Tobin, G., & McQuaid, P. (2001). Mental health status of homeless children and their families. Irish Journal of Psychological Medicine, 18(1), 11-15.

Israeli

Feldman, R., Eidelman, A. I., & Rotenberg, N. (2004). Parenting Stress, Infant Emotion Regulation, Maternal Sensitivity, and the Cognitive Development of Triplets: A Model for Parent and Child Influences in a Unique Ecology. Child Development, 75(6), 1774-1791. doi: 10.1111/j.1467-8624.2004.00816.x.

Rimmerman, A., Turkel, L., & Crossman, R. (2003). Perception of child development, child-related stress and dyadic adjustment: pair analysis of married couples of young children with developmental disabilities. Journal of Intellectual & Developmental Disability, 28(2), 188. doi: Article.

Italian

Golombok, S., Brewaeys, A., Cook, R., Giavazzi, M. T., Guerra, D., Mantovani, A., Vanhall, E., Crosignani, P. G., & Dexeus, S. (1996). The European Study of Assisted Reproduction Families - Family Functioning and Child-Development. Human Reproduction, 11(10), 2324-2331.

Japanese

Ando, J., Nonaka, K., Ozaki, K., Sato, N., Fujisawa, K. K., Suzuki, K., et al. (2006). The Tokyo Twin Cohort Project: overview and initial findings. Twin Research and Human Genetics: The Official Journal of the International Society for Twin Studies, 9(6), 817-826. doi: 10.1375/183242706779462480.

Hanada, H., Honda, S., Tokumaru, T., & Hiroki, O. (2006). Association Between Mothers' Concern About Child Rearing and Their Parenting Stress. Acta Med Nagasaki Ensia, 51(4), 115-120.

Holaday, B., Turner-Henson, A., Kanematsu, Y., Krulik, T., & Wang, R. (1997). Stress in mothers of chronically ill children: a cross cultural study. Australian Paediatric Nurse, 6(1), 2-9.

Kazui, M., Muto, T., & Sonoda, N. (1996). The roles of marital quality and parenting stress in mother-preschooler relationships. (Japanese). Japanese Journal of Developmental Psychology, 7(1), 31-40.

Nakagawa, M., Teti, D. M., & Lamb, M. E. (1992). An ecological study of child-mother attachments among Japanese sojourners in the United States. Developmental Psychology, 28, 584-592.

Korean

Ha, E. H., Oh, K. J., & Kim, E. J. (1999). Depressive symptoms and family relationship of married women: Focused on parenting stress and marital dissatisfaction. Korean Journal of Clinical Psychology, 18(1), 79-93.

Malaysian

Ong, L. C., Chandran, V., & Boo, N. Y. (2001). Comparison of parenting stress between Malaysian mothers of four-year-old very low birthweight and normal birthweight children. Acta Paediatrica, 90(12), 1464-1469.

Mexican American

Domoto, P., Weinstein, P., Leroux, B., Koday, M., Ogura, S., & Iatridiroberson, I. (1994). White Spots Caries in Mexican-American Toddlers and Parental Preference for Various Strategies. Journal of Dentistry for Children, 61(5-6), 342-346.

Ispa, J. M., Fine, M. A., Halgunseth, L. C., Harper, S., Robinson, J., Boyce, L., et al. (2004). Maternal Intrusiveness, Maternal Warmth, and Mother–Toddler Relationship Outcomes: Variations Across Low-Income Ethnic and Acculturation Groups. Child Development, 75(6), 1613-1631. doi: 10.1111/j.1467-8624.2004.00806.x.

McCabe, K., & Yeh, M. (2009). Parent–Child Interaction Therapy for Mexican Americans: A Randomized Clinical Trial. Journal of Clinical Child & Adolescent Psychology, 38(5), 753. doi: 10.1080/15374410903103544

Uno, D., Florsheim, P., & Uchino, B. N. (1998). Psychosocial Mechanisms Underlying Quality of Parenting Among Mexican-American and White Adolescent Mothers. Journal of Youth and Adolescence, 27(5), 585-605.

Weinstein, P., Domoto, P., Wohlers, K., & Koday, M. (1992). Mexican-American Parents with Children at Risk for Baby Bottle Tooth-Decay - Pilot-Study at a Migrant Farmworkers Clinic. Journal of Dentistry for Children, 59(5), 376-383.

Native American

Dalla, R. L., & Gamble, W. C. (1997). Exploring Factors Related to Parenting Competence Among Navajo Teenage Mothers - Dual Techniques of Inquiry. Family Relations, 46(2), 113-121.

Probst, J., Jong-Yi Wang, Martin, A. B., Moore, C. G., Paul, B., & Samuels, M. E. (2008). Potentially Violent Disagreements and Parenting Stress Among American Indian/Alaska Native Families: Analysis Across Seven States.. Maternal & Child Health Journal, 12, 91-102. doi: 10.1007/s10995-008-0370-0.

Norwegian

Glavin, K., Smith, L., Sørum, R., & Ellefsen, B. (2010). Redesigned community postpartum care to prevent and treat postpartum depression in women - a one-year follow-up study. Journal of Clinical Nursing, 19, 3051-3062.

Olafsen, K. S., Rønning, J. A., Bredrup Dahl, L., Ulvund, S. E., Handegård, B. H., & Kaaresen, P. I.(2007). Infant responsiveness and maternal confidence in the neonatal period. Scandinavian Journal of Psychology, 48(6), 499-509.

Portuguese

Vieira Santos, S. (1994). Characteristics of stress in parents of children with chronic diseases and in parents of children with emotional problems. Analise Psicologica, 12(2-3).

Vieira Santos, S., Ataide, S., & Joao, P. (1996). Parental stress in parents of children with obesity and with chronic renal disease. Analise Psicologica, 14(2-3), 231-243.

Swedish

Dellve, L., Samuelsson, L., Tallborn, A., Fasth, A., & Hallberg, L. R. (2006). Stress and well-being among parents of children with rare diseases: a prospective intervention study. Journal of Advanced Nursing, 53(4), 392-402. doi: 10.1111/j.1365-2648.2006.03736.x.

Jackson, K., Ternestedt, B., Magnuson, A., & Schollin, J. (2007). Parental stress and toddler behaviour at age 18 months after pre-term birth. Acta Paediatrica, 96(2), 227-232. doi: 10.1111/j.1651-2227.2007.00015.x.

Ponjaert-Kristoffersen, I., Tjus, T., Nekkebroeck, J., Squires, J., Verté, D., Heimann, M., ... Wennerholm, U. B. (2004). Psychological follow-up study of 5-year-old ICSI children. Human Reproducation, 19(12), 2791-2797.

Thunström, M. (2002). Severe sleep problems in infancy associated with subsequent development of attention-deficit/hyperactivity disorder at 5.5 years of age. Acta Paediatrica, 91(5), 584. doi: Article.

Turkish

Mert, E., Hallioglu, O., & Ankarali Camdeviren, H. (2008). Turkish Version of the Parenting Stress Index Short Form: A Psychometric Study. Turkiye Klinikleri J Med Sci, 28, 291–296.

Vietnamese

Shin, J., Nhan, N. V., Crittenden, K. S., Hong, H. T. D., Flory, M., & Ladinsky, J. (2006). Parenting stress of mothers and fathers of young children with cognitive delays in Vietnam. Journal of Intellectual Disability Research, 50(10), 748-760. doi: 10.1111/j.1365-2788.2006.00840.x.

Depression

Ando, J., Nonaka, K., Ozaki, K., Sato, N., Fujisawa, K. K., Suzuki, K., et al. (2006). The Tokyo Twin Cohort Project: overview and initial findings. Twin Research and Human Genetics: The Official Journal of the International Society for Twin Studies, 9(6), 817-826. doi: 10.1375/183242706779462480.

Armstrong, K. L., Fraser, J. A., Dadds, M. R., & Morris, J. (2000). Promoting secure attachment, maternal mood and child health in a vulnerable population: a randomized controlled trial. Journal of Paediatrics & Child Health, 36(6), 555-62.

August, G. J., Realmuto, G. M., Hektner, J. M., & Bloomquist, M. L. (2001). An integrated components preventive intervention for aggressive elementary school children: the early risers program. Journal of Consulting and Clinical Psychology, 69(4), 614-626.

Baker, B. L., & Heller, T. L. (1996). Preschool-Children with Externalizing Behaviors - Experience of Fathers and Mothers. Journal of Abnormal Child Psychology, 24(4), 513-532.

Bech, P., Malt, U. F., Dencker, S. J., & Ahlfors, U. G. (1993). Scales for Assessment of Diagnosis and Severity of Mental-Disorders. Acta Psyciatrica Scandinavica, 87(S372).

Bigras, M., Lafreniere, P., & Dumas, J. (1996). Discriminant validity of the parent and child scales of the parenting stress index. Early Education & Development, 7(2), 167-178.

Blakeney, P., Moore, P., Broemeling, L., Hunt, R., Herndon, D. N., & Robson, M. (1993). Parental stress as a cause and effect of pediatric burn injury. Journal of Burn Care & Rehabilitation, 14(1), 73-9.

Breen, M. J., & Barkley, R. A. (1988). Child psychopathology and parenting stress in girls and boys having attention deficit disorder with hyperactivity. Journal of Pediatric Psychology, 13(2), 265-280.

Buist, A. (1998). Childhood Abuse, Parenting and Postpartum Depression. Australian and New Zealand Journal of Psychiatry, 32(4), 479-487.

Buist, A., & Janson, H. (1995). Effect of Exposure to Dothiepin and Northiaden in Breast-Milk on Child-Development. British Journal of Psychiatry, 167(SEP), 370-373.

Byrne, J. M., Dewolfe, N. A., & Bawden, H. N. (1998). Assessment of Attention-Deficit Hyperactivity Disorder in Preschoolers. Child Neuropsychology, 4(1), 49-66.

Calam, R., Bolton, C., & Roberts, J. (2002). Maternal expressed emotion, attributions and depression and entry into therapy for children with behaviour problems. British Journal of Clinical Psychology, 41(2), 213216.

Calzada, E. J., Eyberg, S. M., Rich, B., & Querido, J. G. (2004). Parenting disruptive preschoolers: Experiences of mothers and fathers. Journal of Abnormal Child Psychology, 32(2), 203–213.

Campbell, S. B. (1994). Hard-to-Manage Preschool Boys - Externalizing Behavior, Social Competence, and Family Context at 2-Year Follow-Up. Journal of Abnormal Child Psychology, 22(2), 147-166.

Campis, L. B., DeMaso, D. R., & Twente, A. W. (1995). The role of maternal factors in the adaptation of children with craniofacial disfigurement. Cleft Palate Craniofacial Journal, 32(1), 55-61.

Cappelli, M., Mcgrath, P. J., Daniels, T., Manion, I., & Schillinger, J. (1994). Marital Quality of Parents of Children with Spina-Bifida - A Case-Comparison Study. Journal of Developmental and Behavioral Pediatrics, 15(5), 320-326.

Chazan-Cohen, R., Ayoub, C., Pan, B. A., Roggman, L., Raikes, H., Mckelvey, L., et al. (2007). It takes time: Impacts of Early Head Start that lead to reductions in maternal depression two years later. Infant Mental Health Journal, 28(2), 151-170. doi: 10.1002/imhj.20127.

Chisholm, K. (1998). A 3 Year Follow-Up of Attachment and Indiscriminate Friendliness in Children Adopted from Romanian Orphanages. Child Development, 69(4), 1092-1106.

Clark, R., Hyde, J. S., Essex, M. J., & Klein, M. H. (1997). Length of Maternity Leave and Quality of Mother-Infant Interactions. Child Development, 68(2), 364-383.

Coleman, P. K., & Karraker, K. H. (1998). Self-Efficacy and Parenting Quality - Findings and Future Applications. Developmental Review, 18(1), 47-85.

Cornish, A. M., McMahon, C. A., Ungerer, J. A., Barnett, B., Kowalenko, N., & Tennant, C. (2006). Maternal depression and the experience of parenting in the second postnatal year. Journal of Reproductive & Infant Psychology, 24(2), 121-132. doi: 10.1080/02646830600644021.

DeGangi, G. A., Sickel, R. Z., Wiener, A. S., & Kaplan, E. P. (1996). Fussy babies: to treat or not to treat? British Journal of Occupational Therapy, 59(10), 457-64.

Deković, M., Janssens, J. M., & van As, N. M. (2003). Family Predictors of Antisocial Behavior in Adolescence. Family Process, 42(2), 223. doi: Article.

Dumas, J. E., & Wekerle, C. (1995). Maternal Reports of Child-Behavior Problems and Personal Distress as Predictors of Dysfunctional Parenting. Development and Psychopathology, 7(3), 465-479.

Dumas, J. E., Wolf, L. C., Fisman, S. N., & Culligan, A. (1991). Parenting stress, child behavior problems, and dysphoria in parents of children with autism, Down syndrome, behavior disorders, and normal development. Exceptionality, 2(2), 97-110.

Dundas, S., & Kaufman, M. (2000). The Toronto Lesbian Family Study. Journal of Homosexuality, 40(2), 65-79.

Ethier, L. S., Lacharite, C., & Couture, G. (1995). Childhood adversity, parental stress, and depression of negligent mothers. Child Abuse & Neglect, 19(5), 619-32.

Eyberg, S. M. B. S. R. A. J. (1995). Parent-child interaction therapy: A psychosocial model for the treatment of young children with conduct problem behavior and their families. Psychopharmacology Bulletin, 31(1), 83-91.

Fisman, S., Wolf, L., Ellison, D., Gillis, B., Freeman, T., & Szatmari, P. (1996). Risk and Protective Factors Affecting the Adjustment of Siblings of Children with Chronic Disabilities. Journal of the American Academy of Child and Adolescent Psychiatry, 35(11), 1532-1541.

Fitzgerald, M., Butler, B., & Kinsella, A. (1990). The burden on a family having a child with special needs. Irish Journal of Psychological Medicine, 7(2), 109-113.

Flory, V. (2004). A Novel Clinical Intervention for Severe Childhood Depression and Anxiety. Clinical Child Psychology and Psychiatry, 9(1), 9-23. doi: 10.1177/1359104504039167.

Forgays, D. K., Hasazi, J. E., & Wasserman, R. C. (1992). Recurrent otitis media and parenting stress in mothers of two-year-old. Journal of Developmental & Behavioral Pediatrics, 13(5), 321-325.

Frankel, K. K., & Harmon, R. J. (1996). Depressed Mothers - They Dont Always Look as Bad as They Feel. Journal of the American Academy of Child and Adolescent Psychiatry, 35(3), 289-298.

Gartstein, M. A., & Sheeber, L. (2004). Child Behavior Problems and Maternal Symptoms of Depression: A Mediational Model. Journal of Child & Adolescent Psychiatric Nursing, 17(4), 141-150. doi: Article.

Gelfand, D. M., Teti, D. M., Seiner, S. A., & Jameson, P. B. (1996). Helping Mothers Fight Depression - Evaluation of a Home-Based Intervention Program for Depressed Mothers and Their Infants. Journal of Clinical Child Psychology, 25(4), 406-422.

Gross, D., Fogg, L., & Tucker, S. (1995). The Efficacy of Parent Training for Promoting Positive Parent Toddler Relationships. Research in Nursing & Health, 18(6), 489-499.

Ha, E. H., Oh, K. J., & Kim, E. J. (1999). Depressive symptoms and family relationship of married women: Focused on parenting stress and marital dissatisfaction. Korean Journal of Clinical Psychology, 18(1), 79-93.

Hall, S., Bobrow, M., & Marteau, T. M. (1997). Parents attributions of blame for the birth of a child with Down syndrome: a pilot study. Psychology & Health, 12(4), 579-87.

Harmer, A. L. M., Sanderson, J., & Mertin, P. (1999). Influence of Negative Childhood Experiences on Psychological Functioning, Social Support, and Parenting for Mothers Recovering from Addiction. Child Abuse & Neglect, 23(5), 421-433.

Hauenstein, E. J., Marvin, R. S., Snyder, A. L., & Clarke, W. L. (1989). Stress in parents of children with diabetes mellitus. Diabetes Care, 12(1), 18-23.

Hooper, S. R., Burchinal, M. R., Roberts, J. E., Zeisel, S., & Neebe, E. C. (1998). Social and Family Risk-Factors for Infant Development at One-Year - An Application of the Cumulative Risk Model. Journal of Applied Developmental Psychology, 19(1), 85-96.

Horwitz, S. M., Briggs-Gowan, M. J., Storfer-Isser, A., & Carter, A. S. (2009). Persistence of Maternal Depressive Symptoms throughout the Early Years of Childhood. Journal of Women's Health, 18(5), 637-645. doi: 10.1089/jwh.2008.1229.

Hughes, J. N., Cavell, T. A., & Grossman, P. B. (1997). A Positive View of Self - Risk or Protection for Aggressive-Children. Development and Psychopathology, 9(1), 75-94.

Jackson, A. P. (1998). The Role of Social Support in Parenting for Low-Income, Single, Black Mothers. Social Service Review, 72(3), 365-378.

Jackson, A. P. (1999). The Effects of Nonresident Father Involvement on Single Black Mothers and Their Young-Children. Social Work, 44(2), 156-166.

Jackson, A. P., Gyamfi, P., Brooksgunn, J., & Blake, M. (1998). Employment Status, Psychological Well-Being, Social Support, and Physical Discipline Practices of Single Black Mothers. Journal of Marriage and the Family, 60(4), 894-902.

Kazdin, A. E. (1994). Family adversity, socioeconomic disadvantage, and parental stress: Contextual variables related to premature termination from child behavior therapy. Psicologia Conductual, 2(1), 5-21.

Kazdin, A. E., Siegel, T. C., & Bass, D. (1992). Cognitive problem-solving skills training and parent management training in the treatment of antisocial behavior in children. Journal of Consulting and Clinical Psychology, 60, 733-747.

Kern, J. K., West, E. Y., Grannemann, B. D., Greer, T., Snell, L. M., Cline, L. L., …Trivedi, M. H. (2004). Reduction in stress and depressive symptoms in mothers of substance-exposed infants, participating in a psychosocial program. Maternal & Child Health Journal, 8(3), 127-136.

Kobe, F. H., & Hammer, D. (1994). Parenting stress and depression in children with mental retardation and developmental disabilities. Research in Developmental Disabilities, 15(3), 209-21.

Krauss, M. W. (1993). Child-Related and Parenting Stress - Similarities and Differences Between Mothers and Fathers of Children with Disabilities. American Journal on Mental Retardation, 97(4), 393-404.

Kushalnagar, P., Krull, K., Hannay, J., Mehta, P., Caudle, S., & Oghalai, J. (2007). Intelligence, parental depression, and behavior adaptability in deaf children being considered for cochlear implantation. Journal of Deaf Studies and Deaf Education, 12(3), 335-349.

Leigh, B., & Milgrom, J. (2008). Risk factors for antenatal depression, postnatal depression and parenting stress. BMC psychiatry, 8(1), 24.

Levendosky, A. A., & Grahambermann, S. A. (1998). The Moderating Effects of Parenting Stress on Childrens Adjustment in Woman-Abusing Families. Journal of Interpersonal Violence, 13(3), 383-397.

Ligezinska, M., Firestone, P., Manion, I. G., Mcintyre, J., Ensom, R., & Wells, G. (1996). Childrens Emotional and Behavioral Reactions Following the Disclosure of Extrafamilial Sexual Abuse - Initial Effects. Child Abuse & Neglect, 20(2), 111-125.

Lynn, T., Singer, P., Ann Salvator, M. S., Shenyang Guo, P., Marc Collin, M. D., Lawrence Lilien, M. D., & Jill Baley, M. D. (1999). Maternal Psychological Distress and Parenting Stress After the Birth of a Very Low-Birth-Weight Infant. Journal of the American Medical Association, 281(9), 799-805.

Mattie-Luksic, M., Javornisky, G., & DiMario, F. J. (2000). Assessment of stress in mothers of children with severe breath-holding spells. Pediatrics, 106(1 Pt 1), 1-5.

McBride, B. A. (1991). Parental support programs and paternal stress: An exploratory study. Early Childhood Research Quarterly, 6(2), 137-140.

McCarthy, P., Sundby, M., Merladet, J., & Luxenberg, M. G. (1997). Identifying Attendance Correlates for a Teen and Young-Adult Parenting Program. Family Relations, 46(2), 107-112.

McCue Horwitz, S., Briggs-Gowan, M. J., Storfer-Isser, A., & Carter, A. S. (2007). Prevalence, Correlates, and Persistence of Maternal Depression. Journal of Women's Health, 16(5), 678-691. doi: 10.1089/jwh.2006.0185.

Meager, I., & Milgrom, J. (1996). Group treatment for postpartum depression: A pilot study. Australian & New Zealand Journal of Psychiatry, 30(6), 852-860.

Messer, S. C., & Gross, A. M. (1995). Childhood Depression and Family-Interaction - A Naturalistic Observation Study. Journal of Clinical Child Psychology, 24(1), 77-88.

Meyer, W. J., Blakeney, P., Moore, P., Murphy, L., Robson, M., & Herndon, D. (1994). Parental well-being and behavioral adjustment of pediatric survivors of burns. Journal of Burn Care & Rehabilitation, 15(1), 62-8.

Miceli, P. J., Goeke-Morey, M. C., Whitman, T. L., Kolberg, K. S., Miller-Loncar, C., & White, R. D. (2000). Brief report: birth status, medical complications, and social environment: individual differences in development of preterm, very low birth weight infants. Journal of Pediatric Psychology, 25(5), 353-8.

Milgrom, J., & McCloud, P. (1996). Parenting Stress and Postnatal Depression. Stress Medicine, 12(3), 177-186.

Milgrom, J., Westley, D. T., & McCloud, P. I. (1995). Do Infants of Depressed Mothers Cry More Than Other Infants. Journal of Paediatrics and Child Health, 31(3), 218-221.

Mitchelson, J. K., & Burns, L. R. (1998). Career Mothers and Perfectionism - Stress at Work and at Home. Personality and Individual Differences, 25(3), 477-485.

Musil, C. M. (1998). Health, stress, coping, and social support in grandmother caregivers. Health Care for Women International, 19(5), 441-55.

Mylod, D. E., Whitman, T. L., & Borkowski, J. G. (1997). Predicting Adolescent Mothers Transition to Adulthood. Journal of Research on Adolescence, 7(4), 457-478.

Oberlander, T. F., Reebye, P., Misri, S., Papsdorf, M., Kim, J., & Grunau, R. E. (2007). Externalizing and attentional behaviors in children of depressed mothers treated with a selective serotonin reuptake inhibitor antidepressant during pregnancy. Archives of Pediatrics & Adolescent Medicine, 161(1), 22-29. doi: 10.1001/archpedi.161.1.22.

Ostberg, M., Hagekull, B., & Wettergren, S. (1997). A measure of parental stress in mothers with small children: dimensionality, stability and validity. Scandinavian Journal of Psychology, 38(3), 199-208.

Planos, R., Zayas, L. H., & Buschrossnagel, N. A. (1997). Mental-Health Factors and Teaching Behaviors Among Low-Income Hispanic Mothers. Families in Society-The Journal of Contemporary Human Services, 78(1), 4-12.

Quittner, A. L., Digirolamo, A. M., Michel, M., & Eigen, H. (1992). Parental Response to Cystic-Fibrosis - A Contextual Analysis of the Diagnosis Phase. Journal of Pediatric Psychology, 17(6), 683-704.

Radcliffe, J., Bennett, D., Kazak, A. E., Foley, B., & Phillips, P. C. (1996). Adjustment in childhood brain tumor survival: child, mother, and teacher report. Journal of Pediatric Psychology, 21(4), 529-39.

Reid, M. J., Walter, A. L., & Oleary, S. G. (1999). Treatment of Young Childrens Bedtime Refusal and Nighttime Wakings - A Comparison of Standard and Graduated Ignoring Procedures. Journal of Abnormal Child Psychology, 27(1), 5-16.

Reissland, N., Hopkins, B., Helms, P., & Williams, B. (2009). Maternal stress and depression and the lateralisation of infant cradling. Journal of Child Psychology and Psychiatry, and Allied Disciplines, 50(3), 263-269. doi: 10.1111/j.1469-7610.2007.01791.x.

Rhodes, J. E., Ebert, L., & Fischer. (1992). Natural mentors: An overlooked resource in the social networks of young, African-American mothers. American Journal of Community Psychology, 20(4), 445-461.

Richters, J. E. (1992). Depressed Mothers as Informants About Their Children - A Critical-Review of the Evidence for Distortion. Psychological Bulletin, 112(3), 485-499.

Scott, B. S., Atkinson, L., Minton, H. L., & Bowman, T. (1997). Psychological Distress of Parents of Infants with Down-Syndrome. American Journal on Mental Retardation, 102(2), 161-171.

Silver, E. J., Henegham, A. M., Bauman, L. J., & Stein, R. (2006). The relationship of depressive symptoms to parenting competence and social support in inner-city mothers of young children. Maternal & Child Health Journal, 10(1), 105-112.

Singer, L. T., Davillier, M., Bruening, P., Hawkins, S., & Yamashita, T. S. (1996). Social Support, Psychological Distress, and Parenting Strains in Mothers of Very-Low-Birth-Weight Infants. Family Relations, 45(3), 343-350.

Singer, L. T., Salvator, A., Guo, S. Y., Collin, M., Lilien, L., & Baley, J. (1999). Maternal Psychological Distress and Parenting Stress After the Birth of a Very-Low-Birth-Weight Infant. Journal of the American Medical Association, 281(9), 799-805.

Singer, L. T., Song, L.-y., Hill, B. P., & Jaffe, A. C. (1990). Stress and depression in mothers of failure-to-thrive children. Journal of Pediatric Psychology, 15(6), 711-720.

Soliday, E., McCluskey-Fawcett, K., & O'Brien, M. (1999). Postpartum Affect and Depressive Symptoms in Mothers and Fathers. American Journal of Orthopsychiatry, 69(1), 30-38.

Speltz, M. L., Endriga, M. C., Fisher, P. A., & Mason, C. A. (1997). Early Predictors of Attachment in Infants with Cleft-Lip and/or Palate. Child Development, 68(1), 12-25.

Tan, S., & Rey, J. (2005). Depression in the young, parental depression and parenting stress. Australasian Psychiatry, 13(1), 76-79. doi: 10.1111/j.1440-1665.2004.02155.x.

Teti, D. M., Messinger, D. S., Gelfand, D. M., & Isabella, R. (1995). Maternal Depression and the Quality of Early Attachment - An Examination of Infants, Preschoolers, and Their Mothers. Developmental Psychology, 31(3), 364-376.

Thome, M., & Alder, B. (1999). A Telephone Intervention to Reduce Fatigue and Symptom Distress in Mothers with Difficult Infants in the Community. Journal of Advanced Nursing, 29(1), 128-137.

Volling, B. L., Notaro, P. C., & Larsen, J. J. (1998). Adult Attachment Styles - Relations with Emotional Well-Being, Marriage, and Parenting. Family Relations, 47(4), 355-367.

Wall, J. E., & Holden, E. W. (1994). Aggressive, Assertive, and Submissive Behaviors in Disadvantaged, Inner-City Preschool-Children. Journal of Clinical Child Psychology, 23(4), 382-390.

Wanamaker, C. E., & Glenwick, D. S. (1998). Stress, Coping, and Perceptions of Child-Behavior in Parents of Preschoolers with Cerebral-Palsy. Rehabilitation Psychology, 43(4), 297-312.

Webster-Stratton, C. (1988). Mothers' and fathers' perceptions of child deviance: Roles of parent and child behaviors and parent adjustment. Journal of Consulting & Clinical Psychology, 56(6), 909-915.

Webster-Stratton, C., & Herman, K. C. (2008). The Impact of Parent Behavior-Management Training on Child Depressive Symptoms. Journal of Counseling Psychology, 55(4), 473-484.

Webster-Stratton, C., & Spitzer, A. (1996). Parenting a Young-Child with Conduct Problems - New Insights Using Qualitative Methods. Advances in Clinical Child Psychology, 18, 1-62.

Weinreb, M., & Varda, K. (1996). Birthmothers: A retrospective analysis of the surrendering experience. Psychotherapy in Private Practice, 15(1), 59-70.

Wolf, L. C., & Fisman, N. S. (1989). Psychological effects of parenting stress on parents of autistic children. Journal of Autism & Developmental Disorders, 19(1), 157-166.

Wong, F., & Poon, A. (2010). Cognitive behavioural group treatment for Chinese parents with children with developmental disabilities in Melbourne, Australia: An efficacy study, Australian and New Zealand Journal of Psychiatry, 44, 742-749.

Wysocki, T., Huxtable, K., Linscheid, T. R., & Wayne, W. (1989). Adjustment to diabetes mellitus in preschoolers and their mothers. Diabetes Care, 12(8), 524-9.

Postpartum Depression

Birkeland, R., Thompson, J. K., & Phares, V. (2005). Adolescent motherhood and postpartum depression. Journal of Clinical Child and Adolescent Psychology, 34(2), 292–300.

Dunne, L., Sneddon, H., Iwaniec, D., & Stewart, M. C. (2007). Maternal mental health and faltering growth in infants. Child Abuse Review, 16(5), 283-295.

Feldman, R., Granat, A., Pariente, C., Kanety, H., Kuint, J., & Gilboa-Schechtman, E. (2009). Maternal Depression and Anxiety Across the Postpartum Year and Infant Social Engagement, Fear Regulation, and Stress Reactivity. Journal of Amer Academy of Child & Adolescent Psychiatry, 48(9), 919 - 927.

Glavin, K., Smith, L., Sørum, R., & Ellefsen, B. (2010). Redesigned community postpartum care to prevent and treat postpartum depression in women - a one-year follow-up study. Journal of Clinical Nursing, 19, 3051-3062.

Misri, S., Reebye, P., Milis, L., & Shah, S. (2006). The impact of treatment intervention on parenting stress in postpartum depressed mothers: a prospective study. American Journal of Orthopsychiatry, 76(1), 115–119.

Morrell, C. J., Slade, P., Warner, R., Paley, G., Dixon, S., Walters, S. J., … Nicholl, J. (2009). Clinical effectiveness of health visitor training in psychologically informed approaches for depression in postnatal women: pragmatic cluster randomised trial in primary care. British Medical Journal, 338:a3045.

Tervo, R. (2010). Attention Problems and Parent-Rated Behavior and Stress in Young Children at Risk for Developmental Delay, Journal of Child Neurology, 25, 1325-1330.

Thome, M. (2003). Severe postpartum distress in Icelandic mothers with difficult infants: a follow-up study on their health care. Scandinavian Journal of Caring Sciences, 17(2), 104-112. doi: 10.1046/j.1471-6712.2003.00110.x.

Zelkowitz, P., & Milet, T. H. (2001). The course of postpartum psychiatric disorders in women and their partners. The Journal of Nervous and Mental Disease, 189(9), 575-582.

Developmental Disabilities

Butter, E. M., Mulick, J. A., & Metz, B. (2006). Eight case reports of learning recovery in children with pervasive developmental disorders after early intervention. Behavioral Interventions, 21(4), 227-243. doi: 10.1002/bin.225.

Cowen, P. S., & Reed, D. A. (2002). Effects of respite care for children with developmental disabilities: evaluation of an intervention for at risk families. Public Health Nursing, 19(4), 272-283.

Drummond, J., McDonald, L., MacKenzie-Keating, S., & Fleming, D. (2004). Types of support accessed by families of young children with disabilities living in Alberta. Developmental Disabilities Bulletin, 32(1), 1-27.

Guralnick, M., Neville, B., Hammond, M., & Connor, R. (2007). The friendships of young children with developmental delays: A longitudinal analysis. Journal of Applied Developmental Psychology, 28(1), 64-79. doi: 10.1016/j.appdev.2006.10.004.

Kersh, J., Hedvat, T., Hauser-Cram, P., & Warfield, M. E. (2006). The contribution of marital quality to the well-being of parents of children with developmental disabilities. Journal of Intellectual Disability Research, 50(12), 883-893. doi: 10.1111/j.1365-2788.2006.00906.x.

Nachshen, J. S., & Minnes, P. (2005). Empowerment in parents of school-aged children with and without developmental disabilities. Journal of Intellectual Disability Research, 49(12), 889–904.

Nereo, N. E., Fee, R. J., & Hinton, V. J. (2003). Parental stress in mothers of boys with Duchenne muscular dystrophy. Journal of Pediatric Psychology, 28(7), 473-484.

Oelofsen, N., & Richardson, P. (2006). Sense of coherence and parenting stress in mothers and fathers of preschool children with developmental disability. Journal of Intellectual & Developmental Disability, 31(1), 1-12. doi: 10.1080/13668250500349367.

Quinn, M., Carr, A., Carroll, L., & O Sullivan, D. (2006). An evaluation of the Parents Plus Programme for pre-school children with conduct problems: A comparison of those with and without developmental disabilities. Irish Journal of Psychology, 27(3/4), 168-182.

Rimmerman, A., Turkel, L., & Crossman, R. (2003). Perception of child development, child-related stress and dyadic adjustment: pair analysis of married couples of young children with developmental disabilities. Journal of Intellectual & Developmental Disability, 28(2), 188. doi: Article.

Singh, N. N., Lancioni, G. E., Winton, A. S., Singh, J., Curtis, W. J., Wahler, R. G., McAleavey, K. M. (2007). Mindful parenting decreases aggression and increases social behavior in children with developmental disabilities. Behavior Modification, 31(6), 749-771.

Trute, B., & Hiebert-Murphy, D. (2002). Family adjustment to childhood developmental disability: A measure of parent appraisal of family impacts. Journal of Pediatric Psychology, 27(3), 271-280.

Weiss, J. A., Sullivan, A., & Diamond, T. (2003). Parent stress and adaptive functioning of individuals with developmental disabilities. Journal on Developmental Disabilities, 10(1), 129-136.

Woolfson, L., & Grant, E. (2006). Authoritative parenting and parental stress in parents of pre-school and older children with developmental disabilities. Child: Care, Health & Development, 32(2), 177-184.

Autism

Baker-Ericzén, M. J., Brookman-Frazee, L., & Stahmer, A. (2005). Stress levels and adaptability in parents of toddlers with and without autism spectrum disorders. Research and Practice for Persons with Severe Disabilities, 30(4), 194–204.

Corbett, B. A, Schupp, C. W., Levine, S., & Mendoza, S. (2009). Comparing cortisol, stress, and sensory sensitivity in children with autism. Autism Research, 2(1),39-49.

Davis, N. O., & Carter, A. S. (2008). Parenting Stress in Mothers and Fathers of Toddlers with Autism Spectrum Disorders: Associations with Child Characteristics.. Journal of Autism & Developmental Disorders, 38(7), 1278-1291. doi: 10.1007/s10803-007-0512-z.

Donenberg, G., & Baker, B. L. (1993). The Impact of Young-Children with Externalizing Behaviors on Their Families. Journal of Abnormal Child Psychology, 21(2), 179-198.

Dumas, J. E., Wolf, L. C., Fisman, S. N., & Culligan, A. (1991). Parenting stress, child behavior problems, and dysphoria in parents of children with autism, Down syndrome, behavior disorders, and normal development. Exceptionality, 2(2), 97-110.

Fisman, S., Wolf, L., Ellison, D., Gillis, B., Freeman, T., & Szatmari, P. (1996). Risk and Protective Factors Affecting the Adjustment of Siblings of Children with Chronic Disabilities. Journal of the American Academy of Child and Adolescent Psychiatry, 35(11), 1532-1541.

Freeman, N. L. P. A. (1991). Child behaviours as stressors: Replicating and extending the use of the CARS. Journal of Child Psychology & Psychiatry & Allied Disciplines, 32(6), 1025-1030.

Hindley, P. (1997). Psychiatric Aspects of Hearing Impairments. Journal of Child Psychology and Psychiatry and Allied Disciplines, 38(1), 101-117.

Hoffman, C. D., Sweeney, D. P., Hodge, D., Lopez-Wagner, M. C., & Looney, L. (2009). Parenting Stress and Closeness: Mothers of Typically Developing Children and Mothers of Children With Autism. Focus on Autism and Other Developmental Disabilities, 24(3), 178-187. doi: 10.1177/1088357609338715

Hoppes, K., & Harris, S. L. (1990). Perceptions of child attachment and maternal gratification in mothers of children with Autism and Down's Syndrome. Journal of Clinical Child Psychology, 19, 365-370.

Kasari, C., & Sigman, M. (1997). Linking Parental Perceptions to Interactions in Young-Children with Autism. Journal of Autism and Developmental Disorders, 27(1), 39-57.

Kenworthy, L., & Charnas, L. (1995). Evidence for a Discrete Behavioral-Phenotype in the Oculocerebrorenal Syndrome of Lowe. American Journal of Medical Genetics, 59(3), 283-290.

Kuhn, J. C., & Carter, A. S. (2006). Maternal self-efficacy and associated parenting cognitions among mothers of children with autism. American Journal of Orthopsychiatry, 76(4), 564–575.

Lecavalier, L., Leone, S., & Wiltz, J. (2006). The impact of behaviour problems on caregiver stress in young people with autism spectrum disorders. Journal of Intellectual Disability Research, 50 (3), 172-183.

Lobato, D. (1985). Preschool siblings of handicapped children: Impact of peer support and training. Journal of Autism & Developmental Disorders, 15(3), 345-350.

Mak, W. W. S., Ho, A .H. Y., & Law, R. W. (2007). Sense of coherence, parenting attitudes and stress among mothers of children with autism in Hong Kong. Journal of Applied Research in Intellectual Disabilities, 20(2), 157-167.

Ooi, Y. P., Lam, C. M., Sung, M., Tan, W. T. S., Goh, T. J., Fung, D. S. S., et al. (2008). Effects of cognitive-behavioural therapy on anxiety for children with high-functioning autistic spectrum disorders. Singapore Medical Journal, 49(3), 215-220.

Osborne, L. A., & Reed, P. (2009). The Relationship Between Parenting Stress and Behavior Problems of Children With Autistic Spectrum Disorders. Exceptional Children, 76(1), 54-73.

Perry, A., Sarlo-McGarvey, N., & Factor, D. C. (1992). Stress and family functioning in parents of girls with Rett syndrome. Journal of Autism & Developmental Disorders, 22(2), 235-248.

Qin, X., Tang, C., Zhu, S., Liang, Y., & Zou, X. (2009). Parenting stress and related factors in mothers of children with autism. Chinese Mental Health Journal, 23(9), 629-633.

Richman, D., Belmont, J., Kim, M., Slavin, C., & Hayner, A. (2009). Parenting stress in families of children with Cornelia de Lange Syndrome and Down Syndrome, Journal of Developmental and Physical Disabilities, 21, 537-553.

Robbins, F. R., & Dunlap, G. (1992). Effects of task difficulty on parent teaching skills and behavior problems of young children with autism. American Journal on Mental Retardation, 96(6), 631-643.

Robbins, F. R., Dunlap, G., & Plienis, A. J. (1991). Family characteristics, family training, and the progress of young children with autism. Journal of Early Intervention, 15(2), 173-184.

Rodger, S., Keen, D., Braithwaite, M., & Cook, S. (2008). Mothers' Satisfaction with a Home Based Early Intervention Programme for Children with ASD. Journal of Applied Research in Intellectual Disabilities, 21(2), 174-182. doi: 10.1111/j.1468-3148.2007.00393.x.

Solomon, M., Ono, M., Timmer, S., & Goodlin-Jones, B. (2008). The Effectiveness of Parent–Child Interaction Therapy for Families of Children on the Autism Spectrum.. Journal of Autism & Developmental Disorders, 38(9), 1767-1776. doi: 10.1007/s10803-008-0567-5.

Sperry, L. A., & Symons, F. J. (2003). Maternal judgments of intentionality in young children with autism: the effects of diagnostic information and stereotyped behavior. Journal of Autism and Developmental Disorders, 33(3), 281–287.

Szatmari, P., Archer, L., Fisman, S., & Streiner, D. L. (1994). Parent and Teacher Agreement in the Assessment of Pervasive Developmental Disorders. Journal of Autism and Developmental Disorders, 24(6), 703-717.

Tomanik, S., Harris, G. E., & Hawkings, J. (2004). The relationship between behaviours exhibited by children with autism and maternal stress. Journal of Intellectual & Developmental Disability, 29(1), 16-26.

Wolf, L. C., & Fisman, N. S. (1989). Psychological effects of parenting stress on parents of autistic children. Journal of Autism & Developmental Disorders, 19(1), 157-166.

Wong, V., & Kwan, Q. (2009). Randomized control trial for early intervention for Autism: A pilot study of the Autism 1-2-3 Project. Journal of Autism and Developmental Disorders, 40, 677-688.

Zaidman-Zait, A., Mirena, P., Zumbo, B., Wellington, S., Dua, V., & Kalynchuk, K. (2010). An item response theory analysis of the Parenting Stress Index-Short Form with parents of children with autism spectrum disorders. Journal of Child Psychology and Psychiatry, 51, 1269-1277.

Zelazo, P. R. (1997). Infant-Toddler Information-Processing Treatment of Children with Pervasive Developmental Disorder and Autism .2. Infants and Young Children, 10(2), 1-13.

Asperger's Syndrome

Epstein, T., Saltzman-Benaiah, J., O'Hare, A., Goll, J. C., & Tuck, S. (2008). Associated features of Asperger Syndrome and their relationship to parenting stress. Child: Care, Health & Development, 34(4),503-511.

Zaidman-Zait, A., Mirena, P., Zumbo, B., Wellington, S., Dua, V., & Kalynchuk, K. (2010). An item response theory analysis of the Parenting Stress Index-Short Form with parents of children with autism spectrum disorders. Journal of Child Psychology and Psychiatry, 51, 1269-1277.

Down's Syndrome

Cuskelly, M., Chant, D., & Hayes, A. (1998). Behaviour problems in the siblings of children with Down syndrome: Associations with family responsibilities and parental stress. International Journal of Disability, Development & Education, 45(3), 295-311.

Duis, S. S., Summers, M., & Summers, C. R. (1997). Parent Versus Child Stress in Diverse Family Types - An Ecological Approach. Topics in Early Childhood Special Education, 17(1), 53-73.

Dumas, J. E., Wolf, L. C., Fisman, S. N., & Culligan, A. (1991). Parenting stress, child behavior problems, and dysphoria in parents of children with autism, Down syndrome, behavior disorders, and normal development. Exceptionality, 2(2), 97-110.

Fey, M. E., Warren, S. F., Brady, N., Finestack, L. H., Bredin-Oja, S. L., Fairchild, M., et al. (2006). Early Effects of Responsivity Education/Prelinguistic Milieu Teaching for Children With Developmental Delays and Their Parents. Journal of Speech, Language & Hearing Research, 49(3), 526-547. doi: 10.1044/1092-4388(2006/039).

Fisman, S., Wolf, L., Ellison, D., Gillis, B., Freeman, T., & Szatmari, P. (1996). Risk and Protective Factors Affecting the Adjustment of Siblings of Children with Chronic Disabilities. Journal of the American Academy of Child and Adolescent Psychiatry, 35(11), 1532-1541.

Greaves, D. (1997). The effect of rational-emotive parent education on the stress of mothers of young children with Down syndrome. Journal of Rational-Emotive & Cognitive Behavior Therapy, 15(4), 249-267.

Hall, S., & Marteau, T. M. (2003). Causal attributions and blame: associations with mothers' adjustment to the birth of a child with Down syndrome. Psychology, Health & Medicine, 8(4), 415. doi: 10.1080/1354850310001604559.

Hall, S., Bobrow, M., & Marteau, T. M. (1997). Parents attributions of blame for the birth of a child with Down syndrome: a pilot study. Psychology & Health, 12(4), 579-87.

Hanson, M. J., & Hanline, M. F. (1990). Parenting a child with a disability: A longitudinal study of parental stress and adaptation. Journal of Early Intervention, 14(3), 234-248.

Hodapp, R. M., Ricci, L. A., Ly, T. A., & Fidler, D. J. (2003). The effects of the child with Down syndrome on maternal stress. British Journal of Developmental Psychology, 21(1), 137-151.

Hoppes, K., & Harris, S. L. (1990). Perceptions of child attachment and maternal gratification in mothers of children with Autism and Down's Syndrome. Journal of Clinical Child Psychology, 19, 365-370.

Krauss, M. W., Upshur, C. C., Shonkoff, J. P., & Hausercram, P. (1993). The Impact of Parent Groups on Mothers of Infants with Disabilities. Journal of Early Intervention, 17(1), 8-20.

Mahoney, G., Perales, F., Wiggers, B., & Herman, B. (2006). Responsive Teaching: Early intervention for children with Down syndrome and other disabilities. DOWNS SYNDROME RESEARCH AND PRACTICE, 11(1), 18.

Most, D. E., Fidler, D. J., Laforce-Booth, C., & Kelly, J. (2006). Stress trajectories in mothers of young children with Down syndrome. Journal of Intellectual Disability Research, 50(7), 501-514. doi: 10.1111/j.1365-2788.2006.00796.x.

Richman, D., Belmont, J., Kim, M., Slavin, C., & Hayner, A. (2009). Parenting stress in families of children with Cornelia de Lange Syndrome and Down Syndrome, Journal of Developmental and Physical Disabilities, 21, 537-553.

Scott, B. S., Atkinson, L., Minton, H. L., & Bowman, T. (1997). Psychological Distress of Parents of Infants with Down-Syndrome. American Journal on Mental Retardation, 102(2), 161-171.

Wolf, L. C., & Fisman, N. S. (1989). Psychological effects of parenting stress on parents of autistic children. Journal of Autism & Developmental Disorders, 19(1), 157-166.

Wolf, L. C., Fisman, S., Ellison, D., & Freeman, T. (1998). Effect of Sibling Perception of Differential Parental Treatment in Sibling Dyads with One Disabled-Child. Journal of the American Academy of Child and Adolescent Psychiatry, 37(12), 1317-1325.

Mental Retardation

Beck, A., Hastings, R. P., Daley, D., & Stevenson, J. (2004). Pro-social behaviour and behaviour problems independently predict maternal stress. Journal of Intellectual and Developmental Disability, 29(4), 339–349.

Beckman, P. J. (1991). Comparison of mothers' and fathers' perceptions of the effect of young children with and without disabilities. American Journal on Mental Retardation, 95(5), 585-595.

Cameron, S. J., & Orr, R. (1989). Stress in families of school-aged children with delayed mental development. Canadian Journal of Rehabilitation, 2(3), 137-144.

Camfield, C., Breau, L., & Camfield, P. (2001). Impact of pediatric epilepsy on the family: a new scale for clinical and research use. Epilepsia, 42(1), 104-12.

Embregts, P., Grimbel du Bois, M., & Graef, N. (2010). Behavior problems in children with mild intellectual disabilities: An initial step towards prevention. Research in Developmental Disabilities, 31, 1398-1403.

Feldman, M. A., & Waltonallen, N. (1997). Effects of Maternal Mental-Retardation and Poverty on Intellectual, Academic, and Behavioral Status of School-Age-Children. American Journal on Mental Retardation, 101(4), 352-364.

Feldman, M. A., Leger, M., & Walton-Allen, N. (1997). Stress in mothers with intellectual disabilities. Journal of Child & Family Studies, 6(4), 471-485.

Feldman, M. A., Varghese, J., Ramsay, J., & Rajska, D. (2002). Relationships between social support, stress and mother-child interactions in mothers with intellectual disabilities. Journal of Applied Research in Intellectual Disabiliites, 15(4), 314-323.

Fey, M. E., Warren, S. F., Brady, N., Finestack, L. H., Bredin-Oja, S. L., Fairchild, M., et al. (2006). Early Effects of Responsivity Education/Prelinguistic Milieu Teaching for Children With Developmental Delays and Their Parents. Journal of Speech, Language & Hearing Research, 49(3), 526-547. doi: 10.1044/1092-4388(2006/039).

Golombok, S., Cook, R., Bish, A., & Murray, C. (1993). Quality of Parenting in Families Created by the New Reproductive Technologies - A Brief Report of Preliminary Findings. Journal of Psychosomatic Obstetrics and Gynecology, 14(S), 17-22.

Guralnick, M. J., Hammond, M. A., Connor, R. T., & Neville, B. (2006). Stability, change, and correlates of the peer relationships of young children with mild developmental delays. Child development, 77(2), 312–324.

Hassall, R., Rose, J., & McDonald, J. (2005). Parenting stress in mothers of children with an intellectual disability: the effects of parental cognitions in relation to child characteristics and family support. Journal of Intellectual Disability Research, 49(6), 405-418.

Hill, C., & Rose, J. (2009). Parenting stress in mothers of adults with an intellectual disability: parental cognitions in relation to child characteristics and family support. Journal of Intellectual Disability Research: JIDR. doi: 10.1111/j.1365-2788.2009.01207.x

Huang, W., Rubin, S. E., & Zhang, F. (1998). Correlates of stress level in Chinese mothers of a child with mental retardation. International Journal of Rehabilitation Research, 21(2), 237-40.

Kasari, C., & Sigman, M. (1997). Linking Parental Perceptions to Interactions in Young-Children with Autism. Journal of Autism and Developmental Disorders, 27(1), 39-57.

Kobe, F. H., & Hammer, D. (1994). Parenting stress and depression in children with mental retardation and developmental disabilities. Research in Developmental Disabilities, 15(3), 209-21.

Krauss, M. W. (1993). Child-Related and Parenting Stress - Similarities and Differences Between Mothers and Fathers of Children with Disabilities. American Journal on Mental Retardation, 97(4), 393-404.

LaFiosca, T., & Loyd, B. H. (1986). Defensiveness and the assessment of parental stress and anxiety. Journal of Clinical Child Psychology, 15(3), 254-259.

Mrazek, D. A., Mrazek, P., & Klinnert, M. (1995). Clinical-Assessment of Parenting. Journal of the American Academy of Child and Adolescent Psychiatry, 34(3), 272-282.

Msall, M. E., Rogers, B. T., Ripstein, H., Lyon, N., & Wilczenski, F. (1997). Measurements of Functional Outcomes in Children with Cerebral-Palsy. Mental Retardation and Developmental Disabilities Research Reviews, 3(2), 194-203.

Nachshen, J. S., Garcin, N., & Minnes, P. (2005). Problem behavior in children with intellectual disabilities: Parenting stress, empowerment and school services. Mental Health Aspects of Developmental Disabilities, 8(4), 105.

Orr, R. R., Cameron, S. J., Dobson, L. A., & Day, D. M. (1993). Age-related changes in stress experienced by families with a child who has developmental delays. Mental Retardation, 31(3), 171-6.

Quinn, M., Carr, A., Carroll, L., & O'Sullivan, D. (2007). Parents Plus Programme 1: Evaluation of its effectivesness for pre-school children with developmental disabilities

and behavioral problems. Journal of Applied Research in Intellectual Disabilities, 20(4), 345-359.

Sarimski, K. (1995). Psychological aspects of the Prader-Willi syndrome: Results of a survey of parents by mail. Zeitschrift Fuer Kinder- und Jugendpsychiatrie, 23(4), 267-274.

Sarimski, K. (1997). Behavioural phenotypes and family stress in three mental retardation syndromes. European Child & Adolescent Psychiatry, 6(1), 26-31.

Sarimski, K. (1997). Communication, Social-Emotional Development and Parenting Stress in Cornelia-de-Lange-Syndrome. Journal of Intellectual Disability Research, 41(FEB), 70-75.

Sarimski, K. (1998). Stress in Mothers of Children with Genetic Syndromes. Zeitschrift fur Klinische Psychologie, Psychiatrie, und Psychotherapie, 46(3), 233-244.

Scott, B. S., Atkinson, L., Minton, H. L., & Bowman, T. (1997). Psychological Distress of Parents of Infants with Down-Syndrome. American Journal on Mental Retardation, 102(2), 161-171.

Shin, J., Nhan, N. V., Crittenden, K. S., Hong, H. T. D., Flory, M., & Ladinsky, J. (2006). Parenting stress of mothers and fathers of young children with cognitive delays in Vietnam. Journal of Intellectual Disability Research, 50(10), 748-760. doi: 10.1111/j.1365-2788.2006.00840.x.

Warfield, M. E., Krauss, M. W., Hauser-Cram, P., Upshur, C. C., & Shonkoff, J. P. (1999). Adaptation during early childhood among mothers of children with disabilities. Journal of Developmental & Behavioral Pediatrics, 20(1), 9-16.

Warren, S. F., Fey, M. E., Finestack, L. H., Brady, N. C., Bredin-Oja, S. L., & Fleming, K. K. (2008). A Randomized Trial of Longitudinal Effects of Low-Intensity Responsivity Education/Prelinguistic Milieu Teaching. Journal of Speech, Language & Hearing Research, 51(2), 451-470. doi: 10.1044/1092-4388(2008/033).

Weiss, J. A., & Diamond, T. (2005). Stress in parents of adults with intellectual disabilities attending Special Olympics competitions. Journal of Applied Research in Intellectual Disabilities, 18(3), 263-270.

Screening for Disabilities

Bithoney, W. G., Van Sciver, M. M., Foster, S., Corso, S., & Tentindo, C. (1995). Parental stress and growth outcome in growth-deficient children. Pediatrics, 96(4 Pt 1), 707-11.

Bradley, R. H., & A., B. J. (1990). Assessment of the home environment. In J. H. Johnson (Ed.), Developmental assessment in clinical child psychology: A handbook (Vol. 163, pp. 219-250). New York: Pergamon Press, Inc.

Bramlett, R. K., Hall, J. D., Barnett, D. W., & Rowell, K. (1995). Child developmental/educational status in kindergarten and family coping as predictors of parenting stress: Issues for parent consultation. Journal of Psychoeducational Assessment, 13(2), 157-166.

Fischel, J. E., Whitehurst, G. J., Caulfield, M. B., & DeBaryshe, B. (1989). Language growth in children with expressive language delay. Pediatrics, 83(2), 218-27.

Girolametto, L., & Tannock, R. (1994). Correlates of Directiveness in the Interactions of Fathers and Mothers of Children with Developmental Delays. Journal of Speech and Hearing Research, 37(5), 1178-1191.

Goldberg, S., Janus, M., Washington, J., Simmons, R. J., Maclusky, I., & Fowler, R. S. (1997). Prediction of Preschool Behavioral-Problems in Healthy and Pediatric Samples. Journal of Developmental and Behavioral Pediatrics, 18(5), 304-313.

Gurian, E. A., Kinnamon, D. D., Henry, J. J., & Waisbren, S. E. (2006). Expanded newborn screening for biochemical disorders: the effect of a false-positive result. Pediatrics, 117(6), 1915-1921.

Hall, J. D., & Barnett, D. W. (1991). Classification of risk status in preschool screening: A comparison of alternative measures. Journal of Psychoeducational Assessment, 9(2), 152-159.

Hall, S., Bobrow, M., & Marteau, T. M. (1997). Parents attributions of blame for the birth of a child with Down syndrome: a pilot study. Psychology & Health, 12(4), 579-87.

Lessenberry, B. M., & Rehfeldt, R. A. (2004). Evaluating Stress Levels of Parents of Children with Disabilities. Exceptional Children, 70(2), 231–245.

Little, M., Murphy, J. M., Jellinek, M. S., Bishop, S. J., & Arnett, H. L. (1994). Screening 4-Year-Old and 5-Year-Old Children for Psychosocial Dysfunction - A Preliminary-Study with the Pediatric Symptom Checklist. Journal of Developmental and Behavioral Pediatrics, 15(3), 191-197.

Lynn, T., Singer, P., Ann Salvator, M. S., Shenyang Guo, P., Marc Collin, M. D., Lawrence Lilien, M. D., & Jill Baley, M. D. (1999). Maternal Psychological Distress and Parenting Stress After the Birth of a Very Low-Birth-Weight Infant. Journal of the American Medical Association, 281(9), 799-805.

Messer, S. C., & Gross, A. M. (1995). Childhood Depression and Family-Interaction - A Naturalistic Observation Study. Journal of Clinical Child Psychology, 24(1), 77-88.

Patterson, K. A., & Starn, J. R. (1993). Program for women and infants exposed to drugs: a legal alternative. Nurse Practitioner Forum, 4(4), 224-30.

Singer, L. T., Salvator, A., Guo, S. Y., Collin, M., Lilien, L., & Baley, J. (1999). Maternal Psychological Distress and Parenting Stress After the Birth of a Very-Low-Birth-Weight Infant. Journal of the American Medical Association, 281(9), 799-805.

Tam, K.-k., Chan, Y.-c., & Wong, C.-k. M. (1994). Validation of the Parenting Stress Index among Chinese mothers in Hong Kong. Journal of Community Psychology, 22(3), 211-223.

Waisbren, S. E., Albers, S., Amato, S., Brewster, T. G., Demmer, L., Eaton, R. B., …Levy, H. L. (2003). Effect of expanded newborn screening for biochemical genetic disorders on child outcomes and parental stress. Journal of the American Medical Association, 290(19), 2564-2572.

Adoption

Belanger, K., Copeland, S., & Cheung, M. (2008). The Role of Faith in Adoption: Achieving Positive Adoption Outcomes for African American Children. Child Welfare, 87(2), 99. doi: Article.

Chisholm, K. (1998). A 3 Year Follow-Up of Attachment and Indiscriminate Friendliness in Children Adopted from Romanian Orphanages. Child Development, 69(4), 1092-1106.

Chisholm, K., Carter, M. C., Ames, E. W., & Morison, S. J. (1995). Attachment security and indiscriminately friendly behavior in children adopted from Romanian orphanages. Development & Psychopathology, 7(2), 283-294.

Cook, R., Golombok, S., Bish, A., & Murray, C. (1995). Disclosure of Donor Insemination - Parental Attitudes. American Journal of Orthopsychiatry, 65(4), 549-559.

Deater-Deckard, K. (1996). Within Family Variability in Parental Negativity and Control. Journal of Applied Developmental Psychology, 17(3), 407-422.

Demick, J., & Andreoletti, C. (1995). Some Relations Between Clinical and Environmental Psychology. Environment and Behavior, 27(1), 56-72.

Mainemer, H., Gilman, L. C., & Ames, E. W. (1998). Parenting stress in families adopting children from Romanian orphanages. Journal of Family Issues, 19(2), 164-180.

McCarty, C., Waterman, J., Burge, D., & Edelstein, S. B. (1999). Experiences, concerns, and service needs of families adopting children with prenatal substance exposure: Summary and recommendations. Child Welfare, 78(5), 561-577.

McGlone, K., Santos, L., Kazama, L., Fong, R., & Mueller, C. (2002). Psychological Stress in Adoptive Parents of Special-Needs Children. Child Welfare, 81(2), 151. doi: Article.

Miranda, A., Grau, D., Rosel, J., & Meliá, A. (2009). Understanding discipline in families of children with attention-deficit/hyperactivity disorder: A structural equation model. The Spanish Journal of Psychology, 12(2), 496-505.

Timmer, S. G., Llrquiza, A. I., Herschell, A. D., McGrath, J. M., Zebell, N. M., Porter, A. L., et al. (2006). Parent-Child Interaction Therapy: Application of an Empirically Supported Treatment to Maltreated Children in Foster Care. Child Welfare, 85(6), 919. doi: Article.

Vorria, P., Papaligoura, Z., Sarafidou, J., Kopakaki, M., Dunn, J., Van IJzendoorn, M. H., Kontopoulou, A. (2006). The development of adopted children after institutional care: A follow-up study. Journal of Child Psychology and Psychiatry, 47(12), 1246–1253.

Weinreb, M., & Varda, K. (1996). Birthmothers: A retrospective analysis of the surrendering experience. Psychotherapy in Private Practice, 15(1), 59-70.

Discipline

Burbach, A. D., Fox, R. A., & Nicholson, B. C. (2004). Challenging Behaviors in Young Children: The Father's Role. Journal of Genetic Psychology, 165(2), 169. doi: Article.

Deković, M., Janssens, J. M., & van As, N. M. (2003). Family Predictors of Antisocial Behavior in Adolescence. Family Process, 42(2), 223. doi: Article.

Murphy, D., Marelich, W., Armistead, L., Herbeck, D. and Payne, D. (2010). Anxiety/stress among mothers living with HIV: effects on parenting skills and child outcomes, AIDS Care, 22, 1449 – 1458.

Child Custody

Bow, J. N., & Quinnell, F. A. (2002). A critical review of child custody evaluation reports. Family Court Review, 40(2), 164-176. doi: 10.1111/j.174-1617.2002.tb00827.x.

Cole, S. A. (2002). Security of Attachment of Infants in Foster Care. In Proceedings of the... National Symposium on Doctoral Research in Social Work (p. 12). Presented at the National Symposium on Doctoral Research in Social Work, Columbus, OH: Ohio State University.

Quinnell, F. A., & Bow, J. N. (2001). Psychological tests used in child custody evaluations. Behavioral Sciences & the Law, 19(4), 491–501.

Divorce

Abidin, R. R., & Brunner, J. F. (1995). Development of a Parenting Alliance Inventory. Journal of Clinical Child Psychology, 24(1), 31-40.

Altmaier, E., & Maloney, R. (2007). An initial evaluation of a mindful parenting program. Journal of Clinical Psychology, 63(12), 1231-1238. doi: 10.1002/jclp.20395.

Arditti, J. A., & Maddenderdich, D. (1997). Joint and Sole Custody Mothers - Implications for Research and Practice. Families in Society - The Journal of Contemporary Human Services, 78(1), 36-45.

Bigras, M., & Lafreniere, P. J. (1994). Influence of Psychosocial Risk, Marital Conflicts and Parental Stress on the Quality of Mother-Son and Mother-Daughter Interactions. Canadian Journal of Behavioural Science, 26(2), 280-297.

Golombok, S., Tasker, F., & Murray, C. (1997). Children Raised in Fatherless Families from Infancy - Family Relationships and the Socioemotional Development of Children of Lesbian and Single Heterosexual Mothers. Journal of Child Psychology and Psychiatry and Allied Disciplines, 38(7), 783-791.

Heinze, M. C., & Grisso, T. (1996). Review of instruments assessing parenting competencies used in child custody evaluations. Behavioral Sciences & the Law, 14(3), 293-313.

Milgrom, J., & McCloud, P. (1996). Parenting Stress and Postnatal Depression. Stress Medicine, 12(3), 177-186.

Summers, M., Summers, C. R., & Ascione, F. R. (1993). A Comparison of Sibling Interaction in Intact and Single-Parent Families. Journal of Divorce & Remarriage, 20(1-2), 215-227.

Employment

Kinnunen, U., Geurts, S., & Mauno, S. (2004). Work-to-family conflict and its relationship with satisfaction and well-being: a one-year longitudinal study on gender differences. Work & Stress, 18(1), 1-22. doi: 10.1080/02678370410001682005.

Families with a Handicapped Child

Burrell, B., Thompson, B., & Sexton, D. (1994). Predicting Child-Abuse Potential Across Family Types. Child Abuse & Neglect, 18(12), 1039-1049.

Button, S., Pianta, R. C., & Marvin, R. S. (2001). Mothers' representations of relationships with their children: Relations with parenting behavior, mother characteristics, and child disability status. Social Development, 10(4), 455-472. doi: 10.1111/1467-9507.00175

Button, S., Pianta, R. C., & Marvin, R. S. (2001). Partner support and maternal stress in families raising young children with cerebral palsy. Journal of Developmental & Physical Disabilities, 13(1), 61-81.

Cappelli, M., Mcgrath, P. J., Daniels, T., Manion, I., & Schillinger, J. (1994). Marital Quality of Parents of Children with Spina-Bifida - A Case-Comparison Study. Journal of Developmental and Behavioral Pediatrics, 15(5), 320-326.

Dempsey, I., & Keen, D. (2008). A Review of Processes and Outcomes in Family-Centered Services for Children With a Disability. Topics in Early Childhood Special Education, 28(1), 42-52. doi: 10.1177/0271121408316699.

Drummond, J., McDonald, L., MacKenzie-Keating, S., & Fleming, D. (2004). Types of support accessed by families of young children with disabilities living in Alberta. Developmental Disabilities Bulletin, 32(1), 1-27.

Fey, M. E., Warren, S. F., Brady, N., Finestack, L. H., Bredin-Oja, S. L., Fairchild, M., et al. (2006). Early Effects of Responsivity Education/Prelinguistic Milieu Teaching for Children With Developmental Delays and Their Parents. Journal of Speech, Language & Hearing Research, 49(3), 526-547. doi: 10.1044/1092-4388(2006/039).

Fisman, S., Wolf, L., Ellison, D., Gillis, B., Freeman, T., & Szatmari, P. (1996). Risk and Protective Factors Affecting the Adjustment of Siblings of Children with Chronic Disabilities. Journal of the American Academy of Child and Adolescent Psychiatry, 35(11), 1532-1541.

Hung, J. W., Wu, Y., & Yeh, C. (2004). Comparing stress levels of parents of children with cancer and parents of children with physical disabilities. Psycho-Oncology, 13(12), 898-903.

Innocenti, M. S., Huh, K., & Boyce, G. C. (1992). Families of children with disabilities: Normative data and other considerations on parenting stress. Topics in Early Childhood Special Education, 12(3), 403-427.

Kazak, A. E., & Marvin, R. S. (1984). Differences, difficulties and adaptation: Stress and social networks in families with a handicapped child. Family Relations: Journal of Applied Family & Child Studies, 33(1), 67-77.

Krauss, M. W. (1993). Child-Related and Parenting Stress - Similarities and Differences Between Mothers and Fathers of Children with Disabilities. American Journal on Mental Retardation, 97(4), 393-404.

Lobato, D. (1985). Preschool siblings of handicapped children: Impact of peer support and training. Journal of Autism & Developmental Disorders, 15(3), 345-350.

Maclas, M. M., Roberts, K. M., Saylor, C. F., & Fussell, J. J. (2006). Toileting concerns, parenting stress, and behavior problems in children with special health care needs. Clinical Pediatrics, 45(5), 415-422.

Molfese, V., Rudasill, K., Beswick, J., Jacobi-Vessels, J., Ferguson, M., & White, J. (2010). Infant temperament, maternal personality, and parenting stress as contributors to infant developmental outcomes, Merrill-Palmer Quarterly, 56, 49-79.

Mott, S. E. (1986). Methods for assessing child and family outcomes in early childhood special education programs: Some views from the field. Topics in Early Childhood Special Education, 6(2), 1-15.

Pelchat, D., & Lefebvre, H. (2004). A holistic intervention programme for families with a child with a disability. Journal of Advanced Nursing, 48(2), 124-131. doi: 10.1111/j.1365-2648.2004.03179.x.

Quittner, A. L., Digirolamo, A. M., Michel, M., & Eigen, H. (1992). Parental Response to Cystic-Fibrosis - A Contextual Analysis of the Diagnosis Phase. Journal of Pediatric Psychology, 17(6), 683-704.

Richman, D., Belmont, J., Kim, M., Slavin, C., & Hayner, A. (2009). Parenting stress in families of children with Cornelia de Lange Syndrome and Down Syndrome, Journal of Developmental and Physical Disabilities, 21, 537-553.

Sarimski, K. (1997). Parenting Stress in Families with Craniofacially Disordered Children. Praxis Der Kinderpsychologie und Kinderpsychiatrie, 46(1), 2-14.

Sarimski, K. (1998). Stress in Mothers of Children with Genetic Syndromes. Zeitschrift fur Klinische Psychologie, Psychiatrie, und Psychotherapie, 46(3), 233-244.

Smith, T. B., Oliver, M. N., & Innocenti, M. S. (2001). Parenting stress in families of children with disabilities. American Journal of Orthopsychiatry, 71(2), 257-261.

Sontag, J. C. (1996). Toward a Comprehensive Theoretical Framework for Disability Research - Bronfenbrenner Revisited. Journal of Special Education, 30(3), 319-344.

Trute, B., & Hiebert-Murphy, D. (2005). Predicting family adjustment and parenting stress in childhood disability services using brief assessment tools. Journal of Intellectual & Developmental Disability, 30(4), 217-225. doi: 10.1080/13668250500349441.

Volenski, L. T. (1995). Building school support systems for parents of handicapped children: The parent education and guidance program. Psychology in the Schools, 32(2), 124-129.

Wells, R. D., & Schwebel, A. I. (1987). Chronically ill children and their mothers: Predictors of resilience and vulnerability to hospitalization and surgical stress. Journal of Developmental & Behavioral Pediatrics, 8(2), 83-89.

Family Support

Feldman, R., Granat, A., Pariente, C., Kanety, H., Kuint, J., & Gilboa-Schechtman, E. (2009). Maternal Depression and Anxiety Across the Postpartum Year and Infant Social Engagement, Fear Regulation, and Stress Reactivity. Journal of Amer Academy of Child & Adolescent Psychiatry, 48(9), 919 - 927.

Glenn, S. S., Cunningham, C. C., Poole, H. H., Reeves, D. D., & Weindling, M. M. (2009). Maternal parenting stress and its correlates in families with a young child with cerebral palsy. Child: Care, Health and Development, 35(1), 71-78.

Kim, H. (. K., Viner-Brown, S. I., & Garcia, J. (2007). Children's Mental Health and Family Functioning in Rhode Island. Pediatrics, 119(Supplement_1), S22-28. doi: 10.1542/peds.2006-2089E.

Pemberton, J. R., & Borrego, J. (2005). The relationship between treatment acceptability and familism. International Journal of Behavioral Consultation and Therapy, 1(4), 329-337.

Family Violence

DiLauro, M. D. (2004). Psychosocial Factors Associated with Types of Child Maltreatment. Child Welfare, 83(1), 69. doi: Article.

Dixon, L., Hamilton-Giachritsis, C., Browne, K., & Ostapuik, E. (2007). The Co-occurrence of Child and Intimate Partner Maltreatment in the Family: Characteristics of the Violent Perpetrators. Journal of Family Violence, 22(8), 675-689. doi: 10.1007/s10896-007-9115-x.

Edelson, M., Hokoda, A., & Ramos-Lira, L. (2007). Differences in Effects of Domestic Violence Between Latina and Non-Latina Women. Journal of Family Violence, 22(1), 1-10. doi: 10.1007/s10896-006-9051-1.

Holden, E. W., & Banez, G. A. (1996). Child abuse potential and parenting stress within maltreating families. Journal of Family Violence, 11(1), 1-12.

Holigrocki, R. J., & Hudson-Crain, R.(2004).Victim-victimizer relational dynamics as maintained by representational, defensive, and neurobiological functioning. Bulletin of the Menninger Clinic, 68(3), 197-212. doi: Article.

Huth-Bocks, A., & Hughes, H. (2008). Parenting Stress, Parenting Behavior, and Children's Adjustment in Families Experiencing Intimate Partner Violence. Journal of Family Violence, 23(4), 243-251. doi: 10.1007/s10896-007-9148-1.

Levendosky, A. A., & Graham-Bermann, S. A. (2001). Parenting in battered women: the effects of domestic violence on women and their children. Journal of Family Violence, 16(2), 171-192.

Lévesque, S., Clément, M., & Chamberland, C. (2007). Factors Associated with Co-occurrence of Spousal and Parental Violence: Quebec Population Study. Journal of Family Violence, 22(8), 661-674. doi: 10.1007/s10896-007-9106-y.

Lutzker, J. R., Bigelow, K. M., Doctor, R. M., & Kessler, M. L. (1998). Safety, health care, and bonding within an ecobehavioral approach to treating and preventing child abuse and neglect. Journal of Family Violence, 13(2), 163-185.

Milner, J. S. (1994). Assessing Physical Child-Abuse Risk - The Child-Abuse Potential Inventory. Clinical Psychology Review, 14(6), 547-583.

Mitchell, M. D., Hargrove, G. L., Collins, M. H., Thompson, M. P., Reddick, T. L., & Kaslow, N. J. (2006). Coping variables that mediate the relation between intimate partner violence and mental health outcomes among low-income, African American women. Journal of Clinical Psychology, 62(12), 1503-1520. doi: 10.1002/jclp.20305.

Moore, C. G., Probst, J. C., Tompkins, M., Cuffe, S., & Martin, A. B. (2007). The prevalence of violent disagreements in US families: effects of residence, race/ethnicity, and parental stress. Pediatrics, 119, 68-76.

Pithers, W. D., Gray, A., Busconi, A., & Houchens, P. (1998). Caregivers of children with sexual behavior problems: Psychological and familial functioning. Child Abuse & Neglect, 22(2), 129-141.

Probst, J., Jong-Yi Wang, Martin, A. B., Moore, C. G., Paul, B., & Samuels, M. E. (2008). Potentially Violent Disagreements and Parenting Stress Among American Indian/Alaska Native Families: Analysis Across Seven States.. Maternal & Child Health Journal, 12, 91-102. doi: 10.1007/s10995-008-0370-0.

Rudo, Z. H., Powell, D. S., & Dunlap, G. (1998). The Effects of Violence in the Home on Childrens Emotional, Behavioral, and Social Functioning - A Review of the Literature. Journal of Emotional and Behavioral Disorders, 6(2), 94-113.

Taft, A. J., Small, R., Hegarty, K. L., Lumley, J., Watson, L. F., & Gold, L. (2009). MOSAIC (Mothers' Advocates In the Community): protocol and sample description of a cluster randomised trial of mentor mother support to reduce intimate partner violence among pregnant of recent mothers. BMC Public Health, 9, 1-13. doi: 10.1186/1471-2458-9-159.

Tandon, S. D., Parillo, K. M., Jenkins, C., & Duggan, A. K. (2005). Formative Evaluation of Home Visitors' Role in Addressing Poor Mental Health, Domestic Violence, and Substance Abuse Among Low-Income Pregnant and Parenting Women.. Maternal & Child Health Journal, 9(3), 273-283. doi: 10.1007/s10995-005-0012-8.

Taylor, C. A., Guterman, N. B., Lee, S. J., & Rathouz, P. J. (2009). Intimate Partner Violence, Maternal Stress, Nativity, and Risk for Maternal Maltreatment of Young Children. American Journal of Public Health, 99(1), 175. doi: 10.2105/AJPH.2007.126722.

Whipple, E. E. (1999). Reaching Families with Preschoolers at Risk of Physical Child-Abuse - What Works. Families in Society-The Journal of Contemporary Human Services, 80(2), 148-160.

Foster Care

Cole, S. (2005). Infants in foster care: Relational and environmental factors affecting attachment. JOURNAL OF REPRODUCTIVE AND INFANT PSYCHOLOGY, 23, 43-61.

Cole, S. A. (2002). Security of Attachment of Infants in Foster Care. In Proceedings of the... National Symposium on Doctoral Research in Social Work (p. 12). Presented at the National Symposium on Doctoral Research in Social Work, Columbus, OH: Ohio State University.

Jacobsen, T., & Miller, L. J. (1998). Mentally Ill Mothers Who Have Killed - 3 Cases Addressing the Issue of Future Parenting Capability. Psychiatric Services, 49(5), 650-657.

Kelley, S. J. (1992). Parenting stress and child maltreatment in drug-exposed children. Child Abuse & Neglect, 16(3), 317-28.

Klee, L., Kronstadt, D., & Zlotnick, C. (1997). Foster care's youngest: A preliminary report. American Journal of Orthopsychiatry, 67(2), 290-299.

Timmer, S. G., Llrquiza, A. I., Herschell, A. D., McGrath, J. M., Zebell, N. M., Porter, A. L., et al. (2006). Parent-Child Interaction Therapy: Application of an Empirically Supported Treatment to Maltreated Children in Foster Care. Child Welfare, 85(6), 919. doi: Article.

Timmer, S. G., Sedlar, G., & Urquiza, A. J. (2004). Challenging children in kin versus nonkin foster care: perceived costs and benefits to caregivers. Child Maltreatment, 9(3), 251-262. doi: 10.1177/1077559504266998.

Timmer, S. G., Urquiza, A. J., & Zebell, N. (2006). Challenging foster caregiver–maltreated child relationships: The effectiveness of parent–child interaction therapy. Children and Youth Services Review, 28(1), 1-19. doi: 10.1016/j.childyouth.2005.01.006.

Whitley, D. M., White, K. R., Kelley, S. J., & Yorke, B. (1999). Strengths-Based Case-Management - The Application to Grandparents Raising Grandchildren. Families in Society-The Journal of Contemporary Human Services, 80(2), 110-119.

Gay and Lesbian Families

Bos, H. M. W., van Balen, F., & van den Boom, D. C. (2007). Child adjustment and parenting in planned lesbian-parent families. The American Journal of Orthopsychiatry, 77(1), 38-48. doi: 10.1037/0002-9432.77.1.38.

Bos, H. M., van Balen, F., & van den Boom, D. C. (2004). Experience of parenthood, couple relationship, social support, and child-rearing goals in planned lesbian mother families. Journal of Child Psychology and Psychiatry, 45(4), 755–764.

Chan, R. W., Raboy, B., & Patterson, C. J. (1998). Psychosocial Adjustment Among Children Conceived via Donor Insemination by Lesbian and Heterosexual Mothers. Child Development, 69(2), 443-457.

Dundas, S., & Kaufman, M. (2000). The Toronto Lesbian Family Study. Journal of Homosexuality, 40(2), 65-79.

Golombok, S., Perry, B., Burston, A., Murray, C., Mooney-Somers, J., Stevens, M., et al. (2003). Children with lesbian parents: a community study. Developmental Psychology, 39(1), 20-33.

Golombok, S., Tasker, F., & Murray, C. (1997). Children Raised in Fatherless Families from Infancy - Family Relationships and the Socioemotional Development of Children of Lesbian and Single Heterosexual Mothers. Journal of Child Psychology and Psychiatry and Allied Disciplines, 38(7), 783-791.

Grandparents as Primary Caregivers

Kelley, S. J., Yorker, B. C., Whitley, D. M., & Sipe, T. A. (2001). A Multimodal Intervention for Grandparents Raising Grandchildren: Results of an Exploratory Study. Child Welfare, 80(1), 27. doi: Article.

Incarceration

Loper, A. B. (2006). How Do Mothers in Prison Differ From Non-Mothers? Journal of Child & Family Studies, 15(1), 83-95. doi: 10.1007/s10826-005-9005-x.

Mackintosh, V. H., Myers, B. J., & Kennon, S. S. (2006). Children of Incarcerated Mothers and Their Caregivers: Factors Affecting the Quality of Their Relationship. Journal of Child & Family Studies, 15(5), 579-594. doi: 10.1007/s10826-006-9030-4.

“Latch-Key” Children

Hyde, J. S., Else-Quest, N. M., Goldsmith, H. H., & Biesanz, J. C. (2004). Children's Temperament and Behavior Problems Predict Their Employed Mothers' Work Functioning. Child Development, 75(2), 580-594. doi: 10.1111/j.1467-8624.2004.00694.x.

Leventhal-Belfer, L., Cowan, P. A., & Cowan, C. P. (1992). Satisfaction with Child-Care Arrangements - Effects on Adaptation to Parenthood. American Journal of Orthopsychiatry, 62(2), 165-177.

Lovko, A. M., & Ullman, D. G. (1989). Research on the adjustment of latchkey children: Role of background/demographic and latchkey situation variables. Journal of Clinical Child Psychology, 18, 16-24.

Military

Chartrand, M. M., Frank, D. A., White, L. F., & Shope, T. R. (2008). Effect of Parents' Wartime Deployment on the Behavior of Young Children in Military Families. Arch Pediatr Adolesc Med, 162(11), 1009-1014. doi: 10.1001/archpedi.162.11.1009.

Flake, E., Davis, B., Johnson, P., & Middleton, L. (2009). The Psychosocial Effects of Deployment on Military Children, Journal of Developmental & Behavioral Pediatrics, 30, 271-278.

Kelley, M. L., Herzog-Simmer, P. A., & Harris, M. A. (1994). Effects of military-induced separation on the parenting stress and family functioning of deploying mothers. Military Psychology, 6(2), 125-138.

Schaeffer, C. M., Alexander, P. C., Bethke, K., & Kretz, L. S. (2005). Predictors of Child Abuse Potential Among Military Parents: Comparing Mothers and Fathers. Journal of Family Violence, 20(2), 123-129. doi: 10.1007/s10896-005-3175-6.

Prediction of Marital Adjustment

Abidin, R. R., & Brunner, J. F. (1995). Development of a Parenting Alliance Inventory. Journal of Clinical Child Psychology, 24(1), 31-40.

Anastopoulos, A. D., Shelton, T. L., DuPaul, G. J., & Guevremont, D. C. (1993). Parent training for attention-deficit hyperactivity disorder: Its impact on parent functioning. Journal of Abnormal Child Psychology, 21(5).

Bigras, M., & Lafreniere, P. J. (1994). Influence of Psychosocial Risk, Marital Conflicts and Parental Stress on the Quality of Mother-Son and Mother-Daughter Interactions. Canadian Journal of Behavioural Science, 26(2), 280-297.

Bigras, M., Lafreniere, P., & Dumas, J. (1996). Discriminant validity of the parent and child scales of the parenting stress index. Early Education & Development, 7(2), 167-178.

Eddy, M. E., Carter, B. D., Kronenberger, W. G., Conradsen, S., Eid, N. S., Bourland, S. L., & Adams, G. (1998). Parent relationships and compliance in cystic fibrosis. Journal of Pediatric Health Care, 12(4), 196-202.

Kazui, M., Muto, T., & Sonoda, N. (1996). The roles of marital quality and parenting stress in mother-preschooler relationships. (Japanese). Japanese Journal of Developmental Psychology, 7(1), 31-40.

Kersh, J., Hedvat, T., Hauser-Cram, P., & Warfield, M. E. (2006). The contribution of marital quality to the well-being of parents of children with developmental disabilities. Journal of Intellectual Disability Research, 50(12), 883-893. doi: 10.1111/j.1365-2788.2006.00906.x.

Leung, S. S. L., Leung, C., & Chan, R. (2007). Perceived child behaviour problems, parenting stress, and marital satisfaction: comparison of new arrival and local parents of preschool children in Hong Kong. Hong Kong Medical Journal / Xianggang Yi Xue Za Zhi / Hong Kong Academy of Medicine, 13(5), 364-371.

Rimmerman, A., Turkel, L., & Crossman, R. (2003). Perception of child development, child-related stress and dyadic adjustment: pair analysis of married couples of young children with developmental disabilities. Journal of Intellectual & Developmental Disability, 28(2), 188. doi: Article.

Soliday, E., McCluskey-Fawcett, K., & O’Brien, M. (1999). Postpartum affect and depressive symptoms in mothers and fathers. American Journal of Orthopsychiatry, 69(1), 30-38.

Volling, B. L., Notaro, P. C., & Larsen, J. J. (1998). Adult Attachment Styles - Relations with Emotional Well-Being, Marriage, and Parenting. Family Relations, 47(4), 355-367.

Webster-Stratton, C. (1988). Mothers' and fathers' perceptions of child deviance: Roles of parent and child behaviors and parent adjustment. Journal of Consulting & Clinical Psychology, 56(6), 909-915.

Parent Disability

Adam, E. K., & Gunnar, M. R. (2001). Relationship functioning and home and work demands predict individual differences in diurnal cortisol patterns in women. Psychoneuroendocrinology, 26(2), 189-208.

Bos, H. M. W., van Balen, F., & van den Boom, D. C. (2007). Child adjustment and parenting in planned lesbian-parent families. The American Journal of Orthopsychiatry, 77(1), 38-48. doi: 10.1037/0002-9432.77.1.38.

Parent Personality

Damashek, A. L., Williams, N. A., Sher, K. J., Peterson, L., Lewis, T., & Schweinle, W. (2005). Risk for minor childhood injury: an investigation of maternal and child factors. Journal of Pediatric Psychology, 30(6), 469-480. doi: 10.1093/jpepsy/jsi072.

Dumas, J. E., Nissley, J., Nordstrom, A., Smith, E. P., Prinz, R. J., & Levine, D. W. (2005). Home Chaos: Sociodemographic, Parenting, Interactional, and Child Correlates. Journal of Clinical Child & Adolescent Psychology, 34(1), 93-104. doi: 10.1207/s15374424jccp3401_9.

Molfese, V., Rudasill, K., Beswick, J., Jacobi-Vessels, J., Ferguson, M., & White, J. (2010). Infant temperament, maternal personality, and parenting stress as contributors to infant developmental outcomes, Merrill-Palmer Quarterly, 56, 49-79.

Vermaes, I. P. R., Janssens, J. M. A. M., Mullaart, R. A., Vinck, A., & Gerris, J. R. M. (2008). Parents' personality and parenting stress in families of children with spina bifida. Child: Care, Health & Development, 34(5),665-674.

Parent Mental Illness

Brunette, M. F., Richardson, F., White, L., Bemis, G., & Eelkema, R. E. (2004). Integrated family treatment for parents with severe psychiatric disabilities. Psychiatric Rehabilitation Journal, 28(2), 177-180. doi: Article.

Dunne, L., Sneddon, H., Iwaniec, D., & Stewart, M. C. (2007). Maternal mental health and faltering growth in infants. Child Abuse Review, 16(5), 283-295.

Feldman, M. A., Varghese, J., Ramsay, J., & Rajska, D. (2002). Relationships between social support, stress and mother-child interactions in mothers with intellectual disabilities. Journal of Applied Research in Intellectual Disabiliites, 15(4), 314-323.

Mowbray, C. T., Bybee, D., Hollingsworth, L., Goodkind, S., & Oyserman, D. (2005). Living Arrangements and Social Support: Effects on the Well-Being of Mothers with Mental Illness. Social Work Research, 29(1), 41. doi: Article.

Mowbray, C., Oyserman, D., Bybee, D., & MacFarlane, P. (2002). Parenting of mothers with a serious mental illness: Differential effects of diagnosis, clinical history, and other mental health variables. Social Work Research, 26(4), 225. doi: Article.

Mullick, M., Miller, L. J., & Jacobsen, T. (2001). Insight into mental illness and child maltreatment risk among mothers with major psychiatric disorders. Psychiatric Services, 52(4), 488-492.

Newman, L. K., Stevenson, C. S., Bergman, L. R., & Boyce, P. (2007). Borderline personality disorder, mother-infant interaction and parenting perceptions: preliminary findings. Australian & New Zealand Journal of Psychiatry, 41(7), 598-605. doi: 10.1080/00048670701392833.

Plant, K., Byrne, L., Barkla, J., McLean, D., Hearle, J., & McGrath, J. (2002). Parents with Psychosis: A Pilot Study Examining Self-Report Measures Related to Family Functioning. Australian e-Journal for the Advancement of Mental Health, 1(1).

Tzang, R., Chang, Y., & Liu, S. (2009). The association between children's ADHD subtype and parenting stress and parental symptoms, International Journal of Psychiatry in Clinical Practice, 13, 318-325.

Parenting Adult Children

Hill, C., & Rose, J. (2009). Parenting stress in mothers of adults with an intellectual disability: parental cognitions in relation to child characteristics and family support. Journal of Intellectual Disability Research: JIDR. doi: 10.1111/j.1365-2788.2009.01207.x

Weiss, J. A., & Diamond, T. (2005). Stress in parents of adults with intellectual disabilities attending Special Olympics competitions. Journal of Applied Research in Intellectual Disabilities, 18(3), 263-270.

Prediction of Child Adjustment

Campis, L. B., DeMaso, D. R., & Twente, A. W. (1995). The role of maternal factors in the adaptation of children with craniofacial disfigurement. Cleft Palate Craniofacial Journal, 32(1), 55-61.

Carothers, S. S., Borkowski, J. G., & Whitman, T. L. (2006). Children of Adolescent Mothers: Exposure to Negative Life Events and the Role of Social Supports on Their Socioemotional Adjustment. Journal of Youth and Adolescence, 35(5), 822-832. doi: 10.1007/s10964-006-9096-8.

Colpin, H., & Soenen, S. (2002). Parenting and psychosocial development of IVF children: a follow-up study. Human Reproduction, 17(4), 1116-1123.

Dalla, R. L., & Gamble, W. C. (1997). Exploring Factors Related to Parenting Competence Among Navajo Teenage Mothers - Dual Techniques of Inquiry. Family Relations, 46(2), 113-121.

Day, C., & Davis, H. (2006). The effectiveness and quality of routine child and adolescent mental health care outreach clinics. British Journal of Clinical Psychology, 45(4), 439-452. doi: 10.1348/014466505X79986.

Dekovic, M., & Meeus, W. (1997). Peer Relations in Adolescence - Effects of Parenting and Adolescents Self-Concept. Journal of Adolescence, 20(2), 163-176.

Dekovic, M., & Rabotegsaric, Z. (1997). Parental Child-Rearing Practices and Adolescent Peer Relations. Drustvenaistraizivanja, 6(4-5), 427-445.

Dumas, J. E., & Wekerle, C. (1995). Maternal Reports of Child-Behavior Problems and Personal Distress as Predictors of Dysfunctional Parenting. Development and Psychopathology, 7(3), 465-479.

Flake, E., Davis, B., Johnson, P., & Middleton, L. (2009). The Psychosocial Effects of Deployment on Military Children, Journal of Developmental & Behavioral Pediatrics, 30, 271-278.

Goldberg, S., Janus, M., Washington, J., Simmons, R. J., Maclusky, I., & Fowler, R. S. (1997). Prediction of Preschool Behavioral-Problems in Healthy and Pediatric Samples. Journal of Developmental and Behavioral Pediatrics, 18(5), 304-313.

Huth-Bocks, A., & Hughes, H. (2008). Parenting Stress, Parenting Behavior, and Children's Adjustment in Families Experiencing Intimate Partner Violence. Journal of Family Violence, 23(4), 243-251. doi: 10.1007/s10896-007-9148-1.

Krueckeberg, S. M., & Kappsimon, K. A. (1993). Effect of Parental Factors on Social Skills of Preschool-Children with Craniofacial Anomalies. Cleft Palate-Craniofacial Journal, 30(5), 490-496.

Levendosky, A. A., & Graham-Bermann, S. A. (2001). Parenting in battered women: the effects of domestic violence on women and their children. Journal of Family Violence, 16(2), 171-192.

Rhodes, J. E., Ebert, L., & Fischer. (1992). Natural mentors: An overlooked resource in the social networks of young, African-American mothers. American Journal of Community Psychology, 20(4), 445-461.

Speltz, M. L., Endriga, M. C., Fisher, P. A., & Mason, C. A. (1997). Early Predictors of Attachment in Infants with Cleft-Lip and/or Palate. Child Development, 68(1), 12-25.

Wanamaker, C. E., & Glenwick, D. S. (1998). Stress, Coping, and Perceptions of Child-Behavior in Parents of Preschoolers with Cerebral-Palsy. Rehabilitation Psychology, 43(4), 297-312.

Wolf, L. C., Fisman, S., Ellison, D., & Freeman, T. (1998). Effect of Sibling Perception of Differential Parental Treatment in Sibling Dyads with One Disabled-Child. Journal of the American Academy of Child and Adolescent Psychiatry, 37(12), 1317-1325.

Single Parents

Chacko, A., Wymbs, B. T., Flammer, L. M., Pelham, W. E., Walker, K. S., Arnold, F. W., …Herbst, L.(2008). A pilot study of the feasibility and efficacy of the Strategies to Enhance Positive Parenting (STEPP) Program for single mothers of children with ADHD. Journal of Attention Disorders, 12(3), 270-280.

Copeland, S., & Harbaugh, B. L. (2005). Differences in parenting stress between married and single first time mothers at six to eight weeks after birth. Issues in Comprehensive Pediatric Nursing, 28(3), 139-152.

Jackson, A. P., Bentler, P. M., & Franke, T. M. (2008). Low-Wage Maternal Employment and Parenting Style. Social Work, 53(3), 267. doi: Article.

Murray, C., & Golombok, S. (2005). Solo mothers and their donor insemination infants: follow-up at age 2 years. Human Reproduction (Oxford, England), 20(6), 1655-1660. doi: 10.1093/humrep/deh823.

Unger, D. G., Jones, C. W., Park, E., & Tressell, P. A. (2001). Promoting Involvement Between Low-Income Single Caregivers and Urban Early Intervention Programs. Topics in Early Childhood Special Education, 21(4), 197. doi: Article.

Youngblut, J. M., Brooten, D., Singer, L. T., Standing, T., Lee, H., & Rodgers, W. L. (2001). Effects of maternal employment and prematurity on child outcomes in single parent families. Nursing Research, 50(6), 346-355.

Teenage Parents

Andreozzi, L., Flanagan, P., Seifer, R., Brunner, S., & Lester, B. (2002). Attachment classifications among 18-month-old children of adolescent mothers. Archives of Pediatrics & Adolescent Medicine, 156(1), 20-26.

Andresen, P. A., & Telleen, S. L. (1992). The Relationship Between Social Support and Maternal Behaviors and Attitudes - A Meta-Analytic Review. American Journal of Community Psychology, 20(6), 753-774.

Antshel, K. M., & Joseph, G. (2006). Maternal Stress in Nonverbal Learning Disorder: A Comparison With Reading Disorder. Journal of Learning Disabilities, 39(3), 194. doi: Article.

Barnet, B., Duggan, A. K., Devoe, M., & Burrell, L. (2002). The effect of volunteer home visitation for adolescent mothers on parenting and mental health outcomes: A randomized trial. Archives of Pediatrics & Adolescent Medicine, 156(12), 1216-1222.

Birkeland, R., Thompson, J. K., & Phares, V. (2005). Adolescent motherhood and postpartum depression. Journal of Clinical Child and Adolescent Psychology, 34(2), 292–300.

Black, M. M., & Nitz, K. (1996). Grandmother co-residence, parenting, and child development among low income, urban teen mothers. Journal of Adolescent Health, 18(3), 218-26.

Carothers, S. S., Borkowski, J. G., & Whitman, T. L. (2006). Children of Adolescent Mothers: Exposure to Negative Life Events and the Role of Social Supports on Their Socioemotional Adjustment. Journal of Youth and Adolescence, 35(5), 822-832. doi: 10.1007/s10964-006-9096-8.

Coleman, P. K., & Karraker, K. H. (1998). Self-Efficacy and Parenting Quality - Findings and Future Applications. Developmental Review, 18(1), 47-85.

Dalla, R. L., & Gamble, W. C. (1997). Exploring Factors Related to Parenting Competence Among Navajo Teenage Mothers - Dual Techniques of Inquiry. Family Relations, 46(2), 113-121.

Dukewich, T. L., Borkowski, J. G., & Whitman, T. L. (1996). Adolescent Mothers and Child-Abuse Potential - An Evaluation of Risk-Factors. Child Abuse & Neglect, 20(11), 1031-1047.

Dunham, P. J., Hurshman, A., Litwin, E., Gusella, J., Ellsworth, C., & Dodd, P. W. D. (1998). Computer-Mediated Social Support - Single Young Mothers as a Model System. American Journal of Community Psychology, 26(2), 281-306.

Fagan, J., Bernd, E., & Whiteman, V. (2007). Adolescent Fathers' Parenting Stress, Social Support, and Involvement with Infants. Journal of Research on Adolescence (Blackwell Publishing Limited), 17(1), 1-22. doi: 10.1111/j.1532-7795.2007.00510.x.

Florsheim, P., Moore, D., Zollinger, L., MacDonald, J., & Sumida, E. (1999). The transition to parenthood among adolescent fathers and their partners: Does antisocial behavior predict problems in parenting? Applied Developmental Science, 3(3), 178-191.

Fuscaldo, D., Kaye, J. W., & Philliber, S. (1998). Evaluation of a Program for Parenting. Families in Society:The Journal of Contemporary Human Services, 79(1), 53-61.

Gorzka, P. A. (1999). Homeless parents' perceptions of parenting stress. Journal of Child & Adolescent Psychiatric Nursing, 12(1), 7-16.

Holub, C. K., Kershaw, T. S., Ethier, K. A., Lewis, J. B., Milan, S., & Ickovics, J. R. (2007). Prenatal and Parenting Stress on Adolescent Maternal Adjustment: Identifying a High-Risk Subgroup.. Maternal & Child Health Journal, 11(2), 153-159. doi: 10.1007/s10995-006-0159-y.

Kelly, L. E. (1995). Adolescent mothers: what factors relate to level of preventive health care sought for their infants? Journal of Pediatric Nursing: Nursing Care of Children & Families, 10(2), 105-13.

Larson, N. C. (2004). Parenting stress among adolescent mothers in the transition to adulthood. Child & Adolescent Social Work Journal, 21(5), 457-476.

Miller, C. L., Miceli, P. J., Whitman, T. L., & Borkowski, J. G. (1996). Cognitive Readiness to Parent and Intellectual-Emotional Development in Children of Adolescent Mothers. Developmental Psychology, 32(3), 533-541.

Mylod, D. E., Whitman, T. L., & Borkowski, J. G. (1997). Predicting Adolescent Mothers Transition to Adulthood. Journal of Research on Adolescence, 7(4), 457-478.

Nitz, K., Ketterlinus, R. D., & Brandt, L. J. (1995). The role of stress, social support, and family environment in adolescent mothers' parenting. Journal of Adolescent Research, 10(3), 358-382.

Passino, A. W., Whitman, T. L., Borkowski, J. G., Schellenbach, C. J., Maxwell, S. E., Keogh, D., & Rellinger, E. (1993). Personal Adjustment During Pregnancy and Adolescent Parenting. Adolescence, 28(109), 97-122.

Richardson, R. A., Barbour, N. E., & Bubenzer, D. L. (1995). Peer relationships as a source of support for adolescent mothers. Journal of Adolescent Research, 10(2), 278-290.

Secco, M. L., & Moffatt, M. (2003). Situational, maternal, and infant influences on parenting stress among adolescent mothers. Issues in Comprehensive Pediatric Nursing, 26(2), 103-122.

Solis, M. L., & Abidin, R. R. (1991). The Spanish version Parenting Stress Index: A psychometric study. Journal of Clinical Child Psychology, 20(4), 372-378.

Stoiber, K. C., & Houghton, T. G. (1993). The Relationship of Adolescent Mothers Expectations, Knowledge, and Beliefs to Their Young Childrens Coping Behavior. Infant Mental Health Journal, 14(1), 61-79.

Stoiber, K. C., & Houghton, T. G. (1994). Adolescent mothers' cognitions and behaviors as at-risk indicators. School Psychology Quarterly, 9(4), 295-316.

Uno, D., Florsheim, P., & Uchino, B. N. (1998). Psychosocial Mechanisms Underlying Quality of Parenting Among Mexican-American and White Adolescent Mothers. Journal of Youth and Adolescence, 27(5), 585-605.

Fathers

Ahmann, E., Wulff, L., & Meny, R. G. (1992). Home apnea monitoring and disruptions in family life: a multidimensional controlled study. American Journal of Public Health, 82(5), 719-22.

Arditti, J. A., & Maddenderdich, D. (1997). Joint and Sole Custody Mothers - Implications for Research and Practice. Families in Society - The Journal of Contemporary Human Services, 78(1), 36-45.

Badr, L. K., Garg, M., & Kamath, M. (2006). Intervention for infants with brain injury: Results of a randomized controlled study. Infant Behavior and Development, 29(1), 80-90. doi: 10.1016/j.infbeh.2005.08.003.

Bagner, D. M., & Eyberg, S. M. (2003). Father involvement in parent training: When does it matter?. Journal of Clinical Child & Adolescent Psychology, 32(4), 599-605.

Baker, D. B. (1994). Parenting stress and ADHD: A comparison of mothers and fathers. Journal of Emotional & Behavioral Disorders, 2(1), 46-50.

Baker-Ericzén, M. J., Brookman-Frazee, L., & Stahmer, A. (2005). Stress levels and adaptability in parents of toddlers with and without autism spectrum disorders. Research and Practice for Persons with Severe Disabilities, 30(4), 194–204.

Beckman, P. J. (1991). Comparison of mothers' and fathers' perceptions of the effect of young children with and without disabilities. American Journal on Mental Retardation, 95(5), 585-595.

Burbach, A. D., Fox, R. A., & Nicholson, B. C. (2004). Challenging Behaviors in Young Children: The Father's Role. Journal of Genetic Psychology, 165(2), 169. doi: Article.

Colpin, H., DeMunter, A., Nys, K., & Vandemuelebroecke, L. (2000). Pre- and postnatal determinants of parenting stress in mothers of one-year-old twins. Marriage & Family Review, 30(1-2), 99-107.

Cowan, P. A., Cowan, C. P., Pruett, M. K., Pruett, K., & Wong, J. J. (2009). Promoting Fathers' Engagement With Children: Preventive Interventions for Low-Income Families. Journal of Marriage and Family, 71(3), 663-679. doi: 10.1111/j.1741-3737.2009.00625.x.

Darke, P. R., & Goldberg, S. (1994). Father-Infant interaction and parent stress with healthy and medically compromised infants. Infant Behavior & Development, 17(1), 3-14.

Deater-Deckard, K. (1998). Parenting Stress and Child Adjustment - Some Old Hypotheses and New Questions. Clinical Psychology - Science and Practice, 5(3), 314-332.

Deater-Deckard, K., & Scarr, S. (1996). Parenting Stress Among Dual-Earner Mothers and Fathers - Are There Gender Differences. Journal of Family Psychology, 10(1), 45-59.

Deater-Deckard, K., Scarr, S., McCartney, K., & Eisenberg, M. (1994). Paternal Separation Anxiety - Relationships with Parenting Stress, Child-Rearing Attitudes, and Maternal Anxieties. Psychological Science, 5(6), 341-346.

Fagan, J., Bernd, E., & Whiteman, V. (2007). Adolescent Fathers' Parenting Stress, Social Support, and Involvement with Infants. Journal of Research on Adolescence (Blackwell Publishing Limited), 17(1), 1-22. doi: 10.1111/j.1532-7795.2007.00510.x.

Florsheim, P., Moore, D., Zollinger, L., MacDonald, J., & Sumida, E. (1999). The transition to parenthood among adolescent fathers and their partners: Does antisocial behavior predict problems in parenting? Applied Developmental Science, 3(3), 178-191.

Gavin, L., & Wysocki, T. (2006). Associations of paternal involvement in disease management with maternal and family outcomes in families with children with chronic illness. Journal of Pediatric Psychology, 31(5), 481-489.

Girolametto, L., & Tannock, R. (1994). Correlates of Directiveness in the Interactions of Fathers and Mothers of Children with Developmental Delays. Journal of Speech and Hearing Research, 37(5), 1178-1191.

Gorzka, P. A. (1999). Homeless parents' perceptions of parenting stress. Journal of Child & Adolescent Psychiatric Nursing, 12(1), 7-16.

Harrison, M. J., & Magill-Evans, J. (1996). Mother and father interactions over the first year with term and preterm infants. Research in Nursing & Health, 19(6), 451-9.

Harvey, E. (1998). Parental Employment and Conduct Problems Among Children with Attention-Deficit/Hyperactivity Disorder - An Examination of Child-Care Workload and Parenting Well-Being as Mediating Variables. Journal of Social and Clinical Psychology, 17(4), 476-490.

Holmbeck, G. N., Goreyferguson, L., Hudson, T., Seefeldt, T., Shapera, W., Turner, T., & Uhler, J. (1997). Maternal, Paternal, and Marital Functioning in Families of Preadolescents with Spina-Bifida. Journal of Pediatric Psychology, 22(2), 167-181.

Jackson, A. P. (1999). The Effects of Nonresident Father Involvement on Single Black Mothers and Their Young-Children. Social Work, 44(2), 156-166.

Jarvis, P. A., & Creasey, G. L. (1991). Parental stress, coping, and attachment in families with an 18-month-old infant. Infant Behavior & Development, 14(4), 383-395.

Kelly, L. E. (1995). Adolescent mothers: what factors relate to level of preventive health care sought for their infants? Journal of Pediatric Nursing: Nursing Care of Children & Families, 10(2), 105-13.

Levin, R., & Banks, S. (1991). Stress in parents of children with epilepsy. Canadian Journal of Rehabilitation, 4(4), 229-38.

Magill-Evans, J., & Harrison, M. J. (1999). Parent-child interactions and development of toddlers born preterm. Western Journal of Nursing Research, 21(3), 292-307.

Mainemer, H., Gilman, L. C., & Ames, E. W. (1998). Parenting stress in families adopting children from Romanian orphanages. Journal of Family Issues, 19(2), 164-180.

McBride, B. A. (1991). Parental support programs and paternal stress: An exploratory study. Early Childhood Research Quarterly, 6(2), 137-140.

McKelvey, L. M., Whiteside-Mansell, L., Faldowski, R. A., Shears, J., Ayoub, C., & Hart, A. D. (2009). Validity of the short form of the parenting stress index for fathers of toddlers. Journal of Child and Family Studies, 18(1), 102-111.

Meadow-Orlans, K. P. (1994). Stress, support, and deafness: Perceptions of infants' mothers and fathers. Journal of Early Intervention, 18(1), 91-102.

Milgrom, J., & McCloud, P. (1996). Parenting Stress and Postnatal Depression. Stress Medicine, 12(3), 177-186.

Milner, J. S., & Murphy, W. D. (1995). Assessment of Child Physical and Sexual Abuse Offenders. Family Relations, 44(4), 478-488.

Robson, A. L. (1997). Low-Birth-Weight and Parenting Stress During Early-Childhood. Journal of Pediatric Psychology, 22(3), 297-311.

Schuhmann, E. M., Foote, R. C., Eyberg, S. M., Boggs, S. R., & Algina, J. (1998). Efficacy of Parent-Child Interaction Therapy - Interim-Report of a Randomized Trial with Short-Term Maintenance. Journal of Clinical Child Psychology, 27(1), 34-45.

Scott, B. S., Atkinson, L., Minton, H. L., & Bowman, T. (1997). Psychological Distress of Parents of Infants with Down-Syndrome. American Journal on Mental Retardation, 102(2), 161-171.

Scott, K., & Crooks, C. V. (2007). Preliminary Evaluation of an Intervention Program for Maltreating Fathers. Brief Treatment and Crisis Intervention, 7(3), 224-238. doi: 10.1093/brief-treatment/mhm007.

Soliday, E., McCluskey-Fawcett, K., & O'Brien, M. (1999). Postpartum affect and depressive symptoms in mothers and fathers. American Journal of Orthopsychiatry, 69(1), 30-38.

Tucker, S., Gross, D., Fogg, L., Delaney, K., & Lapporte, R. (1998). The long-term efficacy of a behavioral parent training intervention for families with 2-year-olds. Research in Nursing & Health, 21(3), 199-210.

Wanamaker, C. E., & Glenwick, D. S. (1998). Stress, Coping, and Perceptions of Child-Behavior in Parents of Preschoolers with Cerebral-Palsy. Rehabilitation Psychology, 43(4), 297-312.

Webster-Stratton, C. (1988). Mothers' and fathers' perceptions of child deviance: Roles of parent and child behaviors and parent adjustment. Journal of Consulting & Clinical Psychology, 56(6), 909-915.

Webster-Stratton, C., & Spitzer, A. (1996). Parenting a Young-Child with Conduct Problems - New Insights Using Qualitative Methods. Advances in Clinical Child Psychology, 18, 1-62.

Wiener, L. S., Vasquez, M. J., & Battles, H. B. (2001). Brief report: fathering a child living with HIV/AIDS: psychosocial adjustment and parenting stress. Journal of Pediatric Psychology, 26(6), 353-358.

Wolf, L. C., & Fisman, N. S. (1989). Psychological effects of parenting stress on parents of autistic children. Journal of Autism & Developmental Disorders, 19(1), 157-166.

Wysocki, T., & Gavin, L. (2004). Psychometric Properties of a New Measure of Fathers' Involvement in the Management of Pediatric Chronic Diseases. J. Pediatr. Psychol., 29(3), 231-240. doi: 10.1093/jpepsy/jsh024.

Forensic

Archer, R. P., Buffington-Vollum, J. K., Stredny, R. V., & Handel, R. W. (2006). A survey of psychological test use patterns among forensic psychologists. Journal of Personality Assessment, 87(1), 84-94. doi: 10.1207/s15327752jpa8701_07.

Bow, J. N., & Quinnell, F. A. (2002). A critical review of child custody evaluation reports. Family Court Review, 40(2), 164-176. doi: 10.1111/j.174-1617.2002.tb00827.x.

Harnett, P. H. (2007). A procedure for assessing parents' capacity for change in child protection cases. Children and Youth Services Review, 29(9), 1179-1188. doi: 10.1016/j.childyouth.2007.04.005.

Ireland, J. L. (2008). Psychologists as witnesses: background and good practice in the delivery of evidence. Educational Psychology in Practice, 24(2), 115-127. doi: 10.1080/02667360802019172.

Health Care

Bryan, T., Burstein, L., Chao, P., & Ergul, C. (2006). The relationship between health status, language development, and behavior in young children. Physical Disabilities: Education and Related Services, 24(2), 7-19.

DeCaro, J. A., & Worthman, C. M. (2008). Return to school accompanied by changing associations between family ecology and cortisol. Developmental Psychobiology, 50(2), 183–195.

Apneic

Ahmann, E., Wulff, L., & Meny, R. G. (1992). Home apnea monitoring and disruptions in family life: a multidimensional controlled study. American Journal of Public Health, 82(5), 719-22.

Bendell, D., Goldberg, M. S., Urbano, M. T., & Urbano, R. C. (1987). Differential impact of parenting sick infants. Infant Mental Health Journal, 8(1), 28-36.

Mattie-Luksic, M., Javornisky, G., & DiMario, F. J. (2000). Assessment of stress in mothers of children with severe breath-holding spells. Pediatrics, 106(1 Pt 1), 1-5.

Phipps, S., & Drotar, D. (1990). Determinants of parenting stress in home apnea monitoring. Journal of Pediatric Psychology, 15(3), 385-399.

Taylor, H. G., Klein, N., Schatschneider, C., & Hack, M. (1998). Predictors of Early School-Age Outcomes in Very-Low-Birth-Weight Children. Journal of Developmental and Behavioral Pediatrics, 19(4), 235-243.

Asthma

Berz, J. B., Carter, A. S., Wagmiller, R. L., Horwitz, S. M., Murdock, K. K., & Briggs-Gowan, M. (2007). Prevalence and Correlates of Early Onset Asthma and Wheezing in a Healthy Birth Cohort of 2- to 3-Year Olds. J. Pediatr. Psychol., 32(2), 154-166. doi: 10.1093/jpepsy/jsj123.

Carson, D. K., & Schauer, R. W. (1992). Mothers of children with asthma: Perceptions of parenting stress and the mother-child relationship. Psychological Reports, 71(3, Pt 2), 1139-1148.

DeMore, M., Adams, C., Wilson, N., & Hogan, M. B. (2005). Parenting Stress, Difficult Child Behavior, and Use of Routines in Relation to Adherence in Pediatric Asthma. Children's Health Care, 34(4), 245-259. doi: 10.1207/s15326888chc3404_1.

Fagnano, M., van Wijngaarden, E., Connolly, H. V., Carno, M. A., Forbes-Jones, E., & Halterman, J. S. (2009). Sleep-Disordered Breathing and Behaviors of Inner-City Children With Asthma. Pediatrics, 124(1), 218-225. doi: 10.1542/peds.2008-2525.

Fedele, D., Grant, D., Wolfe-Christensen, C., Mullins, L., & Ryan, J. (2010). An examination of the factor structure of parenting capacity measures in chronic illness populations, Journal of Pediatric Psychology, 35, 1083-1092.

Halterman, J. S., Borrelli, B., Tremblay, P., Conn, K. M., Fagnano, M., Montes, G., et al. (2008). Screening for Environmental Tobacco Smoke Exposure Among Inner-City Children With Asthma. PEDIATRICS, 122(6), 1277-1283. doi: 10.1542/peds.2008-0104.

Markson, S., & Fiese, B. H. (2000). Family rituals as a protective factor for children with asthma. Journal of Pediatric Psychology, 25(7), 471-80.

Mrazek, D. A., Mrazek, P., & Klinnert, M. (1995). Clinical-Assessment of Parenting. Journal of the American Academy of Child and Adolescent Psychiatry, 34(3), 272-282.

Mullins, L. L., Wolfe-Christensen, C., Hoff Pai, A. L., Carpentier, M. Y., Gillaspy, S., Cheek, J., & Page, M. (2007). The relationship of parental overprotection, perceived child vulnerability, and parenting stress to uncertainty in youth with chronic illness. Journal of Pediatric Psychology, 32(8),973-982.

Weinreb, L., Goldberg, R., Bassuk, E., & Perloff, J. (1998). Determinants of Health and Service Use Patterns in Homeless and Low-Income Housed Children. Pediatrics, 102(3), 554-562.

Childhood Cancer

Fedele, D., Grant, D., Wolfe-Christensen, C., Mullins, L., & Ryan, J. (2010). An examination of the factor structure of parenting capacity measures in chronic illness populations, Journal of Pediatric Psychology, 35, 1083-1092.

Hung, J. W., Wu, Y., & Yeh, C. (2004). Comparing stress levels of parents of children with cancer and parents of children with physical disabilities. Psycho-Oncology, 13(12), 898-903.

Roddenberry, A., & Renk, K. (2008). Quality of Life in Pediatric Cancer Patients: The Relationships Among Parents' Characteristics, Children's Characteristics, and Informant Concordance. Journal of Child & Family Studies, 17(3), 402-426. doi: 10.1007/s10826-007-9155-0.

Streisnad, R., Braniecki, S., Tercyak, K. P., & Kazak, A. E. (2001). Childhood illness-related parenting stress: the pediatric inventory for parents. Journal of Pediatric Psychology, 26(3), 155-162.

Children's Health Care

Abidin, R. R. (1983). Parenting stress and the utilization of pediatric services. Children's Health Care, 11(2), 70-3.

Abidin, R. R., & Wilfong, E. (1989). Parenting stress and its relationship to child health care. Children's Health Care, 18(2), 114-6.

Allen, K. D., Maguire, K. B., Williams, G. E., & Sanger, W. G. (1996). The effects of infertility on parent-child relationships and adjustment. Children's Health Care, 25(2), 93-105.

Berz, J. B., Carter, A. S., Wagmiller, R. L., Horwitz, S. M., Murdock, K. K., & Briggs-Gowan, M. (2007). Prevalence and Correlates of Early Onset Asthma and Wheezing in a Healthy Birth Cohort of 2- to 3-Year Olds. J. Pediatr. Psychol., 32(2), 154-166. doi: 10.1093/jpepsy/jsj123.

Carlton-Conway, D., Ahluwalia, R., Henry, L., Michie, C., Wood, L., & Tulloh, R. (2005). Behaviour sequelae following acute Kawasaki disease. BMC Pediatrics, 5(1), 14. doi: 10.1186/1471-2431-5-14.

Caserta, M. T., O'Connor, T. G., Wyman, P. A., Wang, H., Moynihan, J., Cross, W., et al. (2008). The associations between psychosocial stress and the frequency of illness, and innate and adaptive immune function in children☆. Brain, Behavior, and Immunity, 22(6), 933-940. doi: 10.1016/j.bbi.2008.01.007.

Cohn, E. S., & Cermak, S. A. (1998). Including the Family Perspective in Sensory Integration Outcomes Research. American Journal of Occupational Therapy, 52(7), 540-546.

Gelman, V. S., & King, N. J. (2001). Wellbeing of Mothers with Children Exhibiting Sleep Disturbance. Australian Journal of Psychology, 53(1), 18-22.

Greer, A. J., Gulotta, C. S., Masler, E. A., & Laud, R. B. (2008). Caregiver Stress and Outcomes of Children with Pediatric Feeding Disorders Treated in an Intensive Interdisciplinary Program. J. Pediatr. Psychol., 33(6), 612-620. doi: 10.1093/jpepsy/jsm116.

Hoffmann, R. G., III, Rodrigue, J. R., Andres, J. M., & Novak, D. A. (1995). Moderating effects of family functioning on the social adjustment of children with liver disease. Children's Health Care, 24(2), 107-17.

Hutcheson, J. J., & Black, M. M. (1996). Psychometric properties of the Parenting Stress Index in a sample of low-income African-American mothers of infants and toddlers. Early Education & Development, 7(4), 381-400.

Ievers-Landis, C. E., Storfer-Isser, A., Rosen, C., Johnson, N. L., & Redline, S. (2008). Relationship of Sleep Parameters, Child Psychological Functioning, and Parenting Stress to Obesity Status Among Preadolescent Children. Journal of Developmental & Behavioral Pediatrics, 29(4), 243-252. doi: 10.1097/DBP.0b013e31816d923d.

Kelly, L. E. (1995). Adolescent mothers: what factors relate to level of preventive health care sought for their infants? Journal of Pediatric Nursing: Nursing Care of Children & Families, 10(2), 105-13.

Levin, R., & Banks, S. (1991). Stress in parents of children with epilepsy. Canadian Journal of Rehabilitation, 4(4), 229-38.

Lutzker, J. R., Bigelow, K. M., Doctor, R. M., & Kessler, M. L. (1998). Safety, health care, and bonding within an ecobehavioral approach to treating and preventing child abuse and neglect. Journal of Family Violence, 13(2), 163-185.

Musil, C. M. (1998). Health, stress, coping, and social support in grandmother caregivers. Health Care for Women International, 19(5), 441-55.

Ostberg, M. (1998). Parental stress, psychosocial problems and responsiveness in help-seeking parents with small (2-45 months old) children. Acta Paediatrica, 87(1), 69-76.

Ruckart, P. Z., Kakolewski, K., Bove, F. J., & Kaye, W. E. (2004). Long-Term Neurobehavioral Health Effects of Methyl Parathion Exposure in Children in Mississippi and Ohio. Environmental Health Perspectives, 112(1), 46-51. doi: Article.

Stapleton, S. R., Drummond, J., Kysela, G. M., McDonald, L., Alexander, J., & Fleming, D. (1996). Team-building: making collaborative practice work. Risk and resiliency in two samples of Canadian families. Journal of Nurse-Midwifery, 4(1), 117-51.

Surkan, P. J., Zhang, A., Trachtenberg, F., Daniel, D. B., McKinlay, S., & Bellinger, D. C. (2007). Neuropsychological function in children with blood lead levels< 10μg/dL. Neurotoxicology, 28(6), 1170–1177.

Waisbren, S. E., Rones, M., Read, C. Y., Marsden, D., & Levy, H. L. (2004). Brief report: Predictors of parenting stress among parents of children with biochemical genetic disorders. Journal of Pediatric Psychology, 29(7), 565-570.

Wake, M., Morton-Allen, E., Poulakis, Z., Hiscock, H., Gallagher, S., & Oberklaid, F. (2006). Prevalence, stability, and outcomes of cry-fuss and sleep problems in the first 2 years of life: prospective community-based study. Pediatrics, 117(3), 836.

Wulffaert, J., Scholte, E. M., Dijkxhoorn, Y. M., Bergman, J. E., van Ravenswaaij-Arts, C. M., & van Berckelaer-Onnes, I. A. (2009). Parenting stress in CHARGE syndrome and the relationship with child characteristics. Journal of Developmental and Physical Disabilities, 21(4), 301-313.

Wysocki, T., & Gavin, L. (2004). Psychometric Properties of a New Measure of Fathers' Involvement in the Management of Pediatric Chronic Diseases. J. Pediatr. Psychol., 29(3), 231-240. doi: 10.1093/jpepsy/jsh024.

Zeitz, P., Kakolewski, K., Imtiaz, R., & Kaye, W. (2002). Methods of assessing neurobehavioral development in children exposed to methyl parathion in Mississippi and Ohio. Environmental Health Perspectives, 110 Suppl 6, 1079-1083.

Chronic Pain

Eccleston, C., Malleson, P. N., Clinch, J., Connell, H., & Sourbut, C. (2003). Chronic pain in adolescents: evaluation of a programme of interdisciplinary cognitive behaviour therapy. Archives of Disease in Childhood, 88(10), 881-885.

Diabetes

Chisholm, V., Atkinson, L., Donaldson, C., Noyes, K., Payne, A., & Kelnar, C. (2007). Predictors of treatment adherence in young children with type 1 diabetes. Journal of Advanced Nursing, 57(5), 482-493. doi: 10.1111/j.1365-2648.2006.04137.x.

Fedele, D., Grant, D., Wolfe-Christensen, C., Mullins, L., & Ryan, J. (2010). An examination of the factor structure of parenting capacity measures in chronic illness populations, Journal of Pediatric Psychology, 35, 1083-1092.

Fox, L. A., Buckloh, L. M., Smith, S. D., Wysocki, T., & Mauras, N. (2005). A randomized controlled trial of insulin pump therapy in young children with type 1 diabetes. Diabetes Care, 28(6), 1277.

Hauenstein, E. J., Marvin, R. S., Snyder, A. L., & Clarke, W. L. (1989). Stress in parents of children with diabetes mellitus. Diabetes Care, 12(1), 18-23.

Mullins, L. L., Wolfe-Christensen, C., Hoff Pai, A. L., Carpentier, M. Y., Gillaspy, S., Cheek, J., & Page, M. (2007). The relationship of parental overprotection, perceived child vulnerability, and parenting stress to uncertainty in youth with chronic illness. Journal of Pediatric Psychology, 32(8),973-982.

Phillip, M., Battelino, T., Rodriguez, H., Danne, T., & Kaufman, F. (2007). Use of Insulin Pump Therapy in the Pediatric Age-Group. Diabetes Care, 30(6), 1653-1662. doi: 10.2337/dc07-9922.

Powers, S. W., Byars, K. C., Mitchell, M. J., Patton, S. R., Standiford, D. A., & Dolan, L. M. (2002). Parent report of mealtime behavior and parenting stress in young children with type 1 diabetes and in healthy control subjects. Diabetes Care, 25(2), 313-318.

Sullivan-Bolyai, S., Deatrick, J., Gruppuso, P., Tamborlane, W., & Grey, M. (2002). Mothers' Experiences Raising Young Children With Type 1 Diabetes. Journal for Specialists in Pediatric Nursing, 7(3), 93. doi: Article.

Wysocki, T., Huxtable, K., Linscheid, T. R., & Wayne, W. (1989). Adjustment to diabetes mellitus in preschoolers and their mothers. Diabetes Care, 12(8), 524-9.

Yu, M. S., Norris, J. M., Mitchell, C. M., Butler-Simon, N., Groshek, M., Follansbee, D., Erlich, H., Rewers, M., & Klingensmith, G. J. (1999). Impact on maternal parenting stress of receipt of genetic information regarding risk of diabetes in newborn infants. American Journal of Medical Genetics, 86(3), 219-26.

Feeding Issues

Pedersen S. D., Parsons, H. G., & Dewey, D. (2004). Stress levels experienced by the parents of enterally fed children. Chld: Care, Health & Development, 30(5), 507-513.

Epilepsy

Button, S., Pianta, R. C., & Marvin, R. S. (2001). Mothers' representations of relationships with their children: Relations with parenting behavior, mother characteristics, and child disability status. Social Development, 10(4), 455-472. doi: 10.1111/1467-9507.00175

Camfield, C., Breau, L., & Camfield, P. (2001). Impact of pediatric epilepsy on the family: a new scale for clinical and research use. Epilepsia, 42(1), 104-12.

Levin, R., & Banks, S. (1991). Stress in parents of children with epilepsy. Canadian Journal of Rehabilitation, 4(4), 229-38.

Rodenburg, R., Meijer, A. M., Deković, M., & Aldenkamp, A. P. (2007). Parents of children with enduring epilepsy: Predictors of parenting stress and parenting. Epilepsy and Behavior, 11(2), 197–207.

Failure to Thrive

Black, M. M. (1995). Failure-to-Thrive - Strategies for Evaluation and Intervention. School Psychology Review, 24(2), 171-185.

Dunne, L., Sneddon, H., Iwaniec, D., & Stewart, M. C. (2007). Maternal mental health and faltering growth in infants. Child Abuse Review, 16(5), 283-295.

Hutcheson, J. J., Black, M. M., & Starr, R. H. (1993). Developmental Differences in Interactional Characteristics of Mothers and Their Children with Failure-to-Thrive. Journal of Pediatric Psychology, 18(4), 453-466.

Singer, L. T., Song, L.-y., Hill, B. P., & Jaffe, A. C. (1990). Stress and depression in mothers of failure-to-thrive children. Journal of Pediatric Psychology, 15(6), 711-720.

Fragile X Syndrome

Johnston, C., Hessl, D., Blasey, C., Eliez, S., Erba, H., Dyer-Friedman, J.,…Reiss, A. L. (2003). Factors associated with parenting stress in mothers of children with fragile X syndrome. Journal of Developmental and Behavioral Pediatrics, 24(4), 267-275.

Wheeler, A., Hatton, D., Reichardt, A., & Bailey, D. (2007). Correlates of maternal behaviours in mothers of children with fragile X syndrome. Journal of Intellectual Disability Research, 51(6), 447-462. doi: 10.1111/j.1365-2788.2006.00896.x.

Functional Somatic Symptoms

Janssens, K. A., Oldehinkel, A. J., & Rosmalen, J. G. (2009). Parental overprotection predicts the development of functional somatic symptoms in young adolescents. The Journal of Pediatrics, 154(6), 918-923.

Wolff, N., Darlington, A., Hunfeld, J., Verhulst, F., Jaddoe, V., Hofman, A., et al. (2009). Determinants of Somatic Complaints in 18-month-old Children: The Generation R Study. J. Pediatr. Psychol.. doi: 10.1093/jpepsy/jsp058

HIV/AIDS

Bauman, L. J., Camacho, S., Westbrook, L., & Forbes-Jones, E. (1997). Correlates of personal stigma and social stigma among mothers with HIV/AIDS : National Conference of Women With HIV.

Hochhauser, C. J., Gaur, S., Marone, R., & Lewis, M. (2008). The impact of environmental risk factors on HIV-associated cognitive decline in children. AIDS Care, 20(6), 692-699. doi: 10.1080/09540120701693982.

Marhefka, S. L., Tepper, V. J., Brown, J. L., & Farley, J. J. (2006). Caregiver psychosocial characteristics and children's adherence to antiretroviral therapy. AIDS Patient Care & STDs, 20(6), 429-437.

Murphy, D., Marelich, W., Armistead, L., Herbeck, D. and Payne, D. (2010). Anxiety/stress among mothers living with HIV: effects on parenting skills and child outcomes, AIDS Care, 22, 1449 – 1458.

Potterton, J., Stewart, A., & Cooper, P. (2007). Parenting stress of caregivers of young children who are HIV positive. African Journal of Psychiatry, 10(4), 210-214.

Wiener, L. S., Vasquez, M. J., & Battles, H. B. (2001). Brief report: fathering a child living with HIV/AIDS: psychosocial adjustment and parenting stress. Journal of Pediatric Psychology, 26(6), 353-358.

Hospitalization

Bloom, A. A., Wright, J. A., Morris, R. D., Campbell, R. M., & Krawiecki, N. S. (1997). Additive Impact of In-hospital Cardiac Arrest on the Functioning of Children With Heart Disease. Pediatrics, 99(3), 390-398.

Small, F., Alderdice, F., McCusker, C., Stevenson, M., & Stewart, M. (2005). A prospective cohort study comparing hospital admission for gastro-enteritis with home management. Child: Care, Health & Development, 31(5), 555-562. doi: 10.1111/j.1365-2214.2005.00550.x.

Wells, R. D., & Schwebel, A. I. (1987). Chronically ill children and their mothers: Predictors of resilience and vulnerability to hospitalization and surgical stress. Journal of Developmental & Behavioral Pediatrics, 8(2), 83-89.

Youngblut, J. M., & Brooten, D. (2006). Pediatric head trauma: parent, parent-child, and family functioning 2 weeks after hospital discharge. Journal of Pediatric Psychology, 31(6), 608-618.

Youngblut, J. M., & Brooten, D. (2008). Mother's mental health, mother-child relationship, and family functioning 3 months after a preschooler's head injury. The Journal of Head Trauma Rehabilitation, 23(2), 92-102. doi: 10.1097/01.HTR.0000314528.85758.30.

Infant Colic

Miller-Loncar, C., Bigsby, R., High, P., Wallach, M., & Lester, B. (2004). Infant colic and feeding difficulties. Archives of Disease in Childhood, 89(10), 908.

Stifter, C. A., Bono, M., & Spinrad, T. (2003). Parent characteristics and conceptualizations associated with the emergence of infant colic. Journal of Reproductive & Infant Psychology, 21(4), 309-322. doi: 10.1080/02646830310001622123.

Nocturnal Enuresis

Chang, S. S. Y., Ng, C. F. N., & Wong, S. N. (2002). Behavioural problems in children and parenting stress associated with primary nocturnal enuresis in Hong Kong. Acta Paediatrica, 91(4), 475-479.

De Bruyne, E., Van Hoecke, E., Van Gompel, K., Verbeken, S., Baeyens, D., et al. (2009). Problem Behavior, Parental Stress and Enuresis. The Journal of Urology, 182(4), 2015-2021. doi: 10.1016/j.juro.2009.05.102

Seabrook, J. A., Gorodzinsky, F., & Freedman, S. (2005). Treatment of primary nocturnal enuresis: A randomized clinical trial comparing hypnotherapy and alarm therapy. Paediatrics & Child Health, 10(10), 609-610.

Motor Impairment/Cerebral Palsy

Button, S., Pianta, R. C., & Marvin, R. S. (2001). Partner support and maternal stress in families raising young children with cerebral palsy. Journal of Developmental & Physical Disabilities, 13(1), 61-81.

Glenn, S. S., Cunningham, C. C., Poole, H. H., Reeves, D. D., & Weindling, M. M. (2009). Maternal parenting stress and its correlates in families with a young child with cerebral palsy. Child: Care, Health and Development, 35(1), 71-78.

Msall, M. E., Rogers, B. T., Ripstein, H., Lyon, N., & Wilczenski, F. (1997). Measurements of Functional Outcomes in Children with Cerebral-Palsy. Mental Retardation and Developmental Disabilities Research Reviews, 3(2), 194-203.

Ong, L. C., Afifah, I., Sofiah, A., & Lye, M. S. (1998). Parenting stress among mothers of Malaysian children with cerebral palsy: predictors of child- and parent-related stress. Annals of Tropical Paediatrics, 18(4), 301-7.

Sheeran, T., Marvin, R. S., & Pianta, R. C. (1997). Mothers' resolution of their child's diagnosis and self-reported measures of parenting stress, marital relations, and social support. Journal of Pediatric Psychology, 22(2), 197-212.

Wanamaker, C. E., & Glenwick, D. S. (1998). Stress, Coping, and Perceptions of Child-Behavior in Parents of Preschoolers with Cerebral-Palsy. Rehabilitation Psychology, 43(4), 297-312.

Wang, H., & Jong, Y. (2004). Parental Stress and Related Factors in Parents of Children with Cerebral Palsy. The Kaohsiung Journal of Medical Sciences, 20(7), 334-340. doi: 10.1016/S1607-551X(09)70167-6.

Weiss, K. L., Marvin, R. S., & Pianta, R. C. (1997). Ethnographic detection and description of family strategies for child care: Applications to the study of cerebral palsy. Journal of Pediatric Psychology, 22(2), 263-278.

Wells, R. D., & Schwebel, A. I. (1987). Chronically ill children and their mothers: Predictors of resilience and vulnerability to hospitalization and surgical stress. Journal of Developmental & Behavioral Pediatrics, 8(2), 83-89.

White-Koning, M., Arnaud, C., Dickinson, H. O., Thyen, U., Beckung, E., Fauconnier, J.,…Colver, A. (2007). Determinants of child-parent agreement in quality-of-life reports: a European study of children with cerebral palsy. Pediatrics, 120(4), e804-814. doi: 10.1542/peds.2006-3272

Otitis Media

Forgays, D. K., Hasazi, J. E., & Wasserman, R. C. (1992). Recurrent otitis media and parenting stress in mothers of two-year-old. Journal of Developmental & Behavioral Pediatrics, 13(5), 321-325.

McCallum, M. S., & McKim, M. K. (1999). Recurrent otitis media and attachment security: A path model. Early Education and Development, 10(4), 517-534.

Minter, K. R., Roberts, J. E., Hooper, S. R., Burchinal, M. R., & Zeisel, S. A. (2001). Early childhood otitis media in relation to children's attention-related behavior in the first six years of life. Pediatrics, 107(5), 1037-1042.

Paradise, J. L., Campbell, T. F., Dollaghan, C. A., Feldman, H. M., Bernard, B. S., Colborn, D. K., et al. (2005). Developmental outcomes after early or delayed insertion of tympanostomy tubes. The New England Journal of Medicine, 353(6), 576.

Paradise, J. L., Dollaghan, C. A., Campbell, T. F., Feldman, H. M., Bernard, B. S., Colborn, D. K., …Smith, C. G. (2003). Otitis media and tympanostomy tube insertion during the first three years of life: developmental outcomes at the age of four years. Pediatrics, 112(2), 265-277.

Paradise, J. L., Feldman, H. M., Campbell, T. F., Dollaghan, C. A., Colborn, D. K., Bernard, B. S., et al. (2001). Effect of early or delayed insertion of tympanostomy tubes for persistent otitis media on developmental outcomes at the age of three years. The New England Journal of Medicine, 344(16), 1179-1187.

Paradise, J. L., Feldman, H. M., Colborn, D. K., Campbell, T. F., Dollaghan, C. A., Rockette, H. E., Janosky, J. E., Kurs-Lasky, M., Bernard, B. S., & Smith, C. G. (1999). Parental stress and parent-rated child behavior in relation to otitis media in the first three years of life. Pediatrics, 104(6), 1264-73.

Weinreb, L., Goldberg, R., Bassuk, E., & Perloff, J. (1998). Determinants of Health and Service Use Patterns in Homeless and Low-Income Housed Children. Pediatrics, 102(3), 554-562.

Parent's Health

Adam, E. K., & Gunnar, M. R. (2001). Relationship functioning and home and work demands predict individual differences in diurnal cortisol patterns in women. Psychoneuroendocrinology, 26(2), 189-208.

Physical Disability

Dempsey, I., & Keen, D. (2008). A Review of Processes and Outcomes in Family-Centered Services for Children With a Disability. Topics in Early Childhood Special Education, 28(1), 42-52. doi: 10.1177/0271121408316699.

Hung, J. W., Wu, Y., & Yeh, C. (2004). Comparing stress levels of parents of children with cancer and parents of children with physical disabilities. Psycho-Oncology, 13(12), 898-903.

Miller, A. C., Cate, I. M. P., & Johann-Murphy, M. (2001). When Chronic Disability Meets Acute Stress: Psychological and Functional Changes. Developmental Medicine & Child Neurology, 43(3), 214-216.

Pelchat, D., & Lefebvre, H. (2004). A holistic intervention programme for families with a child with a disability. Journal of Advanced Nursing, 48(2), 124-131. doi: 10.1111/j.1365-2648.2004.03179.x.

Premature Infants/Low Birth Weight

Als, H., & Gilkerson, L. (1997). The Role of Relationship-Based Developmentally Supportive Newborn Intensive-Care in Strengthening Outcome of Preterm Infants. Semeinars in Perinatology, 21(3), 178-189.

Badr, L. K., Bookheimer, S., Purdy, I., & Deeb, M. (2009). Predictors of neurodevelopmental outcome for preterm infants with brain injury: MRI, medical and environmental factors. Early human development, 85(5), 279–284.

Badr, L. K., Garg, M., & Kamath, M. (2006). Intervention for infants with brain injury: Results of a randomized controlled study. Infant Behavior and Development, 29(1), 80-90. doi: 10.1016/j.infbeh.2005.08.003.

Barrera, M. E., & Kitching, K. J. (1991). A 3-year early home intervention follow-up study with low birthweight infants and their parents. Topics in Early Childhood Special Education, 10(4), 14-28.

Bendell, D., Goldberg, M. S., Urbano, M. T., & Urbano, R. C. (1987). Differential impact of parenting sick infants. Infant Mental Health Journal, 8(1), 28-36.

Browne, J. V., & Talmi, A. (2005). Family-based intervention to enhance infant-parent relationships in the neonatal intensive care unit. Journal of Pediatric Psychology, 30(8), 667-677.

Douglas, J. E., & Bryon, M. (1996). Interview Data on Severe Behavioral Eating Difficulties in Young-Children. Archives of Disease in Childhood, 75(4), 304-308.

Doussard-Roosevelt, J. A., Porges, S. W., Scanlon, J. W., Alemi, B., & Scanlon, K. B. (1997). Vagal regulation of heart rate in the prediction of developmental outcome for very low birth weight preterm infants. Child Development, 68(2), 173-86.

Doussard-Roosevelt, J., Porges, S. W., & McClenny, B. D. (1996). Behavioral Sleep States in Very-Low-Birth-Weight Preterm Neonates - Relation to Neonatal Health and Vagal Maturation. Journal of Pediatric Psychology, 21(6), 785-802.

Eisengart, S. P., Singer, L. T., Fulton, S., & Baley, J. E. (2003). Coping and Psychological Distress in Mothers of Very Low Birth Weight Young Children. Parenting: Science and Practice, 3(1), 49. doi: 10.1207/S15327922PAR0301_03.

Farel, A. M., & Hooper, S. R. (1998). Relationship between the Maternal Social Support Index and the Parenting Stress Index in mothers of very-low-birthweight children now age 7. Psychological Reports, 83(1), 173-4.

Farel, A. M., Hooper, S. R., Teplin, S. W., Henry, M. M., & Kraybill, E. N. (1998). Very-low-birthweight infants at seven years: an assessment of the health and neurodevelopmental risk conveyed by chronic lung disease. Journal of Learning Disabilities, 31(2), 118-26.

Halpern, L. F., Brand, K. L., & Malone, A. F. (2001). Parenting stress in mothers of very-low-birth-weight (VLBW) and full-term infants: a function of infant behavioral characteristics and child-rearing attitudes. Journal of Pediatric Psychology, 26(2), 93-104.

Harrison, M. J., & Magill-Evans, J. (1996). Mother and father interactions over the first year with term and preterm infants. Research in Nursing & Health, 19(6), 451-9.

Jackson, K., Ternestedt, B., Magnuson, A., & Schollin, J. (2007). Parental stress and toddler behaviour at age 18 months after pre-term birth. Acta Paediatrica, 96(2), 227-232. doi: 10.1111/j.1651-2227.2007.00015.x.

Kazdin, A. E. (1994). Family adversity, socioeconomic disadvantage, and parental stress: Contextual variables related to premature termination from child behavior therapy. Psicologia Conductual, 2(1), 5-21.

Kazdin, A. E., & Wassell, G. (1998). Treatment Completion and Therapeutic Change Among Children Referred for Outpatient Therapy. Professional Psychology-Research and Practice, 29(4), 332-340.

Kazdin, A. E., Holland, L., & Crowley, M. (1997). Family Experience of Barriers to Treatment and Premature Termination from Child Therapy. Journal of Consulting and Clinical Psychology, 65(3), 453-463.

Kazdin, A. E., Mazurick, J. L., & Bass, D. (1993). Risk for Attrition in Treatment of Antisocial Children and Families. Journal of Clinical Child Psychology, 22(1), 2-16.

Kazdin, A. E., Mazurick, J. L., & Siegel, T. C. (1994). Treatment Outcome Among Children with Externalizing Disorder Who Terminate Prematurely Versus Those Who Complete Psychotherapy. Journal of the American Academy of Child and Adolescent Psychiatry, 33(4), 549-557.

Magill-Evans, J., & Harrison, M. J. (1999). Parent-child interactions and development of toddlers born preterm. Western Journal of Nursing Research, 21(3), 292-307.

Magura, S., Laudet, A., Kang, S.-Y., & Whitney, S. A. (1999). Effectiveness of comprehensive services for crack-dependent mothers with newborns and young children. Journal of Psychoactive Drugs, 31(4), 321-338.

Meijssen, D., Wolf, M., Koldewijn, K., van Wassenaer, A., Kok, J., & van Baar, A. (2010). Parenting stress in mothers after very preterm birth and the effect of the Infant Behavioural Assessment and Intervention Program. Child: care, health and development, 37, 195-202.

Miceli, P. J., Goeke-Morey, M. C., Whitman, T. L., Kolberg, K. S., Miller-Loncar, C., & White, R. D. (2000). Brief report: birth status, medical complications, and social environment: individual differences in development of preterm, very low birth weight infants. Journal of Pediatric Psychology, 25(5), 353-8.

Miles, R., Cowan, F., Glover, V., Stevenson, J., & Modi, N. (2006). A controlled trial of skin-to-skin contact in extremely preterm infants. Early Human Development, 82(7), 447-455. doi: 10.1016/j.earlhumdev.2005.11.008.

Olafsen, K. S., Rønning, J. A., Bredrup Dahl, L., Ulvund, S. E., Handegård, B. H., & Kaaresen, P. I.(2007). Infant responsiveness and maternal confidence in the neonatal period. Scandinavian Journal of Psychology, 48(6), 499-509.

Ong, L. C., Chandran, V., & Boo, N. Y. (2001). Comparison of parenting stress between Malaysian mothers of four-year-old very low birthweight and normal birthweight children. Acta Paediatrica, 90(12), 1464-1469.

Onufrak, B., Saylor, C. F., Taylor, M. J., Eyberg, S. M., & Boyce, G. C. (1995). Determinants of responsiveness in mothers of children with intraventricular hemorrhage. Journal of Pediatric Psychology, 20(5), 587-99.

Robson, A. L. (1997). Low-Birth-Weight and Parenting Stress During Early-Childhood. Journal of Pediatric Psychology, 22(3), 297-311.

Saylor, C. F., Casto, G., & Huntington, L. (1996). Predictors of Developmental Outcomes for Medically Fragile Early Intervention Participants. Journal of Pediatric Psychology, 21(6), 869-887.

Taylor, H. G., Klein, N., Minich, N. M., & Hack, M. (2001). Long-term family outcomes for children with very low birth weights. Archives of Pediatrics & Adolescent Medicine, 155(2), 155-161.

Taylor, H. G., Klein, N., Schatschneider, C., & Hack, M. (1998). Predictors of Early School-Age Outcomes in Very-Low-Birth-Weight Children. Journal of Developmental and Behavioral Pediatrics, 19(4), 235-243.

van der Pal, S. M., Maguire, C. M., le Cessie, S., van Zwieten, P., Veen, S., Wit, J., & Walther, F. (2008). Very pre-term infants' behaviour at 1 and 2 years of age and

parental stress following basic developmental care. British Journal of Developmental Psychology, 26(1), 103-115.

Veddovi, M., Gibson, F., Kenny, D., Bowen, J., & Starte, D. (2004). Preterm behavior, maternal adjustment, and competencies in the newborn period: What influence do they have at 12 months postnatal age? Infant Mental Health Journal, 25(6), 580-599. doi: 10.1002/imhj.20026.

Youngblut, J. M., Brooten, D., Singer, L. T., Standing, T., Lee, H., & Rodgers, W. L. (2001). Effects of maternal employment and prematurity on child outcomes in single parent families. Nursing Research, 50(6), 346-355.

Youngblut, J. M., Singer, L. T., Madigan, E. A., Swegart, L. A., & Rodgers, W. L. (1998). Maternal employment and parent-child relationships in single-parent families of low-birth-weight preschoolers. Nursing Research, 47(2), 114-21.

Reproduction/Assistive Reproduction

Allen, K. D., Maguire, K. B., Williams, G. E., & Sanger, W. G. (1996). The effects of infertility on parent-child relationships and adjustment. Children's Health Care, 25(2), 93-105.

Barnes, J., Sutcliffe, A. G., Kristoffersen, I., Loft, A., Wnnerholm, U., Tarlatzis, B. C., … Bonduelle, M. (2004). The influence of assisted reproduction on family functioning and children's socio-emotional development: results from a European study. Human Reproduction, 19(6), 1480-1487.

Colpin, H., & Soenen, S. (2002). Parenting and psychosocial development of IVF children: a follow-up study. Human Reproduction, 17(4), 1116-1123.

Flykt, M., Lindblom, J., Punamaki, R., Poikkeus, P., Repokari, L., Unkila-Kallio, L., Vilska, S., Sinkkonen, J., Tiitinen, A., Almqvist, F., & Tulppala, M. (2009). Prenatal expectations in transition to parenthood: Former infertility and family dynamic considerations. Journal of Family Psychology, 23, 779-789.

Golombok, S., Jadva, V., Lycett, E., Murray, C., & MacCallum, F. (2005). Families created by gamete donation: follow-up at age 2. Human Reproduction, 20(1), 286-293.

Golombok, S., Murray, C., Jadva, V., Lycett, E., MacCallum, F., & Rust, J. (2006). Non-genetic and non-gestational parenthood: consequences for parent-child relationships and the psychological well-being of mothers, fathers and children at age 3. Human Reproduction, 21(7), 1918-1924. doi: 10.1093/humrep/del039

Golombok, S., Murray, C., Jadva, V., MacCallum, F., & Lycett, E. (2004). Families created through surrogacy arrangements: parent-child relationships in the 1st year of life. Developmental Psychology, 40(3), 400-411. doi: 10.1037/0012-1649.40.3.400.

Golombok, S., Olivennes, F., Ramogida, C., Rust, J., & Freeman, T. (2007). Parenting and the psychological development of a representative sample of triplets conceived by assisted reproduction. Human Reproduction (Oxford, England), 22(11), 2896-2902. doi: 10.1093/humrep/dem260.

Knoester, M., Helmerhorst, F. M., van der Westerlaken, L. A., Walther, F. J., & Veen, S. (2007). Matched follow-up study of 5 8-year old ICSI singletons: child behaviour, parenting stress and child (health-related) quality of life. Human Reproduction, 22(12), 3098-3107.

Murray, C., & Golombok, S. (2005). Solo mothers and their donor insemination infants: follow-up at age 2 years. Human Reproduction (Oxford, England), 20(6), 1655-1660. doi: 10.1093/humrep/deh823.

Nekkebroeck, J., Bonduelle, M., & Ponjaert-Kristoffersen, I. (2008). Maternal disclosure attitudes and practices of ICSI/IVF conception vis-à-vis a 5-year-old child. Journal of Reproductive & Infant Psychology, 26(1), 44-56. doi: 10.1080/02646830701813343.

Nekkebroeck, J., Bonduelle, M., Desmyttere, S., Van den Broeck, W., & Ponjaert-Kristoffersen, I. (2008). Socio-emotional and language development of 2-year-old children born after PGD/PGS, and parental well-being. Hum. Reprod., den179. doi: 10.1093/humrep/den179.

Ponjaert-Kristoffersen, I., Tjus, T., Nekkebroeck, J., Squires, J., Verté, D., Heimann, M., ... Wennerholm, U. B. (2004). Psychological follow-up study of 5-year-old ICSI children. Human Reproducation, 19(12), 2791-2797.

Sexual Development Disorders

Baker, A. J. L., Gries, L., Schneiderman, M., Parker, R., Archer, M., & Friedrich, B. (2008). Children with Problematic Sexualized Behaviors in the Child Welfare System. Child Welfare, 87(1), 5. doi: Article.

Duguid, A., Morrison, S., Robertson, A., Chalmers, J., Youngson, G., & Ahmed, S. F. (2007). The psychological impact of genital anomalies on the parents of affected children. Acta Paediatrica, 96(3), 348-352. doi: 10.1111/j.1651-2227.2006.00112.x.

Lux, A., Kropf, S., Kleinemeier, E., Jürgensen, M., & Thyen, U. (2009). Clinical evaluation study of the German network of disorders of sex development (DSD)/intersexuality: study design, description of the study population, and data quality. BMC Public Health, 9, 1-17. doi: 10.1186/1471-2458-9-110.

Sleep Disorders

Fagnano, M., van Wijngaarden, E., Connolly, H. V., Carno, M. A., Forbes-Jones, E., & Halterman, J. S. (2009). Sleep-Disordered Breathing and Behaviors of Inner-City Children With Asthma. Pediatrics, 124(1), 218-225. doi: 10.1542/peds.2008-2525.

Gelman, V. S., & King, N. J. (2001). Wellbeing of Mothers with Children Exhibiting Sleep Disturbance. Australian Journal of Psychology, 53(1), 18-22.

Sadeh, A., Lavie, P., & Scher, A. (1994). Sleep and temperament: Maternal perceptions of temperament of sleep-disturbed toddlers. Early Education & Development, 5(4), 311-322.

Thome, M., & Skuladottir, A. (2005). Changes in sleep problems, parents distress and impact of sleep problems from infancy to preschool age for referred and unreferred children. Scandinavian Journal of Caring Sciences, 19(2), 86-94. doi: 10.1111/j.1471-6712.2005.00322.x.

Thome, M., & Skuladottir, A. (2005). Evaluating a family-centred intervention for infant sleep problems. Journal of Advanced Nursing, 50(1), 5-11. doi: 10.1111/j.1365-2648.2004.03343.x.

Thunström, M. (2002). Severe sleep problems in infancy associated with subsequent development of attention-deficit/hyperactivity disorder at 5.5 years of age. Acta Paediatrica, 91(5), 584. doi: Article.

Wake, M., Morton-Allen, E., Poulakis, Z., Hiscock, H., Gallagher, S., & Oberklaid, F. (2006). Prevalence, stability, and outcomes of cry-fuss and sleep problems in the first 2 years of life: prospective community-based study. Pediatrics, 117(3), 836.

Tourette Syndrome

Corbett, B. A, Schupp, C. W., Levine, S., & Mendoza, S. (2009). Comparing cortisol, stress, and sensory sensitivity in children with autism. Autism Research, 2(1),39-49.

Corbett, B., Mendoza, S., Baym, C., Bunge, S., & Levine, S. (2008). Examining cortisol rhythmicity and responsivity to stress in children with Tourette syndrome. Psychoneuroendocrinology, 33(6), 810-820. doi: 10.1016/j.psyneuen.2008.03.014.

Lee, M., Chen, Y., Wang, H., & Chen, D. (2007). Parenting stress and related factors in parents of children with Tourette Syndrome. Journal of Nursing Research, 15(3), 165-174.

Wilkinson, B., Marshall, R., & Curtwright, B. (2008). Impact of Tourette's Disorder on Parent Reported Stress. Journal of Child & Family Studies, 17(4), 582-598. doi: 10.1007/s10826-007-9176-8.

Traumatic Brain Injury (TBI)

Bagner, D. M., & Eyberg, S. M. (2003). Father involvement in parent training: When does it matter?. Journal of Clinical Child & Adolescent Psychology, 32(4), 599-605.

Beardmore, S., Tate, R., & Liddle, B. (1999). Does Information and Feedback Improve Childrens Knowledge and Awareness of Deficits After Traumatic Brain Injury. Neuropsychological Rehabilitation, 9(1), 45-62.

Butcher, P. R., Wind, T., & Bouma, A. (2008). Parenting stress in mothers and fathers of a child with a hemiparesis: sources of stress, intervening factors and long-term expressions of stress. Child: Care, Health & Development, 34(4), 530-541. doi: 10.1111/j.1365-2214.2008.00842.x.

Farmer, J. E., Clippard, D. S., Luehrwiemann, Y., Wright, E., & Owings, S. (1996). Assessing Children with Traumatic Brain Injury During Rehabilitation - Promoting School and Community Reentry. Journal of Learning Disabilities, 29(5), 532-548.

Hawley, C. A., Ward, A. B., Magnay, A. R., & Long, J. (2003). Parental stress and burden following traumatic brain injury amongst children and adolescents. Brain Injury, 17(1), 1-23.

Youngblut, J. M., & Brooten, D. (2008). Mother's mental health, mother-child relationship, and family functioning 3 months after a preschooler's head injury. The Journal of Head Trauma Rehabilitation, 23(2), 92-102. doi: 10.1097/01.HTR.0000314528.85758.30.

Traumatic Injuries

Bijttebier, P., Vertommen, H., & Florentie, K. (2003). Risk-Taking Behavior as a Mediator of the Relationship between Childrens Temperament and Injury Liability. Psychology and Health, 18, 645-653. doi: 10.1080/0887044031000094831.

Gan, C., Campbell, K. A., Gemeinhardt, M., & McFadden, G. T. (2006). Predictors of family system functioning after brain injury. Brain Injury, 20(6), 587-600.

Youngblut, J. M., & Brooten, D. (2006). Pediatric head trauma: parent, parent-child, and family functioning 2 weeks after hospital discharge. Journal of Pediatric Psychology, 31(6), 608-618.

Learning Disabled

Fuller, G. B., & Rankin, R. E. (1994). Differences in levels of parental stress among mothers of learning disabled, emotionally impaired, and regular school children. Perceptual & Motor Skills, 78(2), 583-92.

Hodapp, R. M., Ricci, L. A., Ly, T. A., & Fidler, D. J. (2003). The effects of the child with Down syndrome on maternal stress. British Journal of Developmental Psychology, 21(1), 137-151.

Low Socio-Economic Status

Adamakos, H., Kathleen, R., G., U. D., & John, P. (1986). Maternal social support as a predictor of mother-child stress and stimulation. Child Abuse & Neglect, 10(4), 463-470.

Ammerman, R. T., & Patz, R. J. (1996). Determinants of Child-Abuse Potential - Contribution of Parent and Child Factors. Journal of Clinical Child Psychology, 25(3), 300-307.

Anderson, L. S. (2008). Predictors of Parenting Stress in a Diverse Sample of Parents of Early Adolescents in High-Risk Communities. Nursing Research, 57(5), 340-350. doi: 10.1097/01.NNR.0000313502.92227.87.

Bagley, C., & Mallick, K. (1997). Temperament, CNS Problems and Maternal Stressors - Interactive Predictors of Conduct Disorder in 9-Yr-Olds. Perceptual and Motor Skills, 84(2), 617-618.

Barnett, D. W., Hall, J. D., & Bramlett, R. K. (1990). Family factors in preschool assessment and intervention: A validity study of parenting stress and coping measures. Journal of School Psychology, 28(1), 13-20.

Black, M. M., & Nitz, K. (1996). Grandmother co-residence, parenting, and child development among low income, urban teen mothers. Journal of Adolescent Health, 18(3), 218-26.

Briggs-Gowan, M. J., Carter, A. S., Moye Skuban, E., & McCue Horwitz, S. (2001). Prevalence of Social-Emotional and Behavioral Problems in a Community Sample of 1- and 2-year-old Children. Journal of the American Academy of Child & Adolescent Psychiatry, 40(7), 811-819.

Brotman, L. M., Klein, R. G., Kamboukos, D., Brown, E. J., Coard, S. I., & Sosinsky, L. S. (2003). Preventive intervention for urban, low-income preschoolers at familial risk for conduct problems: A randomized pilot study. Journal of Clinical Child and Adolescent Psychology, 32(2), 246–257.

Browne, J. V., & Talmi, A. (2005). Family-based intervention to enhance infant-parent relationships in the neonatal intensive care unit. Journal of Pediatric Psychology, 30(8), 667-677.

Cain, D. S., & Combs-Orme, T. (2005). Family structure effects on parenting stress and practices in the African American family. Journal of Sociology and Social Welfare, 32(2), 19-40.

Casady, A., Diener, M., Isabella, R., & Wright, C. (2001). Attachment security among families in poverty: maternal, child, and contextual characteristics. Paper presented at

the 2001 Biennial Conference of the Society for Research in Child Development, Minneapolis, MN.

Coleman, P. K., & Karraker, K. H. (1998). Self-Efficacy and Parenting Quality - Findings and Future Applications. Developmental Review, 18(1), 47-85.

Cowan, P. A., Cowan, C. P., Pruett, M. K., Pruett, K., & Wong, J. J. (2009). Promoting Fathers' Engagement With Children: Preventive Interventions for Low-Income Families. Journal of Marriage and Family, 71(3), 663-679. doi: 10.1111/j.1741-3737.2009.00625.x.

Danseco, E. R., & Holden, E. W. (1998). Are There Different Types of Homeless Families - A Typology of Homeless Families Based on Cluster-Analysis. Family Relations, 47(2), 159-165.

DiLauro, M. D. (2004). Psychosocial Factors Associated with Types of Child Maltreatment. Child Welfare, 83(1), 69. doi: Article.

Fagnano, M., van Wijngaarden, E., Connolly, H. V., Carno, M. A., Forbes-Jones, E., & Halterman, J. S. (2009). Sleep-Disordered Breathing and Behaviors of Inner-City Children With Asthma. Pediatrics, 124(1), 218-225. doi: 10.1542/peds.2008-2525.

Feldman, M. A., & Waltonallen, N. (1997). Effects of Maternal Mental-Retardation and Poverty on Intellectual, Academic, and Behavioral Status of School-Age-Children. American Journal on Mental Retardation, 101(4), 352-364.

Gershoff, E. T., Raver, C. C., Aber, J. L., & Lennon, M. C. (2007). Income Is Not Enough: Incorporating Material Hardship Into Models of Income Associations With Parenting and Child Development. Child Development, 78(1), 70-95. doi: 10.1111/j.1467-8624.2007.00986.x.

Gorzka, P. A. (1999). Homeless parents: parenting education to prevent abusive behaviors. Journal of Child & Adolescent Psychiatric Nursing, 12(3), 101-9.

Gorzka, P. A. (1999). Homeless parents’ perceptions of parenting stress. Journal of Child & Adolescent Psychiatric Nursing, 12(1), 7-16.

Hall, J. D., & Barnett, D. W. (1991). Classification of risk status in preschool screening: A comparison of alternative measures. Journal of Psychoeducational Assessment, 9(2), 152-159.

Hooper, S. R., Burchinal, M. R., Roberts, J. E., Zeisel, S., & Neebe, E. C. (1998). Social and Family Risk-Factors for Infant Development at One-Year - An Application of the Cumulative Risk Model. Journal of Applied Developmental Psychology, 19(1), 85- 96.

Hutchings, J., Bywater, T., Daley, D., Gardner, F., Whitaker, C., Jones, K.,... Edwards, R. T. (2007). Parenting intervention in Sure Start services for children at risk of developing conduct disorder: pragmatic randomized controlled trial. British Medical Journal, doi:10.1136/bmj.39126.620799.55.

Ispa, J. M., Fine, M. A., Halgunseth, L. C., Harper, S., Robinson, J., Boyce, L., et al. (2004). Maternal Intrusiveness, Maternal Warmth, and Mother–Toddler Relationship Outcomes: Variations Across Low-Income Ethnic and Acculturation Groups. Child Development, 75(6), 1613-1631. doi: 10.1111/j.1467-8624.2004.00806.x.

Jackson, A. P., Bentler, P. M., & Franke, T. M. (2008). Low-Wage Maternal Employment and Parenting Style. Social Work, 53(3), 267. doi: Article.

Kemp, L., Harris, E., McMahon, C., Matthey, S., Vimpani, G., Anderson, T., et al. (2008). Miller Early Childhood Sustained Home-visiting (MECSH) trial: design, method and sample description. BMC Public Health, 8, 1-12. doi: 10.1186/1471-2458-8-424.

Larson, N. C. (2004). Parenting stress among adolescent mothers in the transition to adulthood. Child & Adolescent Social Work Journal, 21(5), 457-476.

Luthar, S. S., & Sexton, C. C. (2007). Maternal drug abuse versus maternal depression: Vulnerability and resilience among school-age and adolescent offspring. Development and Psychopathology, 19(01). doi: 10.1017/S0954579407070113.

Miranda, A., Grau, D., Rosel, J., & Meliá, A. (2009). Understanding discipline in families of children with attention-deficit/hyperactivity disorder: A structural equation model. The Spanish Journal of Psychology, 12(2), 496-505.

Mitchell, M. D., Hargrove, G. L., Collins, M. H., Thompson, M. P., Reddick, T. L., & Kaslow, N. J. (2006). Coping variables that mediate the relation between intimate partner violence and mental health outcomes among low-income, African American women. Journal of Clinical Psychology, 62(12), 1503-1520. doi: 10.1002/jclp.20305.

Murphy, D., Marelich, W., Armistead, L., Herbeck, D. and Payne, D. (2010). Anxiety/stress among mothers living with HIV: effects on parenting skills and child outcomes, AIDS Care, 22, 1449 – 1458.

Naik-Polan, A. T., & Budd, K.S. (2008). Stimulus generalization of parenting skills during parent-child interaction therapy. Journal of Early and Intensive Behavior Intervention, 5(3), 71-92.

New, M., Razzino, B., Lewin, A., Schlumpf, K., & Joseph, J. (2002). Mental Health Service Use in a Community Head Start Population. Arch Pediatr Adolesc Med, 156(7), 721-727. doi: 10.1001/archpedi.156.7.721.

Noel, M., Peterson, C., & Jesso, B. (2008). The relationship of parenting stress and child temperament to language development among economically disadvantaged preschoolers. Journal of Child Language, 35(4), 823-843. doi: 10.1017/S0305000908008805.

Pan, B. A., Rowe, M. L., Singer, J. D., & Snow, C. E. (2005). Maternal correlates of growth in toddler vocabulary production in low-income families. Child Development, 76(4), 763-782. doi: 10.1111/j.1467-8624.2005.00876.x.

Raver, C. C., Gershoff, E. T., & Aber, J. L. (2007). Testing Equivalence of Mediating Models of Income, Parenting, and School Readiness for White, Black, and Hispanic Children in a National Sample. Child Development, 78(1), 96-115. doi: 10.1111/j.1467-8624.2007.00987.x.

Razzino, B. E., New, M., Lewin, A., & Joseph, J. (2004). Need for and use of mental health services among parents of children in the head start program. Psychiatric Services (Washington, D.C.), 55(5), 583-586.

Reitman, D., Currier, R. O., & Stickle, T. R. (2002). A critical evaluation of the Parenting Stress Index-Short Form (PSI-SF) in a head start population. Journal of Clinical Child & Adolescent Psychology, 31(3), 384-392.

Silver, E. J., Henegham, A. M., Bauman, L. J., & Stein, R. (2006). The relationship of depressive symptoms to parenting competence and social support in inner-city mothers of young children. Maternal & Child Health Journal, 10(1), 105-112.

Slack, K. S., & Yoo, J. (2005). Food Hardship and Child Behavior Problems among Low-Income Children. Social Service Review, 79(3), 511-536. doi: 10.1086/430894.

Tandon, S. D., Parillo, K. M., Jenkins, C., & Duggan, A. K. (2005). Formative Evaluation of Home Visitors' Role in Addressing Poor Mental Health, Domestic Violence, and Substance Abuse Among Low-Income Pregnant and Parenting Women.. Maternal & Child Health Journal, 9(3), 273-283. doi: 10.1007/s10995-005-0012-8.

Taylor, J. A., Davis, R. L., & Kemper, K. J. (1997). A Randomized Controlled Trial of Group Versus Individual Well Child-Care for High-Risk Children - Maternal-Child Interaction and Developmental Outcomes. Pediatrics, 99(6), E91-E96.

Unger, D. G., Jones, C. W., Park, E., & Tressell, P. A. (2001). Promoting Involvement Between Low-Income Single Caregivers and Urban Early Intervention Programs. Topics in Early Childhood Special Education, 21(4), 197. doi: Article.

Waldron, A., Tobin, G., & McQuaid, P. (2001). Mental health status of homeless children and their families. Irish Journal of Psychological Medicine, 18(1), 11-15.

Mothers

Adam, E. K., & Gunnar, M. R. (2001). Relationship functioning and home and work demands predict individual differences in diurnal cortisol patterns in women. Psychoneuroendocrinology, 26(2), 189-208.

Amankwaa, L., & Pickler, R. (2007). Measuring Maternal Responsiveness. ABNF Journal, 18(1), 4-15. doi: Article.

Baker-Ericzén, M. J., Brookman-Frazee, L., & Stahmer, A. (2005). Stress levels and adaptability in parents of toddlers with and without autism spectrum disorders. Research and Practice for Persons with Severe Disabilities, 30(4), 194–204.

Barnet, B., Duggan, A. K., Devoe, M., & Burrell, L. (2002). The effect of volunteer home visitation for adolescent mothers on parenting and mental health outcomes: A randomized trial. Archives of Pediatrics & Adolescent Medicine, 156(12), 1216-1222.

Beck, A., Hastings, R. P., Daley, D., & Stevenson, J. (2004). Pro-social behaviour and behaviour problems independently predict maternal stress. Journal of Intellectual and Developmental Disability, 29(4), 339–349.

Birkeland, R., Thompson, J. K., & Phares, V. (2005). Adolescent motherhood and postpartum depression. Journal of Clinical Child and Adolescent Psychology, 34(2), 292–300.

Bos, H. M., van Balen, F., & van den Boom, D. C. (2004). Experience of parenthood, couple relationship, social support, and child-rearing goals in planned lesbian mother families. Journal of Child Psychology and Psychiatry, 45(4), 755–764.

Button, S., Pianta, R. C., & Marvin, R. S. (2001). Mothers' representations of relationships with their children: Relations with parenting behavior, mother characteristics, and child disability status. Social Development, 10(4), 455-472. doi: 10.1111/1467-9507.00175

Calam, R., Bolton, C., & Roberts, J. (2002). Maternal expressed emotion, attributions and depression and entry into therapy for children with behaviour problems. British Journal of Clinical Psychology, 41(2), 213216.

Calkins, S. D., Hungerford, A., & Dedmon, S. E. (2004). Mothers' interactions with temperamentally frustrated infants. Infant Mental Health Journal, 25(3), 219–239.

Casady, A., Diener, M., Isabella, R., & Wright, C. (2001). Attachment security among families in poverty: maternal, child, and contextual characteristics. Paper presented at the 2001 Biennial Conference of the Society for Research in Child Development, Minneapolis, MN.

Chazan-Cohen, R., Ayoub, C., Pan, B. A., Roggman, L., Raikes, H., Mckelvey, L., et al. (2007). It takes time: Impacts of Early Head Start that lead to reductions in maternal depression two years later. Infant Mental Health Journal, 28(2), 151-170. doi: 10.1002/imhj.20127.

Combs-Orme, T., Cain, D. S., & Wilson, E. E. (2004). Do maternal concerns at delivery predict parenting stress during infancy? Child Abuse & Neglect, 28(4), 377–392.

Cornish, A. M., McMahon, C. A., Ungerer, J. A., Barnett, B., Kowalenko, N., & Tennant, C. (2006). Maternal depression and the experience of parenting in the second postnatal year. Journal of Reproductive & Infant Psychology, 24(2), 121-132. doi: 10.1080/02646830600644021.

Damashek, A. L., Williams, N. A., Sher, K. J., Peterson, L., Lewis, T., & Schweinle, W. (2005). Risk for minor childhood injury: an investigation of maternal and child factors. Journal of Pediatric Psychology, 30(6), 469-480. doi: 10.1093/jpepsy/jsi072.

De Los Reyes, A., & Kazdin, A. E. (2006). Informant Discrepancies in Assessing Child Dysfunction Relate to Dysfunction Within Mother-Child Interactions. Journal of Child & Family Studies, 15(5), 643-661. doi: 10.1007/s10826-006-9031-3.

DeCaro, J. A., & Worthman, C. M. (2008). Return to school accompanied by changing associations between family ecology and cortisol. Developmental Psychobiology, 50(2), 183–195.

Eisengart, S. P., Singer, L. T., Fulton, S., & Baley, J. E. (2003). Coping and Psychological Distress in Mothers of Very Low Birth Weight Young Children. Parenting: Science and Practice, 3(1), 49. doi: 10.1207/S15327922PAR0301_03.

Eisengart, S. P., Singer, L. T., Kirchner, H. L., Min, M. O., Fulton, S., Short, E. J., et al. (2006). Factor structure of coping: two studies of mothers with high levels of life stress. Psychological Assessment, 18(3), 278-288. doi: 10.1037/1040-3590.18.3.278.

Feldman, M. A., Varghese, J., Ramsay, J., & Rajska, D. (2002). Relationships between social support, stress and mother-child interactions in mothers with intellectual disabilities. Journal of Applied Research in Intellectual Disabiliites, 15(4), 314-323.

Feldman, R., Granat, A., Pariente, C., Kanety, H., Kuint, J., & Gilboa-Schechtman, E. (2009). Maternal Depression and Anxiety Across the Postpartum Year and Infant Social Engagement, Fear Regulation, and Stress Reactivity. Journal of Amer Academy of Child & Adolescent Psychiatry, 48(9), 919 - 927.

Forbes, L. M., Evans, E. M., Moran, G., & Pederson, D. R. (2007). Change in Atypical Maternal Behavior Predicts Change in Attachment Disorganization From 12 to 24

Months in a High-Risk Sample. Child Development, 78(3), 955-971. doi: 10.1111/j.1467-8624.2007.01043.x.

Gartstein, M. A., & Sheeber, L. (2004). Child Behavior Problems and Maternal Symptoms of Depression: A Mediational Model. Journal of Child & Adolescent Psychiatric Nursing, 17(4), 141-150. doi: Article.

Glavin, K., Smith, L., Sørum, R., & Ellefsen, B. (2010). Redesigned community postpartum care to prevent and treat postpartum depression in women - a one-year follow-up study. Journal of Clinical Nursing, 19, 3051-3062.

Glenn, S. S., Cunningham, C. C., Poole, H. H., Reeves, D. D., & Weindling, M. M. (2009). Maternal parenting stress and its correlates in families with a young child with cerebral palsy. Child: Care, Health and Development, 35(1), 71-78.

Hall, S., & Marteau, T. M. (2003). Causal attributions and blame: associations with mothers' adjustment to the birth of a child with Down syndrome. Psychology, Health & Medicine, 8(4), 415. doi: 10.1080/1354850310001604559.

Halpern, L. F., Brand, K. L., & Malone, A. F. (2001). Parenting stress in mothers of very-low-birth-weight (VLBW) and full-term infants: a function of infant behavioral characteristics and child-rearing attitudes. Journal of Pediatric Psychology, 26(2), 93-104.

Hanada, H., Honda, S., Tokumaru, T., & Hiroki, O. (2006). Association Between Mothers' Concern About Child Rearing and Their Parenting Stress. Acta Med Nagasaki Ensia, 51(4), 115-120.

Hodapp, R. M., Ricci, L. A., Ly, T. A., & Fidler, D. J. (2003). The effects of the child with Down syndrome on maternal stress. British Journal of Developmental Psychology, 21(1), 137-151.

Hoffman, C. D., Sweeney, D. P., Hodge, D., Lopez-Wagner, M. C., & Looney, L. (2009). Parenting Stress and Closeness: Mothers of Typically Developing Children and Mothers of Children With Autism. Focus on Autism and Other Developmental Disabilities, 24(3), 178-187. doi: 10.1177/1088357609338715

Holub, C. K., Kershaw, T. S., Ethier, K. A., Lewis, J. B., Milan, S., & Ickovics, J. R. (2007). Prenatal and Parenting Stress on Adolescent Maternal Adjustment: Identifying a High-Risk Subgroup.. Maternal & Child Health Journal, 11(2), 153-159. doi: 10.1007/s10995-006-0159-y.

Horwitz, S. M., Briggs-Gowan, M. J., Storfer-Isser, A., & Carter, A. S. (2009). Persistence of Maternal Depressive Symptoms throughout the Early Years of Childhood. Journal of Women's Health, 18(5), 637-645. doi: 10.1089/jwh.2008.1229.

Huth-Bocks, A. C., Levendosky, A. A., Bogat, G. A., & Von Eye, A. (2004). The Impact of Maternal Characteristics and Contextual Variables on Infant–Mother Attachment. Child Development, 75(2), 480-496. doi: 10.1111/j.1467-8624.2004.00688.x.

Ispa, J. M., Fine, M. A., Halgunseth, L. C., Harper, S., Robinson, J., Boyce, L., et al. (2004). Maternal Intrusiveness, Maternal Warmth, and Mother–Toddler Relationship Outcomes: Variations Across Low-Income Ethnic and Acculturation Groups. Child Development, 75(6), 1613-1631. doi: 10.1111/j.1467-8624.2004.00806.x.

Kemp, L., Harris, E., McMahon, C., Matthey, S., Vimpani, G., Anderson, T., et al. (2008). Miller Early Childhood Sustained Home-visiting (MECSH) trial: design, method and sample description. BMC Public Health, 8, 1-12. doi: 10.1186/1471-2458-8-424.

Knight, D. K., & Wallace, G. (2003). Where are the children? An examination of children's living arrangements when mothers enter residential drug treatment. Journal of Drug Issues, 33(2), 305. doi: Article.

Leigh, B., & Milgrom, J. (2008). Risk factors for antenatal depression, postnatal depression and parenting stress. BMC psychiatry, 8(1), 24.

Levendosky, A. A., & Graham-Bermann, S. A. (2001). Parenting in battered women: the effects of domestic violence on women and their children. Journal of Family Violence, 16(2), 171-192.

Lisul-Mihić, I., & Kapor-Stanulović, N. (2002). Cultural influence on aims of the inclusion of mothers in pre-school children's play. Psihologija, 35(1-2), 49-64. doi: 10.2298/PSI0201049L.

Loper, A. B. (2006). How Do Mothers in Prison Differ From Non-Mothers? Journal of Child & Family Studies, 15(1), 83-95. doi: 10.1007/s10826-005-9005-x.

Luthar, S. S., & Sexton, C. C. (2007). Maternal drug abuse versus maternal depression: Vulnerability and resilience among school-age and adolescent offspring. Development and Psychopathology, 19(01). doi: 10.1017/S0954579407070113.

Mackintosh, V. H., Myers, B. J., & Kennon, S. S. (2006). Children of Incarcerated Mothers and Their Caregivers: Factors Affecting the Quality of Their Relationship. Journal of Child & Family Studies, 15(5), 579-594. doi: 10.1007/s10826-006-9030-4.

McAuley, C., McCurry, N., Knapp, M., Beecham, J., & Sleed, M. (2006). Young families under stress: assessing maternal and child well-being using a mixed-methods

approach. Child & Family Social Work, 11(1), 43-54. doi: 10.1111/j.1365-2206.2006.00390.x.

McCue Horwitz, S., Briggs-Gowan, M. J., Storfer-Isser, A., & Carter, A. S. (2007). Prevalence, Correlates, and Persistence of Maternal Depression. Journal of Women's Health, 16(5), 678-691. doi: 10.1089/jwh.2006.0185.

McPherson, A., Lewis, K., Lynn, A., Haskett, M., & Behrend, T. (2009). Predictors of Parenting Stress for Abusive and Nonabusive Mothers. Journal of Child and Family Studies, 18(1), 61-69. doi: 10.1007/s10826-008-9207-0.

Meijssen, D., Wolf, M., Koldewijn, K., van Wassenaer, A., Kok, J., & van Baar, A. (2010). Parenting stress in mothers after very preterm birth and the effect of the Infant Behavioural Assessment and Intervention Program. Child: care, health and development, 37, 195-202.

Miller-Loncar, C., Bigsby, R., High, P., Wallach, M., & Lester, B. (2004). Infant colic and feeding difficulties. Archives of Disease in Childhood, 89(10), 908.

Miranda, A., Grau, D., Rosel, J., & Meliá, A. (2009). Understanding discipline in families of children with attention-deficit/hyperactivity disorder: A structural equation model. The Spanish Journal of Psychology, 12(2), 496-505.

Misri, S., Reebye, P., Milis, L., & Shah, S. (2006). The impact of treatment intervention on parenting stress in postpartum depressed mothers: a prospective study. American Journal of Orthopsychiatry, 76(1), 115–119.

Most, D. E., Fidler, D. J., Laforce-Booth, C., & Kelly, J. (2006). Stress trajectories in mothers of young children with Down syndrome. Journal of Intellectual Disability Research, 50(7), 501-514. doi: 10.1111/j.1365-2788.2006.00796.x.

Mowbray, C. T., Bybee, D., Hollingsworth, L., Goodkind, S., & Oyserman, D. (2005). Living Arrangements and Social Support: Effects on the Well-Being of Mothers with Mental Illness. Social Work Research, 29(1), 41. doi: Article.

Mowbray, C., Oyserman, D., Bybee, D., & MacFarlane, P. (2002). Parenting of mothers with a serious mental illness: Differential effects of diagnosis, clinical history, and other mental health variables. Social Work Research, 26(4), 225. doi: Article.

Mullick, M., Miller, L. J., & Jacobsen, T. (2001). Insight into mental illness and child maltreatment risk among mothers with major psychiatric disorders. Psychiatric Services, 52(4), 488-492.

Murphy, D., Marelich, W., Armistead, L., Herbeck, D. and Payne, D. (2010). Anxiety/stress among mothers living with HIV: effects on parenting skills and child outcomes, AIDS Care, 22, 1449 – 1458.

Nekkebroeck, J., Bonduelle, M., & Ponjaert-Kristoffersen, I. (2008). Maternal disclosure attitudes and practices of ICSI/IVF conception vis-à-vis a 5-year-old child. Journal of Reproductive & Infant Psychology, 26(1), 44-56. doi: 10.1080/02646830701813343.

Newman, L. K., Stevenson, C. S., Bergman, L. R., & Boyce, P. (2007). Borderline personality disorder, mother-infant interaction and parenting perceptions: preliminary findings. Australian & New Zealand Journal of Psychiatry, 41(7), 598-605. doi: 10.1080/00048670701392833.

O'Neil, M. E., Palisano, R. J., & Westcott, S. L. (2001). Relationship of therapists' attitudes, children's motor ability, and parenting stress to mothers' perceptions of therapists' behaviors during early intervention. Phsyical Therapy, 81(8), 1412-1424.

Ong, L. C., Chandran, V., & Boo, N. Y. (2001). Comparison of parenting stress between Malaysian mothers of four-year-old very low birthweight and normal birthweight children. Acta Paediatrica, 90(12), 1464-1469.

Oyserman, D., Bybee, D., Mowbray, C. T., & MacFarlane, P. (2002). Positive Parenting among African American Mothers with a Serious Illness. Journal of Marriage and Family, 64(1), 65-77.

Paulussen-Hoogeboom, M. C., Stams, G. J. J. M., Hermanns, J. M. A., Peetsma, T. T. D., & Van Den Wittenboer, G. L. H. (2008). Parenting style as a mediator between children's negative emotionality and problematic behavior in early childhood. Journal of Genetic Psychology, 169(3), 209.

Qin, X., Tang, C., Zhu, S., Liang, Y., & Zou, X. (2009). Parenting stress and related factors in mothers of children with autism. Chinese Mental Health Journal, 23(9), 629-633.

Reissland, N., Hopkins, B., Helms, P., & Williams, B. (2009). Maternal stress and depression and the lateralisation of infant cradling. Journal of Child Psychology and Psychiatry, and Allied Disciplines, 50(3), 263-269. doi: 10.1111/j.1469-7610.2007.01791.x.

Secco, M. L., & Moffatt, M. (2003). Situational, maternal, and infant influences on parenting stress among adolescent mothers. Issues in Comprehensive Pediatric Nursing, 26(2), 103-122.

Sperry, L. A., & Symons, F. J. (2003). Maternal judgments of intentionality in young children with autism: the effects of diagnostic information and stereotyped behavior. Journal of Autism and Developmental Disorders, 33(3), 281–287.

Suchman, N. E., & Luthar, S. S. (2001). The mediating role of parenting stress in methadone-maintained mothers' parenting. Parenting, Science and Practice, 1(4), 285-315.

Sullivan-Bolyai, S., Deatrick, J., Gruppuso, P., Tamborlane, W., & Grey, M. (2002). Mothers' Experiences Raising Young Children With Type 1 Diabetes. Journal for Specialists in Pediatric Nursing, 7(3), 93. doi: Article.

Taylor, C. A., Guterman, N. B., Lee, S. J., & Rathouz, P. J. (2009). Intimate Partner Violence, Maternal Stress, Nativity, and Risk for Maternal Maltreatment of Young Children. American Journal of Public Health, 99(1), 175. doi: 10.2105/AJPH.2007.126722.

Timmer, S. G., Borrego, J., & Urquiza, A. J. (2002). Antecedents of Coercive Interactions in Physically Abusive Mother-Child Dyads. J Interpers Violence, 17(8), 836-853. doi: 10.1177/0886260502017008003.

Travis, W. J., & Combs-Orme, T. (2007). Resilient Parenting: Overcoming Poor Parental Bonding. Social Work Research, 31(3), 135. doi: Article.

Weinfield, N. S., Ogawa, J. R., & Egeland, B. (2002). Predictability of observed mother-child interaction from preschool to middle childhood in a high-risk sample. Child development, 528–543.

Wheeler, A., Hatton, D., Reichardt, A., & Bailey, D. (2007). Correlates of maternal behaviours in mothers of children with fragile X syndrome. Journal of Intellectual Disability Research, 51(6), 447-462. doi: 10.1111/j.1365-2788.2006.00896.x.

Wilkinson, B., Marshall, R., & Curtwright, B. (2008). Impact of Tourette's Disorder on Parent Reported Stress. Journal of Child & Family Studies, 17(4), 582-598. doi: 10.1007/s10826-007-9176-8.

Williford, A. P., Calkins, S. D., & Keane, S. P. (2007). Predicting change in parenting stress across early childhood: Child and maternal factors. Journal of Abnormal Child Psychology, 35(2), 251–263.

Wulffaert, J., Scholte, E., & van Berckelaer-Onnes, I. (2010). Maternal parenting stress in families with a child with Angelman syndrome or Prader–Willi syndrome, Journal of Intellectual & Developmental Disability, 35, 165-174.

Youngblut, J. M., Brooten, D., Singer, L. T., Standing, T., Lee, H., & Rodgers, W. L. (2001). Effects of maternal employment and prematurity on child outcomes in single parent families. Nursing Research, 50(6), 346-355.

Mother's Health/Addiction

Adam, E. K., & Gunnar, M. R. (2001). Relationship functioning and home and work demands predict individual differences in diurnal cortisol patterns in women. Psychoneuroendocrinology, 26(2), 189-208.

August, G. J., Realmuto, G. M., Hektner, J. M., & Bloomquist, M. L. (2001). An integrated components preventive intervention for aggressive elementary school children: the early risers program. Journal of Consulting and Clinical Psychology, 69(4), 614-626.

Aunos, M., Feldman, M., & Goupil, G. (2008). Mothering with intellectual disabilities: relationship between social support, health, and well-being, parenting and child behaviour outcomes. Journal of Applied Research in Intellectual Disabilities, 21(4), 320-330.

Boukydis, C. Z., & Lester, B. M. (2008). Mother-infant consultation during drug treatment: Research and innovative clinical practice. Harm Reduction Journal, 5(6),

Brown, J. V., Bakeman, R., Coles, C. D., Platzman, K. A., & Lynch, M. E. (2004). Prenatal cocaine exposure: A comparison of 2-year-old children in parental and nonparental care. Child Development, 75(4), 1282–1295.

Butz, A. M., Pulsifer, M., Marano, N., Belcher, H., Lears, M. K., & Royall, R. (2001). Effectiveness of a home intervention for perceived child behavioral problems and parenting stress in children with in utero drug exposure. Archives of Pediatrics & Adolescent Medicine, 155(9), 1029-1037.

Dawe, S., Harnett, P. H., Rendalls, V., & Staiger, P. (2003). Improving family functioning and child outcome in methadone maintained families: the Parents Under Pressure programme. Drug and Alcohol Review, 22(3), 299–307.

Murphy, D., Marelich, W., Armistead, L., Herbeck, D. and Payne, D. (2010). Anxiety/stress among mothers living with HIV: effects on parenting skills and child outcomes, AIDS Care, 22, 1449 – 1458.

Parent-Child Interaction/Observed Behavior

Aunos, M., Feldman, M., & Goupil, G. (2008). Mothering with intellectual disabilities: relationship between social support, health, and well-being, parenting and child behaviour outcomes. Journal of Applied Research in Intellectual Disabilities, 21(4), 320-330.

Bagner, D. M., & Eyberg, S. M. (2007). Parent—Child Interaction Therapy for Disruptive Behavior in Children with Mental Retardation: A Randomized Controlled Trial. Journal of Clinical Child & Adolescent Psychology, 36(3), 418-429. doi: 10.1080/15374410701448448.

Bigras, M., & Lafreniere, P. J. (1994). Influence of Psychosocial Risk, Marital Conflicts and Parental Stress on the Quality of Mother-Son and Mother-Daughter Interactions. Canadian Journal of Behavioural Science, 26(2), 280-297.

Black, M. M., Hutcheson, J. J., Dubowitz, H., Starr, R. H., & Berensonhoward, J. (1996). The Roots of Competence - Mother-Child Interaction Among Low-Income, Urban, African-American Families. Journal of Applied Developmental Psychology, 17(3), 367-391.

Burbach, A. D., Fox, R. A., & Nicholson, B. C. (2004). Challenging Behaviors in Young Children: The Father's Role. Journal of Genetic Psychology, 165(2), 169. doi: Article.

Button, S., Pianta, R. C., & Marvin, R. S. (2001). Partner support and maternal stress in families raising young children with cerebral palsy. Journal of Developmental & Physical Disabilities, 13(1), 61-81.

Cain, D. S., & Combs-Orme, T. (2005). Family structure effects on parenting stress and practices in the African American family. Journal of Sociology and Social Welfare, 32(2), 19-40.

Calam, R., Bolton, C., & Roberts, J. (2002). Maternal expressed emotion, attributions and depression and entry into therapy for children with behaviour problems. British Journal of Clinical Psychology, 41(2), 213216.

Calkins, S. D., Hungerford, A., & Dedmon, S. E. (2004). Mothers' interactions with temperamentally frustrated infants. Infant Mental Health Journal, 25(3), 219–239.

Campbell, S. B., Pierce, E. W., Moore, G., Marakovitz, S., & Newby, K. (1996). Boys Externalizing Problems at Elementary-School Age - Pathways from Early Behavior Problems, Maternal Control, and Family Stress. Development and Psychopathology, 8(4), 701 -719.

Casady, A., Diener, M., Isabella, R., & Wright, C. (2001). Attachment security among families in poverty: maternal, child, and contextual characteristics. Paper presented at the 2001 Biennial Conference of the Society for Research in Child Development, Minneapolis, MN.

Caulfield, M. B., Fischel, J. E., DeBaryshe, B. D., & Whitehurst, G. J. (1989). Behavioral correlates of developmental expressive language disorder. Journal of Abnormal Child Psychology, 17(2), 187-201.

Clark, R., Hyde, J. S., Essex, M. J., & Klein, M. H. (1997). Length of Maternity Leave and Quality of Mother-Infant Interactions. Child Development, 68(2), 364-383.

Cowan, P. A., Cowan, C. P., Pruett, M. K., Pruett, K., & Wong, J. J. (2009). Promoting Fathers' Engagement With Children: Preventive Interventions for Low-Income Families. Journal of Marriage and Family, 71(3), 663-679. doi: 10.1111/j.1741-3737.2009.00625.x.

Crandell, L. E., Fitzgerald, H. E., & Whipple, E. E. (1997). Dyadic Synchrony in Parent-Child Interactions - A Link with Maternal Representations of Attachment Relationships. Infant Mental Health Journal, 18(3), 247-264.

Dawe, S., Harnett, P. H., Rendalls, V., & Staiger, P. (2003). Improving family functioning and child outcome in methadone maintained families: the Parents Under Pressure programme. Drug and Alcohol Review, 22(3), 299–307.

De Los Reyes, A., & Kazdin, A. E. (2006). Informant Discrepancies in Assessing Child Dysfunction Relate to Dysfunction Within Mother-Child Interactions. Journal of Child & Family Studies, 15(5), 643-661. doi: 10.1007/s10826-006-9031-3.

Deković, M., Janssens, J. M., & van As, N. M. (2003). Family Predictors of Antisocial Behavior in Adolescence. Family Process, 42(2), 223. doi: Article.

Dumas, J. E., & Wekerle, C. (1995). Maternal Reports of Child-Behavior Problems and Personal Distress as Predictors of Dysfunctional Parenting. Development and Psychopathology, 7(3), 465-479.

Dumas, J. E., Nissley, J., Nordstrom, A., Smith, E. P., Prinz, R. J., & Levine, D. W. (2005). Home Chaos: Sociodemographic, Parenting, Interactional, and Child Correlates. Journal of Clinical Child & Adolescent Psychology, 34(1), 93-104. doi: 10.1207/s15374424jccp3401_9.

Eisenstadt, T. H., Eyberg, S., Mcneil, C. B., Newcomb, K., & Funderburk, B. (1993). Parent-Child Interaction Therapy with Behavior Problem Children - Relative

Effectiveness of 2 Stages and Overall Treatment Outcome. Journal of Clinical Child Psychology , 22(1), 42-51.

Emery, J., Paquette, D., & Bigras, M. (2008). Factors predicting attachment patterns in infants of adolescent mothers. Journal of Family Studies, 14(1), 65-90. doi: Article.

Esdaile, S. A. (1996). A play-focused intervention involving mothers of preschoolers. American Journal of Occupational Therapy, 50(2), 113-23.

Esdaile, S. A., & Greenwood, K. M. (1995). A survey of mothers' relationships with their preschoolers. Occupational Therapy International, 2(3), 204-19.

Esdaile, S. A., & Greenwood, K. M. (2003). A comparison of mother's and father's experience of parenting stress and attributions for parent-child interaction outcomes. Occupational Therapy International, 10(2), 115. doi: Article.

Ethier, L. S., & Lafreniere, P. J. (1993). Single-Parent Maternal Stress with Respect to Preschooler Aggression. International Journal of Psychology, 28(3), 273-289.

Ethier, L. S., & Lafreniere, P. J. (1993). The relationship between maternal stress and preschool children's aggressiveness in single-parent families. International Journal of Psychology, 28(3), 273-289.

Eyberg, S. M. B. S. R. A. J. (1995). Parent-child interaction therapy: A psychosocial model for the treatment of young children with conduct problem behavior and their families. Psychopharmacology Bulletin, 31(1), 83-91.

Fagan, J., Bernd, E., & Whiteman, V. (2007). Adolescent Fathers' Parenting Stress, Social Support, and Involvement with Infants. Journal of Research on Adolescence (Blackwell Publishing Limited), 17(1), 1-22. doi: 10.1111/j.1532-7795.2007.00510.x.

Feldman, M. A., Varghese, J., Ramsay, J., & Rajska, D. (2002). Relationships between social support, stress and mother-child interactions in mothers with intellectual disabilities. Journal of Applied Research in Intellectual Disabiliites, 15(4), 314-323.

Feldman, R., Eidelman, A. I., & Rotenberg, N. (2004). Parenting Stress, Infant Emotion Regulation, Maternal Sensitivity, and the Cognitive Development of Triplets: A Model for Parent and Child Influences in a Unique Ecology. Child Development, 75(6), 1774-1791. doi: 10.1111/j.1467-8624.2004.00816.x.

Feldman, R., Granat, A., Pariente, C., Kanety, H., Kuint, J., & Gilboa-Schechtman, E. (2009). Maternal Depression and Anxiety Across the Postpartum Year and Infant Social Engagement, Fear Regulation, and Stress Reactivity. Journal of Amer Academy of Child & Adolescent Psychiatry, 48(9), 919 - 927.

Frankel, K. K., & Harmon, R. J. (1996). Depressed Mothers - They Dont Always Look as Bad as They Feel. Journal of the American Academy of Child and Adolescent Psychiatry, 35(3), 289-298.

Gartstein, M. A., & Marmion, J. (2008). Fear and positive affectivity in infancy: Convergence/discrepancy between parent-report and laboratory-based indicators. Infant Behavior & Development, 31(2), 227-238.

Girolametto, L., & Tannock, R. (1994). Correlates of Directiveness in the Interactions of Fathers and Mothers of Children with Developmental Delays. Journal of Speech and Hearing Research, 37(5), 1178-1191.

Golombok, S., Murray, C., Jadva, V., Lycett, E., MacCallum, F., & Rust, J. (2006). Non-genetic and non-gestational parenthood: consequences for parent-child relationships and the psychological well-being of mothers, fathers and children at age 3. Human Reproduction, 21(7), 1918-1924. doi: 10.1093/humrep/del039

Golombok, S., Murray, C., Jadva, V., MacCallum, F., & Lycett, E. (2004). Families created through surrogacy arrangements: parent-child relationships in the 1st year of life. Developmental Psychology, 40(3), 400-411. doi: 10.1037/0012-1649.40.3.400.

Golombok, S., Tasker, F., & Murray, C. (1997). Children Raised in Fatherless Families from Infancy - Family Relationships and the Socioemotional Development of Children of Lesbian and Single Heterosexual Mothers. Journal of Child Psychology and Psychiatry and Allied Disciplines, 38(7), 783-791.

Grant, D. (1996). Generalizability of Findings of Exploratory Practice-Based Research on Polydrug-Addicted Mothers. Research on Social Work Practice, 6(3), 292-307.

Guralnick, M. J., Neville, B., Hammond, M. A., & Connor, R. T. (2007). Linkages Between Delayed Children's Social Interactions With Mothers and Peers. Child Development, 78(2), 459-473. doi: 10.1111/j.1467-8624.2007.01009.x.

Hadadian, A., & Merbler, J. (1996). Mother’s stress: Implications for attachment relationships. Early Child Development & Care, 125, 59-66.

Hanson, M. J., & Hanline, M. F. (1990). Parenting a child with a disability: A longitudinal study of parental stress and adaptation. Journal of Early Intervention, 14(3), 234-248.

Hutcheson, J. J., & Black, M. M. (1996). Psychometric properties of the Parenting Stress Index in a sample of low-income African-American mothers of infants and toddlers. Early Education & Development, 7(4), 381-400.

Ispa, J. M., Fine, M. A., Halgunseth, L. C., Harper, S., Robinson, J., Boyce, L., et al. (2004). Maternal Intrusiveness, Maternal Warmth, and Mother–Toddler Relationship

Outcomes: Variations Across Low-Income Ethnic and Acculturation Groups. Child Development, 75(6), 1613-1631. doi: 10.1111/j.1467-8624.2004.00806.x.

Jackson, A. P., Gyamfi, P., Brooksgunn, J., & Blake, M. (1998). Employment Status, Psychological Well-Being, Social Support, and Physical Discipline Practices of Single Black Mothers. Journal of Marriage and the Family, 60(4), 894-902.

Kazui, M., Muto, T., & Sonoda, N. (1996). The roles of marital quality and parenting stress in mother-preschooler relationships. (Japanese). Japanese Journal of Developmental Psychology, 7(1), 31-40.

Lafreniere, P. J., & Capuano, F. (1997). Preventive Intervention as Means of Clarifying Direction of Effects in Socialization - Anxious-Withdrawn Preschoolers Case. Development and Psychopathology, 9(3), 551-564.

Mackintosh, V. H., Myers, B. J., & Kennon, S. S. (2006). Children of Incarcerated Mothers and Their Caregivers: Factors Affecting the Quality of Their Relationship. Journal of Child & Family Studies, 15(5), 579-594. doi: 10.1007/s10826-006-9030-4.

Magill-Evans, J., & Harrison, M. J. (1999). Parent-child interactions and development of toddlers born preterm. Western Journal of Nursing Research, 21(3), 292-307.

Mäntymaa, M., Puura, K., Luoma, I., Salmelin, R. K., & Tamminen, T. (2006). Mother's early perception of her infant's difficult temperament, parenting stress and early mother-infant interaction. Nordic Journal of Psychiatry, 60(5), 379-386.

McKay, J. M., Pickens, J., & Stewart, A. L. (1996). Inventoried and observed stress in parent-child interactions. Current Psychology: Developmental, Learning, Personality, Social, 15(3), 223-234.

Mills-Koonce, W. R., Propper, C. B., Gariepy, J., Blair, C., Garrett-Peters, P., & Cox, M. J. (2007). Bidirectional genetic and environmental influences on mother and child behavior: the family system as the unit of analyses. Development and Psychopathology, 19(4), 1073-1087. doi: 10.1017/S0954579407000545.

Mylod, D. E., Whitman, T. L., & Borkowski, J. G. (1997). Predicting Adolescent Mothers Transition to Adulthood. Journal of Research on Adolescence, 7(4), 457-478.

Ray, D. C. (2008). Impact of play therapy on parent-child relationship stress at a mental health training setting. British Journal of Guidance & Counseling, 36(2), 165-187.

Reitman, D., Currier, R. O., & Stickle, T. R. (2002). A critical evaluation of the Parenting Stress Index-Short Form (PSI-SF) in a head start population. Journal of Clinical Child & Adolescent Psychology, 31(3), 384-392.

Rholes, W. S., Simpson, J. A., & Friedman, M. (2006). Avoidant attachment and the experience of parenting. Personality and Social Psychology Bulletin, 32(3), 275-285. doi: 10.1177/0146167205280910.

Sawyer, G. K., Di Loreto, A. R., Flood, M. F., & DiLillo, D. (2002, November). Parent-Child Relationship and Family Variables as Predictors of Child Abuse Potential: Implications for Assessment and Early Intervention. Presented at the Annual Convention of the Association for the Advancement of Behavioral Therapy, Poster, Reno, Nevada. Retrieved November 19, 2009.

Schuhmann, E. M., Foote, R. C., Eyberg, S. M., Boggs, S. R., & Algina, J. (1998). Efficacy of Parent-Child Interaction Therapy - Interim-Report of a Randomized Trial with Short-Term Maintenance. Journal of Clinical Child Psychology, 27(1), 34-45.

Sheeber, L. B. (1995). Empirical Dissociations Between Temperament and Behavior Problems - A Response. Journal of Developmental Psychology, 41(4), 554-561.

Sheeber, L. B., & Johnson, J. H. (1994). Evaluation of a Temperament-Focused, Parent-Training Program. Journal of Clinical Child Psychology, 23(3), 249-259.

Strathearn, L., Fonagy, P., Amico, J., & Montague, P. R. (2009). Adult Attachment Predicts Maternal Brain and Oxytocin Response to Infant Cues. Neuropsychopharmacology, 1, 12.

Tarkka, M. T., Paunonen, M., & Laippala, P. (1999). Factors Related to Successful Breast-Feeding by First-Time Mothers When the Child Is 3 Months Old. Journal of Advanced Nursing, 29(1), 113-118.

Timmer, S. G., Borrego, J., & Urquiza, A. J. (2002). Antecedents of Coercive Interactions in Physically Abusive Mother-Child Dyads. J Interpers Violence, 17(8), 836-853. doi: 10.1177/0886260502017008003.

Timmer, S. G., Urquiza, A. J., Zebell, N. M., & McGrath, J. M. (2005). Parent-child interaction therapy: application to maltreating parent-child dyads. Child Abuse & Neglect, 29(7), 825-842. doi: 10.1016/j.chiabu.2005.01.003

Tripp, G., Schaughency, E., Langlands, R., & Mouat, K. (2007). Family Interactions in Children With and Without ADHD. Journal of Child & Family Studies, 16(3), 385-400. doi: 10.1007/s10826-006-9093-2.

Tucker, S., Gross, D., Fogg, L., Delaney, K., & Lapporte, R. (1998). The long-term efficacy of a behavioral parent training intervention for families with 2-year-olds. Research in Nursing & Health, 21(3), 199-210.

Tynan, W. D., & Nearing, J. (1994). The diagnosis of attention deficit hyperactivity disorder in young children. Infants & Young Children, 6(4), 13-20.

Warfield, M. E. (1995). The Cost-Effectiveness of Home Visiting Versus Group Services in Early Intervention. Journal of Early Intervention, 19(2), 130-148.

Webster-Stratton, C. (1994). Advancing Videotape Parent Training - A Comparison Study. Journal of Consulting and Clinical Psychology, 62(3), 583-593.

Weinfield, N. S., Ogawa, J. R., & Egeland, B. (2002). Predictability of observed mother-child interaction from preschool to middle childhood in a high-risk sample. Child development, 528–543.

Whipple, E. E. (1999). Reaching Families with Preschoolers at Risk of Physical Child-Abuse - What Works. Families in Society-The Journal of Contemporary Human Services, 80(2), 148-160.

Overprotection

Janssens, K. A., Oldehinkel, A. J., & Rosmalen, J. G. (2009). Parental overprotection predicts the development of functional somatic symptoms in young adolescents. The Journal of Pediatrics, 154(6), 918-923.

Mullins, L. L., Wolfe-Christensen, C., Hoff Pai, A. L., Carpentier, M. Y., Gillaspy, S., Cheek, J., & Page, M. (2007). The relationship of parental overprotection, perceived child vulnerability, and parenting stress to uncertainty in youth with chronic illness. Journal of Pediatric Psychology, 32(8),973-982.

Program Evaluation

Acton, R. G., & During, S. M. (1992). Preliminary results of aggression management training for aggressive. Journal of Interpersonal Violence, 7(3), 410-417.

Altmaier, E., & Maloney, R. (2007). An initial evaluation of a mindful parenting program. Journal of Clinical Psychology, 63(12), 1231-1238. doi: 10.1002/jclp.20395.

Amankwaa, L., & Pickler, R. (2007). Measuring Maternal Responsiveness. ABNF Journal, 18(1), 4-15. doi: Article.

August, G. J., Lee, S. S., Bloomquist, M. L., Realmuto, G. M., & Hektner, J. M. (2004). Maintenance Effects of an Evidence-Based Prevention Innovation for Aggressive Children Living in Culturally Diverse Urban Neighborhoods: The Early Risers Effectiveness Study. Journal of Emotional & Behavioral Disorders, 12(4), 194-205. doi: Article.

Barnet, B., Duggan, A. K., Devoe, M., & Burrell, L. (2002). The effect of volunteer home visitation for adolescent mothers on parenting and mental health outcomes: A randomized trial. Archives of Pediatrics & Adolescent Medicine, 156(12), 1216-1222.

Barrett, P. M., Farrell, L., Pina, A. A., Peris, T. S., & Piacentini, J. (2008). Evidence-Based Psychosocial Treatments for Child and Adolescent Obsessive-Compulsive Disorder. Journal of Clinical Child & Adolescent Psychology, 37(1), 131-155. doi: 10.1080/15374410701817956.

Bartholomew, L. K., Czyzewski, D. I., Parcel, G. S., Swank, P. R., Sockrider, M. M., Mariotto, M. J., Schidlow, D. V., Fink, R. J., & Seilheimer, D. K. (1997). Self-management of cystic fibrosis: short-term outcomes of the cystic fibrosis family education program. Health Education & Behavior, 24(5), 652-66.

Birkeland, R., Thompson, J. K., & Phares, V. (2005). Adolescent motherhood and postpartum depression. Journal of Clinical Child and Adolescent Psychology, 34(2), 292–300.

Bithoney, W. G., Van Sciver, M. M., Foster, S., Corso, S., & Tentindo, C. (1995). Parental stress and growth outcome in growth-deficient children. Pediatrics, 96(4 Pt 1), 707-11.

Black, M. M. (1995). Failure-to-Thrive - Strategies for Evaluation and Intervention. School Psychology Review, 24(2), 171-185.

Black, M. M., Nair, P., Kight, C., Wachtel, R., Roby, P., & Schuler, M. (1994). Parenting and early development among children of drug-abusing women: effects of home intervention. Pediatrics, 94(4 Pt 1), 440-8.

Bohr, Y. (2005). Infant mental health programs: Experimenting with innovative models- One center's experience with new program funding. Infant Mental Health Journal, 26(5), 407. doi: 10.1002/imhj.20062.

Brotman, L. M., Klein, R. G., Kamboukos, D., Brown, E. J., Coard, S. I., & Sosinsky, L. S. (2003). Preventive intervention for urban, low-income preschoolers at familial risk for conduct problems: A randomized pilot study. Journal of Clinical Child and Adolescent Psychology, 32(2), 246–257.

Browne, J. V., & Talmi, A. (2005). Family-based intervention to enhance infant-parent relationships in the neonatal intensive care unit. Journal of Pediatric Psychology, 30(8), 667-677.

Brunette, M. F., Richardson, F., White, L., Bemis, G., & Eelkema, R. E. (2004). Integrated family treatment for parents with severe psychiatric disabilities. Psychiatric Rehabilitation Journal, 28(2), 177-180. doi: Article.

Butz, A. M., Pulsifer, M., Marano, N., Belcher, H., Lears, M. K., & Royall, R. (2001). Effectiveness of a home intervention for perceived child behavioral problems and parenting stress in children with in utero drug exposure. Archives of Pediatrics & Adolescent Medicine, 155(9), 1029-1037.

Bywater, T., Hutchings, J., Daley, D., Whitaker, C., Yeo, S. T., Jones, K., et al. (2009). Long-term effectiveness of a parenting intervention for children at risk of developing conduct disorder. The British Journal of Psychiatry: The Journal of Mental Science, 195(4), 318-324. doi: 10.1192/bjp.bp.108.056531

Chacko, A., Wymbs, B. T., Flammer, L. M., Pelham, W. E., Walker, K. S., Arnold, F. W., …Herbst, L.(2008). A pilot study of the feasibility and efficacy of the Strategies to Enhance Positive Parenting (STEPP) Program for single mothers of children with ADHD. Journal of Attention Disorders, 12(3), 270-280.

Dadds, M., & Roth, J. (2008). Prevention of Anxiety Disorders: Results of a Universal Trial with Young Children. Journal of Child & Family Studies, 17(3), 320-335. doi: 10.1007/s10826-007-9144-3.

Davis, H., & Spurr, P. (1998). Parent Counseling - An Evaluation of a Community Child Mental-Health-Service. Journal of Child Psychology and Psychiatry and Allied Disciplines, 39(3), 365-376.

Day, C., & Davis, H. (2006). The effectiveness and quality of routine child and adolescent mental health care outreach clinics. British Journal of Clinical Psychology, 45(4), 439-452. doi: 10.1348/014466505X79986.

Dellve, L., Samuelsson, L., Tallborn, A., Fasth, A., & Hallberg, L. R. (2006). Stress and well-being among parents of children with rare diseases: a prospective intervention study. Journal of Advanced Nursing, 53(4), 392-402. doi: 10.1111/j.1365-2648.2006.03736.x.

Dempsey, I., & Keen, D. (2008). A Review of Processes and Outcomes in Family-Centered Services for Children With a Disability. Topics in Early Childhood Special Education, 28(1), 42-52. doi: 10.1177/0271121408316699.

DePanfilis, D., & Dubowitz, H. (2005). Family connections: a program for preventing child neglect. Child Maltreatment, 10(2), 108-123. doi: 10.1177/1077559505275252.

Dombrowski, S. C., Timmer, S. G., Blacker, D. M., & Urquiza, A. J. (2005). A positive behavioural intervention for toddlers: parent-child attunement therapy. Child Abuse Review, 14(2), 132-151.

Duggan, A., Caldera, D., Rodriguez, K., Burrell, L., Rohde, C., & Crowne, S. S. (2007). Impact of a statewide home visiting program to prevent child abuse. Child Abuse & Neglect, 31(8), 801-827. doi: 10.1016/j.chiabu.2006.06.011.

Dukewich, T. L., Borkowski, J. G., & Whitman, T. L. (1996). Adolescent Mothers and Child-Abuse Potential - An Evaluation of Risk-Factors. Child Abuse & Neglect, 20(11), 1031-1047.

El-Kamary, S. S., Higman, S. M., Fuddy, L., McFarlane, E., Sia, C., & Duggan, A. K. (2004). Hawaii's healthy start home visiting program: determinants and impact of rapid repeat birth. Pediatrics, 114(3), e317-326. doi: 10.1542/peds.2004-0618.

Esdaile, S. A. (1996). A play-focused intervention involving mothers of preschoolers. American Journal of Occupational Therapy, 50(2), 113-23.

Evangelou, M., Brooks, G., & Smith, S. (2007). The Birth to School Study: evidence on the effectiveness of PEEP, an early intervention for children at risk of educational under-achievement. Oxford Review of Education, 33(5), 581-609. doi: 10.1080/03054980701476477.

Feinfield, K. A., & Baker, B. L. (2004). Empirical support for a treatment program for families of young children with externalizing problems. Journal of Clinical Child and Adolescent Psychology: The Official Journal for the Society of Clinical Child and Adolescent Psychology, American Psychological Association, Division 53, 33(1), 182-195. doi: 10.1207/S15374424JCCP3301_17

Fey, M. E., Warren, S. F., Brady, N., Finestack, L. H., Bredin-Oja, S. L., Fairchild, M., et al. (2006). Early Effects of Responsivity Education/Prelinguistic Milieu Teaching for

Children With Developmental Delays and Their Parents. Journal of Speech, Language & Hearing Research, 49(3), 526-547. doi: 10.1044/1092-4388(2006/039).

Finnstevenson, M., Desimone, L., & Chung, A. M. (1998). Linking Child-Care and Support Services with the School - Pilot Evaluation of the School of the 21st-Century. Children and Youth Services Review, 20(3), 177-205.

Forde, H., Lane, H., McCloskey, D., McManus, V., & Tierney, E. (2004). Link Family Support- an evaluation of an in-home support service. Journal of Psychiatric & Mental Health Nursing, 11(6), 698-704.

Francis-Williams, N.(2005). Evaluation of a faith-based socioemotional support program for parents of African American youth with antisocial behaviors. (Unpublished doctoral dissertation). Nova Southeastern University, Florida.

Fuscaldo, D., Kaye, J. W., & Philliber, S. (1998). Evaluation of a Program for Parenting. Families in Society:The Journal of Contemporary Human Services, 79(1), 53-61.

Gelfand, D. M., Teti, D. M., Seiner, S. A., & Jameson, P. B. (1996). Helping Mothers Fight Depression - Evaluation of a Home-Based Intervention Program for Depressed Mothers and Their Infants. Journal of Clinical Child Psychology, 25(4), 406-422.

Glavin, K., Smith, L., Sørum, R., & Ellefsen, B. (2010). Redesigned community postpartum care to prevent and treat postpartum depression in women - a one-year follow-up study. Journal of Clinical Nursing, 19, 3051-3062.

Goldman, J., Sorensen, E., & Ward, M. (1995). Brief Child Assessment Battery to Assist with Treatment Planning and Program-Evaluation. Community Mental Health Journal, 31(5), 437-448.

Gorzka, P. A. (1999). Homeless parents: parenting education to prevent abusive behaviors. Journal of Child & Adolescent Psychiatric Nursing, 12(3), 101-9.

Gross, D., Fogg, L., & Tucker, S. (1995). The Efficacy of Parent Training for Promoting Positive Parent Toddler Relationships. Research in Nursing & Health, 18(6), 489-499.

Hutchings, J., Bywater, T., Daley, D., Gardner, F., Whitaker, C., Jones, K.,... Edwards, R. T. (2007). Parenting intervention in Sure Start services for children at risk of developing conduct disorder: pragmatic randomized controlled trial. British Medical Journal, doi:10.1136/bmj.39126.620799.55.

Janus, M., & Goldberg, S. (1997). Factors Influencing Family Participation in a Longitudinal-Study - Comparison of Pediatric and Healthy Samples. Journal of Pediatric Psychology, 22(2), 245-262.

Kishchuk, N., Laurendeau, M. C., Desjardins, N., & Perreault, R. (1995). Parental Support - Effects of a Mass-Media Intervention. Canadian Journal of Public Health, 86(2), 128-132.

Magura, S., Laudet, A., Kang, S.-Y., & Whitney, S. A. (1999). Effectiveness of comprehensive services for crack-dependent mothers with newborns and young children. Journal of Psychoactive Drugs, 31(4), 321-338.

Margalit, M., & Kleitman, T. (2006). Mothers' stress, resilience and early intervention. European Journal of Special Needs Education, 21(3), 269-283.

Meager, I., & Milgrom, J. (1996). Group treatment for postpartum depression: A pilot study. Australian & New Zealand Journal of Psychiatry, 30(6), 852-860.

Meijssen, D., Wolf, M., Koldewijn, K., van Wassenaer, A., Kok, J., & van Baar, A. (2010). Parenting stress in mothers after very preterm birth and the effect of the Infant Behavioural Assessment and Intervention Program. Child: care, health and development, 37, 195-202.

Milner, J. S. (1994). Assessing Physical Child-Abuse Risk - The Child-Abuse Potential Inventory. Clinical Psychology Review, 14(6), 547-583.

Morrell, C. J., Slade, P., Warner, R., Paley, G., Dixon, S., Walters, S. J., … Nicholl, J. (2009). Clinical effectiveness of health visitor training in psychologically informed approaches for depression in postnatal women: pragmatic cluster randomised trial in primary care. British Medical Journal, 338:a3045.

Msall, M. E., Rogers, B. T., Ripstein, H., Lyon, N., & Wilczenski, F. (1997). Measurements of Functional Outcomes in Children with Cerebral-Palsy. Mental Retardation and Developmental Disabilities Research Reviews, 3(2), 194-203.

Mudge, K., & Langley, J. (1998). Successful parenting: how to live with children. Community Practitioner, 71(9), 289-91.

Naik-Polan, A. T., & Budd, K.S. (2008). Stimulus generalization of parenting skills during parent-child interaction therapy. Journal of Early and Intensive Behavior Intervention, 5(3), 71-92.

Nereo, N. E., Fee, R. J., & Hinton, V. J. (2003). Parental stress in mothers of boys with Duchenne muscular dystrophy. Journal of Pediatric Psychology, 28(7), 473-484.

Nievar, M. A, Jacobson, A., & Dier, S. (2008, November). Home visiting for at-risk preschoolers: A successful model for Latino families. Paper presented at the Annual Meeting of the National Council on Family Relations, Little Rock, Arkansas.

Nixon, R. D., Sweeney, L., Erickson, D. B., & Touyz, S. W. (2003). Parent-child interaction therapy: A comparison of standard and abbreviated treatments for oppositional defiant preschoolers. Journal of Consulting and Clinical Psychology, 71(2), 251–260.

Olsen, L., & DeBoise, T. (2007). Enhancing School Readiness: The Early Head Start Model. Children & Schools, 29(1), 47. doi: Article.

Owen, M. T., & Mulvihill, B. A. (1994). Benefits of a Parent Education and Support Program in the 1st 3 Years. Family Relations, 43(2), 206-212.

Patterson, J., Barlow, J., Mockford, C., Klimes, I., Pyper, C., & Stewart-Brown, S. (2002). Improving mental health through parenting programmes: block randomized controlled trial. Archives of Disease in Childhood, 87(6), 472-477.

Perepletchikova, F., & Kazdin, A. E. (2004). Assessment of Parenting Practices Related to Conduct Problems: Development and Validation of the Management of Children's Behavior Scale. Journal of Child & Family Studies, 13(4), 385-403. doi: Article.

Quinn, M., Carr, A., Carroll, L., & O Sullivan, D. (2006). An evaluation of the Parents Plus Programme for pre-school children with conduct problems: A comparison of those with and without developmental disabilities. Irish Journal of Psychology, 27(3/4), 168-182.

Quinn, M., Carr, A., Carroll, L., & O'Sullivan, D. (2007). Parents Plus Programme 1: Evaluation of its effectivesness for pre-school children with developmental disabilities and behavioral problems. Journal of Applied Research in Intellectual Disabilities, 20(4), 345-359.

Rodrigue, J. R., Macnaughton, K., Hoffmann, R. G., Grahampole, J., Andres, J. M., Novak, D. A., & Fennell, R. S. (1997). Transplantation in Children - A Longitudinal Assessment of Mothers Stress, Coping, and Perceptions of Family Functioning. Psychosomatics, 38(5), 478-486.

Sheeber, L. B., & Johnson, J. H. (1994). Evaluation of a Temperament-Focused, Parent-Training Program. Journal of Clinical Child Psychology, 23(3), 249-259.

Tannock, R., Girolametto, L., & Siegel, L. S. (1992). Language intervention with children who have developmental delays: effects of an interactive approach. American Journal of Mental Retardation, 97(2), 145-60.

Tucker, S., Gross, D., Fogg, L., Delaney, K., & Lapporte, R. (1998). The long-term efficacy of a behavioral parent training intervention for families with 2-year-olds. Research in Nursing & Health, 21(3), 199-210.

Van Hasselt, V. B., Hersen, M., Null, J. A., Ammerman, R. T., Bukstein, O. G., Mcgillivray, J., & Hunter, A. (1993). Drug-Abuse Prevention for High-Risk African-American Children and Their Families - A Review and Model Program. Addictive Behaviors, 18(2), 213-234.

Behavioral Parent Training

Acton, R. G., & During, S. M. (1992). Preliminary results of aggression management training for aggressive. Journal of Interpersonal Violence, 7(3), 410-417.

Anastopoulos, A. D., Shelton, T. L., DuPaul, G. J., & Guevremont, D. C. (1993). Parent training for attention-deficit hyperactivity disorder: Its impact on parent functioning. Journal of Abnormal Child Psychology, 21(5).

Bagner, D. M., & Eyberg, S. M. (2007). Parent—Child Interaction Therapy for Disruptive Behavior in Children with Mental Retardation: A Randomized Controlled Trial. Journal of Clinical Child & Adolescent Psychology, 36(3), 418-429. doi: 10.1080/15374410701448448.

Capage, L. C., Bennett, G., & McNeil, C. B. (2001). A comparison between African American and Caucasian children referred for treatment of disruptive behavior disorders. Child & Family Behavior Therapy, 23(1), 1-14.

Costin, J., & Chambers, S. M. (2007). Parent management training as a treatment for children with oppositional defiant disorder referred to a mental health clinic. Clinical Child Psychology and Psychiatry, 12(4), 511-524.

Danforth, J. S. (1998). The Outcome of Parent Training Using the Behavior Management Flow-Chart with Mothers and Their Children with Oppositional Defiant Disorder and Attention-Deficit Hyperactivity Disorder. Behavior Modification, 22(4), 443-473.

Doubleday, E., & Hey, C. (2004). Is statistical significance enough? Behavioural parent training programmes in real life. Clinical Psychology, 37, 5-9.

Dumka, L. E., Gonzales, N. A., Wood, J. L., & Formoso, D. (1998). Using Qualitative Methods to Develop Contextually Relevant Measures and Preventive Interventions - An Illustration. American Journal of Community Psychology, 26(4), 605-637.

Eisenstadt, T. H., Eyberg, S., Mcneil, C. B., Newcomb, K., & Funderburk, B. (1993). Parent-Child Interaction Therapy with Behavior Problem Children - Relative Effectiveness of 2 Stages and Overall Treatment Outcome. Journal of Clinical Child Psychology, 22(1), 42-51.

Eiserman, W. D., Weber, C., & McCoun, M. (1992). 2 Alternative Program Models for Serving Speech-Disordered Preschoolers - A 2nd Year Follow-Up. Journal of Communication Disorders, 25(2-3), 77-106.

Feldman, M. A., Varghese, J., Ramsay, J., & Rajska, D. (2002). Relationships between social support, stress and mother-child interactions in mothers with intellectual disabilities. Journal of Applied Research in Intellectual Disabiliites, 15(4), 314-323.

Fitzgerald, M., Butler, B., & Kinsella, A. (1990). The burden on a family having a child with special needs. Irish Journal of Psychological Medicine, 7(2), 109-113.

Gross, D., Fogg, L., & Tucker, S. (1995). The Efficacy of Parent Training for Promoting Positive Parent Toddler Relationships. Research in Nursing & Health, 18(6), 489-499.

Kazdin, A. E., Siegel, T. C., & Bass, D. (1992). Cognitive problem-solving skills training and parent management training in the treatment of antisocial behavior in children. Journal of Consulting and Clinical Psychology, 60, 733-747.

Lafreniere, P. J., & Capuano, F. (1997). Preventive Intervention as Means of Clarifying Direction of Effects in Socialization - Anxious-Withdrawn Preschoolers Case. Development and Psychopathology, 9(3), 551-564.

Landreth, G. L., & Lobaugh, A. F. (1998). Filial Therapy with Incarcerated Fathers - Effects on Parental Acceptance of Child, Parental Stress, and Child Adjustment. Journal of Counseling and Development, 76(2), 157-165.

Lauth, G. W., Otte, T., & Heubeck, B. G. (2009). Effectiveness of a competence training programme for parents of socially disruptive children. Emotional & Behavioural Difficulties, 14(2), 117-126.

Lees, D. G., & Ronan, K. R. (2008). Engagement and Effectiveness of Parent Management Training (Incredible Years) for Solo High-Risk Mothers: A Multiple Baseline Evaluation. Behaviour Change, 25(2), 109-128. doi: 10.1375/bech.25.2.109.

Lutzker, J. R., Bigelow, K. M., Doctor, R. M., & Kessler, M. L. (1998). Safety, health care, and bonding within an ecobehavioral approach to treating and preventing child abuse and neglect. Journal of Family Violence, 13(2), 163-185.

Nicholson, B., Anderson, M., Fox, R., & Brenner, V. (2002). One Family at a Time: a prevention program for at-risk parents. Journal of Counseling & Development, 80(3), 362-371.

Robbins, F. R., & Dunlap, G. (1992). Effects of task difficulty on parent teaching skills and behavior problems of young children with autism. American Journal on Mental Retardation, 96(6), 631-643.

Russell, D., & Matson, J. (1998). Fathers as Intervention Agents for Their Children with Developmental-Disabilities. Child & Family Behavior Therapy, 20(3), 29-49.

Sheeber, L. B. (1995). Empirical Dissociations Between Temperament and Behavior Problems - A Response. Journal of Developmental Psychology, 41(4), 554-561.

Sheeber, L. B., & Johnson, J. H. (1994). Evaluation of a Temperament-Focused, Parent-Training Program. Journal of Clinical Child Psychology, 23(3), 249-259.

Taubman, B., Blum, N. J., & Nemeth, N. (2003). Stool toileting refusal: a prospective intervention targeting parental behavior. Archives of Pediatrics & Adolescent Medicine, 157(12), 1193-1196. doi: 10.1001/archpedi.157.12.1193

Tucker, S., Gross, D., Fogg, L., Delaney, K., & Lapporte, R. (1998). The long-term efficacy of a behavioral parent training intervention for families with 2-year-olds. Research in Nursing & Health, 21(3), 199-210.

Volenski, L. T. (1995). Building school support systems for parents of handicapped children: The parent education and guidance program. Psychology in the Schools, 32(2), 124-129.

Webster-Stratton, C. (1994). Advancing Videotape Parent Training - A Comparison Study. Journal of Consulting and Clinical Psychology, 62(3), 583-593.

Webster-Stratton, C., & Hammond, M. (1997). Treating Children with Early-Onset Conduct Problems - A Comparison of Child and Parent Training Interventions. Journal of Consulting and Clinical Psychology, 65(1), 93-109.

Webster-Stratton, C., & Herman, K. C. (2008). The Impact of Parent Behavior-Management Training on Child Depressive Symptoms. Journal of Counseling Psychology, 55(4), 473-484.

Wong, V., & Kwan, Q. (2009). Randomized control trial for early intervention for Autism: A pilot study of the Autism 1-2-3 Project. Journal of Autism and Developmental Disorders, 40, 677-688.

Cognitive Behavioral Therapy

Acton, R. G., & During, S. M. (1992). Preliminary results of aggression management training for aggressive. Journal of Interpersonal Violence, 7(3), 410-417.

Eccleston, C., Malleson, P. N., Clinch, J., Connell, H., & Sourbut, C. (2003). Chronic pain in adolescents: evaluation of a programme of interdisciplinary cognitive behaviour therapy. Archives of Disease in Childhood, 88(10), 881-885.

Fischel, J. E., Whitehurst, G. J., Caulfield, M. B., & DeBaryshe, B. (1989). Language growth in children with expressive language delay. Pediatrics, 83(2), 218-27.

Greaves, D. (1997). The effect of rational-emotive parent education on the stress of mothers of young children with Down syndrome. Journal of Rational-Emotive & Cognitive Behavior Therapy, 15(4), 249-267.

Kazak, A. E., Penati, B., Boyer, B. A., Himelstein, B., Brophy, P., Waibel, M. K., Blackall, G. F., Daller, R., & Johnson, K. (1996). A Randomized Controlled Prospective Outcome Study of a Psychological and Pharmacological Intervention Protocol for Procedural Distress in Pediatric Leukemia. Journal of Pediatric Psychology, 21(5), 615-631.

Kazdin, A. E. (1995). Child, Parent and Family Dysfunction as Predictors of Outcome in Cognitive-Behavioral Treatment of Antisocial Children. Behaviour Research and Therapy, 33(3), 271-281.

Kazdin, A. E., & Crowley, M. J. (1997). Moderators of Treatment Outcome in Cognitively Based Treatment of Antisocial Children. Cognitive Therapy and Research, 21(2), 185-207.

Meager, I., & Milgrom, J. (1996). Group treatment for postpartum depression: A pilot study. Australian & New Zealand Journal of Psychiatry, 30(6), 852-860.

Ooi, Y. P., Lam, C. M., Sung, M., Tan, W. T. S., Goh, T. J., Fung, D. S. S., et al. (2008). Effects of cognitive-behavioural therapy on anxiety for children with high-functioning autistic spectrum disorders. Singapore Medical Journal, 49(3), 215-220.

Taylor, H. G., Klein, N., Schatschneider, C., & Hack, M. (1998). Predictors of Early School-Age Outcomes in Very-Low-Birth-Weight Children. Journal of Developmental and Behavioral Pediatrics, 19(4), 235-243.

Victor, A. M., Bernat, D. H., Bernstein, G. A., & Layne, A. E. (2007). Effects of parent and family characteristics on treatment outcome of anxious children. Journal of Anxiety Disorders, 21(6), 835-848. doi: 10.1016/j.janxdis.2006.11.005.

Wong, F., & Poon, A. (2010). Cognitive behavioural group treatment for Chinese parents with children with developmental disabilities in Melbourne, Australia: An efficacy study, Australian and New Zealand Journal of Psychiatry, 44, 742-749.

Evidence-Based Treatment

Harwood, M. D., & Eyberg, S. M. (2004). Therapist Verbal Behavior Early in Treatment: Relation to Successful Completion of Parent-Child Interaction Therapy. Journal of Clinical Child & Adolescent Psychology, 33(3), 601-612. doi: Article.

Hutchings, J., Lane, E., & Kelly, J. (2004). Comparison of two treatments for children with severely disruptive behaviors: A four-year follow-up. Behavioural and Cognitive Psychotherapy, 32(1), 15-30. doi: 10.1017/S1352465804001018.

Lipman, E. L., Boyle, M. H., Cunningham, C., Kenny, M., Sniderman, C., Duku, E., et al. (2006). Testing effectiveness of a community-based aggression management program for children 7 to 11 years old and their families. Journal of the American Academy of Child and Adolescent Psychiatry, 45(9), 1085-1093. doi: 10.1097/01.chi.0000228132.64579.73.

McCabe, K., & Yeh, M. (2009). Parent–Child Interaction Therapy for Mexican Americans: A Randomized Clinical Trial. Journal of Clinical Child & Adolescent Psychology, 38(5), 753. doi: 10.1080/15374410903103544

Nixon, R. D. V. (2001). Changes in hyperactivity and temperament in behaviourally disturbed preschoolers after Parent-Child Interaction Therapy (PCIT). Behaviour Change, 18 (3), 168-176.

Nixon, R. D. V., Sweeney, L., Erickson, D. B., & Touyz, S. W. (2004). Parent-child interaction therapy: one- and two-year follow-up of standard and abbreviated treatments for oppositional preschoolers. Journal of Abnormal Child Psychology, 32(3), 263-271.

Phillips, J., Morgan, S., Cawthorne, K., & Barnett, B. (2008). Pilot evaluation of parent-child interaction therapy delivered in an Australian community early childhood clinic setting. Australian & New Zealand Journal of Psychiatry, 42(8), 712-719. doi: 10.1080/00048670802206320.

Pincus, D. B., Eyberg, S. M., & Choate, M. L. (2005). Adapting Parent-Child Interaction Therapy for Young Children with Separation Anxiety Disorder. Education & Treatment of Children, 28(2), 163. doi: Article.

Scott, K., & Crooks, C. V. (2007). Preliminary Evaluation of an Intervention Program for Maltreating Fathers. Brief Treatment and Crisis Intervention, 7(3), 224-238. doi: 10.1093/brief-treatment/mhm007.

Silovsky, J. F., Niec, L., Bard, D., & Hecht, D. B. (2007). Treatment for Preschool Children With Interpersonal Sexual Behavior Problems: A Pilot Study. Journal of Clinical Child & Adolescent Psychology, 36(3), 378-391. doi: 10.1080/15374410701444330.

Solomon, M., Ono, M., Timmer, S., & Goodlin-Jones, B. (2008). The Effectiveness of Parent–Child Interaction Therapy for Families of Children on the Autism Spectrum.. Journal of Autism & Developmental Disorders, 38(9), 1767-1776. doi: 10.1007/s10803-008-0567-5.

Timmer, S. G., Llrquiza, A. I., Herschell, A. D., McGrath, J. M., Zebell, N. M., Porter, A. L., et al. (2006). Parent-Child Interaction Therapy: Application of an Empirically Supported Treatment to Maltreated Children in Foster Care. Child Welfare, 85(6), 919. doi: Article.

Timmer, S. G., Sedlar, G., & Urquiza, A. J. (2004). Challenging children in kin versus nonkin foster care: perceived costs and benefits to caregivers. Child Maltreatment, 9(3), 251-262. doi: 10.1177/1077559504266998.

Timmer, S. G., Urquiza, A. J., & Zebell, N. (2006). Challenging foster caregiver–maltreated child relationships: The effectiveness of parent–child interaction therapy. Children and Youth Services Review, 28(1), 1-19. doi: 10.1016/j.childyouth.2005.01.006.

White, C., & Verduyn, C. (2006). The Children And Parents Service (CAPS): a multi-agency early intervention initiative for young children and their families. Child and Adolescent Mental Health, 11(4), 192. doi: 10.1111/j.1475-3588.2006.00410.x.

Family/Filial Therapy

Athanasiou, M. S., & Gunning, M. P. (1999). Filial therapy: effects on two children's behavior and mothers' stress. Psychological Reports, 84(2), 587-90.

Costas, M. B. (1999). Filial therapy with non-offending parents of children who have been sexually abused. Dissertation Abstracts International, 59(7-A).

Jang, M. (2000). Effectiveness of filial therapy for Korean parents. International Journal of Play Therapy, 9(2), 39-56

Landreth, G. L., & Lobaugh, A. F. (1998). Filial Therapy with Incarcerated Fathers - Effects on Parental Acceptance of Child, Parental Stress, and Child Adjustment. Journal of Counseling and Development, 76(2), 157-165.

Parent Education

Anastopoulos, A. D., Shelton, T. L., DuPaul, G. J., & Guevremont, D. C. (1993). Parent training for attention-deficit hyperactivity disorder: Its impact on parent functioning. Journal of Abnormal Child Psychology, 21(5).

Conners, N. A., Edwards, M. C., & S. Grant, A. (2007). An Evaluation of a Parenting Class Curriculum for Parents of Young Children: Parenting the Strong-Willed Child. Journal of Child & Family Studies, 16(3), 321-330. doi: 10.1007/s10826-006-9088-z.

Cowan, P. A., Cowan, C. P., Pruett, M. K., Pruett, K., & Wong, J. J. (2009). Promoting Fathers' Engagement With Children: Preventive Interventions for Low-Income Families. Journal of Marriage and Family, 71(3), 663-679. doi: 10.1111/j.1741-3737.2009.00625.x.

Danforth, J. S. (1998). The Outcome of Parent Training Using the Behavior Management Flow-Chart with Mothers and Their Children with Oppositional Defiant Disorder and Attention-Deficit Hyperactivity Disorder. Behavior Modification, 22(4), 443-473.

Dumka, L. E., Gonzales, N. A., Wood, J. L., & Formoso, D. (1998). Using Qualitative Methods to Develop Contextually Relevant Measures and Preventive Interventions - An Illustration. American Journal of Community Psychology, 26(4), 605-637.

Eiserman, W. D., Weber, C., & McCoun, M. (1992). 2 Alternative Program Models for Serving Speech-Disordered Preschoolers - A 2nd Year Follow-Up. Journal of Communication Disorders, 25(2-3), 77-106.

Eiserman, W. D., Weber, C., & McCoun, M. (1995). Parent and Professional Roles in Early Intervention - A Longitudinal Comparison of the Effects of 2 Intervention Configurations. Journal of Special Education, 29(1), 20-44.

Feldman, M. A., & Waltonallen, N. (1997). Effects of Maternal Mental-Retardation and Poverty on Intellectual, Academic, and Behavioral Status of School-Age-Children. American Journal on Mental Retardation, 101(4), 352-364.

Gershater-Molko, R. M., Lutzker, J. R., & Wesch, D. (2002). Using recidivism data to evaluate Project SafeCare: teaching bonding, safety, and health care skills to parents. Child Maltreatment, 7(3), 277-285.

Greaves, D. (1997). The effect of rational-emotive parent education on the stress of mothers of young children with Down syndrome. Journal of Rational-Emotive & Cognitive Behavior Therapy, 15(4), 249-267.

Gross, D., Fogg, L., & Tucker, S. (1995). The Efficacy of Parent Training for Promoting Positive Parent Toddler Relationships. Research in Nursing & Health, 18(6), 489-499.

Kern, J. K., West, E. Y., Grannemann, B. D., Greer, T., Snell, L. M., Cline, L. L., …Trivedi, M. H. (2004). Reduction in stress and depressive symptoms in mothers of substance-exposed infants, participating in a psychosocial program. Maternal & Child Health Journal, 8(3), 127-136.

Landreth, G. L., & Lobaugh, A. F. (1998). Filial Therapy with Incarcerated Fathers - Effects on Parental Acceptance of Child, Parental Stress, and Child Adjustment. Journal of Counseling and Development, 76(2), 157-165.

Lutzker, J. R., Bigelow, K. M., Doctor, R. M., & Kessler, M. L. (1998). Safety, health care, and bonding within an ecobehavioral approach to treating and preventing child abuse and neglect. Journal of Family Violence, 13(2), 163-185.

McBride, B. A. (1991). Parental support programs and paternal stress: An exploratory study. Early Childhood Research Quarterly, 6(2), 137-140.

Miles, R., Cowan, F., Glover, V., Stevenson, J., & Modi, N. (2006). A controlled trial of skin-to-skin contact in extremely preterm infants. Early Human Development, 82(7), 447-455. doi: 10.1016/j.earlhumdev.2005.11.008.

Owen, M. T., & Mulvihill, B. A. (1994). Benefits of a Parent Education and Support Program in the 1st 3 Years. Family Relations, 43(2), 206-212.

Realmuto, G. M., August, G. J., & Egan, E. A. (2004). Testing the goodness-of-fit of a multifaceted preventive intervention for children at risk for conduct disorder. Canadian Journal of Psychiatry. Revue Canadienne De Psychiatrie, 49(11), 743-752.

Robbins, F. R., & Dunlap, G. (1992). Effects of task difficulty on parent teaching skills and behavior problems of young children with autism. American Journal on Mental Retardation, 96(6), 631-643.

Robbins, F. R., Dunlap, G., & Plienis, A. J. (1991). Family characteristics, family training, and the progress of young children with autism. Journal of Early Intervention, 15(2), 173-184.

Russell, D., & Matson, J. (1998). Fathers as Intervention Agents for Their Children with Developmental-Disabilities. Child & Family Behavior Therapy, 20(3), 29-49.

Selkirk, R., McLaren, S., Ollerenshaw, A., McLachlan, A. J., & Moten, J. (2006). The longitudinal effects of midwife-led postnatal debriefing on the psychological health of mothers. Journal of Reproductive & Infant Psychology, 24(2), 133-147. doi: 10.1080/02646830600643916.

Sheeber, L. B., & Johnson, J. H. (1994). Evaluation of a Temperament-Focused, Parent-Training Program. Journal of Clinical Child Psychology, 23(3), 249-259.

Timmer, S. G., Urquiza, A. J., Zebell, N. M., & McGrath, J. M. (2005). Parent-child interaction therapy: application to maltreating parent-child dyads. Child Abuse & Neglect, 29(7), 825-842. doi: 10.1016/j.chiabu.2005.01.003

Tsang, S. K. M., & Leung, C. (2005). Developing a database for evaluating the effectiveness of parent education and support programs: Results of a pilot study. Illinois Child Welfare Journal, 2(1-2), 77–89.

Tucker, S., Gross, D., Fogg, L., Delaney, K., & Lapporte, R. (1998). The long-term efficacy of a behavioral parent training intervention for families with 2-year-olds. Research in Nursing & Health, 21(3), 199-210.

Volenski, L. T. (1995). Building school support systems for parents of handicapped children: The parent education and guidance program. Psychology in the Schools, 32(2), 124-129.

Warren, S. F., Fey, M. E., Finestack, L. H., Brady, N. C., Bredin-Oja, S. L., & Fleming, K. K. (2008). A Randomized Trial of Longitudinal Effects of Low-Intensity Responsivity Education/Prelinguistic Milieu Teaching. Journal of Speech, Language & Hearing Research, 51(2), 451-470. doi: 10.1044/1092-4388(2008/033).

Webster-Stratton, C. (1994). Advancing Videotape Parent Training - A Comparison Study. Journal of Consulting and Clinical Psychology, 62(3), 583-593.

Webster-Stratton, C., & Hammond, M. (1997). Treating Children with Early-Onset Conduct Problems - A Comparison of Child and Parent Training Interventions. Journal of Consulting and Clinical Psychology, 65(1), 93-109.

Whipple, E. E. (1999). Reaching Families with Preschoolers at Risk of Physical Child-Abuse - What Works. Families in Society-The Journal of Contemporary Human Services, 80(2), 148-160.

Wolfe, R. B., & Hirsch, B. J. (2003). Outcomes of parent education programs based on reevaluation counseling. Journal of Child & Family Studies, 12(1), 61-76.

Parent Groups

Huebner, C. E. (2002). Evaluation of a clinic-based parent education program to reduce the risk of infant and toddler maltreatment. Public Health Nursing, 19(5), 377–389.

Kemp, L., Harris, E., McMahon, C., Matthey, S., Vimpani, G., Anderson, T., et al. (2008). Miller Early Childhood Sustained Home-visiting (MECSH) trial: design, method and sample description. BMC Public Health, 8, 1-12. doi: 10.1186/1471-2458-8-424.

Krauss, M. W., Upshur, C. C., Shonkoff, J. P., & Hausercram, P. (1993). The Impact of Parent Groups on Mothers of Infants with Disabilities. Journal of Early Intervention, 17(1), 8-20.

Rodger, S., Keen, D., Braithwaite, M., & Cook, S. (2008). Mothers' Satisfaction with a Home Based Early Intervention Programme for Children with ASD. Journal of Applied Research in Intellectual Disabilities, 21(2), 174-182. doi: 10.1111/j.1468-3148.2007.00393.x.

Sawyer, G. K., Yancey, C. T., Tsao, E. H., Wynne, A., Hansen, D. J., & Flood, M. F. (2005). Parallel group treatments for sexually abused youth and their nonoffending parents: Treatment integrity, outcomes and social validity of Project SAFE. Presented at the 39th Annual Convention of the Association for Behavioral and Cognitive Therapies, Washington, DC.

Stewart-Brown, S., Patterson, J., Mockford, C., Barlow, J., Klimes, I., & Pyper, C. (2004). Impact of a general practice based group parenting programme: quantitative and qualitative results from a controlled trial at 12 months. Archives of Disease in Childhood, 89(6), 519-525.

Taft, A. J., Small, R., Hegarty, K. L., Lumley, J., Watson, L. F., & Gold, L. (2009). MOSAIC (Mothers' Advocates In the Community): protocol and sample description of a cluster randomised trial of mentor mother support to reduce intimate partner violence among pregnant of recent mothers. BMC Public Health, 9, 1-13. doi: 10.1186/1471-2458-9-159.

Thome, M., & Skuladottir, A. (2005). Evaluating a family-centred intervention for infant sleep problems. Journal of Advanced Nursing, 50(1), 5-11. doi: 10.1111/j.1365-2648.2004.03343.x.

Play Therapy/Attachment Therapy

Ray, D. C. (2008). Impact of play therapy on parent-child relationship stress at a mental health training setting. British Journal of Guidance & Counseling, 36(2), 165-187.

Wong, V., & Kwan, Q. (2009). Randomized control trial for early intervention for Autism: A pilot study of the Autism 1-2-3 Project. Journal of Autism and Developmental Disorders, 40, 677-688.

Premature Termination

Chisholm, V., Atkinson, L., Donaldson, C., Noyes, K., Payne, A., & Kelnar, C. (2007). Predictors of treatment adherence in young children with type 1 diabetes. Journal of Advanced Nursing, 57(5), 482-493. doi: 10.1111/j.1365-2648.2006.04137.x.

Dadds, M., & Roth, J. (2008). Prevention of Anxiety Disorders: Results of a Universal Trial with Young Children. Journal of Child & Family Studies, 17(3), 320-335. doi: 10.1007/s10826-007-9144-3.

DeMore, M., Adams, C., Wilson, N., & Hogan, M. B. (2005). Parenting Stress, Difficult Child Behavior, and Use of Routines in Relation to Adherence in Pediatric Asthma. Children's Health Care, 34(4), 245-259. doi: 10.1207/s15326888chc3404_1.

Friars, P., & Mellor, D. (2007). Drop Out from Behavioral Management Training Programs for ADHD: A Prospective Study. Journal of Child & Family Studies, 16(3), 427-441. doi: 10.1007/s10826-006-9096-z.

Harwood, M. D., & Eyberg, S. M. (2004). Therapist Verbal Behavior Early in Treatment: Relation to Successful Completion of Parent-Child Interaction Therapy. Journal of Clinical Child & Adolescent Psychology, 33(3), 601-612. doi: Article.

Janus, M., & Goldberg, S. (1997). Factors Influencing Family Participation in a Longitudinal-Study - Comparison of Pediatric and Healthy Samples. Journal of Pediatric Psychology, 22(2), 245-262.

Kazdin, A. E. (1994). Family adversity, socioeconomic disadvantage, and parental stress: Contextual variables related to premature termination from child behavior therapy. Psicologia Conductual, 2(1), 5-21.

Kazdin, A. E., & Wassell, G. (1998). Treatment Completion and Therapeutic Change Among Children Referred for Outpatient Therapy. Professional Psychology-Research and Practice, 29(4), 332-340.

Kazdin, A. E., Holland, L., & Crowley, M. (1997). Family Experience of Barriers to Treatment and Premature Termination from Child Therapy. Journal of Consulting and Clinical Psychology, 65(3), 453-463.

Kazdin, A. E., Holland, L., Crowley, M., & Breton, S. (1997). Barriers to Treatment Participation Scale - Evaluation and Validation in the Context of Child Outpatient Treatment. Journal of Child Psychology and Psychiatry and Allied Disciplines, 38(8), 1051-1062.

Kazdin, A. E., Mazurick, J. L., & Bass, D. (1993). Risk for Attrition in Treatment of Antisocial Children and Families. Journal of Clinical Child Psychology, 22(1), 2-16.

Kazdin, A. E., Mazurick, J. L., & Siegel, T. C. (1994). Treatment Outcome Among Children with Externalizing Disorder Who Terminate Prematurely Versus Those Who Complete Psychotherapy. Journal of the American Academy of Child and Adolescent Psychiatry, 33(4), 549-557.

Keeley, M., & Wiens, B. (2008). Family Influences on Treatment Refusal in School-linked Mental Health Services. Journal of Child & Family Studies, 17(1), 109-126. doi: 10.1007/s10826-007-9141-6.

Roberts, A. C., & Nishimoto, R. (2006). Barriers to engaging and retaining African-American post-partum women in drug treatment. Journal of Drug Issues, 36(1), 53. doi: Article.

Unger, D. G., Jones, C. W., Park, E., & Tressell, P. A. (2001). Promoting Involvement Between Low-Income Single Caregivers and Urban Early Intervention Programs. Topics in Early Childhood Special Education, 21(4), 197. doi: Article.

Treatment Acceptability/Expectancies

Nock, M. K., & Kazdin, A. E. (2001). Parent expectancies for child therapy: Assessment and relation to participation in treatment. Journal of Child and Family Studies, 10(2), 155–180.

Pemberton, J. R., & Borrego, J. (2005). The relationship between treatment acceptability and familism. International Journal of Behavioral Consultation and Therapy, 1(4), 329-337.

Psychometrics/Clinical Cut-offs

Doubleday, E., & Hey, C. (2004). Is statistical significance enough? Behavioural parent training programmes in real life. Clinical Psychology, 37, 5-9.

Eisengart, S. P., Singer, L. T., Kirchner, H. L., Min, M. O., Fulton, S., Short, E. J., et al. (2006). Factor structure of coping: two studies of mothers with high levels of life stress. Psychological Assessment, 18(3), 278-288. doi: 10.1037/1040-3590.18.3.278.

Fedele, D., Grant, D., Wolfe-Christensen, C., Mullins, L., & Ryan, J. (2010). An examination of the factor structure of parenting capacity measures in chronic illness populations, Journal of Pediatric Psychology, 35, 1083-1092.

McKelvey, L. M., Whiteside-Mansell, L., Faldowski, R. A., Shears, J., Ayoub, C., & Hart, A. D. (2009). Validity of the short form of the parenting stress index for fathers of toddlers. Journal of Child and Family Studies, 18(1), 102-111.

Preschool/Head Start

Adamakos, H., Kathleen, R., G., U. D., & John, P. (1986). Maternal social support as a predictor of mother-child stress and stimulation. Child Abuse & Neglect, 10(4), 463-470.

Allen, K. D., Maguire, K. B., Williams, G. E., & Sanger, W. G. (1996). The effects of infertility on parent-child relationships and adjustment. Children's Health Care, 25(2), 93-105.

Anastopoulos, A. D., Guevremont, D. C., Shelton, T. L., & DuPaul, G. J. (1992). Parenting stress among families of children with attention deficit hyperactivity disorder. Journal of Abnormal Child Psychology, 20(5), 503-20.

Andrews-Casal, M., Johnston, D., Fletcher, J., Mulliken, J. B., Stal, S., & Hecht, J. T. (1998). Cleft lip with or without cleft palate: effect of family history on reproductive planning, surgical timing, and parental stress. Cleft Palate Craniofacial Journal, 35(1), 52-7.

Antshel, K. M., & Joseph, G. (2006). Maternal Stress in Nonverbal Learning Disorder: A Comparison With Reading Disorder. Journal of Learning Disabilities, 39(3), 194. doi: Article.

Archer, L. A., & Szatmari, P. (1990). Assessment and Treatment of Food Aversion in a 4-Year-Old Boy - A Multidimensional Approach. Canadian Journal of Psychiatry, 35(6), 501-505.

Barkley, R. A., Fischer, M., Edelbrock, C., & Smallish, L. (1991). The adolescent outcome of hyperactive children diagnosed by research. Journal of Child Psychology & Psychiatry & Allied Disciplines, 32(2), 233-255.

Barnett, D. W., Hall, J. D., & Bramlett, R. K. (1990). Family factors in preschool assessment and intervention: A validity study of parenting stress and coping measures. Journal of School Psychology, 28(1), 13-20.

Bartholomew, L. K., Czyzewski, D. I., Parcel, G. S., Swank, P. R., Sockrider, M. M., Mariotto, M. J., Schidlow, D. V., Fink, R. J., & Seilheimer, D. K. (1997). Self-management of cystic fibrosis: short-term outcomes of the cystic fibrosis family education program. Health Education & Behavior, 24(5), 652-66.

Beckman, P. J. (1991). Comparison of mothers' and fathers' perceptions of the effect of young children with and without disabilities. American Journal on Mental Retardation, 95(5), 585-595.

Beg, M. R., Casey, J. E., & Saunders, C. D. (2007). A typology of behavior problems in preschool children. Assessment, 14(2), 111-128.

Bendell, D., Stone, W. L., Field, T. M., & Goldstein, S. (1989). Children's effects on parenting stress in a low income, minority population. Topics in Early Childhood Special Education, 8(4), 58-71.

Bhavnagri, N. P. (1999). Low income African American mothers' parenting stress and instructional strategies to promote peer relationships in preschool children. Early Education & Development, 10(4), 551-571.

Bigras, M., & Lafreniere, P. J. (1994). Influence of Psychosocial Risk, Marital Conflicts and Parental Stress on the Quality of Mother-Son and Mother-Daughter Interactions. Canadian Journal of Behavioural Science, 26(2), 280-297.

Bigras, M., Lafreniere, P., & Dumas, J. (1996). Discriminant validity of the parent and child scales of the parenting stress index. Early Education & Development, 7(2), 167-178.

Blakeney, P., Meyer, W., 3rd, Robert, R., Desai, M., Wolf, S., & Herndon, D. (1998). Long-term psychosocial adaptation of children who survive burns involving 80% or greater total body surface area. Journal of Trauma, 44(4), 625-32.

Briggs-Gowan, M. J., & Carter, A. S. (1998). Preliminary Acceptability and Psychometrics of the Infant-Toddler Social and Emotional Assessment (ITSEA) - A New Adult-Report Questionnaire. Infant Mental Health Journal, 19(4), 422-445.

Brotman, L. M., Klein, R. G., Kamboukos, D., Brown, E. J., Coard, S. I., & Sosinsky, L. S. (2003). Preventive intervention for urban, low-income preschoolers at familial risk for conduct problems: A randomized pilot study. Journal of Clinical Child and Adolescent Psychology, 32(2), 246–257.

Calzada, E. J., Eyberg, S. M., Rich, B., & Querido, J. G. (2004). Parenting disruptive preschoolers: Experiences of mothers and fathers. Journal of Abnormal Child Psychology, 32(2), 203–213.

Cameron, S. J., & Orr, R. (1989). Stress in families of school-aged children with delayed mental development. Canadian Journal of Rehabilitation, 2(3), 137-144.

Cameron, S., Dobson, L., & Day, D. (1991). Stress in parents of developmentally delayed and non-delayed preschool children. Canada's Mental Health, 39(1), 13-17.

Campbell, S. B., Pierce, E. W., Moore, G., Marakovitz, S., & Newby, K. (1996). Boys Externalizing Problems at Elementary-School Age - Pathways from Early Behavior Problems, Maternal Control, and Family Stress. Development and Psychopathology, 8(4), 701-719.

Capage, L. C., Bennett, G., & McNeil, C. B. (2001). A comparison between African American and Caucasian children referred for treatment of disruptive behavior disorders. Child & Family Behavior Therapy, 23(1), 1-14.

Caulfield, M. B., Fischel, J. E., DeBaryshe, B. D., & Whitehurst, G. J. (1989). Behavioral correlates of developmental expressive language disorder. Journal of Abnormal Child Psychology, 17(2), 187-201.

Chaffee, C. A., Cunningham, C. E., & Secord, G. (1991). The influence of parenting stress and child behavior problems on parental. Journal of Abnormal Child Psychology, 19(1), 65-74.

Chan, Y. C. (1994). Parenting stress and social support of mothers who physically abuse their children in Hong Kong. Child Abuse & Neglect, 18(3), 261-9.

Chazan-Cohen, R., Ayoub, C., Pan, B. A., Roggman, L., Raikes, H., Mckelvey, L., et al. (2007). It takes time: Impacts of Early Head Start that lead to reductions in maternal depression two years later. Infant Mental Health Journal, 28(2), 151-170. doi: 10.1002/imhj.20127.

Chisholm, K. (1998). A 3 Year Follow-Up of Attachment and Indiscriminate Friendliness in Children Adopted from Romanian Orphanages. Child Development, 69(4), 1092-1106.

Conners, N. A., Edwards, M. C., & S. Grant, A. (2007). An Evaluation of a Parenting Class Curriculum for Parents of Young Children: Parenting the Strong-Willed Child. Journal of Child & Family Studies, 16(3), 321-330. doi: 10.1007/s10826-006-9088-z.

Crandell, L. E., Fitzgerald, H. E., & Whipple, E. E. (1997). Dyadic Synchrony in Parent-Child Interactions - A Link with Maternal Representations of Attachment Relationships. Infant Mental Health Journal, 18(3), 247-264.

Cuccaro, M. L., Holmes, G. R., & Wright, H. H. (1993). Behavior problems in preschool children: A pilot study. Psychological Reports, 72(1), 121-122.

Danforth, J. S. (1998). The Outcome of Parent Training Using the Behavior Management Flow-Chart with Mothers and Their Children with Oppositional Defiant Disorder and Attention-Deficit Hyperactivity Disorder. Behavior Modification, 22(4), 443-473.

Darke, P. R., & Goldberg, S. (1994). Father-Infant interaction and parent stress with healthy and medically compromised infants. Infant Behavior & Development, 17(1), 3-14.

Deater-Deckard, K., & Scarr, S. (1996). Parenting Stress Among Dual-Earner Mothers and Fathers - Are There Gender Differences. Journal of Family Psychology, 10(1), 45-59.

DeGangi, G. A., Sickel, R. Z., Kaplan, E. P., & Wiener, A. S. (1997). Mother-infant interactions in infants with disorders of self-regulation. Physical & Occupational Therapy in Pediatrics, 17(1), 17-44.

DeGangi, G. A., Sickel, R. Z., Wiener, A. S., & Kaplan, E. P. (1996). Fussy babies: to treat or not to treat? British Journal of Occupational Therapy, 59(10), 457-64.

DeMaso, D. R., Campis, L. K., Wypij, D., Bertram, S., Lipshitz, M., & Freed, M. (1991). The impact of maternal perceptions and medical severity on the adjustment of children with congenital heart disease. Journal of Pediatric Psychology, 16(2), 137-49.

Donenberg, G., & Baker, B. L. (1993). The Impact of Young-Children with Externalizing Behaviors on Their Families. Journal of Abnormal Child Psychology, 21(2), 179-198.

Doussard-Roosevelt, J. A., Porges, S. W., Scanlon, J. W., Alemi, B., & Scanlon, K. B. (1997). Vagal regulation of heart rate in the prediction of developmental outcome for very low birth weight preterm infants. Child Development, 68(2), 173-86.

Drummond, J., Kysela, G. M., McDonald, L., Alexander, J., & Fleming, D. (1996). Risk and resiliency in two samples of Canadian families. Health & Canadian Society, 4(1), 117-51.

Eddy, M. E., Carter, B. D., Kronenberger, W. G., Conradsen, S., Eid, N. S., Bourland, S. L., & Adams, G. (1998). Parent relationships and compliance in cystic fibrosis. Journal of Pediatric Health Care, 12(4), 196-202.

Esdaile, S. A. (1996). A play-focused intervention involving mothers of preschoolers. American Journal of Occupational Therapy, 50(2), 113-23.

Esdaile, S. A., & Greenwood, K. M. (1995). A survey of mothers' relationships with their preschoolers. Occupational Therapy International, 2(3), 204-19.

Esdaile, S. E., & Greenwood, K. M. (1995). Issues of parenting stress: A study involving mothers of toddlers. Journal of Family Studies, 1(2), 153-165.

Ethier, L. S. (1992). Developmental factors related to stress in neglectful and abusive mothers. Apprentissage et Socialisation, 15(3), 222-236.

Ethier, L. S., & Lafreniere, P. J. (1993). The relationship between maternal stress and preschool children's aggressiveness in single-parent families. International Journal of Psychology, 28(3), 273-289.

Ethier, L. S., Lacharite, C., & Couture, G. (1995). Childhood adversity, parental stress, and depression of negligent mothers. Child Abuse & Neglect, 19(5), 619-32.

Evangelou, M., Brooks, G., & Smith, S. (2007). The Birth to School Study: evidence on the effectiveness of PEEP, an early intervention for children at risk of educational under-achievement. Oxford Review of Education, 33(5), 581-609. doi: 10.1080/03054980701476477.

Eyberg, S. M. B. S. R. A. J. (1995). Parent-child interaction therapy: A psychosocial model for the treatment of young children with conduct problem behavior and their families. Psychopharmacology Bulletin, 31(1), 83-91.

Eyberg, S. M., Boggs, S. R., & Rodriguez, C. M. (1992). Relationships between maternal parenting stress and child disruptive behavior. Child & Family Behavior Therapy, 14(4), 1-9.

Farel, A. M., & Hooper, S. R. (1998). Relationship between the Maternal Social Support Index and the Parenting Stress Index in mothers of very-low-birthweight children now age 7. Psychological Reports, 83(1), 173-4.

Fischel, J. E., Whitehurst, G. J., Caulfield, M. B., & DeBaryshe, B. (1989). Language growth in children with expressive language delay. Pediatrics, 83(2), 218-27.

Fitzgerald, M., Butler, B., & Kinsella, A. (1990). The burden on a family having a child with special needs. Irish Journal of Psychological Medicine, 7(2), 109-113.

Forgays, D. K. (1992). Type A behavior and parenting stress in mothers with young children. Current Psychology: Research & Reviews, 11(1), 3-19.

Forgays, D. K., Hasazi, J. E., & Wasserman, R. C. (1992). Recurrent otitis media and parenting stress in mothers of two-year-old. Journal of Developmental & Behavioral Pediatrics, 13(5), 321-325.

Frankel, K. K., & Harmon, R. J. (1996). Depressed Mothers - They Dont Always Look as Bad as They Feel. Journal of the American Academy of Child and Adolescent Psychiatry, 35(3), 289-298.

Freeman, N. L. P. A. (1991). Child behaviours as stressors: Replicating and extending the use of the CARS. Journal of Child Psychology & Psychiatry & Allied Disciplines, 32(6), 1025-1030.

Girolametto, L., & Tannock, R. (1994). Correlates of Directiveness in the Interactions of Fathers and Mothers of Children with Developmental Delays. Journal of Speech and Hearing Research, 37(5), 1178-1191.

Goldberg, S., Janus, M., Washington, J., Simmons, R. J., Maclusky, I., & Fowler, R. S. (1997). Prediction of Preschool Behavioral-Problems in Healthy and Pediatric Samples. Journal of Developmental and Behavioral Pediatrics, 18(5), 304-313.

Gonchar, N. (1995). College-student mothers and on-site child care: luxury or necessity? Social Work in Education, 17(4), 226-34.

Gottlieb, L. N., & Feeley, N. (1996). The McGill Model of Nursing and children with a chronic condition: "who benefits, and why?". Canadian Journal of Nursing Research, 28(3), 29-48.

Gross, D., Fogg, L., & Tucker, S. (1995). The Efficacy of Parent Training for Promoting Positive Parent Toddler Relationships. Research in Nursing & Health, 18(6), 489-499.

Grossman, J., & Shigaki, I. S. (1994). Investigation of Familial and School-Based Risk-Factors for Hispanic Head-Start Children. American Journal of Orthopsychiatry, 64(3), 456-467.

Guralnick, M. J., Hammond, M. A., Connor, R. T., & Neville, B. (2006). Stability, change, and correlates of the peer relationships of young children with mild developmental delays. Child development, 77(2), 312–324.

Hadadian, A., & Merbler, J. (1996). Mother’s stress: Implications for attachment relationships. Early Child Development & Care, 125, 59-66.

Hall, J. D., & Barnett, D. W. (1991). Classification of risk status in preschool screening: A comparison of alternative measures. Journal of Psychoeducational Assessment, 9(2), 152-159.

Hall, S., Bobrow, M., & Marteau, T. M. (1997). Parents attributions of blame for the birth of a child with Down syndrome: a pilot study. Psychology & Health, 12(4), 579-87.

Hanson, M. J., & Hanline, M. F. (1990). Parenting a child with a disability: A longitudinal study of parental stress and adaptation. Journal of Early Intervention, 14(3), 234-248.

Holaday, B., Turner-Henson, A., Kanematsu, Y., Krulik, T., & Wang, R. (1997). Stress in mothers of chronically ill children: a cross cultural study. Australian Paediatric Nurse, 6(1), 2-9.

Holden, E. W., & Banez, G. A. (1996). Child abuse potential and parenting stress within maltreating families. Journal of Family Violence, 11(1), 1-12.

Hooper, S. R., Burchinal, M. R., Roberts, J. E., Zeisel, S., & Neebe, E. C. (1998). Social and Family Risk-Factors for Infant Development at One-Year - An Application of the Cumulative Risk Model. Journal of Applied Developmental Psychology, 19(1), 85-96.

Horsch, U., Weber, C., Bertram, B., & Detrois, P. (1997). Stress experienced by parents of children with cochlear implants compared with parents of deaf children and hearing children. American Journal of Otology, 18(6 Suppl), 161-163.

Horwitz, S. M., Briggs-Gowan, M. J., Storfer-Isser, A., & Carter, A. S. (2009). Persistence of Maternal Depressive Symptoms throughout the Early Years of Childhood. Journal of Women's Health, 18(5), 637-645. doi: 10.1089/jwh.2008.1229.

Hutcheson, J. J., & Black, M. M. (1996). Psychometric properties of the Parenting Stress Index in a sample of low-income African-American mothers of infants and toddlers. Early Education & Development, 7(4), 381-400.

Hutchings, J., Bywater, T., Daley, D., Gardner, F., Whitaker, C., Jones, K.,... Edwards, R. T. (2007). Parenting intervention in Sure Start services for children at risk of developing conduct disorder: pragmatic randomized controlled trial. British Medical Journal, doi:10.1136/bmj.39126.620799.55.

Innocenti, M. S., Huh, K., & Boyce, G. C. (1992). Families of children with disabilities: Normative data and other considerations on parenting stress. Topics in Early Childhood Special Education, 12(3), 403-427.

Kazak, A. E., Penati, B., Waibel, M. K., & Blackall, G. F. (1996). The Perception of Procedures Questionnaire: psychometric properties of a brief parent report measure of procedural distress. Journal of Pediatric Psychology, 21(2), 195-207.

Kazdin, A. E. (1994). Family adversity, socioeconomic disadvantage, and parental stress: Contextual variables related to premature termination from child behavior therapy. Psicologia Conductual, 2(1), 5-21.

Kazui, M., Muto, T., & Sonoda, N. (1996). The roles of marital quality and parenting stress in mother-preschooler relationships. (Japanese). Japanese Journal of Developmental Psychology, 7(1), 31-40.

Kelley, S. J. (1992). Parenting stress and child maltreatment in drug-exposed children. Child Abuse & Neglect, 16(3), 317-28.

Kelley, S. J. (1993). Caregiver stress in grandparents raising grandchildren. Image the Journal of Nursing Scholarship, 25(4), 331-7.

Kelley, S. J. (1998). Stress and coping behaviors of substance-abusing mothers. Journal of the Society of Pediatric Nurses, 3(3), 103-10.

Klee, L., Kronstadt, D., & Zlotnick, C. (1997). Foster care’s youngest: A preliminary report. American Journal of Orthopsychiatry, 67(2), 290-299.

Kobe, F. H., & Hammer, D. (1994). Parenting stress and depression in children with mental retardation and developmental disabilities. Research in Developmental Disabilities, 15(3), 209-21.

Lacharite, C., Ethier, L., & Piche, C. (1992). Parental stress in mothers of preschool children: validation and Quebec norms for the Parental Stress Inventory. Sante Mentale au Quebec, 17(2), 183-203.

LaFiosca, T., & Loyd, B. H. (1986). Defensiveness and the assessment of parental stress and anxiety. Journal of Clinical Child Psychology, 15(3), 254-259.

Leung, S. S. L., Leung, C., & Chan, R. (2007). Perceived child behaviour problems, parenting stress, and marital satisfaction: comparison of new arrival and local parents of preschool children in Hong Kong. Hong Kong Medical Journal / Xianggang Yi Xue Za Zhi / Hong Kong Academy of Medicine, 13(5), 364-371.

Levin, R., & Banks, S. (1991). Stress in parents of children with epilepsy. Canadian Journal of Rehabilitation, 4(4), 229-38.

Little, M., Murphy, J. M., Jellinek, M. S., Bishop, S. J., & Arnett, H. L. (1994). Screening 4-Year-Old and 5-Year-Old Children for Psychosocial Dysfunction - A Preliminary-Study with the Pediatric Symptom Checklist. Journal of Developmental and Behavioral Pediatrics, 15(3), 191-197.

Lobato, D. (1985). Preschool siblings of handicapped children: Impact of peer support and training. Journal of Autism & Developmental Disorders, 15(3), 345-350.

Mainemer, H., Gilman, L. C., & Ames, E. W. (1998). Parenting stress in families adopting children from Romanian orphanages. Journal of Family Issues, 19(2), 164-180.

Manassis, K., Bradley, S., Goldberg, S., Hood, J., & Swinson, R. P. (1994). Attachment in Mothers with Anxiety Disorders and Their Children. Journal of the American Academy of Child and Adolescent Psychiatry, 33(8), 1106-1113.

Manassis, K., Bradley, S., Goldberg, S., Hood, J., & Swinson, R. P. (1995). Behavioral-Inhibition, Attachment and Anxiety in Children of Mothers with Anxiety Disorders. Canadian Journal of Psychiatry, 40(2), 87-92.

McBride, B. A. (1991). Parental support programs and paternal stress: An exploratory study. Early Childhood Research Quarterly, 6(2), 137-140.

McKay, J. M., Pickens, J., & Stewart, A. L. (1996). Inventoried and observed stress in parent-child interactions. Current Psychology: Developmental, Learning, Personality, Social, 15(3), 223-234.

McKelvey, L. M., Whiteside-Mansell, L., Faldowski, R. A., Shears, J., Ayoub, C., & Hart, A. D. (2009). Validity of the short form of the parenting stress index for fathers of toddlers. Journal of Child and Family Studies, 18(1), 102-111.

Milner, J. S., & Crouch, J. L. (1997). Impact and detection of response distortions on parenting measures used to assess risk for child physical abuse. Journal of Personality Assessment, 69(3), 633-50.

Nievar, M. A, Jacobson, A., & Dier, S. (2008, November). Home visiting for at-risk preschoolers: A successful model for Latino families. Paper presented at the Annual Meeting of the National Council on Family Relations, Little Rock, Arkansas.

Nixon, R. D. V. (2001). Changes in hyperactivity and temperament in behaviourally disturbed preschoolers after Parent-Child Interaction Therapy (PCIT). Behaviour Change, 18 (3), 168-176.

Nixon, R. D., Sweeney, L., Erickson, D. B., & Touyz, S. W. (2003). Parent-child interaction therapy: A comparison of standard and abbreviated treatments for oppositional defiant preschoolers. Journal of Consulting and Clinical Psychology, 71(2), 251–260.

Noel, M., Peterson, C., & Jesso, B. (2008). The relationship of parenting stress and child temperament to language development among economically disadvantaged preschoolers. Journal of Child Language, 35(4), 823-843. doi: 10.1017/S0305000908008805.

Oelofsen, N., & Richardson, P. (2006). Sense of coherence and parenting stress in mothers and fathers of preschool children with developmental disability. Journal of Intellectual & Developmental Disability, 31(1), 1-12. doi: 10.1080/13668250500349367.

Ong, L. C., Afifah, I., Sofiah, A., & Lye, M. S. (1998). Parenting stress among mothers of Malaysian children with cerebral palsy: predictors of child- and parent-related stress. Annals of Tropical Paediatrics, 18(4), 301-7.

Onufrak, B., Saylor, C. F., Taylor, M. J., Eyberg, S. M., & Boyce, G. C. (1995). Determinants of responsiveness in mothers of children with intraventricular hemorrhage. Journal of Pediatric Psychology, 20(5), 587-99.

Orr, R. R., Cameron, S. J., Dobson, L. A., & Day, D. M. (1993). Age-related changes in stress experienced by families with a child who has developmental delays. Mental Retardation, 31(3), 171-6.

Ostberg, M. (1998). Parental stress, psychosocial problems and responsiveness in help-seeking parents with small (2-45 months old) children. Acta Paediatrica, 87(1), 69-76.

Ostberg, M., Hagekull, B., & Wettergren, S. (1997). A measure of parental stress in mothers with small children: dimensionality, stability and validity. Scandinavian Journal of Psychology, 38(3), 199-208.

Paradise, J. L., Feldman, H. M., Colborn, D. K., Campbell, T. F., Dollaghan, C. A., Rockette, H. E., Janosky, J. E., Kurs-Lasky, M., Bernard, B. S., & Smith, C. G. (1999). Parental stress and parent-rated child behavior in relation to otitis media in the first three years of life. Pediatrics, 104(6), 1264-73.

Perrott, S. B., & Taylor, H. G. (1991). Neuropsychological sequelae, familial stress, and environmental adaptation. Developmental Neuropsychology, 7(1), 69-86.

Perry, A., Sarlo-McGarvey, N., & Factor, D. C. (1992). Stress and family functioning in parents of girls with Rett syndrome. Journal of Autism & Developmental Disorders, 22(2), 235-248.

Powers, S. W., Byars, K. C., Mitchell, M. J., Patton, S. R., Standiford, D. A., & Dolan, L. M. (2002). Parent report of mealtime behavior and parenting stress in young children with type 1 diabetes and in healthy control subjects. Diabetes Care, 25(2), 313-318.

Quinn, M., Carr, A., Carroll, L., & O Sullivan, D. (2006). An evaluation of the Parents Plus Programme for pre-school children with conduct problems: A comparison of those with and without developmental disabilities. Irish Journal of Psychology, 27(3/4), 168-182.

Quinn, M., Carr, A., Carroll, L., & O'Sullivan, D. (2007). Parents Plus Programme 1: Evaluation of its effectivesness for pre-school children with developmental disabilities and behavioral problems. Journal of Applied Research in Intellectual Disabilities, 20(4), 345-359.

Raver, C. C., Gershoff, E. T., & Aber, J. L. (2007). Testing Equivalence of Mediating Models of Income, Parenting, and School Readiness for White, Black, and Hispanic Children in a National Sample. Child Development, 78(1), 96-115. doi: 10.1111/j.1467-8624.2007.00987.x.

Reid, M. J., Walter, A. L., & Oleary, S. G. (1999). Treatment of Young Childrens Bedtime Refusal and Nighttime Wakings - A Comparison of Standard and Graduated Ignoring Procedures. Journal of Abnormal Child Psychology, 27(1), 5-16.

Robbins, F. R., & Dunlap, G. (1992). Effects of task difficulty on parent teaching skills and behavior problems of young children with autism. American Journal on Mental Retardation, 96(6), 631-643.

Robbins, F. R., Dunlap, G., & Plienis, A. J. (1991). Family characteristics, family training, and the progress of young children with autism. Journal of Early Intervention, 15(2), 173-184.

Robson, A. L. (1997). Low-Birth-Weight and Parenting Stress During Early-Childhood. Journal of Pediatric Psychology, 22(3), 297-311.

Sadeh, A., Lavie, P., & Scher, A. (1994). Sleep and temperament: Maternal perceptions of temperament of sleep-disturbed toddlers. Early Education & Development, 5(4), 311-322.

Sarimski, K. (1997). Behavioural phenotypes and family stress in three mental retardation syndromes. European Child & Adolescent Psychiatry, 6(1), 26-31.

Scheel, M. J., & Rieckmann, T. (1998). An empirically derived description of self-efficacy and empowerment for parents of children identified as psychologically disordered. American Journal of Family Therapy, 26(1), 15-27.

Sheeran, T., Marvin, R. S., & Pianta, R. C. (1997). Mothers' resolution of their child's diagnosis and self-reported measures of parenting stress, marital relations, and social support. Journal of Pediatric Psychology, 22(2), 197-212.

Shelton, T. L., Barkley, R. A., Crosswait, C., Moorehouse, M., Fletcher, K., Barrett, S., Jenkins, L., & Metevia, L. (1998). Psychiatric and Psychological Morbidity as a Function of Adaptive Disability in Preschool-Children with Aggressive and Hyperactive-Impulsive-Inattentive Behavior. Journal of Abnormal Child Psychology, 26(6), 475-494.

Silovsky, J. F., Niec, L., Bard, D., & Hecht, D. B. (2007). Treatment for Preschool Children With Interpersonal Sexual Behavior Problems: A Pilot Study. Journal of Clinical Child & Adolescent Psychology, 36(3), 378-391. doi: 10.1080/15374410701444330.

Singer, L. T., Song, L.-y., Hill, B. P., & Jaffe, A. C. (1990). Stress and depression in mothers of failure-to-thrive children. Journal of Pediatric Psychology, 15(6), 711-720.

Sperry, L. A., & Symons, F. J. (2003). Maternal judgments of intentionality in young children with autism: the effects of diagnostic information and stereotyped behavior. Journal of Autism and Developmental Disorders, 33(3), 281–287.

Stapleton, S. R., Drummond, J., Kysela, G. M., McDonald, L., Alexander, J., & Fleming, D. (1996). Team-building: making collaborative practice work. Risk and resiliency in two samples of Canadian families. Journal of Nurse-Midwifery, 4(1), 117-51.

Tannock, R., Girolametto, L., & Siegel, L. S. (1992). Language intervention with children who have developmental delays: effects of an interactive approach. American Journal of Mental Retardation, 97(2), 145-60.

Tarbell, S. E., & Kosmach, B. (1998). Parental psychosocial outcomes in pediatric liver and/or intestinal transplantation: pretransplantation and the early postoperative period. Liver Transplantation & Surgery, 4(5), 378-87.

Teti, D. M., Nakagawa, M., Das, R., & Wirth, O. (1991). Security of attachment between preschoolers and their mothers: Relations among social interaction, parenting stress, and mother's sorts of the Attachment Q-Set. Developmental Psychology, 27(3), 440-447.

Thome, M., & Skuladottir, A. (2005). Changes in sleep problems, parents distress and impact of sleep problems from infancy to preschool age for referred and unreferred children. Scandinavian Journal of Caring Sciences, 19(2), 86-94. doi: 10.1111/j.1471-6712.2005.00322.x.

Tsang, S., Tsang, S., Chan, E., & Lee, C. (1992). Stress of parents with normal and special pre-school children: A comparison study. Bulletin of the Hong Kong Psychological Society, 28, 63-79.

Tucker, S., Gross, D., Fogg, L., Delaney, K., & Lapporte, R. (1998). The long-term efficacy of a behavioral parent training intervention for families with 2-year-olds. Research in Nursing & Health, 21(3), 199-210.

Tynan, W. D., & Nearing, J. (1994). The diagnosis of attention deficit hyperactivity disorder in young children. Infants & Young Children, 6(4), 13-20.

Warfield, M. E., Krauss, M. W., Hauser-Cram, P., Upshur, C. C., & Shonkoff, J. P. (1999). Adaptation during early childhood among mothers of children with disabilities. Journal of Developmental & Behavioral Pediatrics, 20(1), 9-16.

Webster-Stratton, C. (1988). Mothers' and fathers' perceptions of child deviance: Roles of parent and child behaviors and parent adjustment. Journal of Consulting & Clinical Psychology, 56(6), 909-915.

Wells, R. D., & Schwebel, A. I. (1987). Chronically ill children and their mothers: Predictors of resilience and vulnerability to hospitalization and surgical stress. Journal of Developmental & Behavioral Pediatrics, 8(2), 83-89.

Winton, M. A. (1990). An evaluation of a support group for parents who have a sexually abused child. Child Abuse & Neglect, 14(3), 397-405.

Wolf, L. C., & Fisman, N. S. (1989). Psychological effects of parenting stress on parents of autistic children. Journal of Autism & Developmental Disorders, 19(1), 157-166.

Woolfson, L., & Grant, E. (2006). Authoritative parenting and parental stress in parents of pre-school and older children with developmental disabilities. Child: Care, Health & Development, 32(2), 177-184.

Wysocki, T., Huxtable, K., Linscheid, T. R., & Wayne, W. (1989). Adjustment to diabetes mellitus in preschoolers and their mothers. Diabetes Care, 12(8), 524-9.

Youngblut, J. M., & Brooten, D. (2006). Pediatric head trauma: parent, parent-child, and family functioning 2 weeks after hospital discharge. Journal of Pediatric Psychology, 31(6), 608-618.

Youngblut, J. M., Singer, L. T., Madigan, E. A., Swegart, L. A., & Rodgers, W. L. (1998). Maternal employment and parent-child relationships in single-parent families of low-birth-weight preschoolers. Nursing Research, 47(2), 114-21.

Elementary School

Bramlett, R. K., Rowell, R. K., & Mandenberg, K. (2000). Predicting first grade achievement from kindergarten screening measures: A comparison of child and family predictors. Research in the Schools, 7(1), 1-9.

Burchinal, M., Roberts, J. E., Zeisel, S. A., Hennon, E. A., & Hooper, S. (2006). Social risk and protective child, parenting, and child care factors in early elementary school years. Parenting: Science and Practice, 6(1), 79. doi: 10.1207/s15327922par0601_4

DeCaro, J. A., & Worthman, C. M. (2008). Return to school accompanied by changing associations between family ecology and cortisol. Developmental Psychobiology, 50(2), 183–195.

Edens, J. F., Cavell, T. A., & Hughes, J. N. (1999). The Self-Systems of Aggressive-Children - A Cluster-Analytic Investigation. Journal of Child Psychology and Psychiatry and Allied Disciplines, 40(3), 441-453.

Fuller, G. B., & Rankin, R. E. (1994). Differences in levels of parental stress among mothers of learning disabled, emotionally impaired, and regular school children. Perceptual & Motor Skills, 78(2), 583-92.

Gershoff, E. T., Raver, C. C., Aber, J. L., & Lennon, M. C. (2007). Income Is Not Enough: Incorporating Material Hardship Into Models of Income Associations With Parenting and Child Development. Child Development, 78(1), 70-95. doi: 10.1111/j.1467-8624.2007.00986.x.

Geva, R., Yosipof, R., Eschel, R., Leitner, Y., Valevski, A. F., & Harel, S. (2009). Readiness and Adjustments to School for Children With Intrauterine Growth Restriction (IUGR): An Extreme Test Case Paradigm. Exceptional Children, 75(2), 211-230.

Heller, T. L., Baker, B. L., Henker, B., & Hinshaw, S. P. (1996). Externalizing Behavior and Cognitive-Functioning from Preschool to First-Grade - Stability and Predictors. Journal of Clinical Child Psychology, 25(4), 376-387.

Hughes, J. N., Cavell, T. A., & Grossman, P. B. (1997). A Positive View of Self - Risk or Protection for Aggressive-Children. Development and Psychopathology, 9(1), 75-94.

Huth-Bocks, A., & Hughes, H. (2008). Parenting Stress, Parenting Behavior, and Children’s Adjustment in Families Experiencing Intimate Partner Violence. Journal of Family Violence, 23(4), 243-251. doi: 10.1007/s10896-007-9148-1.

Meisels, S. J., & Liaw, F. R. (1993). Failure in Grade - Do Retained Students Catch-Up. Journal of Educational Research, 87(2), 69-77.

Messer, S. C., & Gross, A. M. (1995). Childhood Depression and Family-Interaction - A Naturalistic Observation Study. Journal of Clinical Child Psychology, 24(1), 77-88.

Nachshen, J. S., & Minnes, P. (2005). Empowerment in parents of school-aged children with and without developmental disabilities. Journal of Intellectual Disability Research, 49(12), 889–904.

Ratner, H. H., Chiodo, L., Covington, C., Sokol, R. J., Ager, J., & Delaney, V. (2006). Violence Exposure, IQ, Academic Performance, and Children's Perception of Safety: Evidence of Protective Effects. Merrill-Palmer Quarterly, 52(2), 264-287. doi: Article.

Southard, N. A., & May, D. C. (1996). The Effects of Pre-First-Grade Programs on Student Reading and Mathematics Achievement. Psychology in the Schools, 33(2), 132-142.

Taylor, H. G., Klein, N., Schatschneider, C., & Hack, M. (1998). Predictors of Early School-Age Outcomes in Very-Low-Birth-Weight Children. Journal of Developmental and Behavioral Pediatrics, 19(4), 235-243.

Woolfson, L., & Grant, E. (2006). Authoritative parenting and parental stress in parents of pre-school and older children with developmental disabilities. Child: Care, Health & Development, 32(2), 177-184.

Social Support

Adamakos, H., Kathleen, R., G., U. D., & John, P. (1986). Maternal social support as a predictor of mother-child stress and stimulation. Child Abuse & Neglect, 10(4), 463-470.

Ando, J., Nonaka, K., Ozaki, K., Sato, N., Fujisawa, K. K., Suzuki, K., et al. (2006). The Tokyo Twin Cohort Project: overview and initial findings. Twin Research and Human Genetics: The Official Journal of the International Society for Twin Studies, 9(6), 817-826. doi: 10.1375/183242706779462480.

Bauman, L. J., Camacho, S., Westbrook, L., & Forbes-Jones, E. (1997). Correlates of personal stigma and social stigma among mothers with HIV/AIDS : National Conference of Women With HIV.

Beckman, P. J. (1991). Comparison of mothers' and fathers' perceptions of the effect of young children with and without disabilities. American Journal on Mental Retardation, 95(5), 585-595.

Benzies, K. M., Harrison, M., & Magill-Evans, J. (2004). Parenting stress, marital quality, and child behaviour problems at age 7 years. Public Health Nursing, 21(2), 111-121.

Black, M. M., Nair, P., Kight, C., Wachtel, R., Roby, P., & Schuler, M. (1994). Parenting and early development among children of drug-abusing women: effects of home intervention. Pediatrics, 94(4 Pt 1), 440-8.

Bos, H. M., van Balen, F., & van den Boom, D. C. (2004). Experience of parenthood, couple relationship, social support, and child-rearing goals in planned lesbian mother families. Journal of Child Psychology and Psychiatry, 45(4), 755–764.

Burrell, B., Thompson, B., & Sexton, D. (1995). Measurement Characteristics of the Perceived Adequacy of Resources Scale. Educational & Psychological Measurement, 55(2), 249-257.

Button, S., Pianta, R. C., & Marvin, R. S. (2001). Partner support and maternal stress in families raising young children with cerebral palsy. Journal of Developmental & Physical Disabilities, 13(1), 61-81.

Campis, L. B., DeMaso, D. R., & Twente, A. W. (1995). The role of maternal factors in the adaptation of children with craniofacial disfigurement. Cleft Palate Craniofacial Journal, 32(1), 55-61.

Carothers, S. S., Borkowski, J. G., & Whitman, T. L. (2006). Children of Adolescent Mothers: Exposure to Negative Life Events and the Role of Social Supports on Their Socioemotional Adjustment. Journal of Youth and Adolescence, 35(5), 822-832. doi: 10.1007/s10964-006-9096-8.

Chan, Y. C. (1994). Parenting stress and social support of mothers who physically abuse their children in Hong Kong. Child Abuse & Neglect, 18(3), 261-9.

Clark, R., Hyde, J. S., Essex, M. J., & Klein, M. H. (1997). Length of Maternity Leave and Quality of Mother-Infant Interactions. Child Development, 68(2), 364-383.

Coleman, P. K., & Karraker, K. H. (1998). Self-Efficacy and Parenting Quality - Findings and Future Applications. Developmental Review, 18(1), 47-85.

Colpin, H., DeMunter, A., Nys, K., & Vandemuelebroecke, L. (2000). Pre- and postnatal determinants of parenting stress in mothers of one-year-old twins. Marriage & Family Review, 30(1-2), 99-107.

Deater-Deckard, K. (1998). Parenting Stress and Child Adjustment - Some Old Hypotheses and New Questions. Clinical Psychology - Science and Practice, 5(3), 314-332.

Drummond, J., McDonald, L., MacKenzie-Keating, S., & Fleming, D. (2004). Types of support accessed by families of young children with disabilities living in Alberta. Developmental Disabilities Bulletin, 32(1), 1-27.

Dumka, L. E., Gonzales, N. A., Wood, J. L., & Formoso, D. (1998). Using Qualitative Methods to Develop Contextually Relevant Measures and Preventive Interventions - An Illustration. American Journal of Community Psychology, 26(4), 605-637.

Dunham, P. J., Hurshman, A., Litwin, E., Gusella, J., Ellsworth, C., & Dodd, P. W. D. (1998). Computer-Mediated Social Support - Single Young Mothers as a Model System. American Journal of Community Psychology, 26(2), 281-306.

Edens, J. F., Cavell, T. A., & Hughes, J. N. (1999). The Self-Systems of Aggressive-Children - A Cluster-Analytic Investigation. Journal of Child Psychology and Psychiatry and Allied Disciplines, 40(3), 441-453.

Emery, J., Paquette, D., & Bigras, M. (2008). Factors predicting attachment patterns in infants of adolescent mothers. Journal of Family Studies, 14(1), 65-90. doi: Article.

Fagan, J., Bernd, E., & Whiteman, V. (2007). Adolescent Fathers' Parenting Stress, Social Support, and Involvement with Infants. Journal of Research on Adolescence (Blackwell Publishing Limited), 17(1), 1-22. doi: 10.1111/j.1532-7795.2007.00510.x.

Farel, A. M., & Hooper, S. R. (1998). Relationship between the Maternal Social Support Index and the Parenting Stress Index in mothers of very-low-birthweight children now age 7. Psychological Reports, 83(1), 173-4.

Feldman, M. A., Varghese, J., Ramsay, J., & Rajska, D. (2002). Relationships between social support, stress and mother-child interactions in mothers with intellectual disabilities. Journal of Applied Research in Intellectual Disabiliites, 15(4), 314-323.

Feldman, R., Eidelman, A. I., & Rotenberg, N. (2004). Parenting Stress, Infant Emotion Regulation, Maternal Sensitivity, and the Cognitive Development of Triplets: A Model for Parent and Child Influences in a Unique Ecology. Child Development, 75(6), 1774-1791. doi: 10.1111/j.1467-8624.2004.00816.x.

Feldman, R., Granat, A., Pariente, C., Kanety, H., Kuint, J., & Gilboa-Schechtman, E. (2009). Maternal Depression and Anxiety Across the Postpartum Year and Infant Social Engagement, Fear Regulation, and Stress Reactivity. Journal of Amer Academy of Child & Adolescent Psychiatry, 48(9), 919 - 927.

Flake, E., Davis, B., Johnson, P., & Middleton, L. (2009). The Psychosocial Effects of Deployment on Military Children, Journal of Developmental & Behavioral Pediatrics, 30, 271-278.

Guralnick, M. J., Hammond, M. A., Neville, B., & Connor, R. T. (2008). The relationship between sources and functions of social support and dimensions of child- and parent-related stress. Journal of Intellectual Disability Research: JIDR, 52(12), 1138-1154. doi: 10.1111/j.1365-2788.2008.01073.x.

Harmer, A. L. M., Sanderson, J., & Mertin, P. (1999). Influence of Negative Childhood Experiences on Psychological Functioning, Social Support, and Parenting for Mothers Recovering from Addiction. Child Abuse & Neglect, 23(5), 421-433.

Harris, H. E., Ellison, G. T. H., & Clement, S. (1999). Relative Importance of Heritable Characteristics and Life-Style in the Development of Maternal Obesity. Journal of Epidemiology and Community Health, 53(2), 66-74.

Hintermair, M. (2000). Hearing impairment, social networks, and coping: The need for families with hearing-impaired children to relate to other parents and to hearing-impaired adults. American Annals of the Deaf, 145(1), 41-53.

Hughes, J. N., Cavell, T. A., & Grossman, P. B. (1997). A Positive View of Self - Risk or Protection for Aggressive-Children. Development and Psychopathology, 9(1), 75-94.

Jackson, A. P., Gyamfi, P., Brooksgunn, J., & Blake, M. (1998). Employment Status, Psychological Well-Being, Social Support, and Physical Discipline Practices of Single Black Mothers. Journal of Marriage and the Family, 60(4), 894-902.

Kazak, A. E., & Marvin, R. S. (1984). Differences, difficulties and adaptation: Stress and social networks in families with a handicapped child. Family Relations: Journal of Applied Family & Child Studies, 33(1), 67-77.

Krauss, M. W., Upshur, C. C., Shonkoff, J. P., & Hausercram, P. (1993). The Impact of Parent Groups on Mothers of Infants with Disabilities. Journal of Early Intervention, 17(1), 8-20.

Lafreniere, P. J., & Capuano, F. (1997). Preventive Intervention as Means of Clarifying Direction of Effects in Socialization - Anxious-Withdrawn Preschoolers Case. Development and Psychopathology, 9(3), 551-564.

Lederberg, A. R., & Golbach, T. (2002). Parenting stress and social support in hearing mothers of deaf and hearing children: a longitudinal study. Journal of Deaf Studies and Deaf Education, 7(4), 330-345.

Manassis, K., Bradley, S., Goldberg, S., Hood, J., & Swinson, R. P. (1994). Attachment in Mothers with Anxiety Disorders and Their Children. Journal of the American Academy of Child and Adolescent Psychiatry, 33(8), 1106-1113.

Manassis, K., Bradley, S., Goldberg, S., Hood, J., & Swinson, R. P. (1995). Behavioral-Inhibition, Attachment and Anxiety in Children of Mothers with Anxiety Disorders. Canadian Journal of Psychiatry, 40(2), 87-92.

Meadow-Orlans, K. P. (1994). Stress, support, and deafness: Perceptions of infants' mothers and fathers. Journal of Early Intervention, 18(1), 91-102.

Meager, I., & Milgrom, J. (1996). Group treatment for postpartum depression: A pilot study. Australian & New Zealand Journal of Psychiatry, 30(6), 852-860.

Miceli, P. J., Goeke-Morey, M. C., Whitman, T. L., Kolberg, K. S., Miller-Loncar, C., & White, R. D. (2000). Brief report: birth status, medical complications, and social environment: individual differences in development of preterm, very low birth weight infants. Journal of Pediatric Psychology, 25(5), 353-8.

Mowbray, C. T., Bybee, D., Hollingsworth, L., Goodkind, S., & Oyserman, D. (2005). Living Arrangements and Social Support: Effects on the Well-Being of Mothers with Mental Illness. Social Work Research, 29(1), 41. doi: Article.

Nachshen, J. S., & Minnes, P. (2005). Empowerment in parents of school-aged children with and without developmental disabilities. Journal of Intellectual Disability Research, 49(12), 889–904.

Nereo, N. E., Fee, R. J., & Hinton, V. J. (2003). Parental stress in mothers of boys with Duchenne muscular dystrophy. Journal of Pediatric Psychology, 28(7), 473-484.

Nitz, K., Ketterlinus, R. D., & Brandt, L. J. (1995). The role of stress, social support, and family environment in adolescent mothers' parenting. Journal of Adolescent Research, 10(3), 358-382.

Passino, A. W., Whitman, T. L., Borkowski, J. G., Schellenbach, C. J., Maxwell, S. E., Keogh, D., & Rellinger, E. (1993). Personal Adjustment During Pregnancy and Adolescent Parenting. Adolescence, 28(109), 97-122.

Planos, R., Zayas, L. H., & Buschrossnagel, N. A. (1997). Mental-Health Factors and Teaching Behaviors Among Low-Income Hispanic Mothers. Families in Society-The Journal of Contemporary Human Services, 78(1), 4-12.

Qin, X., Tang, C., Zhu, S., Liang, Y., & Zou, X. (2009). Parenting stress and related factors in mothers of children with autism. Chinese Mental Health Journal, 23(9), 629-633.

Quittner, A. L., Digirolamo, A. M., Michel, M., & Eigen, H. (1992). Parental Response to Cystic-Fibrosis - A Contextual Analysis of the Diagnosis Phase. Journal of Pediatric Psychology, 17(6), 683-704.

Rhodes, J. E., Ebert, L., & Fischer. (1992). Natural mentors: An overlooked resource in the social networks of young, African-American mothers. American Journal of Community Psychology, 20(4), 445-461.

Richardson, R. A., Barbour, N. E., & Bubenzer, D. L. (1995). Peer relationships as a source of support for adolescent mothers. Journal of Adolescent Research, 10(2), 278-290.

Sabatelli, R. M., & Waldron, R. J. (1995). Measurement Issues in the Assessment of the Experiences of Parenthood. Journal of Marriage and the Family, 57(4), 969-980.

Sheeran, T., Marvin, R. S., & Pianta, R. C. (1997). Mothers' resolution of their child's diagnosis and self-reported measures of parenting stress, marital relations, and social support. Journal of Pediatric Psychology, 22(2), 197-212.

Silver, E. J., Henegham, A. M., Bauman, L. J., & Stein, R. (2006). The relationship of depressive symptoms to parenting competence and social support in inner-city mothers of young children. Maternal & Child Health Journal, 10(1), 105-112.

Singer, L. T., Davillier, M., Bruening, P., Hawkins, S., & Yamashita, T. S. (1996). Social Support, Psychological Distress, and Parenting Strains in Mothers of Very-Low-Birth-Weight Infants. Family Relations, 45(3), 343-350.

Singer, L. T., Salvator, A., Guo, S. Y., Collin, M., Lilien, L., & Baley, J. (1999). Maternal Psychological Distress and Parenting Stress After the Birth of a Very-Low-Birth-Weight Infant. Journal of the American Medical Association, 281(9), 799-805.

Sontag, J. C. (1996). Toward a Comprehensive Theoretical Framework for Disability Research - Bronfenbrenner Revisited. Journal of Special Education, 30(3), 319-344.

Speltz, M. L., Endriga, M. C., Fisher, P. A., & Mason, C. A. (1997). Early Predictors of Attachment in Infants with Cleft-Lip and/or Palate. Child Development, 68(1), 12-25.

Speltz, M. L., Goodell, E. W., Endriga, M. C., & Clarren, S. K. (1994). Feeding Interactions of Infants with Unrepaired Cleft-Lip and or Palate. Infant Behavior & Development, 17(2), 131-139.

Stewart, R. B. (1990). The second child: Family transition and adjustment. Newbury Park, CA: Sage Publications, Inc.

Stoiber, K. C., & Houghton, T. G. (1993). The Relationship of Adolescent Mothers Expectations, Knowledge, and Beliefs to Their Young Childrens Coping Behavior. Infant Mental Health Journal, 14(1), 61-79.

Tarkka, M. T., Paunonen, M., & Laippala, P. (1999). Factors Related to Successful Breast-Feeding by First-Time Mothers When the Child Is 3 Months Old. Journal of Advanced Nursing, 29(1), 113-118.

Taylor, J. A., & Kemper, K. J. (1998). Group well-child care for high-risk families: maternal outcomes. Archives of Pediatrics & Adolescent Medicine, 152(6), 579-84.

Taylor, J. A., Davis, R. L., & Kemper, K. J. (1997). A Randomized Controlled Trial of Group Versus Individual Well Child-Care for High-Risk Children - Maternal-Child Interaction and Developmental Outcomes. Pediatrics, 99(6), E91-E96.

Uno, D., Florsheim, P., & Uchino, B. N. (1998). Psychosocial Mechanisms Underlying Quality of Parenting Among Mexican-American and White Adolescent Mothers. Journal of Youth and Adolescence, 27(5), 585-605.

Van Hasselt, V. B., Hersen, M., Null, J. A., Ammerman, R. T., Bukstein, O. G., Mcgillivray, J., & Hunter, A. (1993). Drug-Abuse Prevention for High-Risk African-American Children and Their Families - A Review and Model Program. Addictive Behaviors, 18(2), 213-234.

Waisbren, S. E., Rones, M., Read, C. Y., Marsden, D., & Levy, H. L. (2004). Brief report: Predictors of parenting stress among parents of children with biochemical genetic disorders. Journal of Pediatric Psychology, 29(7), 565-570.

Wanamaker, C. E., & Glenwick, D. S. (1998). Stress, Coping, and Perceptions of Child-Behavior in Parents of Preschoolers with Cerebral-Palsy. Rehabilitation Psychology, 43(4), 297-312.

Winton, M. A. (1990). An evaluation of a support group for parents who have a sexually abused child. Child Abuse & Neglect, 14(3), 397-405.

Wolf, L. C., & Fisman, N. S. (1989). Psychological effects of parenting stress on parents of autistic children. Journal of Autism & Developmental Disorders, 19(1), 157-166.

Wolf, L. C., Fisman, S., Ellison, D., & Freeman, T. (1998). Effect of Sibling Perception of Differential Parental Treatment in Sibling Dyads with One Disabled-Child. Journal of the American Academy of Child and Adolescent Psychiatry, 37(12), 1317-1325.

Zaidman-Zait, A., Mirena, P., Zumbo, B., Wellington, S., Dua, V., & Kalynchuk, K. (2010). An item response theory analysis of the Parenting Stress Index-Short Form with parents of children with autism spectrum disorders. Journal of Child Psychology and Psychiatry, 51, 1269-1277.

SUBSTANCE ABUSE

Dawe, S., Harnett, P. H., Rendalls, V., & Staiger, P. (2003). Improving family functioning and child outcome in methadone maintained families: the Parents Under Pressure programme. Drug and Alcohol Review, 22(3), 299–307.

Kern, J. K., West, E. Y., Grannemann, B. D., Greer, T., Snell, L. M., Cline, L. L., …Trivedi, M. H. (2004). Reduction in stress and depressive symptoms in mothers of substance-exposed infants, participating in a psychosocial program. Maternal & Child Health Journal, 8(3), 127-136.

Knight, D. K., & Wallace, G. (2003). Where are the children? An examination of children's living arrangements when mothers enter residential drug treatment. Journal of Drug Issues, 33(2), 305. doi: Article.

Luthar, S. S., & Sexton, C. C. (2007). Maternal drug abuse versus maternal depression: Vulnerability and resilience among school-age and adolescent offspring. Development and Psychopathology, 19(01). doi: 10.1017/S0954579407070113.

Metsch, L. R., Wolfe, H. P., Fewell, R., McCoy, C. B., Elwood, W. N., Wohler-Torres, B., et al. (2001). Treating Substance-Using Women and Their Children in Public Housing: Preliminary Evaluation Findings. Child Welfare, 80(2), 199-220. doi: Article.

Roberts, A. C., & Nishimoto, R. (2006). Barriers to engaging and retaining African-American post-partum women in drug treatment. Journal of Drug Issues, 36(1), 53. doi: Article.

Scott, T. J., Short, E. J., Singer, L. T., Russ, S. W., & Minnes, S. (2006). Psychometric properties of the Dominic Interactive Assessment: a computerized self-report for children. Assessment, 13(1), 16-26.

Suchman, N. E., & Luthar, S. S. (2001). The mediating role of parenting stress in methadone-maintained mothers' parenting. Parenting, Science and Practice, 1(4), 285-315.

Tandon, S. D., Parillo, K. M., Jenkins, C., & Duggan, A. K. (2005). Formative Evaluation of Home Visitors' Role in Addressing Poor Mental Health, Domestic Violence, and Substance Abuse Among Low-Income Pregnant and Parenting Women.. Maternal & Child Health Journal, 9(3), 273-283. doi: 10.1007/s10995-005-0012-8.
